# Supplementary material for: METTL14 modulates glycolysis to inhibit colorectal tumorigenesis in p53‐wild‐type cells
Source: EMBO Rep. 2023 Feb 16;24(4):e56325. doi: 10.15252/embr.202256325 (PMC10074077; doi:10.15252/embr.202256325)
Supplement: Supplementary file 1 — Appendix [file EMBR-24-e56325-s005.pdf]

Appendix for METTL14 loss modulates glycolysis reprogramming to drive p53-wild type colorectal tumorigenesis

Yichao Hou[1,†], Xintian Zhang[1,†], Han Yao[1,†], Lidan Hou[1], Qingwei Zhang[2], Enwei Tao[2], Shanshan Jiang[2], Yimeng Ren[2], Xialu Hong[2], Shiyuan Lu[2], Xiaoxu Leng[2], Xiaoqiang Zhu[3], Yile Xie[2], Yaqi Gao[2], Yu Liang[1], Ting Zhong[1], Bohan Long[1], Jing-Yuan Fang [2,*], Xiangjun Meng[1,**]

Table of content Page

Appendix Figure S1……………………………………………………………2
Appendix Figure S2……………………………………………………………3
Appendix Figure S3……………………………………………………………4
Appendix Figure S4……………………………………………………………5
Appendix Figure S5……………………………………………………………6
Appendix Figure S6……………………………………………………………7
Appendix Figure S7……………………………………………………………8
Appendix Figure S8……………………………………………………………9
Appendix Figure S9…………………………………………………………..10
Appendix Table S1……………………………………………………..……..11
Appendix Table S2…………………………………………………….…12, 13
Appendix Table S3…………………………………………………………14

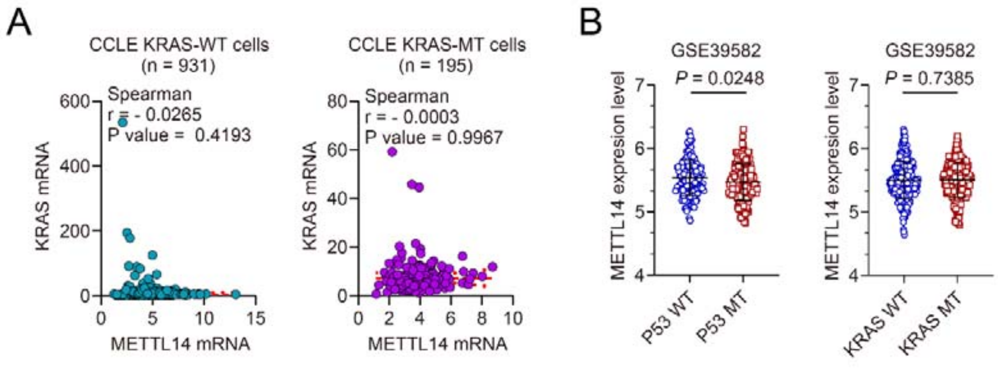

Appendix Figure S1. Correlations of METTL14 and p53 or KRAS in CRC cells or CRC tissues.

A Correlation of METTL14 and p53 mRNA levels in KRAS-wild type (KRAS-WT) and KRAS-mutant (KRAS-MT) CRC cell lines from CCLE database. Statistical significance was determined by analysis of the Pearson's correlation coefficient.

B. Expression analysis of METTL14 in p53-WT (Biological replicates n = 161) and p53-MT (Biological replicates n = 190) or KRAS-WT (Biological replicates n = 328) and KRAS-MT (Biological replicates n = 217) CRC tissues from GSE39582. Data are presented as mean ± SD. Statistical significance was determined by two-tailed Student's t test.

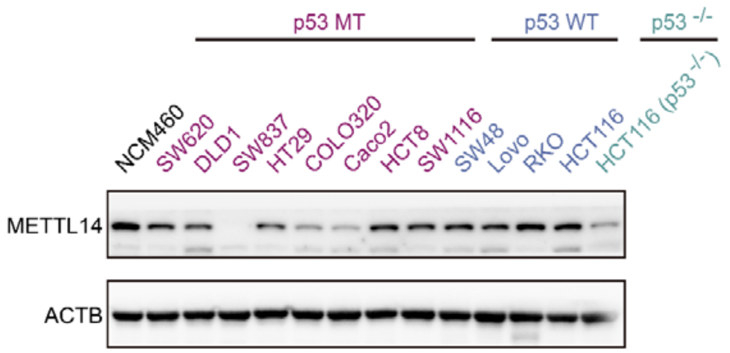

Appendix Figure S2. Western blot analysis of METTL14 protein level in normal (NCM460) and various CRC cell lines.

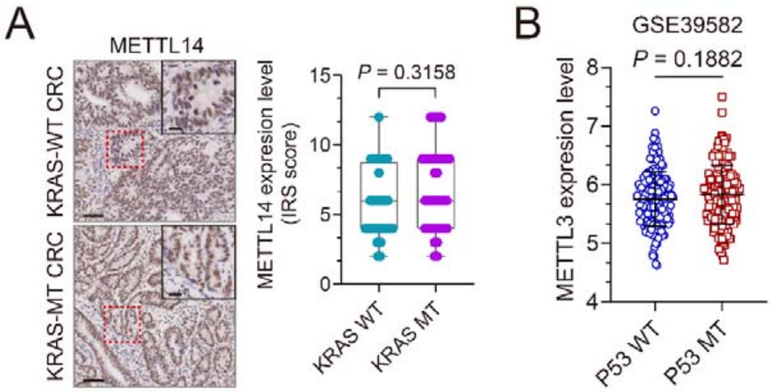

Appendix Figure S3. Expression of METTL3 in p53-WT and p53-MT CRC tissues.

A Representative IHC images, and statistical analysis of immunoreactive score (IRS) of METTL3 expression in p53-WT (Biological replicates n = 63) and p53-MT (Biological replicates n = 41) CRC samples. The insets show enlarged images of indicated p53-WT and p53-MT CRC tissues, respectively. Scale bars = 20 μm and 2 μm (inset). Statistical significance was determined by nonparametric Mann-Whitney test. The horizontal lines represent the median; the bottom and top of the boxes represent the 25% and 75% percentiles, respectively, and the vertical bars represent the range of the data.

B Expression of METTL3 in p53-WT (Biological replicates n = 161) and p53-MT (Biological replicates n =190) CRC tissues from GSE39582. Data are presented as mean ± SD. Statistical significance was determined by two-tailed Student's t test.

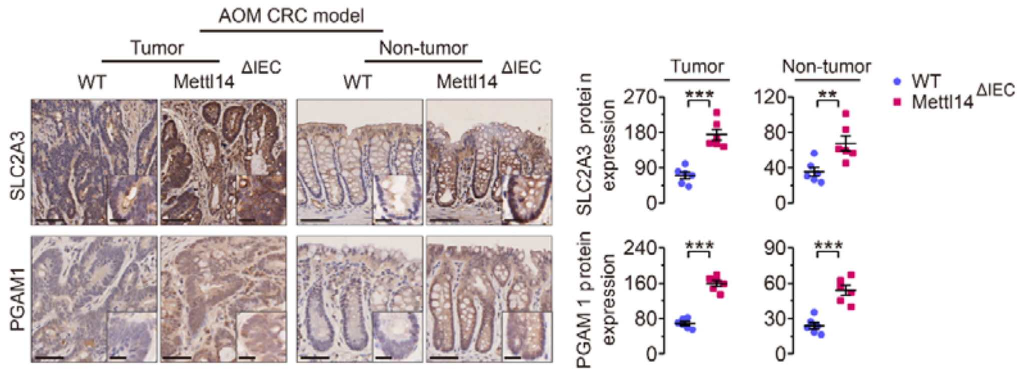

Appendix Figure S4. Representative IHC staining images and quantitative analysis of SLC2A3 and PGAM1 in tumor and non-tumor tissues from AOM-induced Mettl14$^{\Delta IEC}$ and Mettl14^{WT} mice CRC models.

The insets show enlarged images of tumor tissues and non-tumor tissues, respectively. Scale bars = 20 μm and 2 μm (inset). Data are presented as mean ± SD (Biological replicates n = 6; ****P* < 0.001). Statistical significance was determined by two-tailed Student's t test.

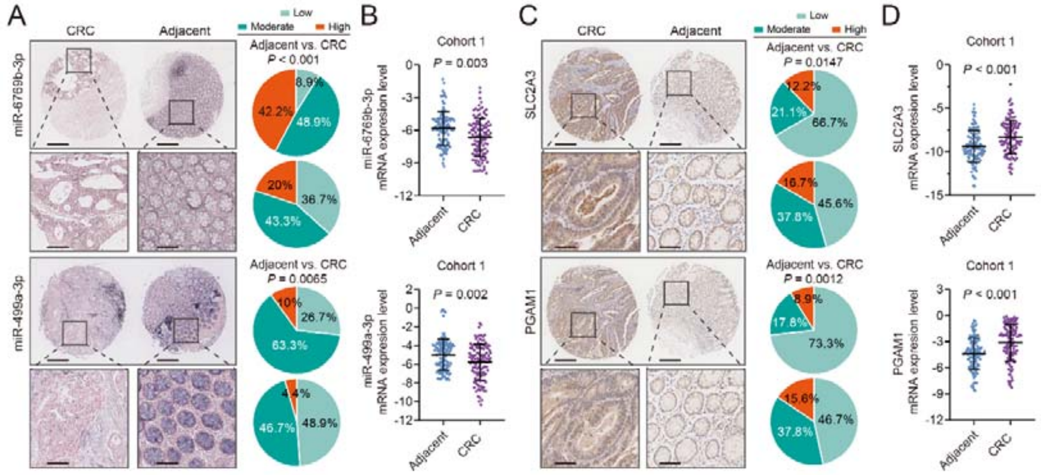

Appendix Figure S5. Expression levels of miR-6769b-3p/miR-499a-3p and SLC2A3/PGAM1 in CRC and adjacent tissues of patients.

A Representative IHC staining images and corresponding quantitative analysis of SLC2A3 and PGAM1 in CRC and adjacent tissues of patients from Cohort 2 (n = 90). Lower panels show enlarged images of indicated normal or CRC tissues. Scale bars = 200 μm (upper) and 40 μm (lower). Data are presented as mean ± SD. Percentages of low, moderate and high expression of SLC2A3 (upper) and PGAM1 (bottom) in CRC and adjacent tissues are shown as pie charts.

B qRT-PCR analysis of SLC2A3 (upper) and PGAM1(bottom) in CRC and adjacent tissues of patients from Jiuyuan Cohort 2 (n = 118). Data are presented as mean ± SD. Statistical significance was determined by two-tailed Student's t test.

C Representative ISH staining images and corresponding quantitative analysis of miR-6769b-3p and miR-499a-3p in CRC and adjacent tissues of patients from Cohort 2 (n = 90). Lower panels show enlarged images of indicated normal or CRC tissues. Scale bars = 200 μm (upper) and 40 μm (lower). Data are presented as mean ± SD. Percentages of low, moderate and high expression of miR-6769b-3p (upper) and miR-499a-3p (bottom) in CRC and adjacent tissues are shown as pie charts.

D qRT-PCR analysis of miR-6769b-3p (upper) and miR-499a-3p (bottom) in CRC and adjacent tissues of patients from Jiuyuan Cohort 2 (n = 118). Data are presented as mean ± SD. Statistical significance was determined by two-tailed Student's t test.

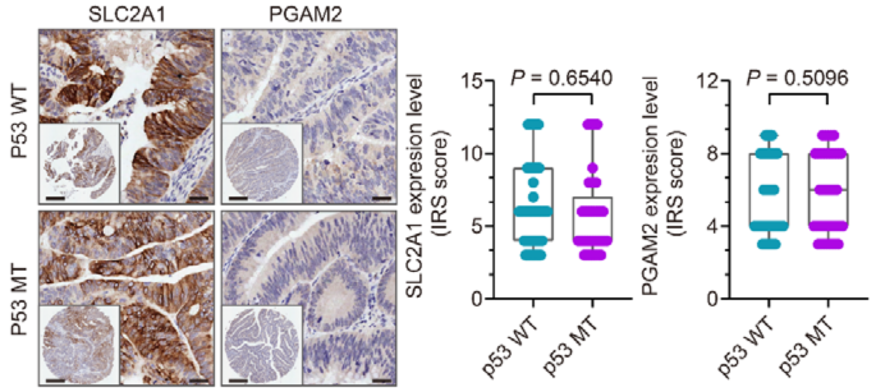

Appendix Figure S6. Expression levels of SLC2A1 and PGAM2 in p53-WT and p53-MT samples.

Representative IHC staining images and corresponding quantitative analysis of SLC2A3 and PGAM2 IRS in p53-WT (n = 63) and p53-MT (n = 41) samples from Cohort 3. The insets show enlarged images of indicated p53-WT and p53-MT CRC tissues, respectively. Scale bars = 200 μm and 20 μm (inset). Statistical significance was determined by nonparametric Mann-Whitney test. The horizontal lines represent the median; the bottom and top of the boxes represent the 25% and 75% percentiles, respectively, and the vertical bars represent the range of the data.

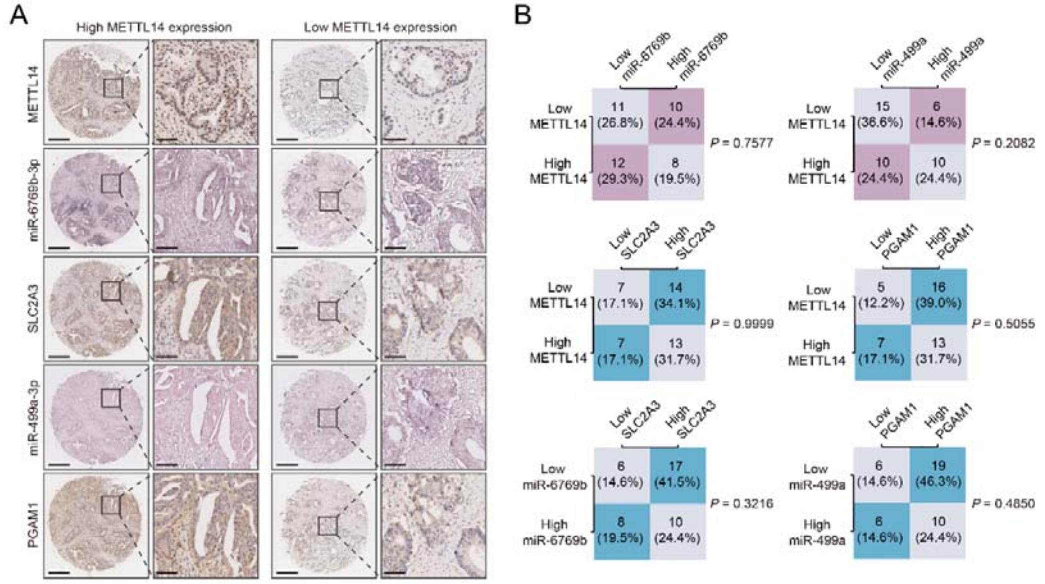

Appendix Figure S7. Correlation analysis of METTL14, SLC2A3, PGAM1, miR-6769b-3p and miR-499a-3p in p53-MT CRC tissues.

A Representative IHC and ISH images of METTL14, SLC2A3, PGAM1, miR-6769b-3p and miR-499a-3p in CRC tissues with higher or lower METTL14 expression in p53-MT (n = 41) samples from Cohort 3. The right panel show enlarged images of indicated p53-MT CRC tissues. Scale bars = 200 μm (left) and 20 μm (right).

B Statistical analysis of METTL14, SLC2A3, PGAM1, miR-6769b-3p and miR-499a-3p in CRC tissues with higher or lower METTL14 expression in p53-MT (n = 41) samples from Cohort 3. Statistical significance was determined by Fisher Exact test.

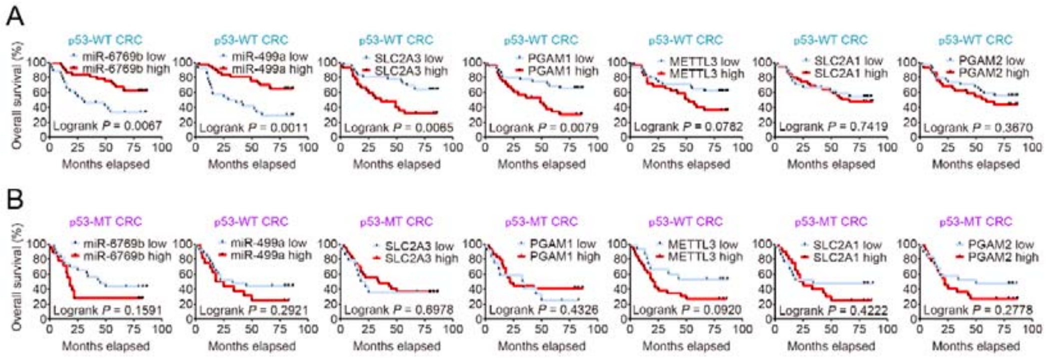

Appendix Figure S8. Survival analysis of CRC patients based on expression levels of miR-6769b-3p, miR-499a-3p, SLC2A3, PGAM1, METTL3, SLC2A1 and PGAM2.

A Kaplan-Meier survival curves of OS in CRC patients with wild type p53 (n = 63) from Cohort 3 database based on expression levels of miR-6769b-3p, miR-499a-3p, SLC2A3, PGAM1, METTL3, SLC2A1 and PGAM2. Statistical significance was performed by log-rank test.

B Kaplan-Meier survival curves of OS in CRC patients with mutant p53 (n = 41) from Cohort 3 database based on expression levels of miR-6769b-3p, miR-499a-3p, SLC2A3, PGAM1, METTL3, SLC2A1 and PGAM2. Statistical significance was performed by log-rank test.

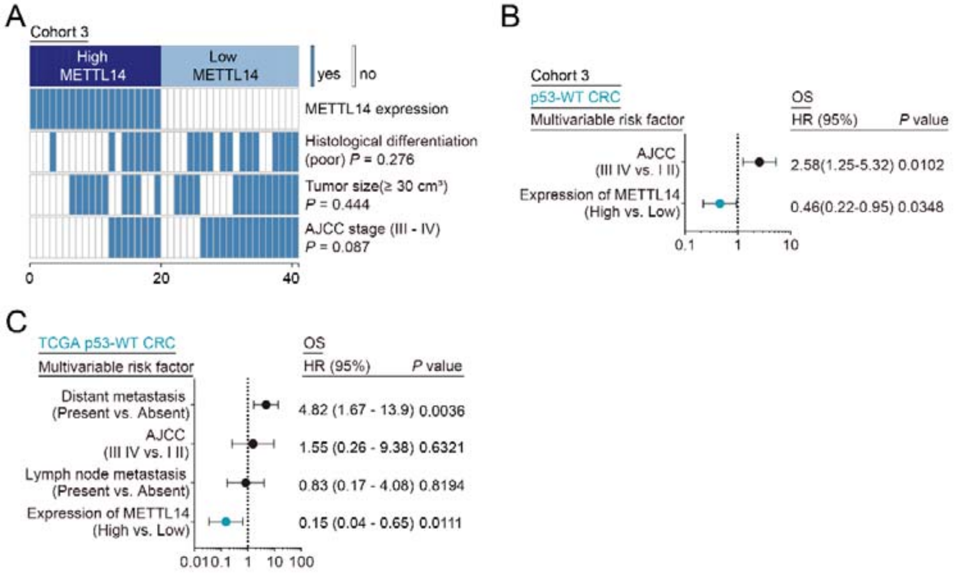

Appendix Figure S9. Correlation analysis of METTL14 and clinicopathological characteristics or OS in p53-MT patients.

A Comparison of clinicopathological characteristics between METTL14 high and low-expression tumors in p53-MT patients from Cohort 3 (n = 41). Statistical significance was performed by the Chi-square test.

B, C Multivariate Cox regression analysis of clinicopathologic factors for OS applied in p53-WT samples from Cohort 3 and TCGA CRC.

Appendix Table S1. The sequences of siRNAs, microRNA mimics and inhibitors used this study.

Gene	Sense	Antisense
METTL14-1	GGAUGAAGGAGAGACAGAUTT	AUCUGUCUCUCCUUCAUCCTT
METTL14-2	GCAGCACCUCGAUCAUUUATT	UAAAUGAUCGAGGUGCUGCTT
p53-1	GUAAUCUACUGGGACGGAATT	UUCCGUCCCAGUAGAUUACTT
p53-2	GAAGAAACCACUGGAUGGATT	UCCAUCCAGUGGUUUCUUCTT
ELK1-1	GCUGAGAGAGCAAGGCAAUTT	AUUGCCUUGCUCUCUCAGCTT
ELK1-2	CCCUUCUAUCAGCGUGGAUTT	AUCCACGCUGAUAGAAGGGTT
HIF-1α	GCUGAUUUGUGAACCCAUUTT	AAUGGGUUCACAAAUCAGCTT
MYC	GAGGAUAUCUGGAAGAAAUTT	AUUUCUUCCAGAUAUCCUCTT
SLC2A3-1	GUAGCUAAGUCGGUUGAAATT	UUUCAACCGACUUAGCUACTT
SLC2A3-2	CCUACAAAGUGCAGCCCUUTT	AAGGGCUGCACUUUGUAGGTT
PGAM1-1	GGCUAUGAGUUUGACAUCUTT	AGAUGUCAAACUCAUAGCCTT
PGAM1-2	CCUCAAUGAGCGGCACUAUTT	AUAGUGCCGCUCAUUGAGGTT
YTHDF1	GGCGUGUGUUCAUCAUCAATT	UUGAUGAUGAACACACGCCTT
YTHDF2	CCUACCAGAUGCAAUGUUUTT	AAACAUUGCAUCUGGUAGGTT
YTHDF3	AGCAGAGGAAACAGGCGAATT	UUCGCCUGUUUCCUCUGCUTT
YTHDC1	GGAGGAAGAAGAAGAAUAUTT	AUAUUCUUCUUCUUCCUCCTT
YTHDC2	GCGACUCAACAAUGGCAUATT	UAUGCCAUUGUUGAGUCGCTT
IGF2BP1	CCGCUUGUAAGAUGAUCUUTT	AAGAUCAUCUUACAAGCGGTT
IGF2BP2	GCGAAAGGAUGGUCAUCAUTT	AUGAUGACCAUCCUUUCGCTT
IGF2BP3	GCUGGAGCUUCAAUUAAGATT	UCUUAAUUGAAGCUCCAGCTT
hsa-miR-6769b-3p mimics	CCCUCUCUGUCCCACCCAUAG	AUGGGUGGGACAGAGAGGGUU
hsa-miR-1321 mimics	CAGGGAGGUGAAUGUGAU	CACAUUCACCUCCCUGUU
hsa-miR-7160-5p mimics	UGCUGAGGUCCGGGCUGUGCC	CACAGCCCGGACCUCAGCAUU
hsa-miR-380-3p mimics	UAUGUAAUAUGGUCCACAUCUU	GAUGUGGACCAUAUUACAUAUU
hsa-miR-3149 mimics	UUUGUAUGGAUAUGUGUGUGUA U	ACACACACAUAUCCAUACAAAU U
has-miR-499a-3p mimics	AACAUCACAGCAAGUCUGUGCU	CACAGACUUGCUGUGAUGUUUU
hsa-miR-6769b-3p inhibitor	CUAUGGGUGGGACAGAGAGGG	
hsa-miR-499a-3p inhibitor	AGCACAGACUUGCUGUGAUGUU	
hsa-mimics-NC	UUCUCCGAACGUGUCACGUTT	ACGUGACACGUUCGGAGAATT
hsa-inhibitor-NC	CAGUACUUUUGUGUAGUACAA	

Appendix Table S2. Sequence of primers used for real-time PCR.

Gene	Primer (Forward)	Primer (Reverse)
METTL14	AGTGCCGACAGCATTGGTG	GGAGCAGAGGTATCATAGGAAGC
p53	CAGCACATGACGGAGGTTGT	TCATCCAAATACTCCACACGC
p21	TGTCCGTCAGAACCCATGC	AAAGTCGAAGTTCCATCGCTC
ELK1	TCCCTGCTTCCTACGCATACA	GCTGCCACTGGATGGAAACT
ALDOA	ATGCCCTACCAATATCCAGCA	GCTCCCAGTGGACTCATCTG
ALDOB	GGCAGTTCCGAGAAATCCTCT	CTCCTTGGTCTAACTTGATTCCC
ALDOC	ATGCCTCACTCGTACCCAG	TTTCCACCCCAATTTGGCTCA
ENO1	TGGTGTCTATCGAAGATCCCTT	CCTTGGCGATCCTCTTTGG
ENO2	AGCCTCTACGGGCATCTATGA	TTCTCAGTCCCATCCAACTCC
ENO3	GGCTGGTTACCCAGACAAGG	TCGTACTTCCCATTGCGATAGAA
FBP1	CGCGCACCTCTATGGCATT	TTCTTCTGACACGAGAACACAC
FBP2	ACCCGCTACGTTATGGAAAAG	GCCGTCAGCATTGAGTTCAG
G6PC	ACTGGCTCAACCTCGTCTTTA	CGGAAGTGTTGCTGTAGTAGTCA
GAPDH	GGAGCGAGATCCCTCCAAAAT	GGCTGTTGTCATACTTCTCATGG
GAPDHS	TGTGGGCATCAATGGATTTGG	ACACCATGTATTCCGGGTCAAT
GCK	CCTGGGTGGCACTAACTTCAG	TAGTCGAAGAGCATCTCAGCA
GPI	CAAGGACCGCTTCAACCACTT	CCAGGATGGGTGTGTTTGACC
HIF-1α	GAACGTCGAAAAGAAAAGTCTCG	CCTTATCAAGATGCGAACTCACA
HK1	GCTCTCCGATGAAACTCTCATAG	GGACCTTACGAATGTTGGCAA
HK2	TGCCACCAGACTAAACTAGACG	CCCGTGCCCACAATGAGAC
HK3	GGACAGGAGCACCCTCATTTC	CCTCCGAATGGCATCTCTCAG
IGF1	GCTCTTCAGTTCGTGTGTGGA	GCCTCCTTAGATCACAGCTCC
LDHA	TTGACCTACGTGGCTTGGAAG	GGTAACGGAATCGGGCTGAAT
LDHAL6B	GGTGGGAGCGAATTTCCTATG	GCTTCTCGGAAGTGAAACGC
LDHB	TGGTATGGCGTGTGCTATCAG	TTGGCGGTCACAGAATAATCTTT
LDHC	AGAACATGGTGATTCTAGTGTGC	ACAGTCCAATAGCCCAAGAGG
MPC1	ACTATGTCCGAAGCAAGGATTTC	CGCCCACTGATAATCTCTGGAG
MPC2	TACCACCGGCTCCTCGATAAA	TATCAGCCAATCCAGCACACA
MYC	TCCCTCCACTCGGAAGGAC	CTGGTGCATTTTCGGTTGTTG
PFKL	GTACCTGGCGCTGGTATCTG	CCTCTCACACATGAAGTTCTCC
PFKM	GGTGCCCGTGTCTTCTTTGT	AAGCATCATCGAAACGCTCTC
PFKP	CGCCTACCTCAACGTGGTG	ACCTCCAGAACGAAGGTCCTC
PGAM1	TCTGGAGGCGCTCCTATGAT	TCTGTGAGGTCTGCATACCTG
PGAM2	AGAAGCACCCCTACTACAACTC	TCTGGGGAACAATCTCCTCGT
PGK1	TGGACGTTAAAGGGAAGCGG	GCTCATAAGGACTACCGACTTGG
PGK2	AAACTGGATGTTAGAGGGAAGCG	GGCCGACCTAGATGACTCATAAG
PKLR	TCAAGGCCGGGATGAACATTG	CTGAGTGGGGAACCTGCAAAG
PKM	ATGTCGAAGCCCCATAGTGAA	TGGGTGGTGAATCAATGTCCA
SLC2A1	GGCCAAGAGTGTGCTAAAGAA	ACAGCGTTGATGCCAGACAG

SLC2A3	GCTGGGCATCGTTGTTGGA	GCACTTTGTAGGATAGCAGGAAG
SLC2A5	GAGGCTGACGCTTGTGCTT	CCACGTTGTACCCATACTGGA
YTHDF1	ATGACAATGACTTTGAGCCCTA	AGGGAGTAAGGAAATCCAATGG
YTHDF2	ACTTCTCAGCATGGGGAAATAA	TATTCATGCCAGGAGCCTTATT
YTHDF3	GCTCCACCAACCCAACCAGTTC	CTGAGGTCCTTGTTGCTGCTGTG
YTHDC1	AGTGACTCTGGTTCTGAATCTG	CTGGTTTGATCTTTTCGGACAG
YTHDC2	GAGAATTGGGCTGTCGTTAAAG	TGAAGCAGGATGAAATCGTACT
IGF2BP1	GATGAAGGCCATCGAAACTTTC	GGGGTGGAATATTTCGGATTTG
IGF2BP2	GATGAACAAGCTTTACATCGGG	GATTTTCCCATGCAATTCCACT
IGF2BP3	GAGGCGCTTTCAGGTAAAATAG	AATGAGGCGGGATATTTCGTAT
ACTB	AGAGCCTCGCCTTTGCCGATCC	CTGGGCCTCGTCGCCCACATA
pre-SLC2A3	CAAGGTCACTTCTCCTGCTGA	GCCAACTTCTACGCCATTCC
pre-PGAM1	CACAACCAGGCACAACTTTACA	GACCACCAACAAACTACAGCAA
pre-miR-6769b	CTTCCTGGTGGGTGGGGA	CTATGGGTGGGACAGAGAGGG
pre-miR-499a	GCGGCTGTTAAGACTTGCA	GCACAGACTTGCTGTGATGTTC
pri-miR-6769b	GCTCCAGGCTGAGCCACTT	TCCATCACCAGTACCTTCTGCC
pri-miR-499a	ACAAGGTAAGGCCCCATCTG	AGCGTAGGGACGGGAAGC

Appendix Table S3. Sequence of primers used for Chip-PCR.

Gene	Primer (Forward)	Primer (Reverse)
METTL14 Promoter #1 (-3000~-2701)	TTAGGTATTTATACTCTCGAACCTAGACA	CCAGCTACTTGGGAGGCT
METTL14 Promoter #2 (-2700~-2401)	GATTACAGGCGCCCAACAC	CACATTATAACTTATGCATCTCTCCCCA
METTL14 Promoter #3 (-2400~-2101)	GATCTCTCTTTACTTCCTTAAATTAAGTGC	TTTACAAAACTGTTGGAGTTGGGC
METTL14 Promoter #4 (-2100~-1801)	TATTACATGGAAGTACATATCTGCATCAGT	CCAGTTGGAGCAGCCCC
METTL14 Promoter #5 (-1800~-1501)	CTCTAGACTTTGTAAGGGACATAGTCA	ATGAGTTTACTGTCTAATGGAGGATTTTTC
METTL14 Promoter #6 (-1500~-1201)	AAAGAAAAACCGACTTTAGTTTTTAATCTTTG	TGAAAGACAATTTTGAACAAGTAGTCCT
METTL14 Promoter #7 (-1200~-901)	GACCTAGTTCTTCGGAAATACATTATAATTA	TATTTGAGTTATTACCATTATTATTCACCACAC
METTL14 Promoter #8 (-900~-601)	AGACTTTGGCCTTTGCTGC	CCACTCTTCCCCACACCTTAC
METTL14 Promoter #9 (-600~-301)	GCAGATGTTGTGGCTGTTACC	CCACTCTTCCCCACACCTTAC
METTL14 Promoter #10 (-300~50)	CTGTCATCTGTGGAATCAATTGAAAAG	GAGTCATAGCCACAAAATTGAGATTATG
