# Supplementary material for: METTL14 modulates glycolysis to inhibit colorectal tumorigenesis in p53‐wild‐type cells
Source: EMBO Rep. 2023 Feb 16;24(4):e56325. doi: 10.15252/embr.202256325 (PMC10074077; doi:10.15252/embr.202256325)
Supplement: Supplementary file 2 — Expanded View Figures PDF [file EMBR-24-e56325-s011.pdf]

Expanded View Figures

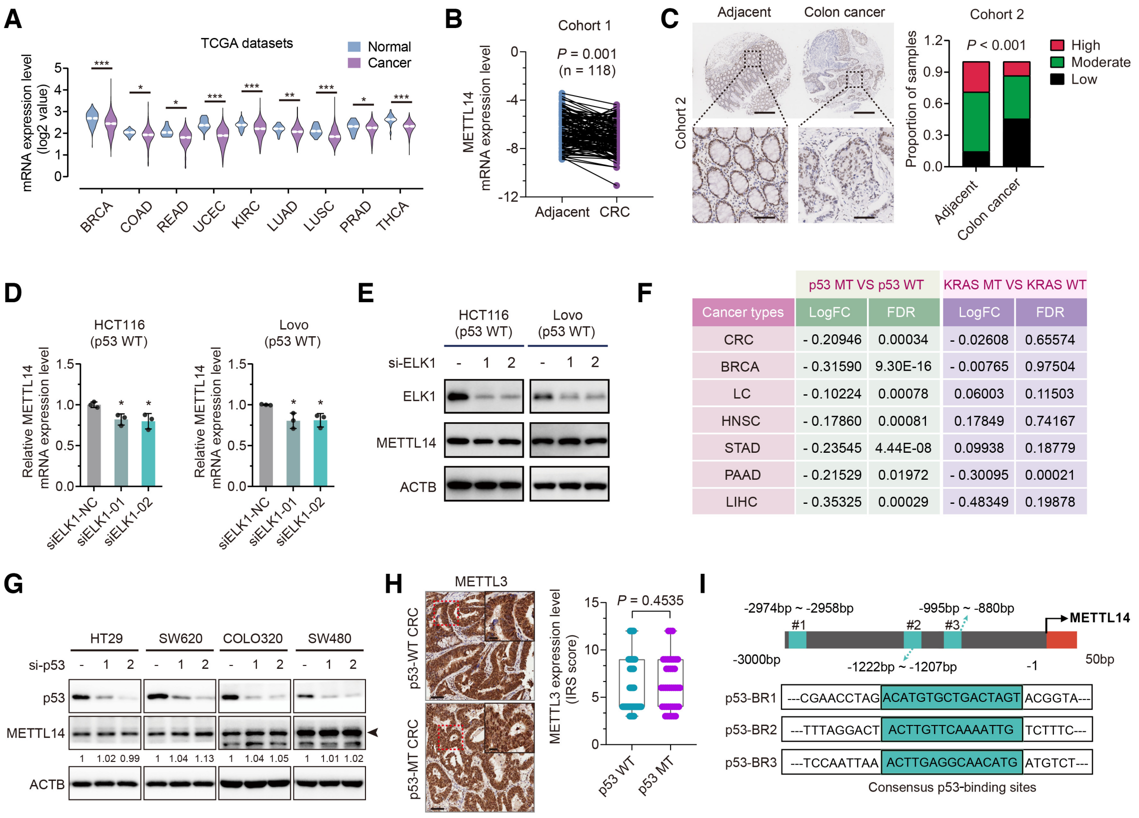

Figure EV1. Wild-type p53 activates METTL14 expression in CRC cell lines.

A METTL14 mRNA levels between tumor and normal tissues in the TCGA database (**P* < 0.05, ***P* < 0.01, ****P* < 0.001). Breast cancer (BRCA—normal tissues *n* = 113, cancer tissues *n* = 1,104); colon cancer (COAD—normal tissues *n* = 41, cancer tissues *n* = 471); rectal cancer (READ—normal tissues *n* = 10, cancer tissues *n* = 167); endometrioid cancer (UCEC—normal tissues *n* = 35, cancer tissues *n* = 548); kidney clear cell carcinoma (KIRC—normal tissues *n* = 72, cancer tissues *n* = 535); lung adenocarcinoma (LUAD—normal tissues *n* = 59, cancer tissues *n* = 526); lung squamous cell carcinoma (LUSC—normal tissues *n* = 49, cancer tissues *n* = 501); prostate cancer (PRAD—normal tissues *n* = 52, cancer tissues *n* = 499); and thyroid cancer (THCA—normal tissues *n* = 58, cancer tissues *n* = 510).

B qRT–PCR determination of the METTL14 level in CRC and adjacent normal tissues from Cohort 1 (*n* = 118).

C Representative IHC staining images and statistical analysis of METTL14 in tumor and para-tumor tissues from Cohort 2 (*n* = 90). Lower panels show enlarged images of indicated normal or CRC tissues. Scale bars = 200 μm (upper) and 40 μm (lower).

D qRT–PCR analysis of the mRNA levels of METTL14 after transfection with control or ELK1 siRNAs with 48 h treatment in HCT116 and Lovo cells. Data are presented as mean ± SD (biological replicates, *n* = 3; **P* < 0.05).

E Western blot analysis of METTL14 and ELK1 protein levels after transfection with control or ELK1 siRNAs with 48 h treatment in HCT116 and Lovo cells.

F Expression analysis of METTL14 in p53-WT and p53-MT or KRAS-WT and KRAS-MT tumor tissues from in TCGA. Lung Cancer (LC); Head and Neck Cancer (HNSC); Stomach Cancer (STAD); Liver Cancer (LIHC) and Pancreatic Cancer (PAAD).

G HT29 (p53R273H), SW620 (p53R273H), COLO320 (p53R248W) and SW480 (p53R273H/P309S) cells were transfected with control or p53 siRNAs. Forty-eight hours after transfection, total protein was then analyzed by western blot analysis. Arrows indicate METTL14 protein.

H Representative IHC images, and statistical analysis of immunoreactive score (IRS) of METTL3 expression in p53-WT (*n* = 63) and p53-MT (*n* = 41) CRC samples from Cohort 3. The insets show enlarged images of indicated p53-WT and p53-MT CRC tissues, respectively. Scale bars = 20 μm and 2 μm (inset). The horizontal lines represent the median; the bottom and top of the boxes represent the 25 and 75% percentiles, respectively, and the vertical bars represent the range of the data.

I Schematic illustration of putative p53-binding site in METTL14 gene promoter.

Data information: For (A, B, D, and F), statistical significance was determined by a two-tailed Student's *t*-test. For (H), statistical significance was determined by the non-parametric Mann–Whitney test. For (C), statistical significance was determined by the χ^2 test. ACTB was used as a loading control.

 © 2023 The Authors

Figure EV2. METTL14 inhibits p53-WT CRC progression.

A, B qRT–PCR and Western blot validation of the METTL14 knockdown efficiency by stable transfection with shNC or shMETTL14 in p53-WT (HCT116 and Lovo) and p53-MT (HT29 and SW620) cells. Data are presented as mean ± SD (biological replicates $n = 3$; ***$P < 0.001$).

C Cell viability assay of CRC cells stably transfected with lentivirus carrying control shRNA (shNC) or METTL14 shRNA (shMETTL14) for indicated time (0, 24, 48, and 96 h). Data are presented as mean ± SD (biological replicates, $n = 6$; ***$P < 0.001$, ns, no significance).

D Representative HE and IHC staining images and quantitative analysis of Ki-67 in subcutaneous tumors from nude mice in Fig 2B and C. Scale bars = 10 μm. Data are presented as mean ± SD (biological replicates, $n = 6$; ns = no significance, ***$P < 0.001$).

E Schematic diagrams of generation of Villin-Cre^{+}/Mettl14$^{FL/FL}$ conditional knockout mice.

F Representative IHC staining images of METTL14 in organs other than colorectum, including lung, liver, spleen, kidney, stomach, and heart, from Mettl14$^{\Delta IEC}$ ($n = 34$) and Mettl14^{WT} ($n = 38$) mice. The insets show enlarged images of indicated tissues. Scale bars = 400 μm and 10 μm (inset).

G Gross appearance of Mettl14$^{\Delta IEC}$ ($n = 13$, $n = 21$) and Mettl14^{WT} ($n = 14$, $n = 24$) C57BL/6 mice from AOM/DSS-induced (Left) and AOM-induced (Right) CRC model.

H The body weight changes during the course of acute colitis with DSS in Mettl14$^{\Delta IEC}$ ($n = 13$) and Mettl14^{WT} ($n = 14$) mice were recorded and expressed as the ratio relative to the initial weight before DSS treatment. Data are expressed as mean ± SD.

I Representative morphology of spleens in Mettl14$^{\Delta IEC}$ ($n = 13$, $n = 21$) and Mettl14^{WT} ($n = 14$, $n = 24$) mice extracted from AOM/DSS-induced (Left) and AOM-induced (Right) CRC models.

J Representative HE staining images of colorectum of Mettl14$^{\Delta IEC}$ ($n = 21$) and Mettl14^{WT} ($n = 24$) mice from AOM/DSS-induced (Left) and AOM-induced (Right) CRC model, showing representative inflammation. Lower panels show the magnified images of the indicated regions. Scale bars = 400 μm (upper) and 100 μm (lower).

Data information: For (A, C, D, and H), statistical significance was determined by a two-tailed Student's *t*-test. ACTB was used as a loading control.

© 2023 The Authors

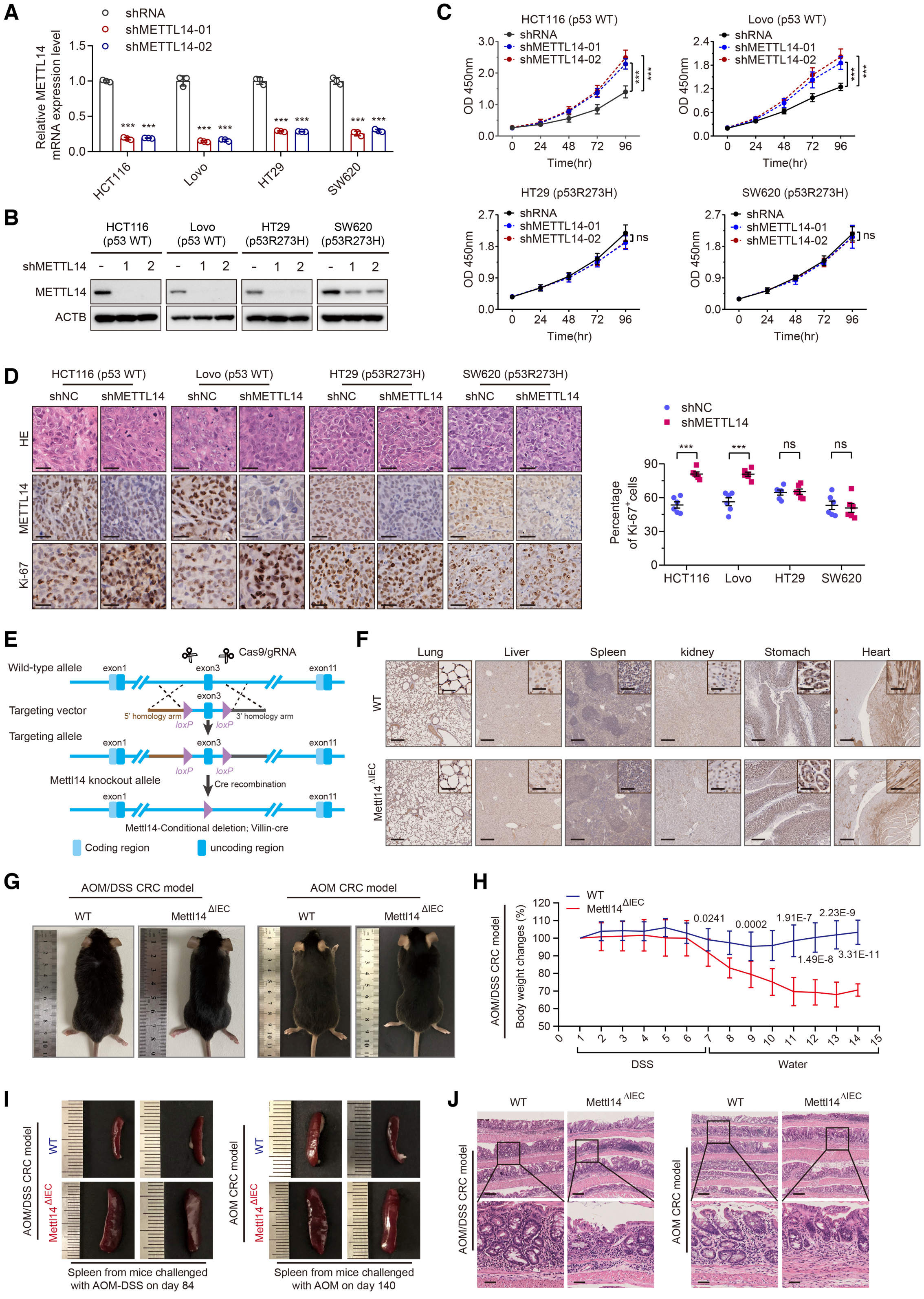

Figure EV2.

 © 2023 The Authors

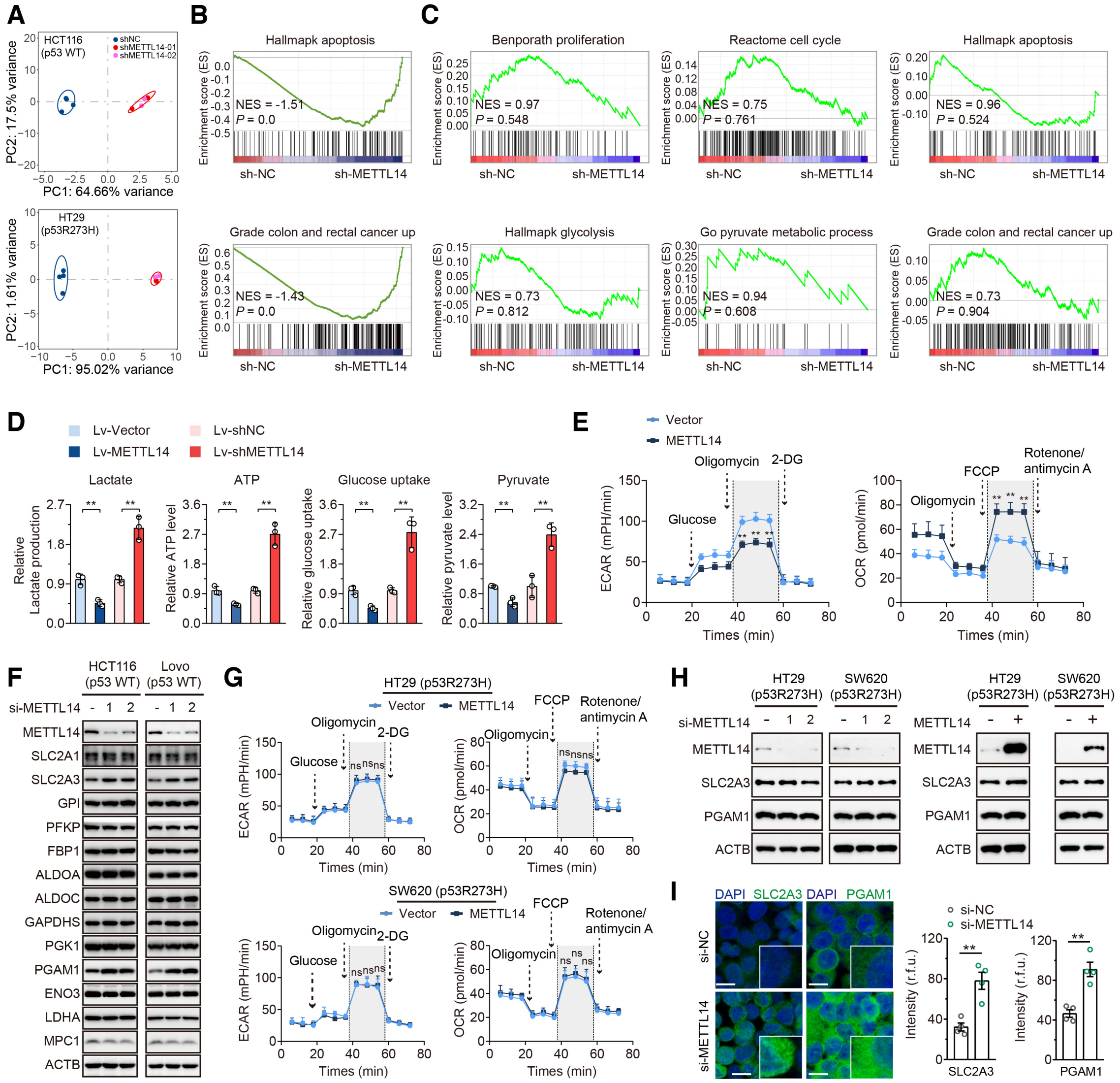

Figure EV3. METTL14 suppresses glycolysis of p53-WT CRC cells by targeting SLC2A3 and PGAM1.

A Principal component analysis of gene expression profiles of shNC ($n = 4$), shMETTL14-01 ($n = 2$), and shMETTL14-02 ($n = 2$) shRNA-expressing p53-WT HCT116 and p53-MT HT29 cells.

B, C Overview of GSEA used to identify the differential gene profiles in HCT116 (p53-WT) and HT29 (p53-MT) cells stably transfected with shNC and shMETTL14, respectively.

D Lactate production, ATP level, glucose uptake, and pyruvate level were determined in Lovo (p53-WT) cells stably transfected with Lv-vector or Lv-METTL14, or with shNC or shMETTL14. Data are presented as mean ± SD (biological replicates, $n = 3$; $^{**}P < 0.01$).

E ECAR and OCR were measured in Lovo (p53-WT) cells stably transfected Lv-vector or Lv-METTL14. Data are presented as mean ± SD (biological replicates, $n = 3$; $^{**}P < 0.01$).

F Western blot analysis of glycolytic gene expression in HCT116 and Lovo (p53-WT) cells transfected with control siRNA or METTL14 siRNAs for 48 h.

G ECAR and OCR were measured in p53-MT (HT29 and SW620) cells stably transfected with vector or METTL14. Data are presented as mean ± SD (biological replicates, $n = 3$; ns, no significance).

H Western blot analysis of METTL14, SLC2A3, and PGAM1 protein levels in p53-MT (HT29 and SW620) cells transfected with control siRNA or METTL14 siRNAs, and empty vector or METTL14 plasmid.

I Representative immunofluorescence (IF) staining and quantitative analysis of SLC2A3 (green) and PGAM1 (green) proteins in HCT116 (p53-WT) cells transfected with control or METTL14 siRNA. Nuclei were stained with DAPI (blue). The insets show enlarged images of CRC cells. Scale bars = 10 μm. Data are presented as mean ± SD (biological replicates, $n = 4$; $^{**}P < 0.01$).

Data information: For (D, E, G, I,), statistical significance was determined by a two-tailed Student's t-test. ACTB was used as a loading control.
Source data are available online for this figure.

Figure EV4. METTL14 decreases SLC2A3 and PGAM1 levels by m^{6}A-YTHDF2-mediated pri-miR-6769b and pri-miR-499a processing.

- A Schematic illustration of the protocol for screening miRNAs that are regulated by METT14 and simultaneously target SLC2A3 and PGAM1 using miRNA Microarray, TargetScan (http://www.targetscan.org/vert_71/) and miRDB (http://mirdb.org/miRDB/) database.
- B qRT–PCR analysis of the SLC2A3 and PGAM1 levels in HCT116 and Lovo (p53-WT) cells transfected with miR-6769b-3p and miR-499a-3p mimics and corresponding inhibitors. Data are presented as mean ± SD (biological replicates, $n = 3$; $^{**}P < 0.01$, $^{***}P < 0.001$).
- C Heatmap of known METTL14 target miRNAs in p53-WT cells identified by miRNA microarrays using HT29 cells stably infected with lentivirus carrying METTL14 overexpression or control vector.
- D qRT–PCR analysis of the miR-6769b-3p and miR-499a-3p levels in stably transfected vector and METTL14 HT29 (p53-MT) cells. Data are presented as mean ± SD (biological replicates, $n = 3$; ns = no significance).
- E Western blot analysis of SLC2A3 and PGAM1 in HT29 and SW620 (p53-MT) cells transfected with control or miRNA mimics and corresponding inhibitors.
- F Schematic diagram of luciferase reporters expressing wild-type or mutant SLC2A3 3′UTRs and wild-type or mutant PGAM1 3′UTRs predicted by TargetScan and miRDB database.
- G qRT–PCR was performed to determine the pri-miR-6769b and pri-miR-499a levels in HCT116 and Lovo (p53-WT) cells transfected with control siRNA or METTL14 siRNAs for 48 h. Data are presented as mean ± SD (biological replicates, $n = 3$; $^{**}P < 0.01$).
- H qRT–PCR was performed to determine the miR-6769b-3p/miR-499a-3p and pri-miR-6769b/pri-miR-499a levels in p53-MT (HT29 and SW620) cells transfected with control or METTL14 siRNAs for 48 h. Data are presented as mean ± SD (biological replicates, $n = 3$; ns = no significance).
- I The sequences of pri-miR-6769b/pre-miR-6769b/miR-6769b-3p and pri-miR-499a/pre-miR-499a/miR-499a-3p are presented and highlighted by different colors. The m^{6}A sites were predicted by SRAMP.
- J qRT–PCR analysis of the pri-miR-6769b/pre-miR-6769b/miR-6769b-3p levels in HCT116 cells transfected with wild-type or mutant pri-miR-6769b plasmids. Data are presented as mean ± SD (biological replicates, $n = 3$, $^{**}P < 0.01$).
- K qRT–PCR analysis of the pri-miR-499a/pre-miR-499a/miR-499a-3p levels in HCT116 cells transfected with wild-type or mutant pri-miR-499a plasmids. Data are presented as mean ± SD (biological replicates, $n = 3$, $^{**}P < 0.01$).
- L qRT–PCR analysis of the pri-miR-6769b/pri-miR-499a levels in HCT116 cells transfected with empty vector, mutant METTL14 (MT-METTL14) or wild-type METTL14 (WT-METTL14) plasmids for 48 h. Data are presented as mean ± SD (biological replicates, $n = 3$).
- M Western blot and m^{6}A dot blot analyses of METTL14, SLC2A3, PGAM1, and global m^{6}A levels in HCT116 cells transfected with empty vector, MT-METTL14 or WT-METTL14 plasmids for 48 h.

Data information: For (B–D, G, H, J–L), statistical significance was determined by a two-tailed Student's *t*-test. ACTB and methylene blue were used as a loading control.

 © 2023 The Authors

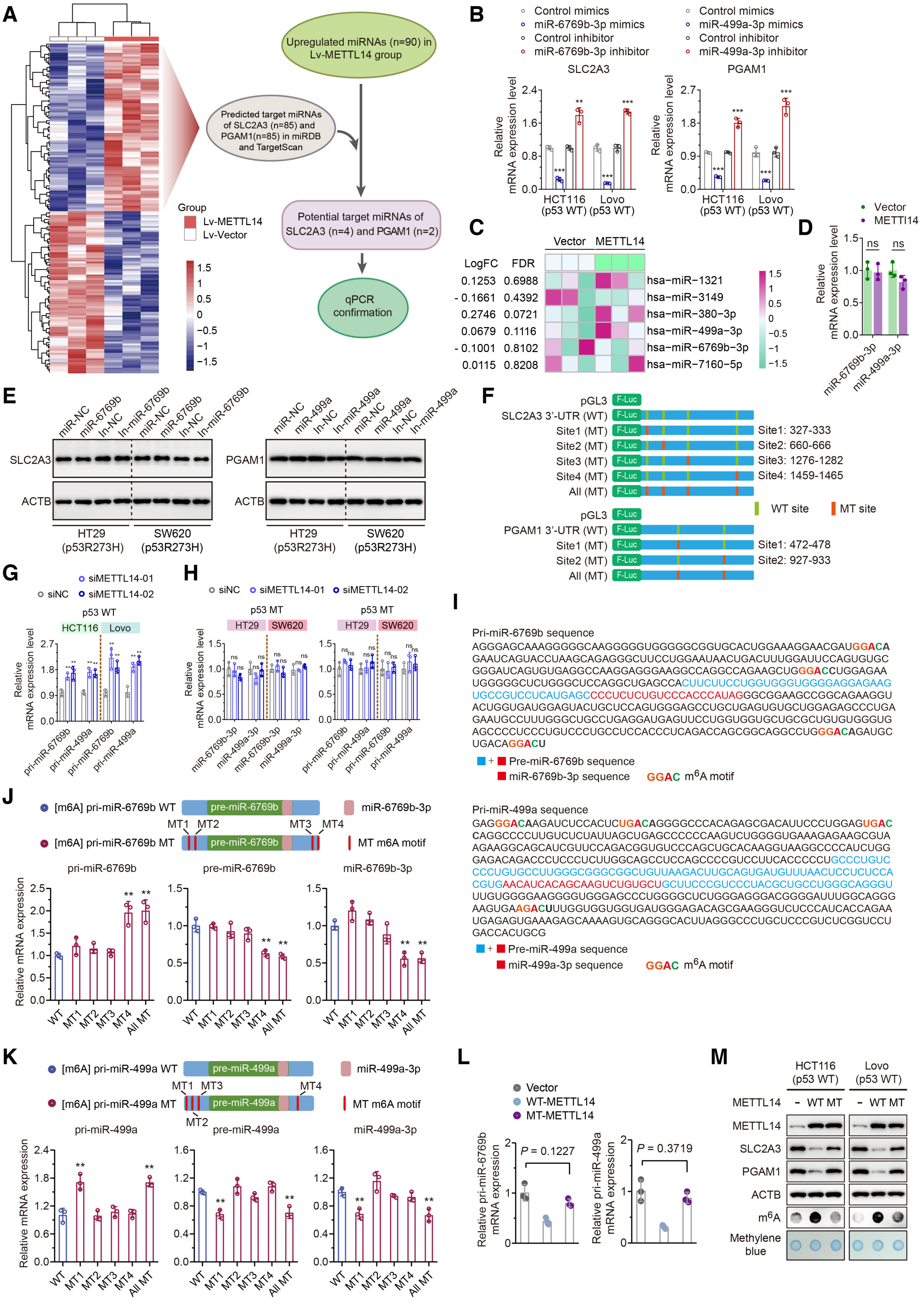
A
Upregulated miRNAs (n=90) in Lv-METTL14 group
Predicted target miRNAs of SLC2A3 (n=85) and PGAM1(n=85) in miRDB and TargetScan
Potential target miRNAs of SLC2A3 (n=4) and PGAM1 (n=2)
qPCR confirmation
Group
Lv-METTL14
Lv-Vector
B
Control mimics
miR-6769b-3p mimics
Control inhibitor
miR-6769b-3p inhibitor
Control mimics
miR-499a-3p mimics
Control inhibitor
miR-499a-3p inhibitor
SLC2A3
PGAM1
Relative mRNA expression level
HCT116 (p53 WT)
Lovo (p53 WT)
C
Vector
METTL14
LogFC
FDR
0.1253 0.6988 hsa−miR−1321
- 0.1661 0.4392 hsa−miR−3149
0.2746 0.0721 hsa−miR−380−3p
0.0679 0.1116 hsa−miR−499a−3p
- 0.1001 0.8102 hsa−miR−6769b−3p
0.0115 0.8208 hsa−miR−7160−5p
D
Vector
Mettl14
Relative mRNA expression level
miR-6769b-3p
miR-499a-3p
E
miR-NC
miR-6769b
In-NC
In-miR-6769b
miR-499a
In-miR-499a
SLC2A3
PGAM1
ACTB
HT29 (p53R273H)
SW620 (p53R273H)
F
pGL3
SLC2A3 3'-UTR (WT)
Site1 (MT)
Site2 (MT)
Site3 (MT)
Site4 (MT)
All (MT)
Site1: 327-333
Site2: 660-666
Site3: 1276-1282
Site4: 1459-1465
WT site
MT site
PGAM1 3'-UTR (WT)
Site1: 472-478
Site2: 927-933
F-Luc
G
siNC
siMETTL14-01
siMETTL14-02
p53 WT
HCT116
Lovo
pri-miR-6769b
pri-miR-499a
H
p53 MT
HT29
SW620
miR-6769b-3p
miR-499a-3p
I
Pri-miR-6769b sequence
Pre-miR-6769b sequence
miR-6769b-3p sequence
GGAC m6A motif
Pri-miR-499a sequence
Pre-miR-499a sequence
miR-499a-3p sequence
J
[m6A] pri-miR-6769b WT
[m6A] pri-miR-6769b MT
pre-miR-6769b
miR-6769b-3p
MT m6A motif
MT1 MT2 MT3 MT4
Relative mRNA expression
WT MT1 MT2 MT3 MT4 All MT
K
[m6A] pri-miR-499a WT
[m6A] pri-miR-499a MT
pre-miR-499a
miR-499a-3p
L
Vector
WT-METTL14
MT-METTL14
P = 0.1227
P = 0.3719
Relative pri-miR-6769b mRNA expression
Relative pri-miR-499a mRNA expression
M
HCT116 (p53 WT)
Lovo (p53 WT)
METTL14
SLC2A3
PGAM1
ACTB
m6A
Methylene blue

Figure EV4.

Figure EV5. METTL14 attenuates the development of p53-WT CRC by repressing glycolysis through miR-6769b-3p/SLC2A3 and miR-499a-3p /PGAM1 axis.

A Lactate production, ATP level, glucose uptake, and pyruvate level were determined in HT29 (p53-MT) cells treated with control, miR-6769b-3p mimics, or inhibitor for 48 h. Data are presented as mean ± SD (biological replicates, $n = 3$; ns = no significance).

B Lactate production, ATP level, glucose uptake, and pyruvate level were determined in HT29 (p53-MT) cells treated with control, miR-499a-3p mimics, or inhibitor for 48 h. Data are presented as mean ± SD (biological replicates, $n = 3$; ns = no significance).

C ECAR and OCR were measured in HT29 (p53-MT) cells transfected with control or miR-6769b-3p mimics for 48 h. Data are presented as mean ± SD (biological replicates, $n = 3$; ns = no significance).

D ECAR and OCR were measured in HT29 (p53-MT) cells transfected with control or miR-499a-3p mimics for 48 h. Data are presented as mean ± SD (biological replicates, $n = 3$; ns = no significance).

E Cell viability assay was performed in HCT116 and Lovo (p53-WT) cells stably transfected with shNC or shMETTL14, or in the stable transfectants with shMETTL14 transfected with control, miR-6769b-3p or miR-499a-3p mimics. Data are presented as mean ± SD (biological replicates, $n = 5$, ***$P < 0.001$).

F Colony formation assay was performed in HCT116 and Lovo (p53-WT) cells stably transfected with shNC or shMETTL14, or in the stable transfectants with shMETTL14 transfected with control, miR-6769b-3p or miR-499a-3p mimics. Data are presented as mean ± SD (biological replicates, $n = 3$, **$P < 0.01$).

G Colony formation assay was performed in p53-WT (HCT116 and Lovo) and p53-MT (HT29 and SW620) cells stably transfected with control, shSLC2A3 or shPGAM1. Data are presented as mean ± SD (biological replicates, $n = 3$, ns = no significance, *$P < 0.05$, **$P < 0.01$).

H Western blot analysis of SLC2A3 and PGAM1 in p53-WT and p53-MT CRC cell lines.

I qRT–PCR analysis of miR-6769b-3p and miR-499a-3p mRNA level in p53-WT and p53-MT CRC cell lines. Data are presented as mean ± SD (biological replicates, $n = 3$, ns = no significance, *$P < 0.05$, **$P < 0.01$, ***$P < 0.001$). The dotted line represents basal mRNA expression of miR-6769b-3p and miR-499a-3p in RKO cells, acting as a control.

J Representative images of tumors and analysis in nude mice intervened with control, shSLC2A3 or shPGAM1 expression p53-WT (HCT116 and Lovo) cells. Data are presented as mean ± SD (biological replicates, $n = 7$).

K Representative images of tumors and analysis in nude mice intervened with control, shSLC2A3 or shPGAM1 expression p53-MT (HT29 and SW620) cells. Data are presented as mean ± SD (biological replicates, $n = 7$).

Data information: For (A–G, J, K), statistical significance was determined by a two-tailed Student's *t*-test. ACTB was used as a loading control.

 © 2023 The Authors

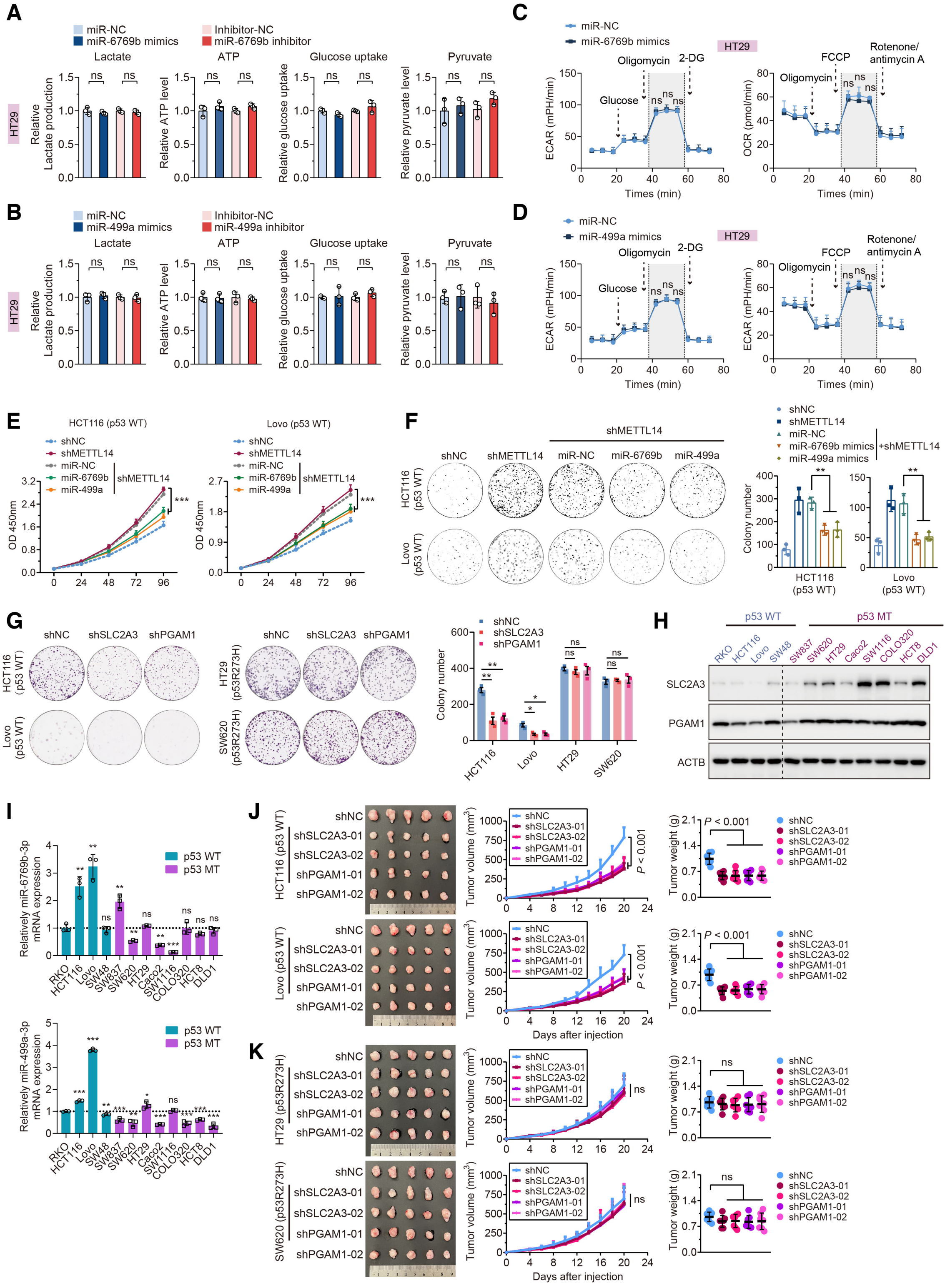
A
miR-NC
miR-6769b mimics
Inhibitor-NC
miR-6769b inhibitor
Lactate
ATP
Glucose uptake
Pyruvate
HT29
B
miR-NC
miR-499a mimics
Inhibitor-NC
miR-499a inhibitor
C
HT29
D
HT29
E
HCT116 (p53 WT)
Lovo (p53 WT)
F
shNC
shMETTL14
miR-NC
miR-6769b
miR-499a
G
shNC
shSLC2A3
shPGAM1
H
p53 WT
p53 MT
SLC2A3
PGAM1
ACTB
I
p53 WT
p53 MT
J
K
Days after injection

Figure EV5.
