# Supplementary figures and images for: METTL14 modulates glycolysis to inhibit colorectal tumorigenesis in p53‐wild‐type cells
Source: EMBO Rep. 2023 Feb 16;24(4):e56325. doi: 10.15252/embr.202256325 (PMC10074077; doi:10.15252/embr.202256325)

Appendix Fig 2

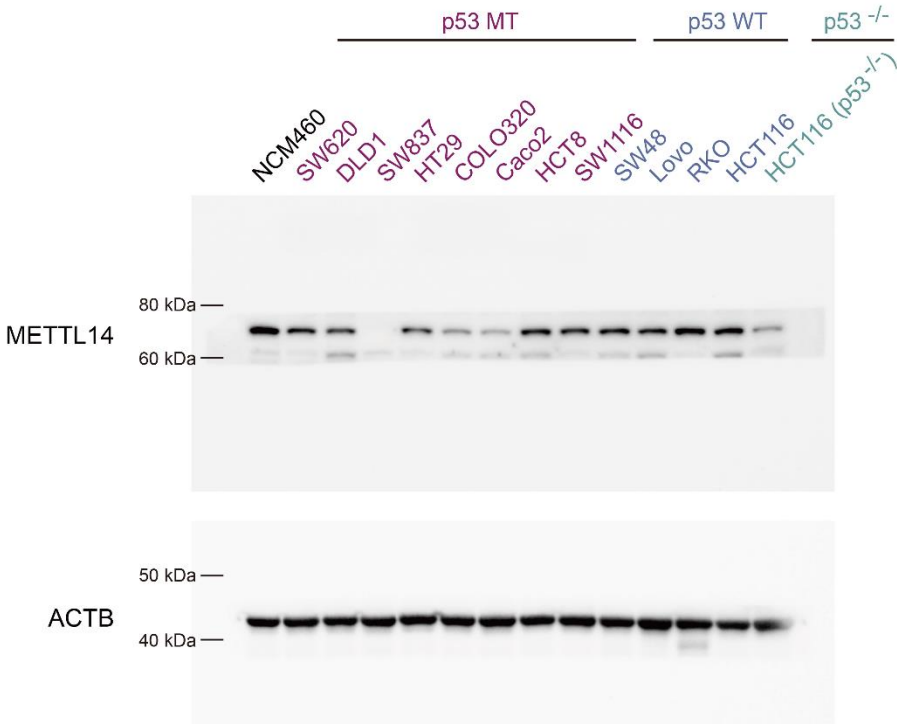

Supplement: Supplementary file 6 — Source Data for Expanded View and Appendix [file EMBR-24-e56325-s007.zip › Appendix_and_EV_Figures/source_data_Appendix fig_2.pdf]

## Source data for Fig EV3F

Fig EV3F

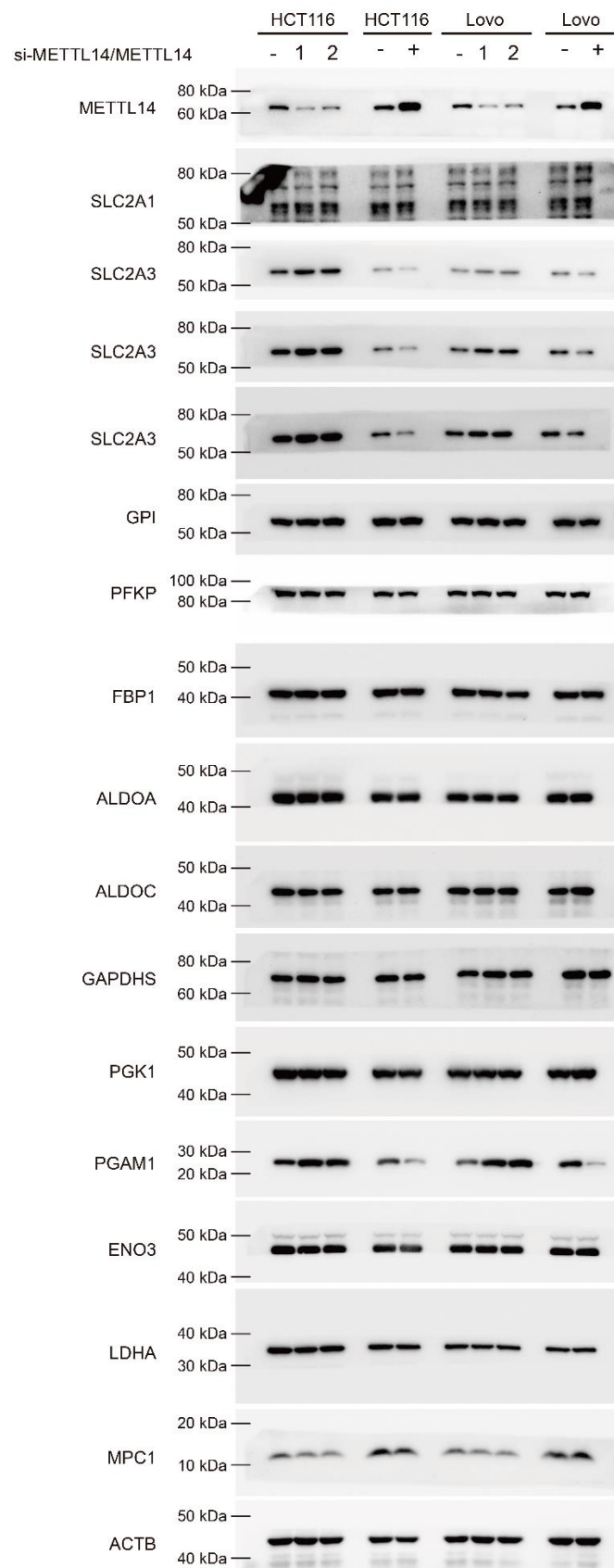

Supplement: Supplementary file 6 — Source Data for Expanded View and Appendix [file EMBR-24-e56325-s007.zip › Appendix_and_EV_Figures/source_data_Fig EV3F.pdf]

Source data for Fig 1

Fig 1D

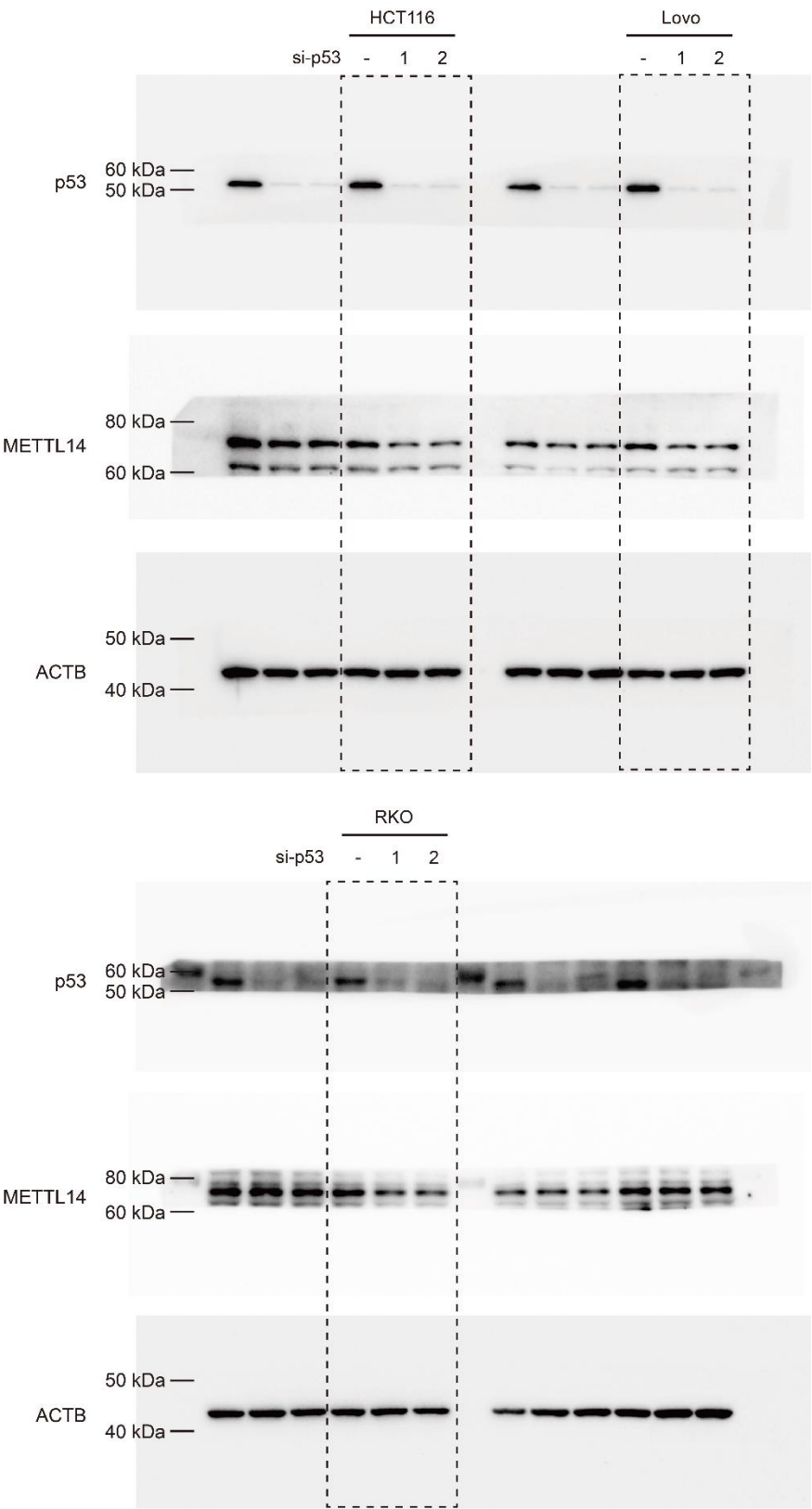

Fig 1E

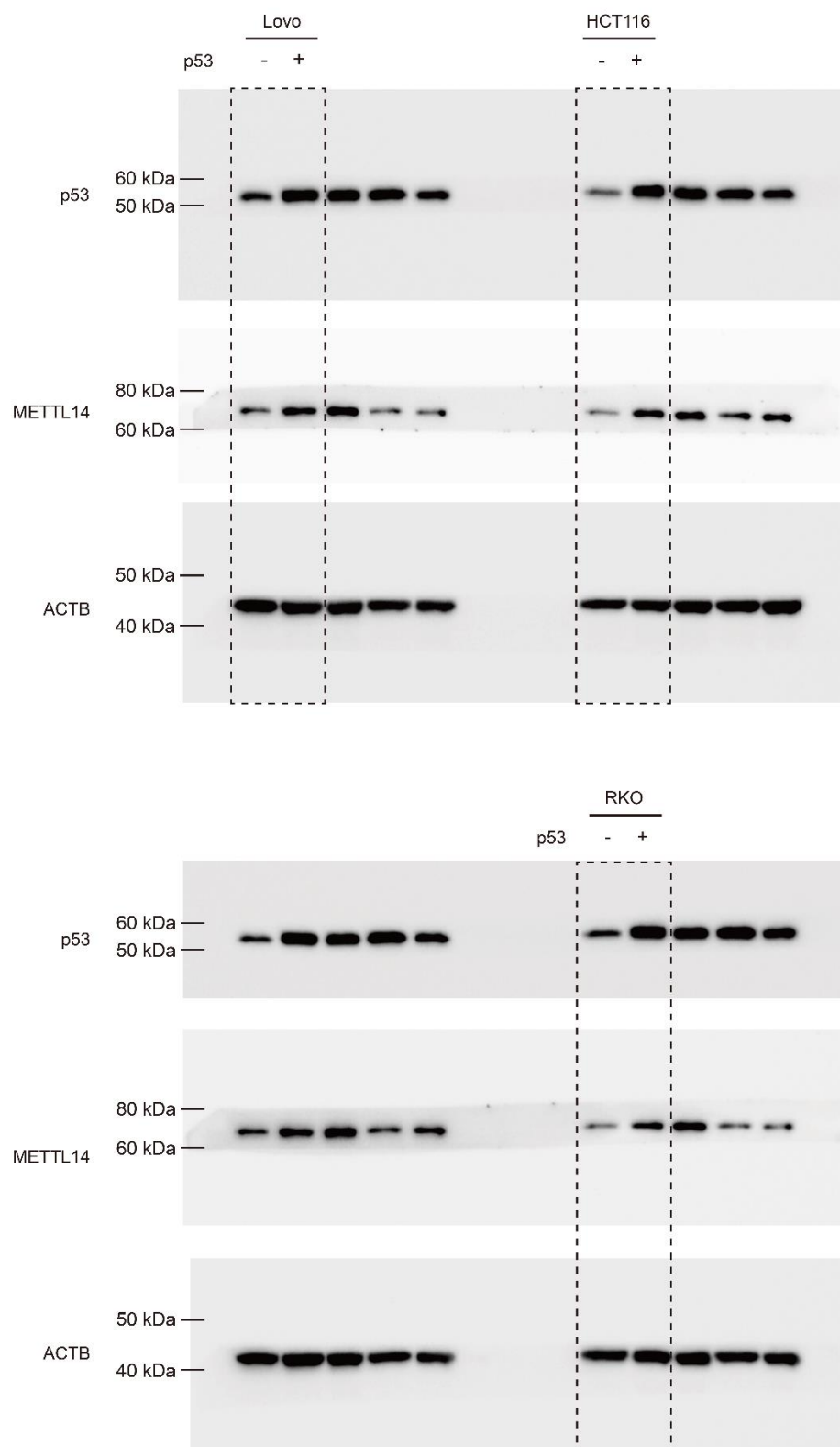

Fig 1l

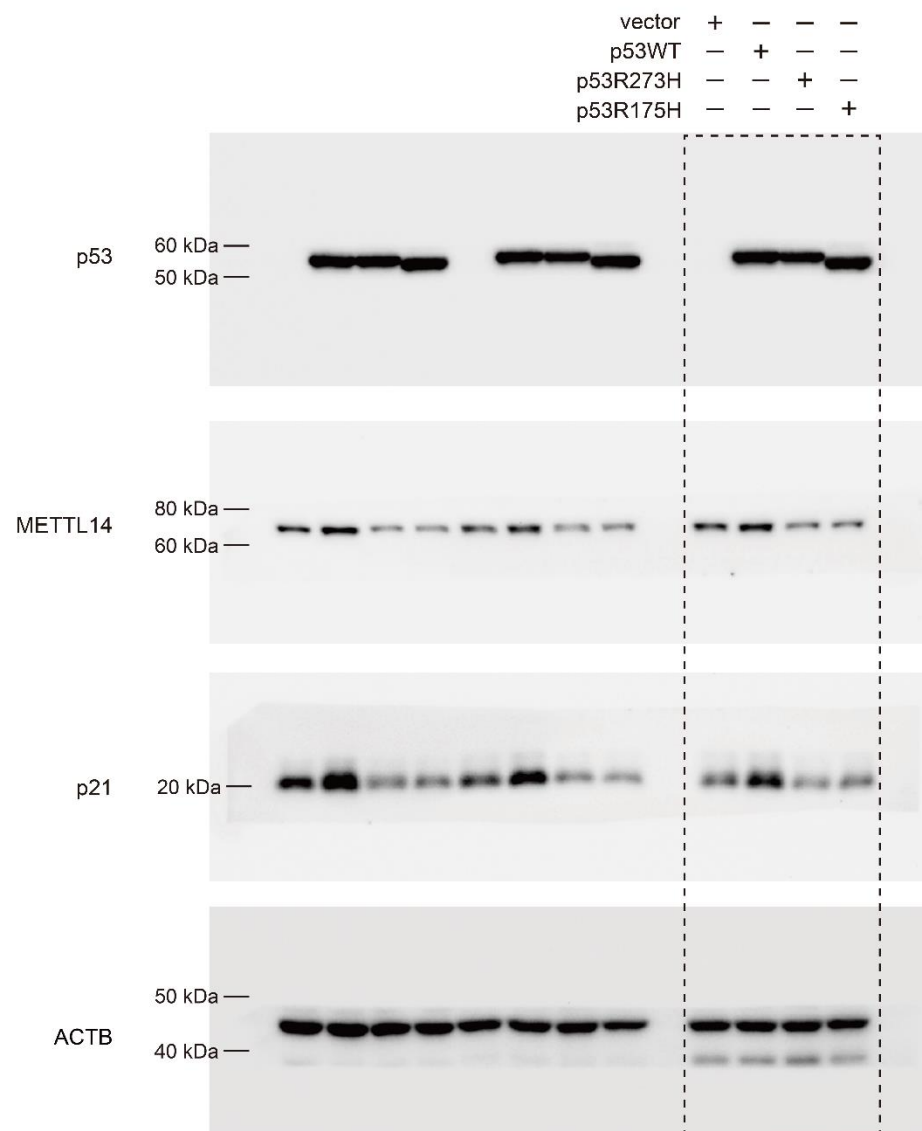

Fig 1K

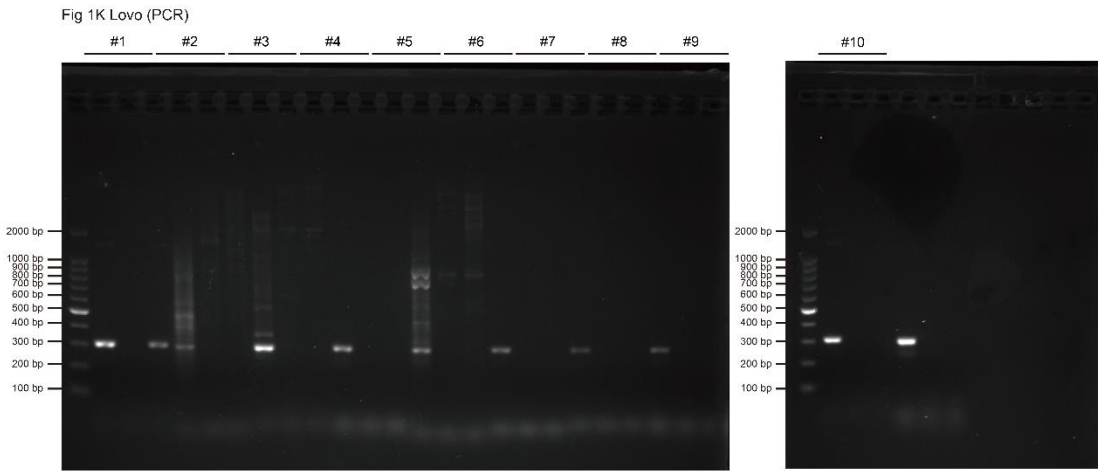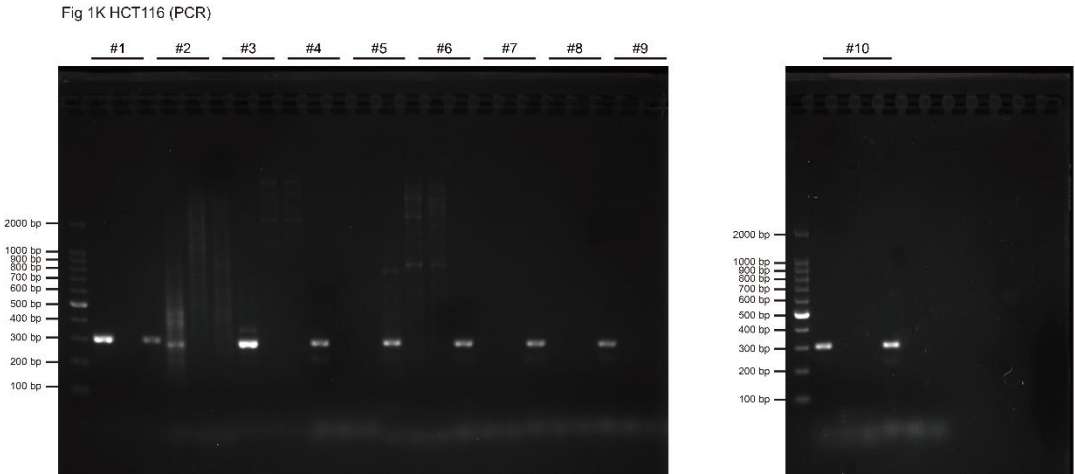

Supplement: Supplementary file 8 — Source Data for Figure 1 [file EMBR-24-e56325-s003.pdf]

Source data for Fig 3

Fig 3G

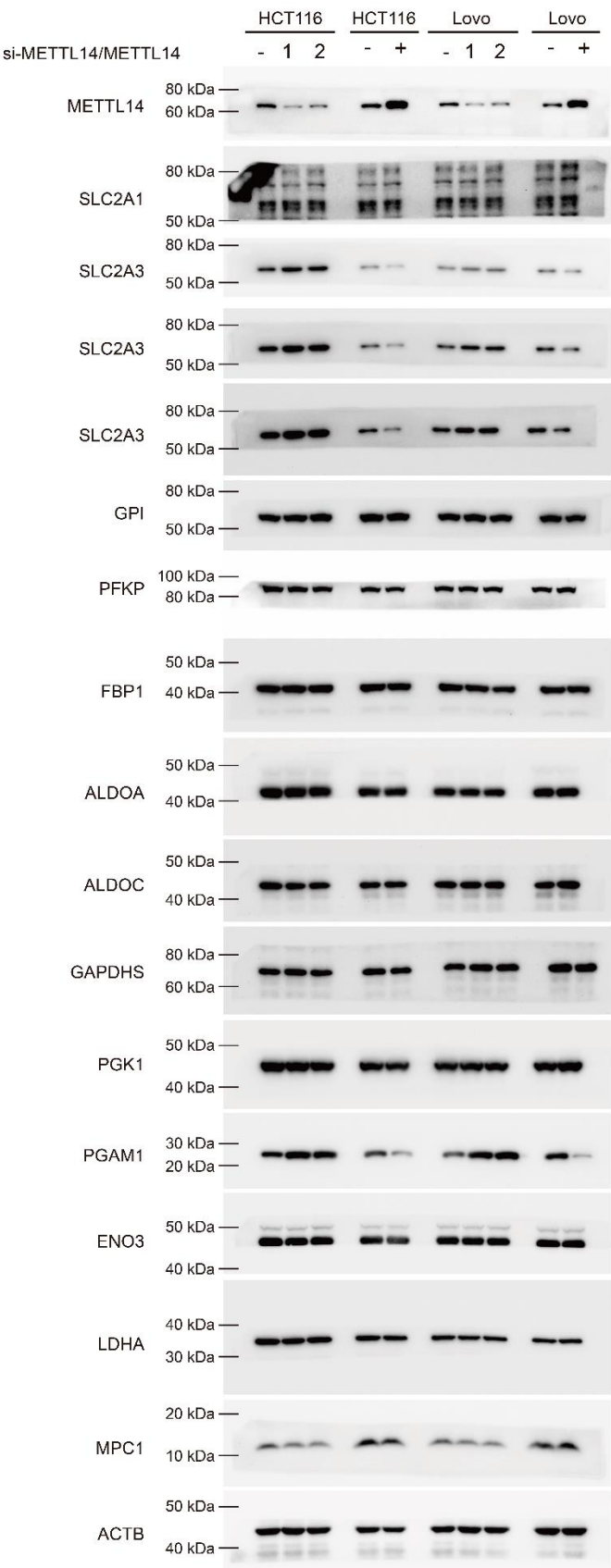

Fig 3H

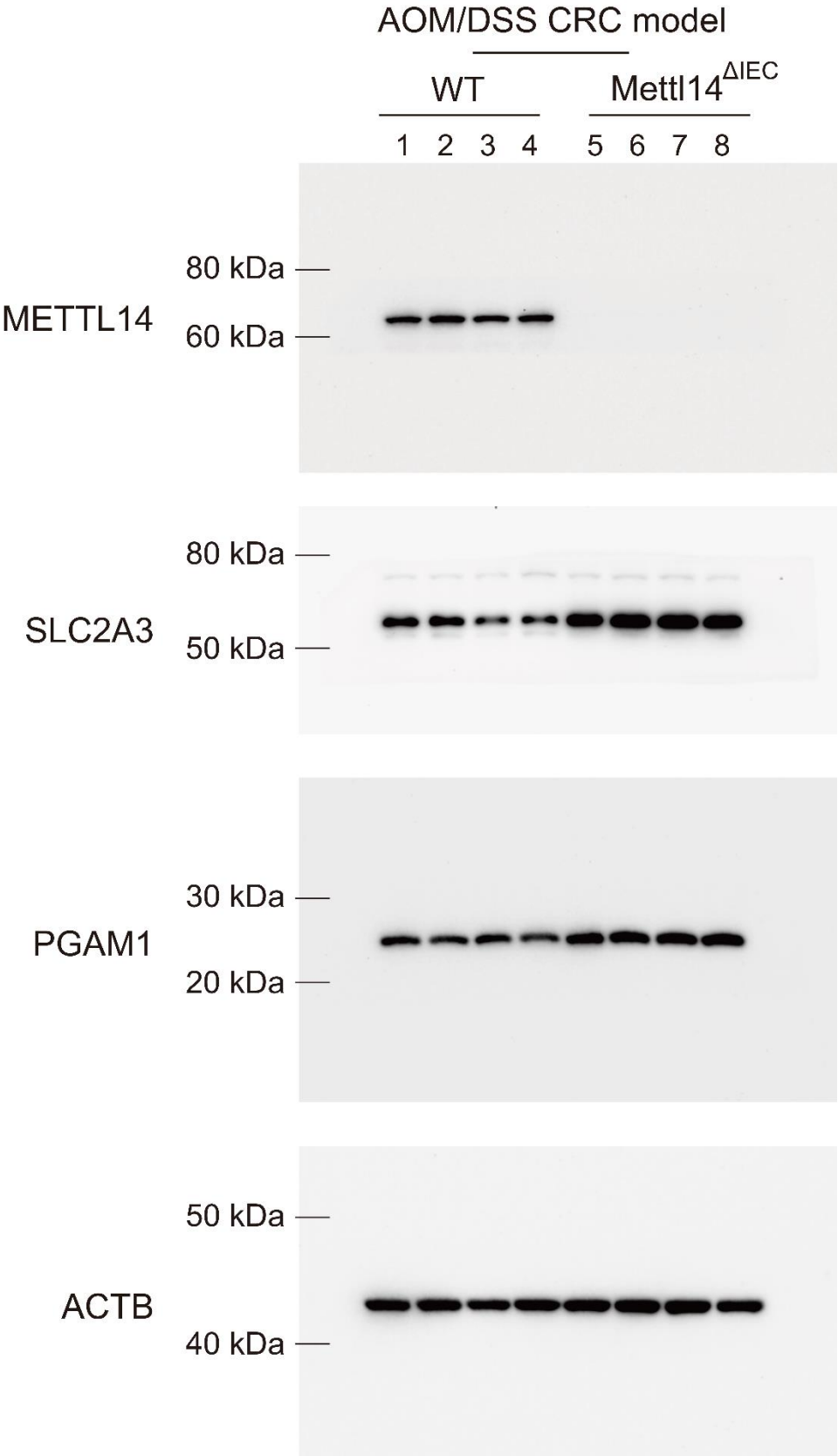

Supplement: Supplementary file 9 — Source Data for Figure 3 [file EMBR-24-e56325-s009.pdf]

Source data for Fig 4

Fig 4A

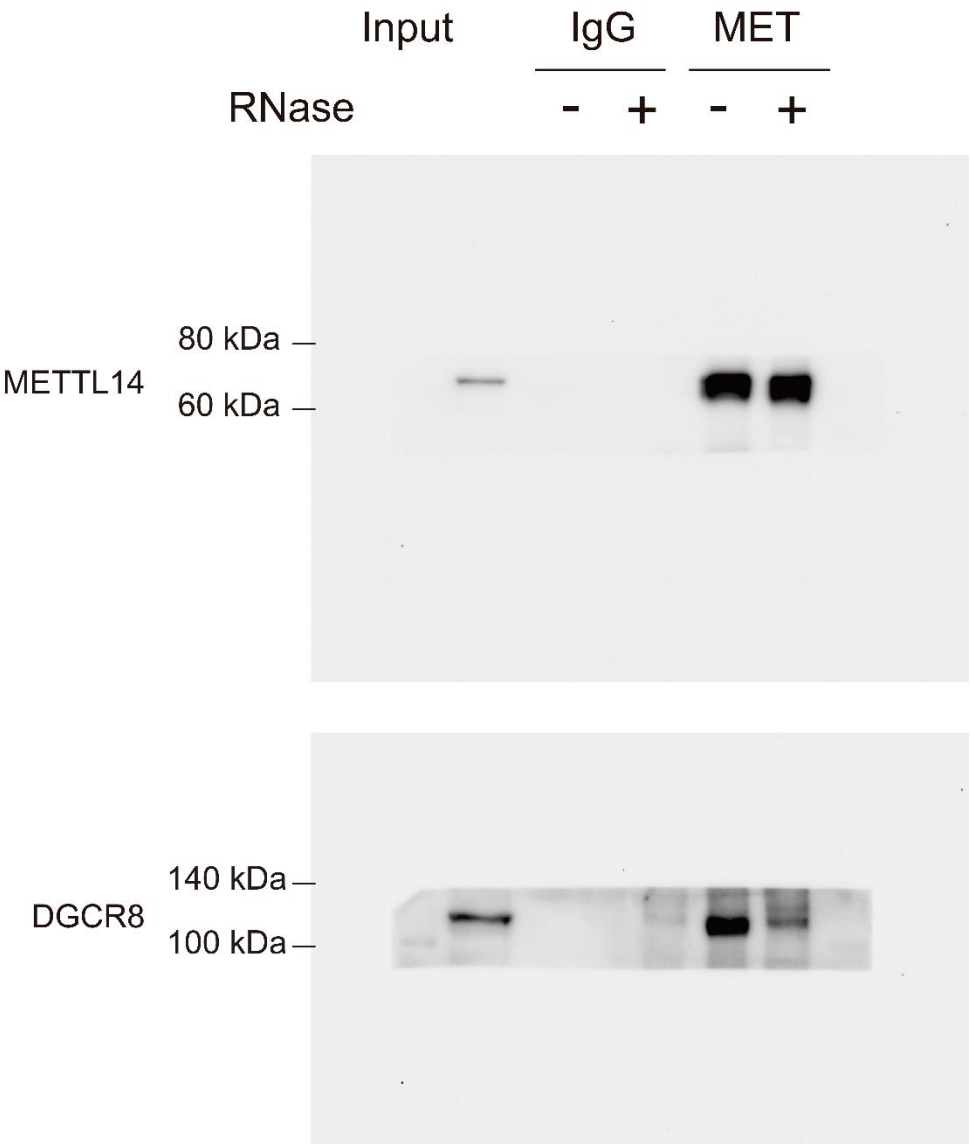

Fig 4E

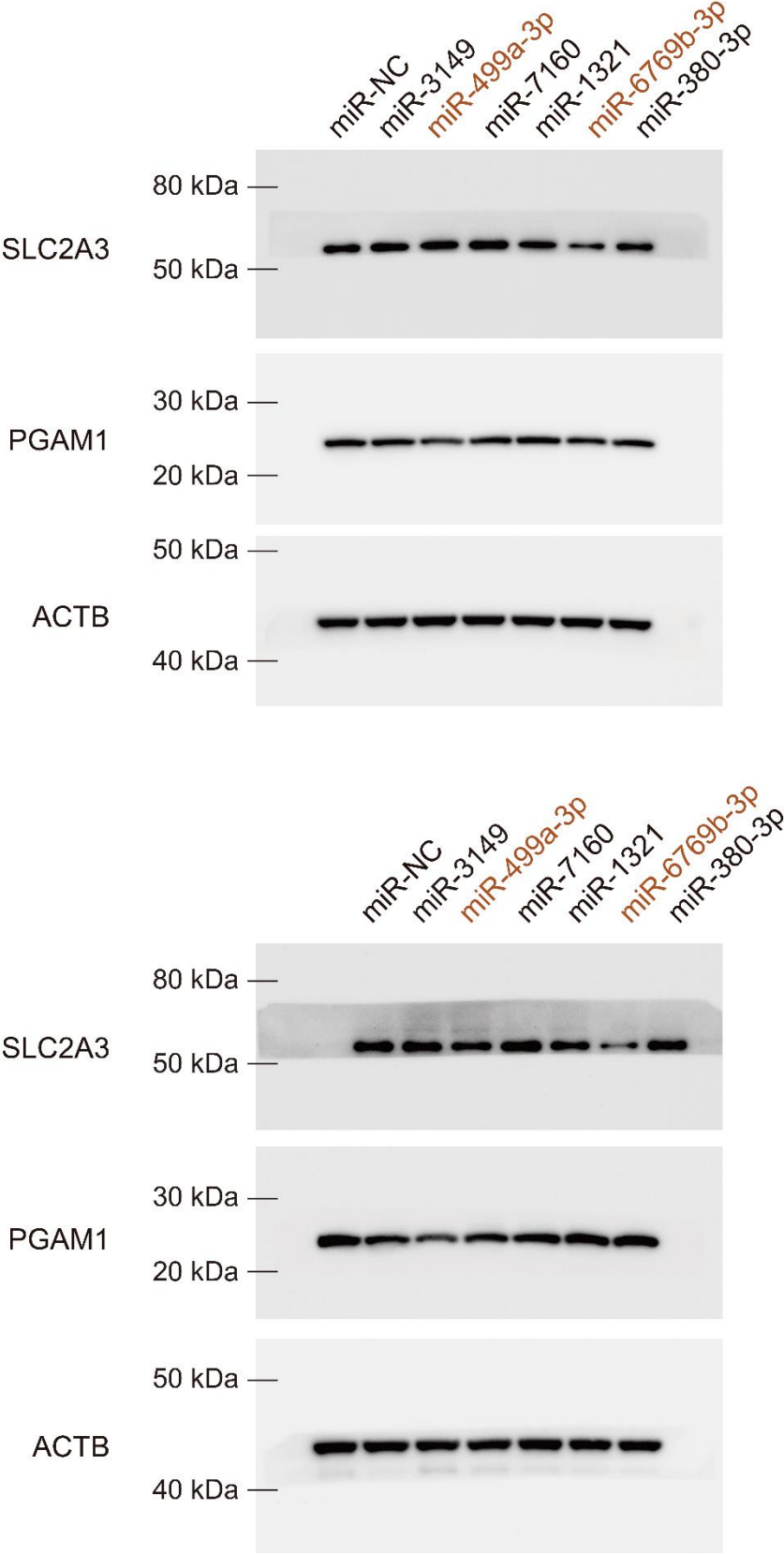

Supplement: Supplementary file 10 — Source Data for Figure 4 [file EMBR-24-e56325-s002.pdf]

Source data for Fig 5

Fig 5E

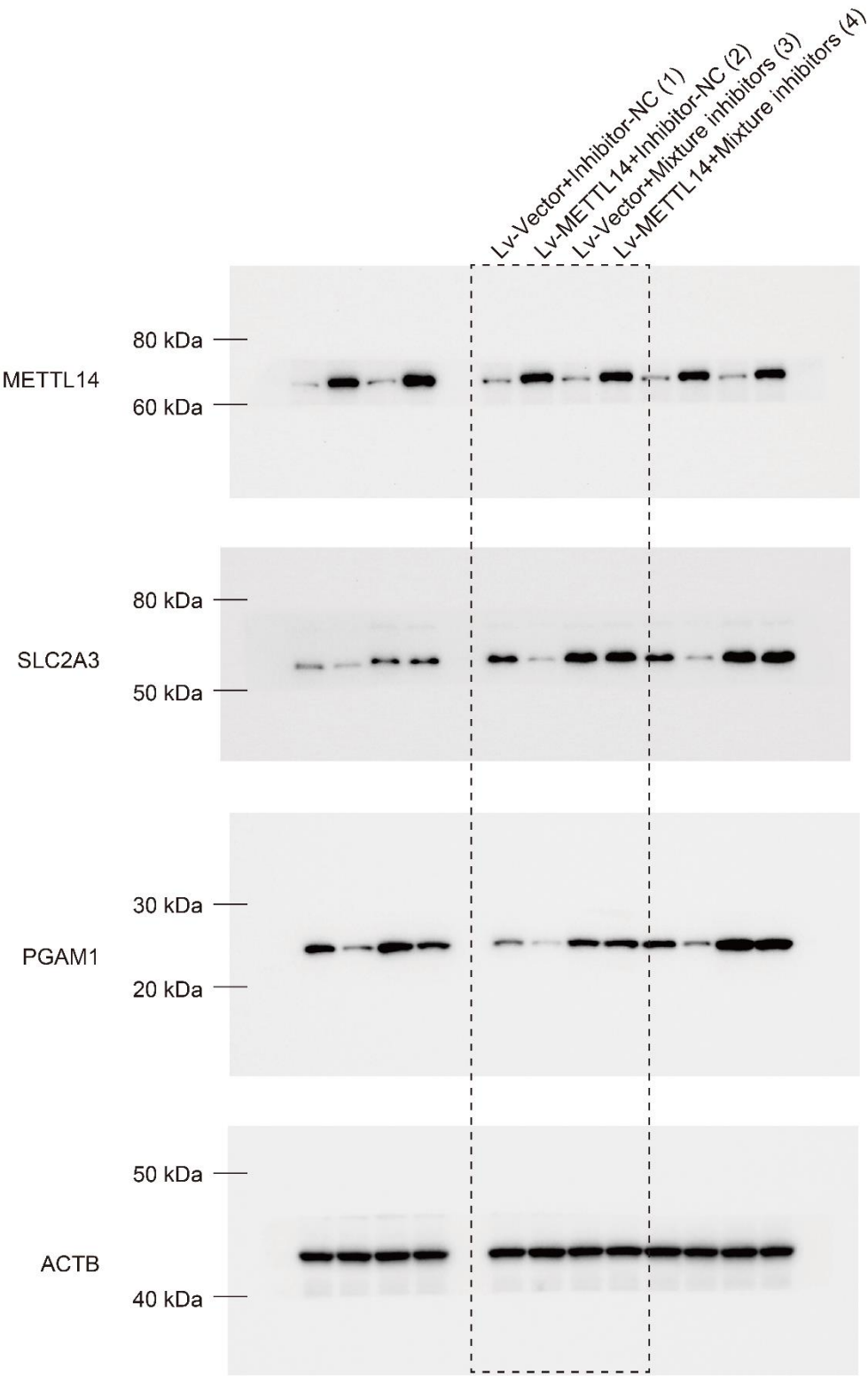

Fig 5F

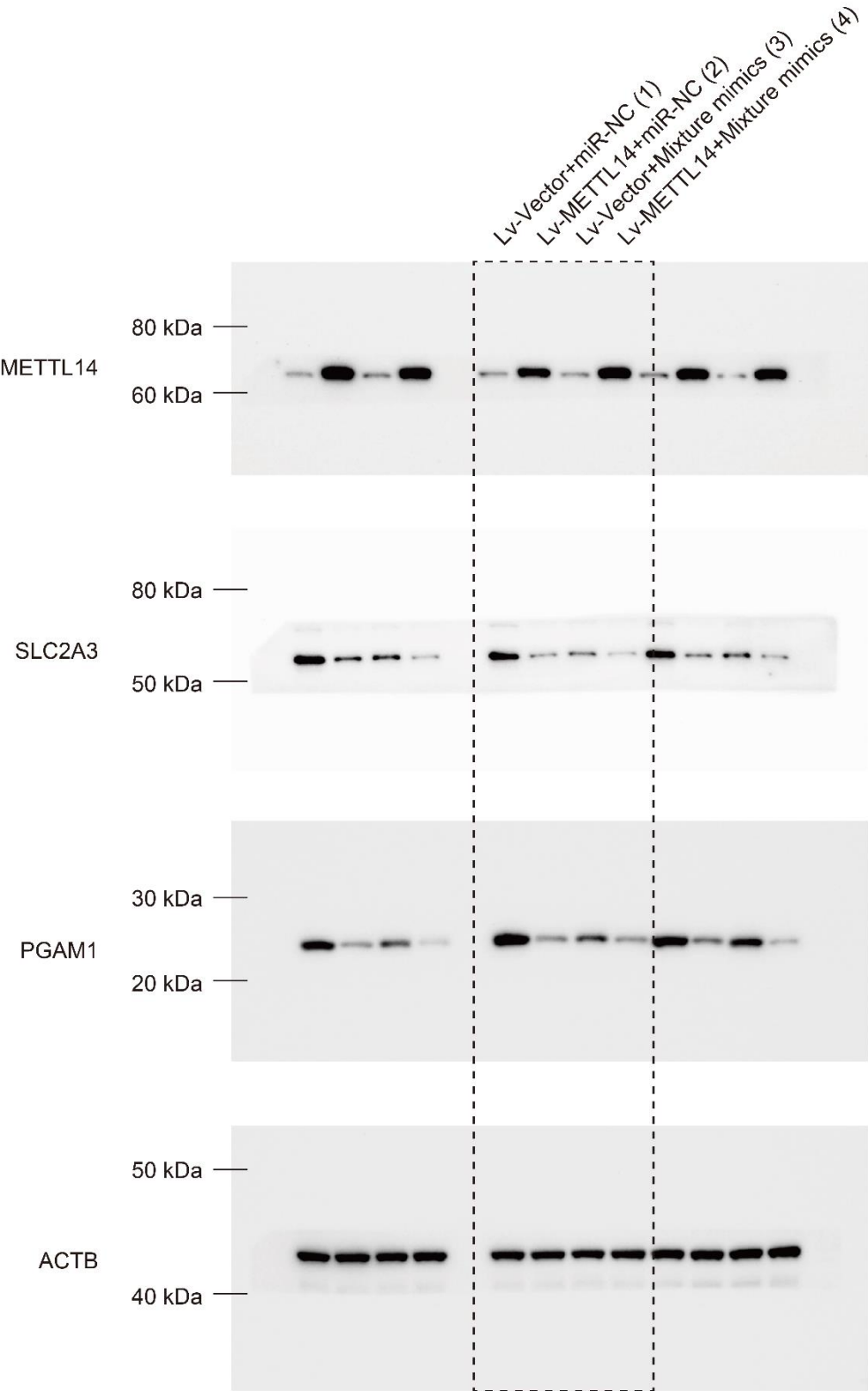

Fig 5K

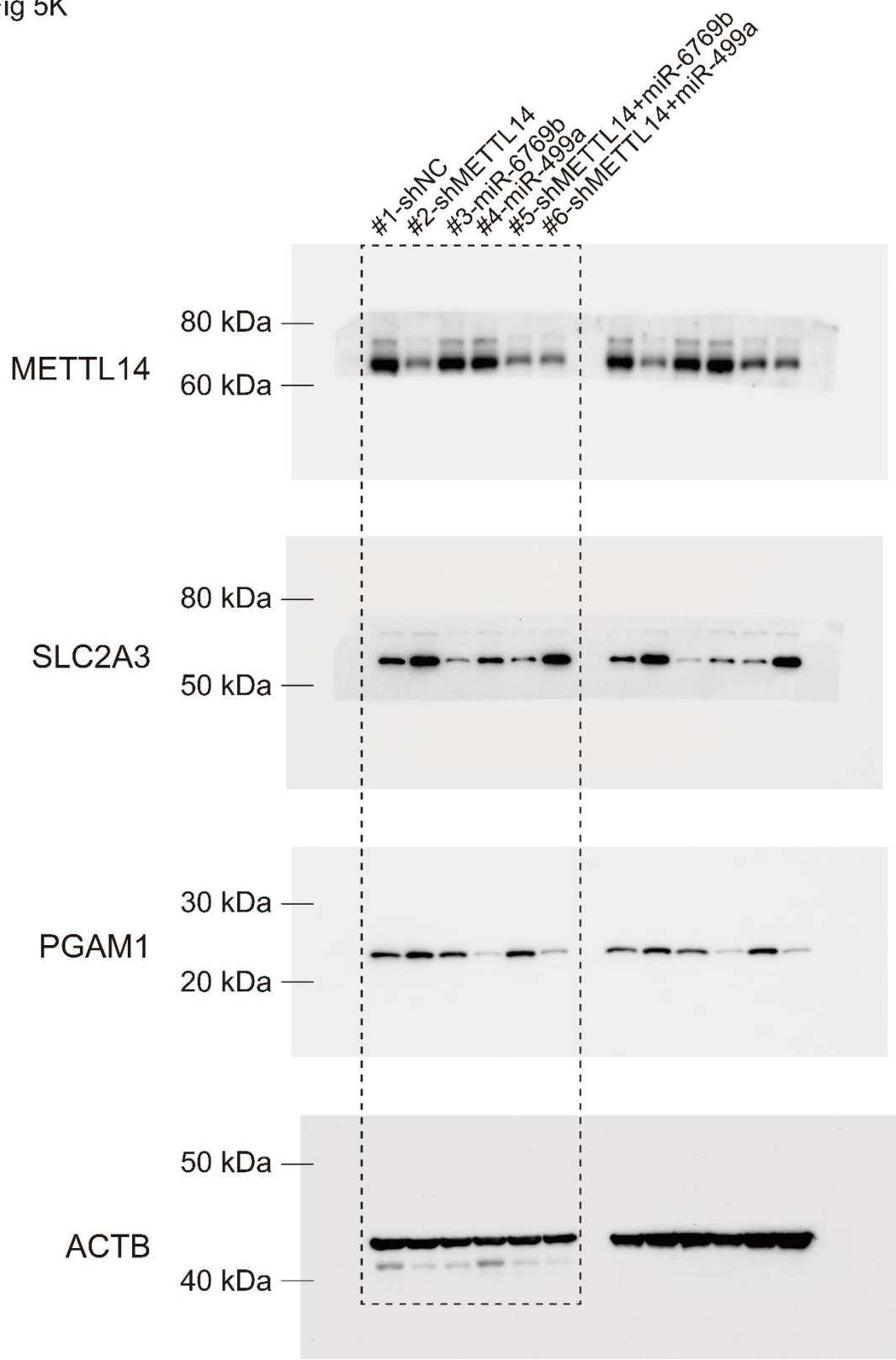

Fig 5N

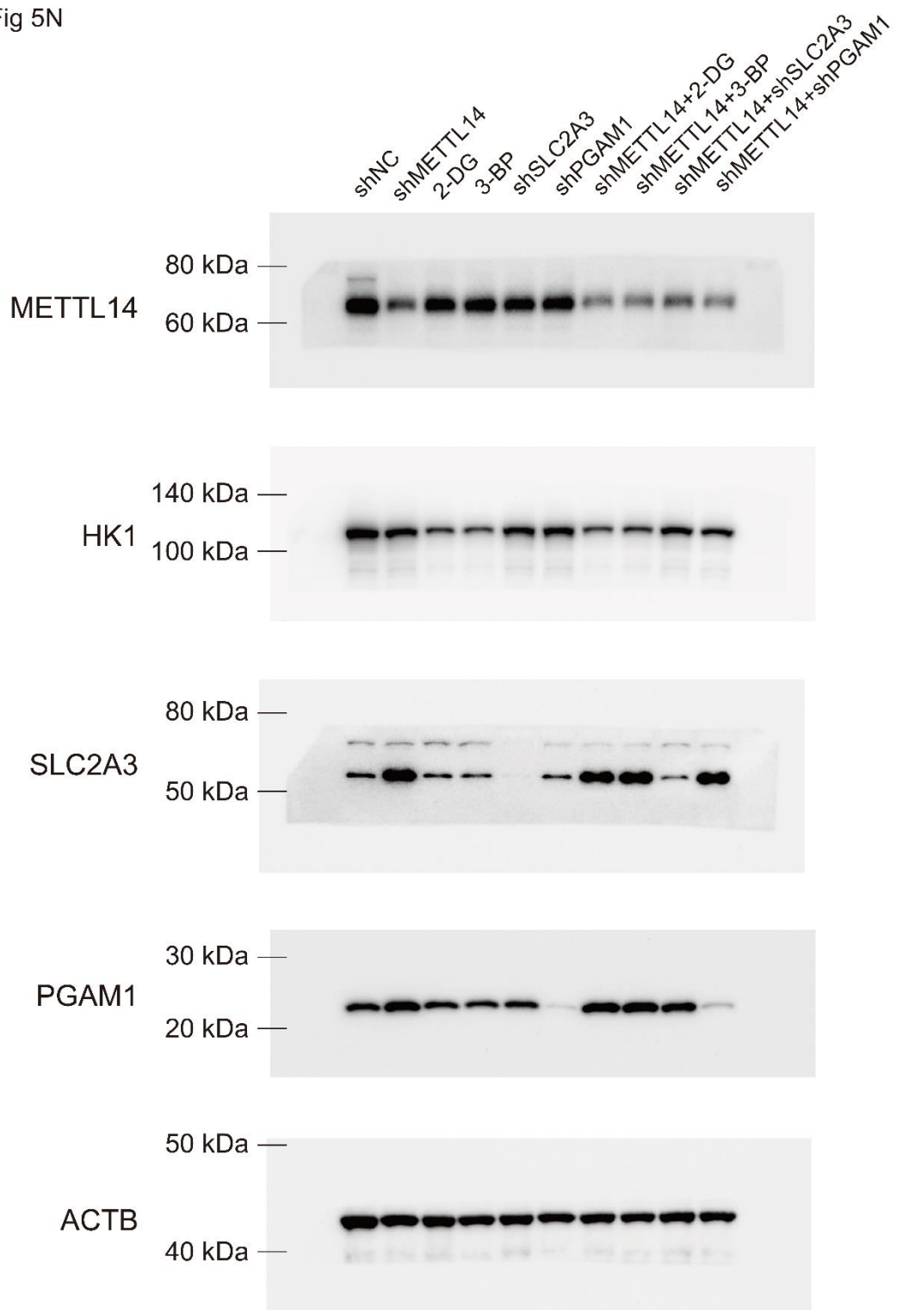

Supplement: Supplementary file 11 — Source Data for Figure 5 [file EMBR-24-e56325-s012.pdf]
