# Supplementary material for: METTL14 modulates glycolysis to inhibit colorectal tumorigenesis in p53‐wild‐type cells
Source: EMBO Rep. 2023 Feb 16;24(4):e56325. doi: 10.15252/embr.202256325 (PMC10074077; doi:10.15252/embr.202256325)
Supplement: Supplementary file 7 — PDF+ [file EMBR-24-e56325-s001.pdf]

Article

SOURCE DATA | TRANSPARENT PROCESS | OPEN ACCESS

EMBO reports

METTL14 modulates glycolysis to inhibit colorectal tumorigenesis in p53-wild-type cells

Yichao Hou[1,†], Xintian Zhang[1,†], Han Yao[1,†], Lidan Hou[1], Qingwei Zhang[2], Enwei Tao[2], Xiaoqiang Zhu[2], Shanshan Jiang[2], Yimeng Ren[2], Xialu Hong[2], Shiyuan Lu[2], Xiaoxu Leng[2], Yile Xie[2], Yaqi Gao[2], Yu Liang[1], Ting Zhong[1], Bohan Long[1], Jing-Yuan Fang[2,*] & Xiangjun Meng[1,**]

Abstract

The frequency of p53 mutations in colorectal cancer (CRC) is approximately 40–50%. A variety of therapies are being developed to target tumors expressing mutant p53. However, potential therapeutic targets for CRC expressing wild-type p53 are rare. In this study, we show that METTL14 is transcriptionally activated by wild-type p53 and suppresses tumor growth only in p53-wild-type (p53-WT) CRC cells. METTL14 deletion promotes both AOM/DSS and AOM-induced CRC growth in mouse models with the intestinal epithelial cell-specific knockout of METTL14. Additionally, METTL14 restrains aerobic glycolysis in p53-WT CRC, by repressing SLC2A3 and PGAM1 expression via selectively promoting m^{6}A-YTHDF2-dependent pri-miR-6769b/pri-miR-499a processing. Biosynthetic mature miR-6769b-3p and miR-499a-3p decrease SLC2A3 and PGAM1 levels, respectively, and suppress malignant phenotypes. Clinically, METTL14 only acts as a beneficial prognosis factor for the overall survival of p53-WT CRC patients. These results uncover a new mechanism for METTL14 inactivation in tumors and, most importantly, reveal that the activation of METTL14 is a critical mechanism for p53-dependent cancer growth inhibition, which could be targeted for therapy in p53-WT CRC.

Keywords aerobic glycolysis; colorectal cancer; m^{6}A; METTL14; wild-type p53
Subject Categories Cancer; Metabolism; RNA Biology
DOI 10.15252/embr.202256325 | Received 18 October 2022 | Revised 19 January 2023 | Accepted 27 January 2023 | Published online 16 February 2023
EMBO Reports (2023) 24: e56325

Introduction

Colorectal cancer (CRC) is the third leading cause of death among various cancers and is attributed to a series of mutational events including the adenomatous polyposis coli (APC), Kirsten rat sarcoma 2 viral oncogene homolog (KRAS), B-Raf proto-oncogene, serine/threonine kinase (BRAF), and tumor protein P53 (p53) (Walther *et al*, 2009; Siegel *et al*, 2021). Of note, approximately 40–50% of CRC patients have p53 mutation (Baker *et al*, 1989). Wild-type p53 is a tumor suppressor and functions as a critical transcription factor in cell-cycle checkpoints, apoptosis, and senescence (Levine & Oren, 2009; Vousden & Prives, 2009). In addition, studies have shown that energy metabolism manipulation is a key point of wild-type p53 in attenuating tumorigenesis and development (Vousden & Ryan, 2009; Levine & Puzio-Kuter, 2010). Wild-type p53 represses Warburg effect by directly or indirectly regulating genes involved in metabolism, including TIGAR, SCO2, and Parkin (Bensaad *et al*, 2006; Zhang *et al*, 2011; Wang *et al*, 2018). Loss of wild-type p53 not only dampens its tumor-suppressive function but also obtains new oncogenic effects, called gain of function (GOF), such as enhanced capacity for metastasis, proliferation, and chemo-resistance (Muller & Vousden, 2013), making the reactivation of wild-type p53 or regulation of its downstream effectors' attractive therapeutic targets.

An emerging hallmark of cancer is metabolic reprogramming (Hanahan & Weinberg, 2011; Pavlova & Thompson, 2016). Cancer cells display altered glucose metabolism characterized by a preference for aerobic glycolysis even when supplemented with abundant oxygen. This aberrant metabolic feature has been known as the Warburg effect, which promotes tumor progression with a higher rate of glucose uptake and elevated ATP and lactate production, as well as supervenient macromolecules and other elements required for the generation of new cells in the context of rapid growth (Koppenol *et al*, 2011; Pavlova & Thompson, 2016). Emerging evidence has suggested that the Warburg effect contributes greatly to tumorigenesis and could be targeted for tumor therapy. For example, endogenous retroelement activation and inhibition of EGFR signaling pathways suppress tumorigenesis by reversing the Warburg effect and reactivating oxidative phosphorylation (De Rosa *et al*, 2015; Fresquet *et al*, 2021), highlighting the perspective therapeutic targets of glycolysis. Lactate, a by-product of glycolysis, may serve as a promising therapeutic target in cancers (Apicella *et al*, 2018; Zhang *et al*, 2019). RNA modifications create a code that functions

1 Shanghai Key Laboratory of Gut Microecology and Associated Major Diseases Research, Digestive Disease Research and Clinical Translation Center, Department of Gastroenterology, Shanghai Ninth People's Hospital, School of Medicine, Shanghai Jiao Tong University, Shanghai, China
2 State Key Laboratory for Oncogenes and Related Genes, Key Laboratory of Gastroenterology and Hepatology, Ministry of Health, Division of Gastroenterology and Hepatology, Shanghai Institute of Digestive Disease, Renji Hospital, School of Medicine, Shanghai Jiao Tong University, Shanghai, China
*Corresponding author. Tel: +86 13918386561; E-mail: jingyuanfang@sjtu.edu.cn
**Corresponding author. Tel: +86 13816170604; Fax: +86 21 5331 5166; E-mail: xiangjunmeng@sjtu.edu.cn; meng_xiangjun@yahoo.com
[†]These authors contributed equally to this work as first authors.

© 2023 The Authors. Published under the terms of the CC BY 4.0 license

as a key sensor of metabolism and is responsible for transducing metabolic changes into stable gene expression patterns (An & Duan, 2022). N^6-methyladenosine (m^{6}A) modification, one of the RNA modifications, is the most abundant RNA modification in eukaryotic mRNAs. Accumulating evidence indicates that m^{6}A regulates glycolysis to affect the occurrence and development of multiple human tumors, such as cervical cancer, liver cancer, and CRC, directly or indirectly through modification of glucose transport protein or glycolytic enzyme (Li *et al*, 2020; Shen *et al*, 2020; Wang *et al*, 2020). Thus, understanding the molecular mechanisms of glycolysis and m^{6}A in CRC is essential for developing future diagnostic and therapeutic strategies.

Deposit of m^{6}A requires a writer complex mainly composed of core methyltransferase-like 3 (METTL3) and METTL14 (Wang *et al*, 2016). Eraser enzyme Fat mass and obesity-associated protein (FTO) and AlkB homolog 5 (ALKBH5) serve as demethylases to reverse the m^{6}A modification (Shi *et al*, 2019). m^{6}A-modified transcripts are recognized by reader proteins such as YT521-B homology (YTH) domain family proteins (YTHDF1-3, YTHDC1, and YTHDC2), among which YTHDF2 was firstly validated, which regulate RNA splicing, stability, and translation (Wang *et al*, 2014; Shi *et al*, 2017; Zhou *et al*, 2019). Besides, available evidence indicates that m^{6}A methylation marks primary microRNA (pri-miRNA) transcripts for processing and then affects cellular process (Alarcón *et al*, 2015b; Cao *et al*, 2021); thus perturbation of miRNA biosynthesis by regulating m^{6}A modification on pri-miRNA could also interfere with cancer progression. Notably, dysregulation of m^{6}A methylated mRNA can regulate cancer development. FTO has been suggested to play a tumorigenic role in acute myeloid leukemia (Li *et al*, 2017; Huang *et al*, 2019b), while inhibition of FTO exerts an extensive anti-leukemic activity *in vitro* and *in vivo* (Su *et al*, 2018). ALKBH5 can maintain the tumorigenicity of glioblastoma stem-like cells via sustaining FOXM1 expression (Zhang *et al*, 2017). Our recent study showed that m^{6}A marks enhance the recognition of pri-miR-30d by YTHDC1, and subsequently increased mature miR-30d inactivates the glycolysis signaling to repress pancreatic tumorigenesis (Hou *et al*, 2021). Although the relationship between m^{6}A modification and tumorigenesis has been proposed in recent years, the precise mechanisms associated with glycolysis in diverse status p53 CRC have not been fully elucidated.

In this study, we employed AOM and AOM/DSS-induced intestinal epithelial cell (IEC)–specific METTL14 knockout (Mettl14$^{\Delta IEC}$) CRC mouse models to verify the crucial suppressive role of METTL14 in both adenoma and colitis-associated malignant transformation. Besides, METTL14 expression was transcriptionally regulated by wild-type p53 and METTL14-mediated m^{6}A modification selectively promoted miRNAs maturation in a YTHDF2-dependent manner, thereby inhibiting Warburg effect in p53-WT CRC by targeting SLC2A3 and PGAM1, respectively. Taken together, METTL14 attenuates p53-WT CRC progression through repressing Warburg effect in an m^{6}A-dependent manner.

Results

Wild-type p53 activates METTL14 expression in CRC cell lines

As one of the core m^{6}A methyltransferases, METTL14 engages in dynamic m^{6}A modification. Analysis of The Cancer Genome Atlas (TCGA) datasets found METTL14 is down-regulated in many solid tumors (Fig EV1A). We then compared the mRNA and protein levels of METTL14 in CRC tissues and paired adjacently normal colorectal tissues in our Cohort 1 and Cohort 2, and we found that the core methyltransferase METTL14 was down-regulated in CRC (Fig EV1B and C). To explore the mechanism of the decreased METTL14 expression in CRC, we found that p53 and ELK1 might act as predictive transcription factors (TFs) for targeting METTL14 using PROMO, ChIPBase, and TRAP database (Fig 1A). Moreover, analysis of multiple datasets using a multiple experiment matrix (MEM) revealed that p53, but not ELK1, was the co-expressed gene with METTL14 in CRC (Fig 1B). Besides, the knockdown of ELK1 slightly decreased the mRNA level of METTL14 whereas failed to affect the protein level of METTL14 (Fig EV1D and E). Notably, p53 overexpression or knockdown up-regulated or down-regulated METTL14 mRNA and protein expression, respectively (Fig 1C–E). Interestingly, we examined the subcellular distribution of METTL14 by immunofluorescence (IF) and found obvious co-expression of METTL14 with p53 in nuclei of p53-WT cells, while the co-expression was not obvious in p53-MT cells (Fig 1F). In line with the relationship between METTL14 expression and wild-type p53 identified *in vitro*, it was demonstrated that the METTL14 and p53 mRNA levels were correlated in p53-WT cancer cells, but not in p53-MT cancer cells (Fig 1G), whereas no significant correlation was present in KRAS-WT or KRAS-MT cancer cells in the Cancer Cell Line Encyclopedia (CCLE) database (Appendix Fig S1A).

Moreover, GEO datasets showed a higher METTL14 level in p53-WT CRC than in p53-MT CRC tissues, whereas no difference was detected between KRAS-WT and KRAS-MT tissues (Appendix Fig S1B). Consistently, datasets from TCGA also demonstrated that diverse tumors harboring wild-type p53 displayed higher METTL14 mRNA levels than tumors harboring p53 mutations (Fig EV1F). KRAS-WT and KRAS-MT tumor tissues displayed no obvious differences in METTL14 mRNA levels (Fig EV1F). Of note, METTL14 was not increased in HCT116 (p53$^{-/-}$) cells transduced with p53 mutants (p53R175H and p53R273H), further highlighting the importance of wild-type p53 activity in transcriptional regulation of METTL14 (Fig 1H and I). Intriguingly, depletion of mutant p53 had no effect on METTL14 protein expression in p53-MT CRC cells (Fig EV1G), indicating that the levels of p53 in CRC cells carrying p53 mutations were not related to METTL14 levels. Correspondingly, METTL14 protein level is relatively higher in p53-WT than that in p53-MT and p53-null CRC cells (Appendix Fig S2). Strikingly, p53-WT CRC had a much higher protein level of METTL14 compared with p53-MT samples (Fig 1J), while no differences were found between KRAS-WT and KRAS-MT CRC samples in Cohort 3 (Appendix Fig S3A). In addition, we found that METTL3, another crucial m^{6}A methyltransferase, displays no obvious differential expression in p53-WT and p53-MT CRC tissues (Fig EV1H and Appendix Fig S3B). These findings suggest that METTL14 might play a more tissue-restricted role in p53-mediated tumor suppression. Since METTL14 expression is dependent on p53 transcriptional activity, we further explored the p53-binding site within METTL14 promoter region. We analyzed the sequence of METTL14 promoter for the potential p53-binding region (p53-BR) using JASPAR profile database (Mathelier *et al*, 2016). Three putative p53-binding sites were identified at 3 kb upstream of METTL14 transcription start site (Fig EV1I). The subsequent ChIP assay verified the interaction of

 © 2023 The Authors

p53 with DNA fragment #1 comprising the p53-BR1 site (Fig 1K). Consistently, quantitative analysis of ChIP–qRT–PCR indicated that p53 is directly bound to DNA fragment #1 but not to other fragments of the METTL14 promoter in HCT116 and Lovo cells (Fig 1L). To further determine whether the p53-BR1 site confers p53-dependent activity, luciferase reporter assay was performed. Results showed

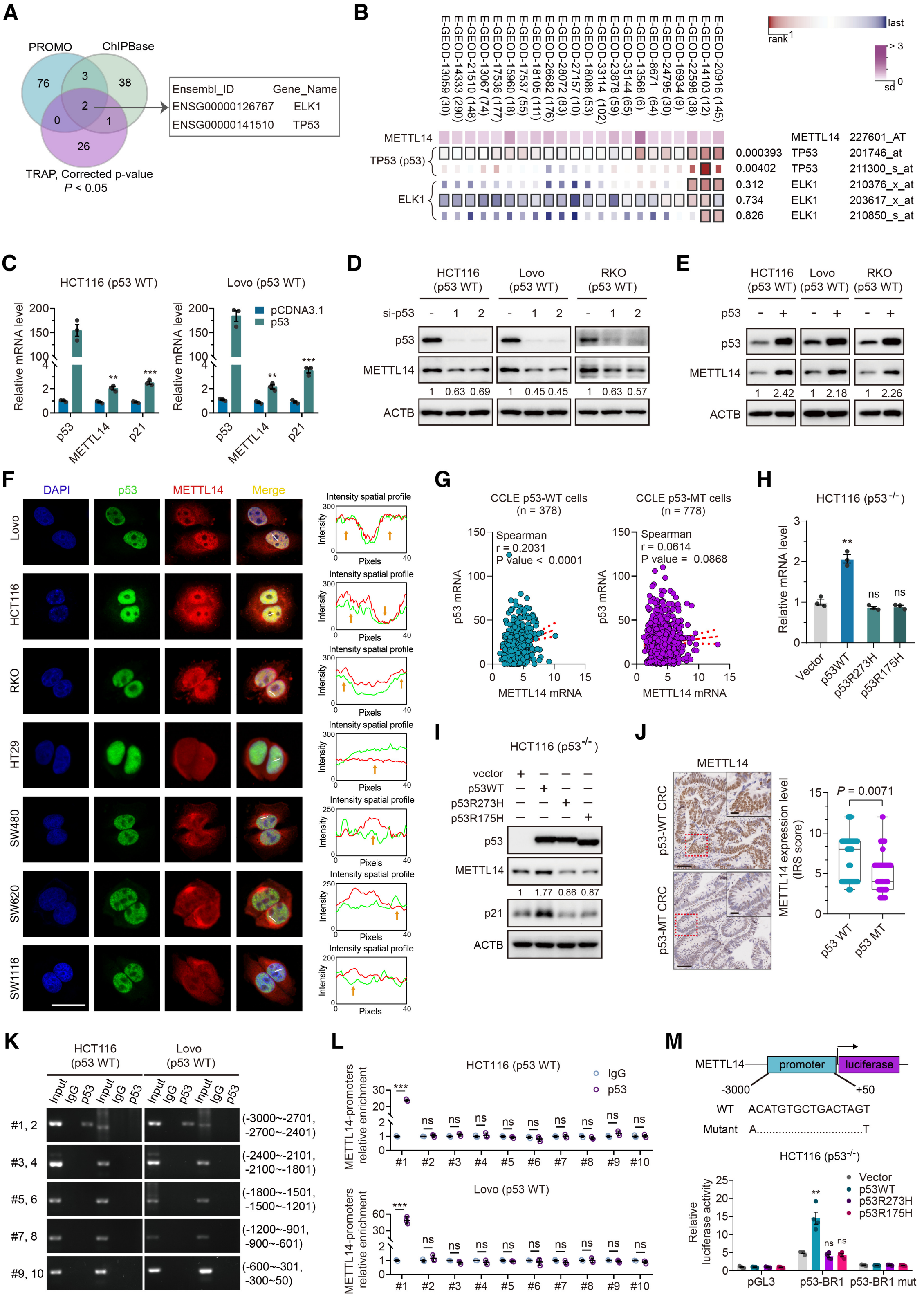

Figure 1.

© 2023 The Authors

Figure 1. Wild-type p53 activates METTL14 expression in CRC cell lines.

A Schematic illustration of screening for transcription factors using ChIPBase (http://rna.sysu.edu.cn/chipbase3/index.php), PROMO alggen (http://alggen.lsi.upc.es/cgi-bin/promo_v3/promo/promoinit.cgi?dirDB=TF_8.3/) and TRAP (http://trap.molgen.mpg.de/).
B MEM (https://biit.cs.ut.ee/mem/) identified correlation between ELK1, p53 and METTL14.
C HCT116 and Lovo cells were transfected with empty vector and p53 plasmid. Forty-eight hours after transfection, total RNA was then analyzed by qRT–PCR analysis. Data are presented as mean ± SD (biological replicates, $n = 3$; $^{**}P < 0.01$, $^{***}P < 0.001$).
D HCT116, Lovo, and RKO cells were transfected with control or p53 siRNAs. Forty-eight hours after transfection, total protein was then analyzed by western blot analysis.
E HCT116, Lovo, and RKO cells were transfected with empty vector and p53 plasmid. Forty-eight hours after transfection, total protein was then analyzed by western blot analysis.
F Representative immunofluorescence staining of the p53 (green) and METTL14 (red) proteins in p53-WT HCT116, Lovo, and RKO cells and p53-MT HT29 (p53R273H), SW480 (p53R273H/P309S), SW620 (p53R273H), and SW1116 (p53A159D) cells. Nuclei were stained with DAPI (blue). Scale bars = 10 μm. The relative mean fluorescence density was analyzed by ImageJ.
G Correlation of METTL14 with p53 mRNA levels in p53-WT and p53-MT CRC cell lines from CCLE database (https://sites.broadinstitute.org/ccle). *r* is the Spearman's rank correlation coefficient.
H qRT–PCR analysis of METTL14 levels after transfection with empty vector, wild-type, or mutant p53 plasmids in HCT116 ($p53^{-/-}$) cells (biological replicates, $n = 3$; $^{**}P < 0.01$, ns = no significance).
I Western blot analysis of METTL14 levels after transfection with empty vector, wild-type, or mutant p53 plasmids in HCT116 ($p53^{-/-}$) cells.
J Representative IHC images, and statistical analysis of immunoreactive score (IRS) of METTL14 expression in p53-WT ($n = 63$) and p53-MT ($n = 41$) CRC samples. The horizontal lines represent the median; the bottom and top of the boxes represent the 25 and 75% percentiles, respectively; and the vertical bars represent the range of the data. The insets show enlarged images of indicated p53-WT and p53-MT CRC tissues, respectively. Scale bars = 20 μm and 2 μm (inset).
K ChIP assay verified the potential p53-binding site in the METTL14 promoter region in HCT116 and Lovo cell lines. Input fractions and IgG were used as controls.
L ChIP–qRT–PCR assay verified the potential p53-binding site in the METTL14 promoter region in HCT116 and Lovo cell lines. IgG was used as a control. Data are presented as mean ± SD (biological replicates, $n = 3$; $^{***}P < 0.001$, ns = no significance).
M Luciferase activities of luciferase reporter plasmid containing wild-type or mutant METTL14 promoter in control, wild-type p53 or mutant p53-overexpressing HCT116 ($p53^{-/-}$) cells. pGL3 and Vector were used as controls. Data are presented as mean ± SD (biological replicates, $n = 4$; $^{**}P < 0.01$, ns = no significance).

Data information: For (C, H, L, M), statistical significance was determined by the two-tailed Student's *t*-test. For (J), statistical significance was determined by the non-parametric Mann–Whitney test. β-actin (ACTB) was used as a loading control, and $p21^{WAF1/Cip1}$ was used as a positive control. The signal intensities were quantified by densitometry using ImageJ software and normalized to the intensity of the internal positive controls.
Source data are available online for this figure.

that co-transfection of wild-type p53 selectively enhanced the transcriptional activity of reporters with intact p53-BR1, whereas mutant p53 did not activate reporter activity in p53-null CRC cells (Fig 1M). Collectively, METTL14 is positively regulated by wild-type p53, which physically interacts with p53-BR1 in the promoter region of METTL14 to induce its expression.

METTL14 inhibits p53-WT CRC progression

The above results prompted us to investigate the role of METTL14 in diverse CRC cells *in vitro* and *in vivo*. We found that the stable knockdown of METTL14 enhanced cell viability and colony formation ability of p53-WT cells compared with the respective controls, whereas p53-MT cells treated in the same way did not show the same phenotype (Figs EV2A–C and 2A). We then examined the *in vivo* roles of METTL14 in p53-WT and p53-MT CRC cells and found that the knockdown of METTL14 increased the tumor volume and weight in p53-WT tumors but not the p53-MT ones (Fig 2B and C). Moreover, IHC analysis of Ki-67, a tumor proliferation marker was performed to evaluate the proliferation of the tumor xenografts. In concordance with the *in vitro* and *in vivo* findings, the Ki-67-positive nuclei staining was increased in xenografts formed by METTL14 silencing p53-WT cells compared with that in xenografts formed by control cells (Fig EV2D), but the difference was not significant statistically between control and METTL14 silencing p53-MT cells (Fig EV2D). Collectively, these findings demonstrate that the wild-type p53 target gene METTL14 might have potent wild-type p53-dependent tumor suppression activity.

To consolidate the importance of METTL14 in the occurrence and development of CRC, we generated Villin-Cre^{+}/Mettl14$^{FL/FL}$ mice (Fig EV2E). IHC staining validated the absence of METTL14 in the intestinal epithelium of Mettl14$^{\Delta IEC}$ mice without affecting its expression in other normally functioning organs (Figs 2D and EV2F). Colon carcinogenesis induced in mice by azoxymethane (AOM) is a useful model as it mimics the adenoma-carcinoma sequence observed in humans (Vivona *et al*, 1993) (Fig 2E). Whereas the AOM/ Dextran sodium sulfate (DSS) model is a powerful platform to employ when studying the pathogenesis of inflammatory CRC (Parang *et al*, 2016) (Fig 2E). In two distinct CRC models, mice from Mettl14$^{\Delta IEC}$ group were relatively emaciated compared with METTL14 wild-type (Mettl14^{WT}) group (Fig EV2G). Upon DSS challenge, Mettl14$^{\Delta IEC}$ group bore more aggravated weight loss than Mettl14^{WT} group (Fig EV2H). Moreover, METTL14 deletion resulted in shorter colons, more congested, and swollen spleens (Figs 2F and G, and EV2I). Histology showed more notable mucosal destruction, nuclear atypia tumor cells, and colitis in the intestinal epithelium of Mettl14$^{\Delta IEC}$ mice than that of Mettl14^{WT} mice (Fig EV2J). Vitally, Mettl14$^{\Delta IEC}$ mice showed both increased tumor numbers and enhancive tumor size (Fig 2H). As expected, Mettl14$^{\Delta IEC}$ mice had more histologic dysplasia compared with their Mettl14^{WT} littermates (Fig 2I). All of these data suggest that METTL14 deficiency promotes the pathogenesis and progression of p53-WT CRC.

METTL14 suppresses glycolysis of p53-WT CRC cells by targeting SLC2A3 and PGAM1

The Gene set enrichment analysis (GSEA) showed that cell proliferative signatures, apoptotic signatures, cell-cycle regulatory signatures, signatures of CRC occurrence, and glycolytic signatures were

 © 2023 The Authors

significantly enriched in p53-WT CRC but not p53-MT with high METTL14 expression (Fig 3A). An RNA sequencing (RNA-seq) analysis was performed to compare the gene expression profiles of the stable knockdown METTL14 group and control group in p53-WT and p53-MT cells. Firstly, the principal component analysis revealed that control and METTL14 knockdown-treated samples were clearly segregated, as expected (Fig EV3A). GSEA showed that besides the gene sets related to cell proliferation, cell-cycle, apoptosis, and CRC-specific signature, gene sets related to glycolysis were enriched in METTL14 knockdown group in p53-WT CRC cells (Figs 3B and EV3B). No significant correlation or even opposite trend between METTL14 knockdown and the above-mentioned signatures was identified in p53-MT cells (Fig EV3C). These data suggest that METTL14 might be related to wild-type p53 and serve as a key modulator of glycolytic metabolism to affect p53-WT CRC tumorigenesis.

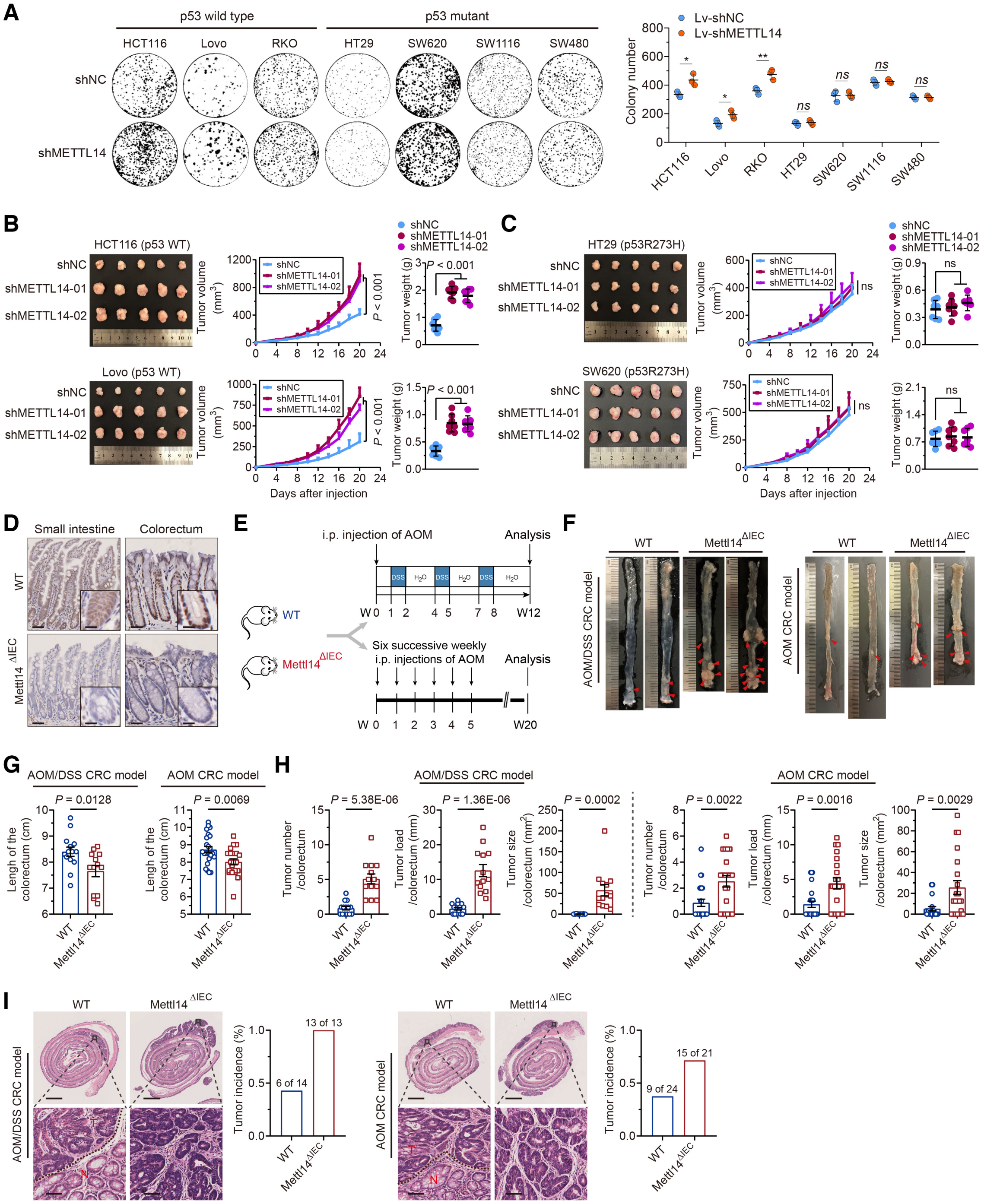

Figure 2.

Figure 2. METTL14 inhibits p53-WT CRC progression.

A Colony formation assay of p53-WT (HCT116, Lovo, and RKO) and p53-MT (HT29, SW620, SW1116, and SW480) cells stably infected with shNC or shMETTL14. Data are presented as mean ± SD (biological replicates, $n = 3$; $^*P < 0.05$, $^{**}P < 0.01$, ns = no significance).

B, C Representative images and analysis of tumors in nude mice bearing stably transfected shNC or shMETTL14 p53-WT (HCT116 and Lovo) and p53-MT (HT29 and SW620) cells. Data are presented as mean ± SD (biological replicates, $n = 7$, ns = no significance).

D Representative IHC staining images of METTL14 in $Mettl14^{WT}$ and $Mettl14^{\Delta IEC}$ mice. The insets show enlarged images of small intestine tissues and colorectal tissues, respectively. Scale bars = 50 μm and 5 μm (inset).

E Schematic diagrams of AOM/DSS-induced and AOM-induced CRC models. i.p., intraperitoneal.

F Colorectum was opened longitudinally, and two representative colorectal images derived from $METTL14^{WT}$ and $METTL14^{\Delta IEC}$ mice from AOM/DSS-induced and AOM-induced CRC models, respectively.

G Comparison of colorectum length between $METTL14^{WT}$ ($n = 14$, $n = 24$) and $METTL14^{\Delta IEC}$ ($n = 13$, $n = 21$) mice from AOM/DSS-induced and AOM-induced CRC models, respectively. Data are expressed as mean ± SD.

H Comparison of tumor number, tumor load, and tumor size between $METTL14^{WT}$ ($n = 14$, $n = 24$) and $METTL14^{\Delta IEC}$ ($n = 14$, $n = 24$) mice from AOM/DSS-induced and AOM-induced CRC models, respectively. Data are expressed as mean ± SD.

I Representative HE staining images of colorectum in $METTL14^{WT}$ ($n = 14$, $n = 24$) and $METTL14^{\Delta IEC}$ ($n = 13$, $n = 21$) mice from AOM/DSS-induced and AOM-induced CRC models. Lower panels show enlarged images of indicated normal or CRC tissues. Scale bars = 2 mm (upper) and 40 μm (lower). Black dashed line refers to the border of tumor (T) and normal (N) tissues. Tumors are classified as adenomas with low to focal high-grade dysplasia. The percentages of mice with dysplasia are shown (right).

Data information: For (A–C, G, H), statistical significance was determined by a two-tailed Student's t-test.

METTL14 overexpression significantly reduced glucose uptake, lactate production, ATP, and pyruvate levels, while METTL14 knockdown led to the opposite results in p53-WT cells (Figs 3C and EV3D). Furthermore, ectopic METTL14 expression also displayed decreased extracellular acidification rate (ECAR), which reflects overall glycolytic flux, and increased oxygen consumption rate (OCR), an indicator of mitochondrial oxidative respiration in p53-WT cells (Figs 3D and EV3E). The heatmap clustering analysis showed that METTL14 down-regulation resulted in the significant alternation of glucose transporters and glycolytic enzymes in p53-WT CRC cells (Fig 3E). In p53-MT CRC cells; however, METTL14 shows no significant or a negative impact on any glycolysis-associated gene regulated by METTL14 in p53-WT CRC cells, echoing the results of the GSEA analysis (Fig 3E). As further confirmation, we found that only SLC2A3 and PGAM1 mRNA and protein were regulated by METTL14 (Figs 3F and G, and EV3F). Strikingly, no significant differences in alteration of ECAR and OCR, as well as the expression levels of SLC2A3 and PGAM1 were identified in p53-MT cells accompanied by METTL14 overexpression or silencing (Fig EV3G and H). Besides, METTL14 knockdown by small interfering RNA (siRNA) up-regulated SLC2A3 and PGAM1 expression at protein levels by IF staining (Fig EV3I). As expected, the isolated intestinal epithelium lysates of $Mettl14^{\Delta IEC}$ mice exhibited higher levels of SLC2A3 and PGAM1 than $Mettl14^{WT}$ mice by western blot analysis (Fig 3H). IHC analysis also showed increased SLC2A3 and PGAM1 staining in tissues from $Mettl13^{\Delta IEC}$ compared with that from $Mettl14^{WT}$ mice (Fig 3I and Appendix Fig S4). Similarly, IECs isolated from $Mettl14^{\Delta IEC}$ mice possessed higher ATP

Figure 3. METTL14 suppresses glycolysis of p53-WT CRC cells by targeting SLC2A3 and PGAM1.

A GSEA comparison between patients with METTL14 high expression and METTL14 low expression in p53-WT and p53-MT CRC from TCGA CRC dataset, respectively. Positive or negative normalized enrichment scores (NES) correspond to the enrichment of a given set in genes that are up- or down-regulated, respectively, in response to high METTL14 expression. Data are presented as pseudo-heatmap with NES magnitude color-coded as indicated in the legend; all light red or nongray comparisons have False Discovery Rate q-value (FDR q) < 0.05.

B Overview of GSEA identified the differential gene profiles between stably transfected shNC ($n = 4$), shMETTL14-01 ($n = 2$), and shMETTL14-02 ($n = 2$) HCT116 (p53-WT) cells, respectively. The sum of the numbers in the small circles of different colors is the total number of genes in each gene sets related to cell proliferation, cell-cycle, glycolysis, and metabolic process. The number where the two circles intersect is the total number of genes that are both regulated by METTL14 and belong to the gene set related to different signatures.

C Lactate production, ATP level, glucose uptake, and pyruvate level in stably transfected Lv-vector and Lv-METTL14 or shNC and shMETTL14 HCT116 (p53-WT) cells. Data are presented as mean ± SD (biological replicates, $n = 3$; $^{**}P < 0.01$).

D ECAR and OCR in stably transfected Lv-vector and Lv-METTL14 HCT116 (p53-WT) cells. Data are presented as mean ± SD (biological replicates, $n = 3$; $^{**}P < 0.01$).

E Schematic diagram of aerobic glycolysis pathway (left) and heatmap of crucial glycolytic genes involved in aerobic glycolysis pathway (right). Significantly differentially expressed genes were identified by DESeq2 under the requirement of Adjusted P-value (adj.P.Val) < 0.05, 0.001 < adj.P.Val < 0.01, or adj.P.Val < 0.001.

F, G Glycolytic gene expression in CRC cells transfected with empty vector or METTL14 plasmid for 48 h by qRT–PCR and western blot. Data are presented as mean ± SD (biological replicates, $n = 3$; $^*P < 0.05$, $^{**}P < 0.01$, $^{***}P < 0.001$).

H Western blot analysis of METTL14, SLC2A3, and PGAM1 protein levels of intestinal epithelial cells from AOM/DSS-induced $Mettl14^{WT}$ ($n = 4$) and $Mettl14^{\Delta IEC}$ ($n = 4$) and mice.

I Representative IHC staining images and quantitative analysis of SLC2A3 and PGAM1 in tumor tissues and nontumor tissues from AOM/DSS-induced $Mettl14^{\Delta IEC}$ and $Mettl14^{WT}$ mice CRC models. The insets show enlarged images of tumor tissues and nontumor tissues, respectively. Scale bars = 40 μm and 4 μm (inset). Data are presented as mean ± SD (biological replicates, $n = 6$; $^{**}P < 0.01$, $^{***}P < 0.001$).

J, K ATP level, lactate production, pyruvate level, and PGAM1 activity in intestinal epithelial cells isolated from AOM/DSS-induced $Mettl14^{WT}$ ($n = 9$) and $Mettl14^{\Delta IEC}$ ($n = 10$) mice. Data are presented as mean ± SD.

Data information: For (C, D, F, I–K), statistical significance was determined by a two-tailed Student's t-test. ACTB was used as a loading control.
Source data are available online for this figure.

© 2023 The Authors

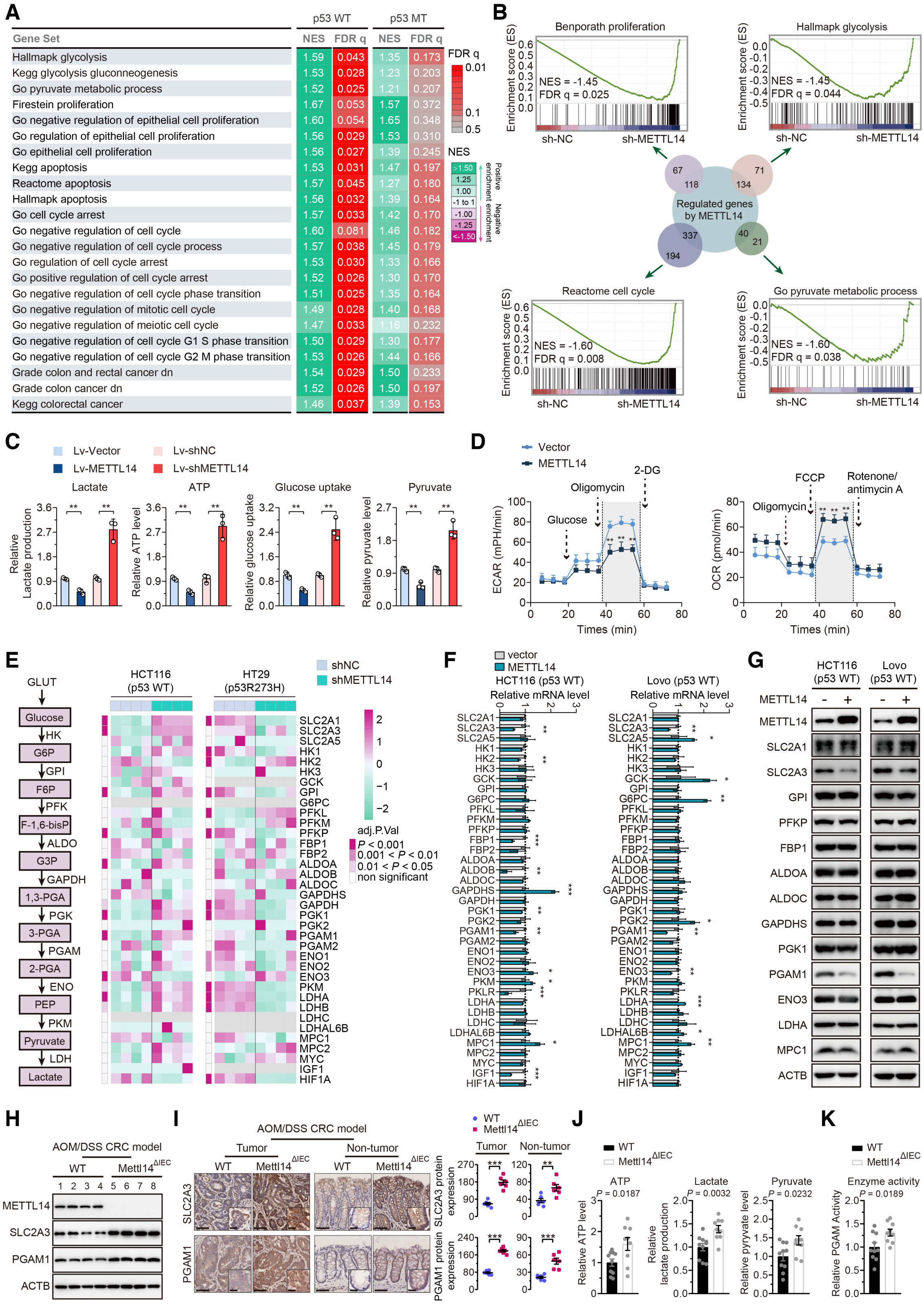
A
p53 WT
p53 MT
Gene Set | NES | FDR q | NES | FDR q
Hallmapk glycolysis 1.59 0.043 1.35 0.173
Kegg glycolysis gluconneogenesis 1.53 0.028 1.23 0.203
Go pyruvate metabolic process 1.52 0.025 1.21 0.207
Firestein proliferation 1.67 0.053 1.57 0.372
Go negative regulation of epithelial cell proliferation 1.60 0.054 1.65 0.348
Go regulation of epithelial cell proliferation 1.56 0.029 1.53 0.310
Go epithelial cell proliferation 1.56 0.027 1.39 0.245
Kegg apoptosis 1.53 0.031 1.47 0.197
Reactome apoptosis 1.57 0.045 1.27 0.180
Hallmapk apoptosis 1.56 0.032 1.39 0.164
Go cell cycle arrest 1.57 0.033 1.42 0.170
Go negative regulation of cell cycle 1.60 0.081 1.46 0.182
Go negative regulation of cell cycle process 1.57 0.038 1.45 0.179
Go regulation of cell cycle arrest 1.53 0.030 1.33 0.166
Go positive regulation of cell cycle arrest 1.52 0.026 1.30 0.170
Go negative regulation of cell cycle phase transition 1.51 0.025 1.35 0.164
Go negative regulation of mitotic cell cycle 1.49 0.028 1.40 0.168
Go negative regulation of meiotic cell cycle 1.47 0.033 1.16 0.232
Go negative regulation of cell cycle G1 S phase transition 1.50 0.029 1.30 0.177
Go negative regulation of cell cycle G2 M phase transition 1.53 0.026 1.44 0.166
Grade colon and rectal cancer dn 1.54 0.029 1.50 0.233
Grade colon cancer dn 1.52 0.026 1.50 0.197
Kegg colorectal cancer 1.46 0.037 1.39 0.153
FDR q
NES
B
Benporath proliferation
NES = -1.45
FDR q = 0.025
Hallmapk glycolysis
NES = -1.45
FDR q = 0.044
Regulated genes by METTL14
Reactome cell cycle
NES = -1.60
FDR q = 0.008
Go pyruvate metabolic process
NES = -1.60
FDR q = 0.038
Enrichment score (ES)
sh-NC
sh-METTL14
C
Lv-Vector
Lv-METTL14
Lv-shNC
Lv-shMETTL14
Lactate
ATP
Glucose uptake
Pyruvate
Relative Lactate production
Relative ATP level
Relative glucose uptake
Relative pyruvate level
D
Vector
METTL14
Glucose
Oligomycin
2-DG
FCCP
Rotenone/ antimycin A
ECAR (mPH/min)
OCR (pmol/min)
Times (min)
E
GLUT
Glucose
HK
G6P
GPI
F6P
PFK
F-1,6-bisP
ALDO
G3P
GAPDH
1,3-PGA
PGK
3-PGA
PGAM
2-PGA
ENO
PEP
PKM
Pyruvate
LDH
Lactate
HCT116 (p53 WT)
HT29 (p53R273H)
shNC
shMETTL14
adj.P.Val
P < 0.001
0.001 < P < 0.01
0.01 < P < 0.05
non significant
F
vector
METTL14
HCT116 (p53 WT)
Lovo (p53 WT)
Relative mRNA level
G
HCT116 (p53 WT)
Lovo (p53 WT)
METTL14
SLC2A1
SLC2A3
GPI
PFKP
FBP1
ALDOA
ALDOC
GAPDHS
PGK1
PGAM1
ENO3
LDHA
MPC1
ACTB
H
AOM/DSS CRC model
WT
Mettl14ΔIEC
I
AOM/DSS CRC model
Tumor
Non-tumor
SLC2A3
PGAM1
SLC2A3 protein expression
PGAM1 protein expression
J
WT
Mettl14ΔIEC
ATP
P = 0.0187
Lactate
P = 0.0032
Pyruvate
P = 0.0232
Relative ATP level
Relative lactate production
Relative pyruvate level
K
Enzyme activity
P = 0.0189
Relative PGAM Activity

Figure 3.

level, lactate production, and pyruvate level than Mettl14^{WT} mice (Fig 3J). Accordingly, a higher enzyme activity was found in Mettl14$^{\Delta IEC}$ mice than Mettl14^{WT} mice (Fig 3K). In summary, up-regulation of METTL14 suppresses glycolysis by down-regulating SLC2A3 and PGAM1, thus impeding Warburg effect of p53-WT CRC cells, sequentially attenuating CRC tumorigenesis in the context of wild-type p53 status.

METTL14 decreases SLC2A3 and PGAM1 levels by m^{6}A-YTHDF2-mediated pri-miR-6769b and pri-miR-499a processing

Considering the important role of m^{6}A methyltransferase and DGCR8 in the miRNA biogenesis, we confirmed physical interaction between the endogenous METTL14 and DGCR8 proteins, and ribonuclease (RNase) treatment weakened the interaction between METTL14 and DGCR8, suggesting that their interaction might be partly mediated by RNAs (Fig 4A). We then conducted genome-wide miRNA expression profiling of p53-WT and p53-MT cells with stable overexpression of METTL14 and control transfectants. Among them, 90 miRNAs were up-regulated in the HCT116 cells with METTL14 overexpression compared with control (Fig EV4A). We then used the TargetScan and miRDB databases to identify potential miRNAs that may regulate SLC2A3 and PGAM1. After overlapping these potential SLC2A3- and PGAM1-regulatory miRNAs with the identified 90 up-regulated miRNAs, we found four and two miRNAs that may regulate SLC2A3 and PGAM1, respectively (Fig EV4A). KEGG enrichment analysis further revealed that the top six enriched biological signatures included pyruvate metabolism and glycolysis/gluconeogenesis in p53-WT cells (Fig 4B), whereas no such significant relationship was identified in p53-MT cells (Fig 4C). These results suggest that METTL14 might directly modulate glycolysis-related miRNAs to regulate SLC2A3 and PGAM1 expression levels. Among these miRNAs, miR-6769b-3p, miR-1321, miR-7160-5p, miR-380-3p and miR-499a-3p were up-regulated after METTL14 overexpression (Fig 4D). Moreover, PCR and western blot assay demonstrated that only METTL14-regulated miR-499a-3p and miR-6769b-3p led to decreased expression of PGAM1 and SLC2A3 levels separately in p53-WT cells (Fig 4E and F, and EV4B). Importantly, we found that METTL14 could not modulate miR-6769b-3p and miR-499a-3p demonstrated by miRNA microarrays and PCR assay in p53-MT cells (Fig EV4C and D). Consistently, miR-6769b-3p and miR-499a-3p display no significant impact on SLC2A3 and PGAM1 in p53-MT cells (Figs EV4E and 4G). Luciferase reporter assay showed that all four predicted binding sites in SLC2A3 3′UTR and the predicted binding site 1 in PGAM1 3′UTR were required for interactions of miR-6769b-3p/SLC2A3 and miR-499a-3p/PGAM1 (Figs 4H and EV4F).

The potential mechanisms responsible for the up-regulation of miR-6769b-3p and miR-499a-3p via METTL14 in p53-WT cells were investigated. *In situ* hybridization (ISH) and PCR assay showed METTL14 silencing increased pri-miR-6769b and pri-miR-499a levels, while METTL14 did not modulate the expression of miR-6769b-3p/miR-499a-3p and pri-miR-6769b/pri-miR-499a in p53-MT CRC cells (Figs 4I and EV4G and H). Considering that eight mainly different readers (IGF2BP1-3, YTHDF1-3, YTHDC1, and YTHDC2) of m^{6}A modification have been identified, we explored which reader

Figure 4. METTL14 decreases SLC2A3 and PGAM1 levels by m^{6}A-YTHDF2-mediated pri-miR-6769b and pri-miR-499a processing.

- A Co-IP of METTL14 and DGCR8 in HCT116 cells in the presence or absence of ribonuclease.
- B KEGG enrichment analysis of differential expression of miRNA target genes by miRWalk (http://mirwalk.umm.uni-heidelberg.de/) and miRDB database (http://mirdb.org/) in p53-WT HCT116 cells.
- C KEGG enrichment analysis of differential expression of miRNA target genes by miRWalk and miRDB database in p53-MT HT29 cells.
- D qRT–PCR analysis of the miRNA levels in stably transfected vector and METTL14 HCT116 (p53-WT) cells. Data are presented as mean ± SD (biological replicates, $n = 3$; ns = no significance, $^*P < 0.05$, $^{**}P < 0.01$, $^{***}P < 0.001$).
- E Western blot analysis of SLC2A3 and PGAM1 protein levels in HCT116 and Lovo (p53-WT) cells transfected with control or miRNA mimics.
- F Representative IF staining and quantitative analysis of the SLC2A3 (green) and PGAM1 (green) proteins in HCT116 (p53-WT) cells transfected with control or miRNA mimics. Nuclei were stained with DAPI (blue). Scale bars = 20 μm. Data are presented as mean ± SD (biological replicates, $n = 4$; $^{**}P < 0.01$).
- G Representative IF staining and quantitative analysis of the SLC2A3 (green) and PGAM1 (green) proteins in HT29 (p53-MT) cells transfected with control or miRNA mimics. Nuclei were stained with DAPI (blue). Scale bars = 20 μm. Data are presented as mean ± SD (biological replicates, $n = 4$; ns = no significance).
- H Luciferase activity of reporters expressing wild-type or mutant SLC2A3 and PGAM1 3′UTRs in HCT116 (p53-WT) cells transfected with control or miRNA (miR-6769b-3p and miR-499a-3p) mimics. Data are presented as mean ± SD (biological replicates $n = 4$; ns = no significance, $^*P < 0.05$, $^{***}P < 0.001$).
- I Representative ISH images and quantitative analysis of miR-6769b-3p and miR-499a-3p in tumor tissues from AOM/DSS-induced Mettl14$^{\Delta IEC}$ and Mettl14^{WT} mice CRC models. The insets show enlarged images of tumor tissues. Scale bars = 20 μm and 2 μm (inset). Data are presented as mean ± SD (biological replicates, $n = 6$; $^{***}P < 0.001$).
- J qRT–PCR analysis of the pri-miR-6769b and pri-miR-499a levels in stably transfected Lv-vector and Lv-METTL14 HCT116 cells with control, YTHDF1-3, YTHDC1-2 or IGF2BP1-3 siRNAs. Data are presented as mean ± SD (biological replicates, $n = 3$; $^{**}P < 0.01$). The dotted line represents basal mRNA expression of pri-miR-6769b and pri-miR-499a in HCT116 cells, acting as a control.
- K RNA stability of pri-miR-6769b and pri-miR-499a in stably transfected shNC and shMETTL14 HCT116 (p53-WT) cells with or without YTHDF2 overexpression. The data are presented as mean ± SD (biological replicates, $n = 3$, $^*P < 0.05$, $^{**}P < 0.01$).
- L RIP and qRT–PCR assays detected the enrichment of pri-miR-6769b and pri-miR-499a to YTHDF2 in stably transfected Lv-vector and Lv-METTL14 HCT116 cells. Data are presented as mean ± SD (biological replicates, $n = 3$).
- M MeRIP–qPCR assay detected m^{6}A modification on pri-miR-6769b and pri-miR-499a in stably transfected vector and METTL14 HCT116 cells. Data are presented as mean ± SD (biological replicates, $n = 3$).
- N Representative images of tumors and analysis in nude mice intervened with control, miR-6769b-3p or miR-499a-3p expression p53-WT (HCT116 and Lovo) cells. Data are presented as mean ± SD (biological replicates, $n = 7$).
- O Representative images of tumors and analysis in nude mice intervened with control, miR-6769b-3p or miR-499a-3p expression p53-MT (HT29 and SW620) cells. Data are presented as mean ± SD (biological replicates, $n = 7$, ns, no significance).

Data information: For (D, F–O), statistical significance was determined by a two-tailed Student's *t*-test. ACTB was used as a loading control.
Source data are available online for this figure.

© 2023 The Authors

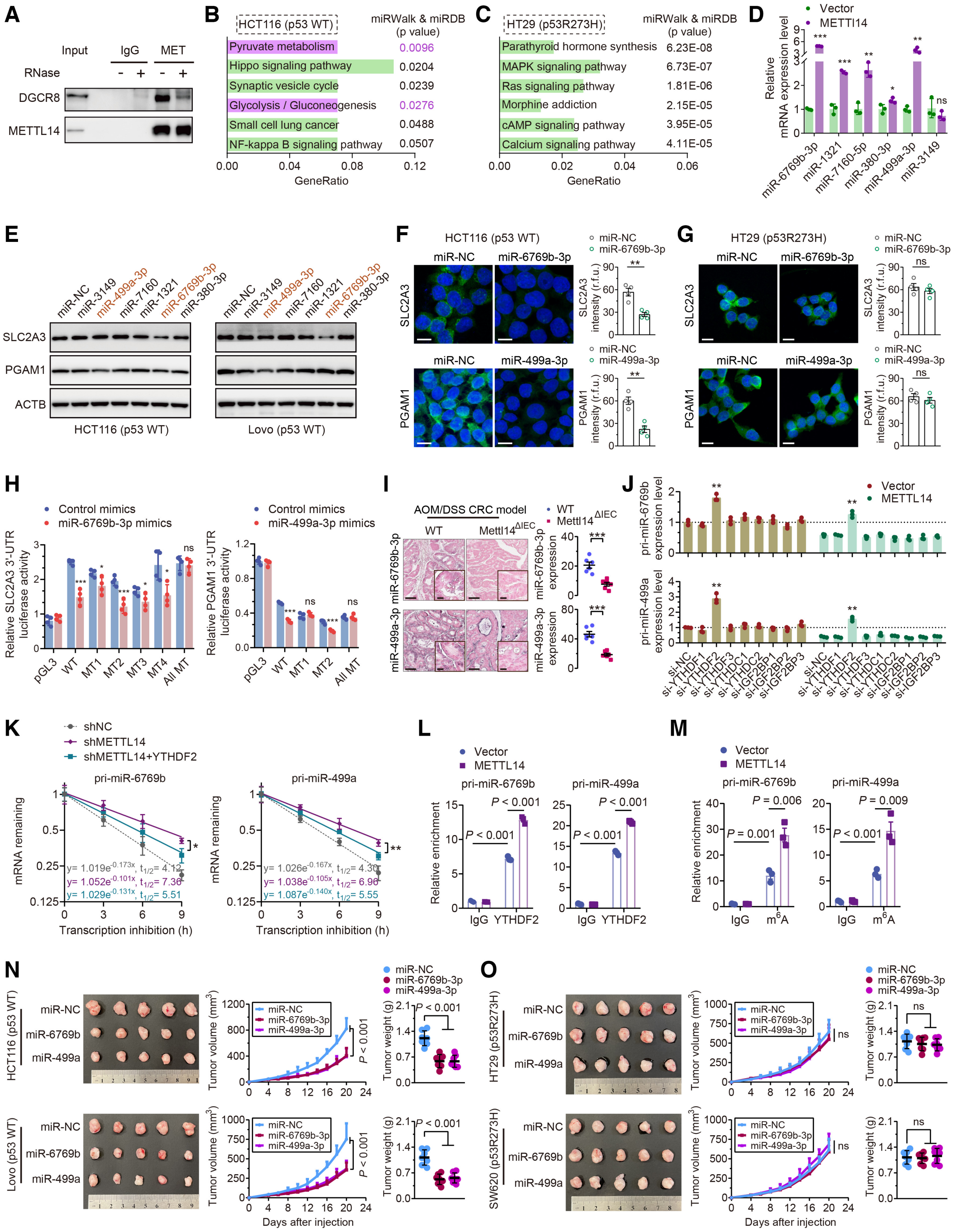

Figure 4.

or readers could specifically recognize pri-miR-6769b and pri-miR499a. We found that the negative regulation of METTL14 overexpression on pri-miR-6769b and pri-miR-499a levels could only be restored by YTHDF2 knockdown instead of other readers (Fig 4J). YTHDF2 has been proved to preferentially recognize m^6A-modified mRNA and mediate mRNA stability in many biological contexts (Wang et al, 2014; Chen et al, 2018). METTL14 down-regulation enhanced the RNA stability of pri-miR-6769b and pri-miR-499a (Fig 4K). Furthermore, ectopic expression of YTHDF2 partly antagonized increased mRNA stability in METTL14 silencing cells (Fig 4K). To determine whether m^6A sites participated in the modulation of pri-miR-6769b and pri-miR-499a expression, we constructed

© 2023 The Authors

expression vectors containing m^{6}A motif mutants and wild types using SRAMP database (Fig EV4I). As a result, All MT or pri-miR-6769b-MT4/pri-miR-499a-MT1 plasmids resulted in significant increases in pri-miR-6769b/pri-miR-499a levels and decreases in pre-miR-6769b/pre-miR-499a and miR-6769b-3p/miR-499a-3p levels compared with plasmids bearing intact m^{6}A sites (Fig EV4J and K). Additionally, cells transfected with mutant METTL14 plasmids (MT-METTL14, R298P) had no effect on pri-miRNA processing, SLC2A3 and PGAM1 protein levels, or global m^{6}A levels (Fig EV4L and M). Moreover, we observed enhanced interaction between pri-miR-6769b/pri-miR-499a and YTHDF2 in METTL14-overexpressing cells (Fig 4L). MeRIP–qPCR assay further affirmed the presence of m^{6}A methylation on pri-miR-6769b/pri-miR-499a and METTL14 up-regulation triggering greater m^{6}A modification on pri-miR-6769b/pri-miR-499a (Fig 4M). Further, we confirmed that miR-6769b-3p/miR-499a-3p up-regulation suppressed p53-WT tumor growth *in vivo* (Fig 4N), while xenograft tumors from p53-MT CRC cells expressing control or miR-6769b-3p/miR-499a-3p exhibited no significant differences (Fig 4O). Collectively, METTL14 may suppress the development of p53-WT CRC by selectively facilitating the processing of pri-miR-6769b and pri-miR-499a in an m^{6}A-YTHDF2-dependent manner.

METTL14 attenuates the development of p53-WT CRC by repressing glycolysis through miR-6769b-3p/SLC2A3 and miR-499a-3p /PGAM1 axis

The selective maturation of miR-6769b-3p and miR-499a-3p in a METTL14-mediated m^{6}A-dependent manner led us to hypothesize that miR-6769b-3p and miR-499a-3p may regulate METTL14-controlled glycolysis reprogramming in p53-WT CRC cells. Above all, we demonstrated that miR-6769b-3p and miR-499a-3p mimics decreased glucose uptake, lactate, ATP, and pyruvate levels in HCT116 cells, while miR-6769b-3p and miR-499a-3p inhibitors showed the opposite effects (Fig 5A and B). Moreover, both miR-6769b-3p and miR-499a-3p mimics displayed decreased ECAR and increased OCR in HCT116 cells (Fig 5C and D). As expected, miR-6769b-3p and miR-499a-3p did not affect metabolic phenotype in p53-MT CRC cells (Fig EV5A–D). In addition, mixture inhibitors rescued decreased cellular glycolytic function caused by METTL14 overexpression in p53-WT CRC cells (Fig 5E). Mixture mimics abolished reduced glucose uptake, lactate, ATP, and pyruvate levels caused by METTL14 overexpression in p53-WT CRC cells (Fig 5F). Furthermore, mixture inhibitors obviously reversed the effects on ECAR and OCR caused by METTL14 overexpression (Fig 5G) and mixture mimics remarkably attenuated glycolysis, which also abolished the effects of METTL14 overexpression on ECAR and OCR (Fig 5H).

To examine the effects of the METTL14/miR-6769b-3p and METTL14/miR-499a-3p axis on glycolysis *in vitro* and *in vivo*, we verified that stable miR-6769b-3p and miR-499a-3p overexpression inhibited tumor growth, and miR-6769b-3p and miR-499a-3p up-regulation antagonized the tumor growth-promoting effect caused by METTL14 knockdown *in vivo* (Figs EV5E and F, and 5I–K). We then found that SLC2A3/PGAM1 down-regulation suppressed the proliferation of p53-WT CRC cells but had no obvious effects on cell proliferation in p53-MT CRC cells (Fig EV5G). Moreover, we found that the expression level of SLC2A3/PGAM1 was lower in p53-WT CRC cell line, whereas miR-6769b-3p/miR-499a-3p expression levels were higher in the p53-WT CRC cell line (Fig EV5H and I). Moreover, the stable knockdown of SLC2A3/PGAM1 in CRC cells demonstrated a significant reduction in tumor volume and weight derived

Figure 5. METTL14 attenuates the development of p53-WT CRC by repressing glycolysis through miR-6769b-3p/SLC2A3 and miR-499a-3p /PGAM1 axis. ▶

A Glucose uptake, lactate, ATP, and pyruvate levels were determined in HCT116 (p53-WT) cells treated with control, miR-6769b-3p mimics, or inhibitor for 48 h. Data are presented as mean ± SD (biological replicates, $n = 3$, *$P < 0.05$, **$P < 0.01$).

B Glucose uptake, lactate, ATP, and pyruvate levels were determined in HCT116 (p53-WT) cells treated with control, miR-499a-3p mimics, or inhibitor for 48 h. Data are presented as mean ± SD (biological replicates, $n = 3$, *$P < 0.05$, **$P < 0.01$).

C ECAR and OCR were determined in HCT116 (p53-WT) cells treated with control, miR-6769b-3p mimics, or inhibitor for 48 h. Data are presented as mean ± SD (biological replicates, $n = 3$, *$P < 0.05$, **$P < 0.01$).

D ECAR and OCR were determined in HCT116 (p53-WT) cells treated with control, miR-499a-3p mimics, or inhibitor for 48 h. Data are presented as mean ± SD (biological replicates, $n = 3$, *$P < 0.05$).

E Glucose uptake, Lactate, ATP, and pyruvate levels were determined in stably transfected Lv-vector and Lv-METTL14 HCT116 (p53-WT) cells treated with control or mixture inhibitors for 48 h. Western blot analysis of the corresponding METTL14, SLC2A3, and PGAM1 protein levels in indicated treatment. Data are presented as mean ± SD (biological replicates $n = 3$, ns = no significance, **$P < 0.01$).

F Glucose uptake, Lactate, ATP, and pyruvate levels were determined in stably transfected Lv-vector and Lv-METTL14 HCT116 (p53-WT) cells treated with control or mixture mimics for 48 h. Western blot analysis of the corresponding METTL14, SLC2A3, and PGAM1 protein levels in indicated treatment. Data are presented as mean ± SD (biological replicates, $n = 3$, ns = no significance, **$P < 0.01$).

G ECAR and OCR were determined in stably transfected Lv-vector and Lv-METTL14 HCT116 (p53-WT) cells treated with control or mixture inhibitors for 48 h. Data are presented as mean ± SD (biological replicates, $n = 3$, ns = no significance, **$P < 0.01$).

H ECAR and OCR were determined in stably transfected Lv-vector and Lv-METTL14 HCT116 (p53-WT) cells treated with control or mixture mimics for 48 h. Data are presented as mean ± SD (biological replicates $n = 3$, ns = no significance, **$P < 0.01$).

I, J Representative images and analysis of tumors in nude mice generated by stably transfected shNC and shMETTL14 or miR-6769b-3p and miR-499a-3p overexpressing HCT116 (p53-WT) cells with or without METTL14 knockdown. Data are presented as mean ± SD (biological replicates, $n = 7$, ***$P < 0.001$).

K Western blot analysis of the expression of METTL14, SLC2A3, and PGAM1 in indicated tumor tissues.

L, M Representative images and analysis of tumors in nude mice generated by stably transfected shSLC2A3 and shPGAM1 HCT116 (p53-WT) cells with or without METTL14 knockdown, and tumors intervened with 2-DG and 3-BP with or without METTL14 knockdown. Data are presented as mean ± SD (biological replicates, $n = 7$, **$P < 0.01$, ***$P < 0.001$).

N Western blot analysis of the expression of METTL14, HK1, SLC2A3, and PGAM1 in indicated tumor tissues.

Data information: For (A–J, L, M), statistical significance was determined by a two-tailed *t*-test. ACTB was used as a loading control.
Source data are available online for this figure.

© 2023 The Authors

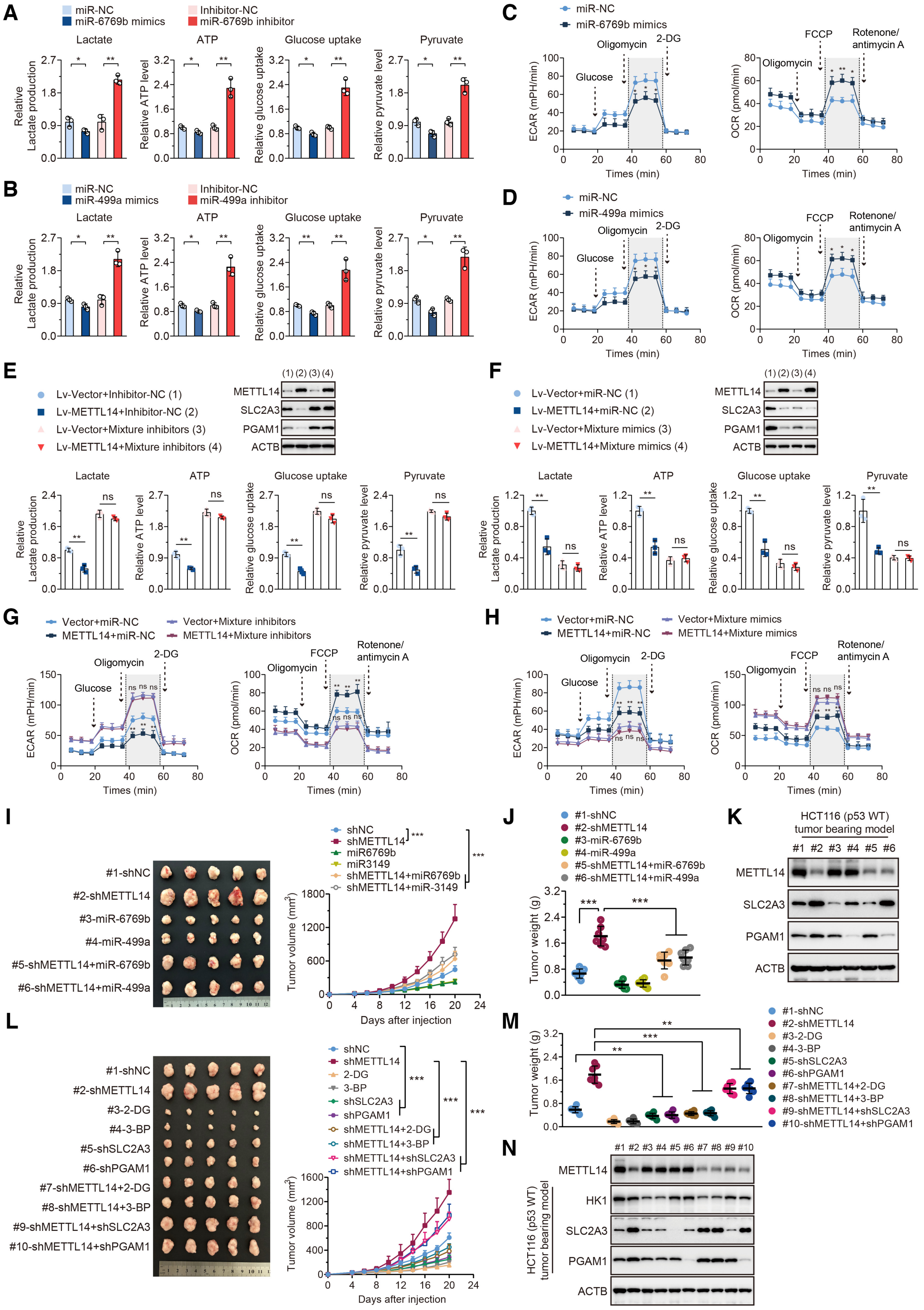
A
miR-NC
miR-6769b mimics
Inhibitor-NC
miR-6769b inhibitor
Lactate
ATP
Glucose uptake
Pyruvate
Relative Lactate production
Relative ATP level
Relative glucose uptake
Relative pyruvate level
B
miR-NC
miR-499a mimics
Inhibitor-NC
miR-499a inhibitor
C
miR-NC
miR-6769b mimics
Glucose
Oligomycin
2-DG
FCCP
Rotenone/ antimycin A
ECAR (mPH/min)
OCR (pmol/min)
Times (min)
D
miR-NC
miR-499a mimics
E
Lv-Vector+Inhibitor-NC (1)
Lv-METTL14+Inhibitor-NC (2)
Lv-Vector+Mixture inhibitors (3)
Lv-METTL14+Mixture inhibitors (4)
METTL14
SLC2A3
PGAM1
ACTB
F
Lv-Vector+miR-NC (1)
Lv-METTL14+miR-NC (2)
Lv-Vector+Mixture mimics (3)
Lv-METTL14+Mixture mimics (4)
G
Vector+miR-NC
METTL14+miR-NC
Vector+Mixture inhibitors
METTL14+Mixture inhibitors
H
Vector+miR-NC
METTL14+miR-NC
Vector+Mixture mimics
METTL14+Mixture mimics
I
#1-shNC
#2-shMETTL14
#3-miR-6769b
#4-miR-499a
#5-shMETTL14+miR-6769b
#6-shMETTL14+miR-499a
shNC
shMETTL14
miR6769b
miR3149
shMETTL14+miR6769b
shMETTL14+miR-3149
Tumor volume (mm³)
Days after injection
J
#1-shNC
#2-shMETTL14
#3-miR-6769b
#4-miR-499a
#5-shMETTL14+miR-6769b
#6-shMETTL14+miR-499a
Tumor weight (g)
K
HCT116 (p53 WT) tumor bearing model
METTL14
SLC2A3
PGAM1
ACTB
L
#1-shNC
#2-shMETTL14
#3-2-DG
#4-3-BP
#5-shSLC2A3
#6-shPGAM1
#7-shMETTL14+2-DG
#8-shMETTL14+3-BP
#9-shMETTL14+shSLC2A3
#10-shMETTL14+shPGAM1
shNC
shMETTL14
2-DG
3-BP
shSLC2A3
shPGAM1
shMETTL14+2-DG
shMETTL14+3-BP
shMETTL14+shSLC2A3
shMETTL14+shPGAM1
M
N
HCT116 (p53 WT) tumor bearing model
METTL14
HK1
SLC2A3
PGAM1
ACTB

Figure 5.

from p53-WT cells compared with those from control-treated cells, whereas the p53-MT xenograft did not respond to the stable knockdown of SLC2A3/PGAM1 (Fig EV5J and K). Finally, we tested whether glycolysis plays a role in METTL14/miR-6769b-3p and METTL14/miR-499a-3p axis-mediated regulation of tumor growth *in vivo*. As expected, the glycolytic inhibitors 2-deoxy-D-glucose (2-DG) and 3-bromopyruvate (3-BP) (Xu *et al*, 2005) dramatically reduced tumor growth (Fig 5L and M). These results were further confirmed by the changes in protein levels of SLC2A3 and PGAM1, and HK1, as therapeutic targets of 2-DG and 3-BP (Pajak *et al*, 2019), by western blot analysis from tumor tissues (Fig 5N). Moreover, 2-DG and 3-BP, as well as SLC2A3 and PGAM1 downregulation, reversed the acceleration of tumor growth caused by METTL14 knockdown (Fig 5L–N). Taken together, METTL14 suppresses p53-WT CRC progression by inhibiting glycolysis through miR-6769b-3p/SLC2A3 and miR-499a-3p/PGAM1 pathways.

METTL14, miR-6769b-3p/SLC2A3 and miR-499a-3p/PGAM1 are clinically relevant in p53-WT CRC patients

To investigate the clinical significance of METTL14, glycolytic components (SLC2A3 and PGAM1), and microRNAs (miR-6769b-3p and miR-499a-3p) in CRC patients, IHC, ISH, and real-time PCR analysis showed that the levels of SLC2A3 and PGAM1 were higher in CRC tissues than those in normal tissues, while the levels of miR-6769b-3p and miR-499a-3p were higher in adjacently normal tissues (Appendix Fig S5A–D). In parallel, we confirmed that p53-WT CRC had much higher levels of miR-6769b-3p/miR-499a-3p and much lower levels of SLC2A3/PGAM1 than p53-MT samples (Fig 6A). Our results also showed expression of other glucose transporter SLC2A1 and phosphoglycerate mutase PGAM2 did not differ between the two groups (Appendix Fig S6). Furthermore, the samples with higher METTL14 expression displayed strong staining for miR-6769b-3p and miR-499a-3p and weak staining for SLC2A3 and PGAM1, while samples with low expression of METTL14 were with low levels of miR-6769b-3p and miR-499a-3p, and high levels of SLC2A3 and PGAM1 in p53-WT CRC samples (Fig 6B and C). However, miR-6769b-3p/miR-499a-3p and SLC2A3/PGAM1 did not show a correlation with METTL14 expression in p53-MT CRC samples, and miR-6769b-3p/miR-499a-3p and SLC2A3/PGAM1 levels were also not correlated in p53-MT CRC samples (Appendix Fig S7A and B). Moreover, we mined data from DepMap portal (Meyers *et al*, 2017), a database comprising many perturbation datasets from hundreds of human cancer cell lines. We specifically inquired the functional effect of METTL14 knockout by CRISPR-Cas9 in various human cancer cell lines. We parsed the cell lines based on p53 status into either wild-type or mutant and plotted the gene effect score, termed the Achilles score that reflects the essentiality of individual genes for proliferation (Bieging-Rolett *et al*, 2020; Raj *et al*, 2022). Interestingly, the Achilles scores for METTL14 were obviously higher in p53-WT cancer cell lines than in cancer cell lines harboring p53 mutations and p53-MT cancer cell lines were less affected by METTL14 perturbation than p53-WT cancer cell lines (Fig 6D). Moreover, the top METTL14 co-dependencies include not only p53 and the p53-positive modulators ATM and TP53BP1, as well as p53 main effector $p21^{WAF1/Cip1}$ (with Achilles score positively correlated with that of METTL14) but also p53-negative modulators MDM2 and PPM1D (with Achilles score negatively correlated with that of METTL14) in p53-WT cancer cells lines or whole cancer cell lines, whereas the significant correlations between METTL14 and p53 disappeared, and the correlations between METTL14 and regulators and effector of p53 tended to get weak in p53-MT cell lines (Fig 6E). As expected, METTL14 and KRAS Achilles scores were not correlated in cancer cell lines among these groups (Fig 5E).

We also showed that higher levels of METTL14, miR-6769b-3p, and miR-499a-3p predicted a better prognosis, but the elevated expression of SLC2A3 and PGAM1 exhibited robustly shorter OS in patients with p53-WT CRC (Fig 6F and Appendix Fig S8A). However, METTL14, miR-6769b-3p/miR-499a-3p, SLC2A3/PGAM1 could not serve as independent prognostic markers in p53-MT CRC (Fig 6F and Appendix Fig S8B). Additionally, METTL3, SLC2A1, and PGAM2 display indiscriminately prognostic values in p53-WT and p53-MT CRC

Figure 6. METTL14, miR-6769b-3p/SLC2A3, and miR-499a-3p/PGAM1 are clinically relevant in p53-WT CRC patients.

A Representative IHC and ISH staining images and corresponding quantitative analysis of miR-6769b-3p/SLC2A3 IRS and miR-499a-3p/PGAM1 IRS in p53-WT ($n = 63$) and p53-MT ($n = 41$) samples from Cohort 3. The insets show enlarged images of indicated p53-WT and p53-MT CRC tissues, respectively. Scale bars = 200 μm and 20 μm (inset). The horizontal lines represent the median; the bottom and top of the boxes represent the 25 and 75% percentiles, respectively, and the vertical bars represent the range of the data.

B Representative IHC and ISH images of METTL14, SLC2A3, PGAM1, miR-6769b-3p, and miR-499a-3p in CRC tissues with higher or lower METTL14 expression in p53-WT ($n = 63$) samples from Cohort 3. Right panels show enlarged images of indicated p53-WT CRC tissues. Scale bars = 200 μm (left) and 20 μm (right).

C Statistical analysis of METTL14, SLC2A3, PGAM1, miR-6769b-3p, and miR-499a-3p in p53-WT CRC tissues ($n = 63$) from Cohort 3.

D Achilles scores for METTL14 in p53-WT cancer cells ($n = 217$) and p53-MT cancer cells ($n = 527$).

E Association between METTL14 and p53, KRAS, ATM, TP53BP1, MDM2, PPM1D, and $p21^{WAF1/Cip1}$ was analyzed by Pearson's correlation analysis in the CCLE dataset.

F Kaplan–Meier survival curves of OS in CRC patients with wild-type p53 ($n = 63$) and mutant p53 ($n = 41$) from Cohort 3 database based on expression levels of METTL14.

G The survival analysis of OS and DFS in various tumors in TCGA database based on expression levels of METTL14. First, we have divided tumor patients diagnosed with the same tumor into patients with wild-type p53, patients with mutant p53, and patients regardless of p53 status. Second, according to optimal cutoff values, patients in three groups were divided into a high-expression METTL14 group and a low-expression METTL14 group, respectively. Finally, Survival differences between the low-expression METTL14 group and the high-expression METTL14 group in each set were assessed by the Kaplan–Meier estimate and compared using the log-rank test. Esophageal Cancer (ESCA); Bladder Cancer (BLCA); Lower Grade Glioma (LGG); Glioblastoma (GBM); Sarcoma (SARC); Skin Cutaneous Melanoma (SKCM).

H Comparison of clinicopathological characteristics between METTL14 high- and low-expression tumors in p53-WT patients from Cohort 3 ($n = 63$).

I Schematic diagram of the relationship among METTL14, glycolysis metabolism, and CRC progression.

Data information: For (A, D), statistical significance was determined by the nonparametric Mann–Whitney test. For (C), statistical significance was determined by the Fisher exact test. For E, statistical significance was determined by analysis of the Pearson's correlation coefficient. For (F, G), statistical significance was performed by the log-rank test. For (H), statistical significance was performed by the Chi-square test.

© 2023 The Authors

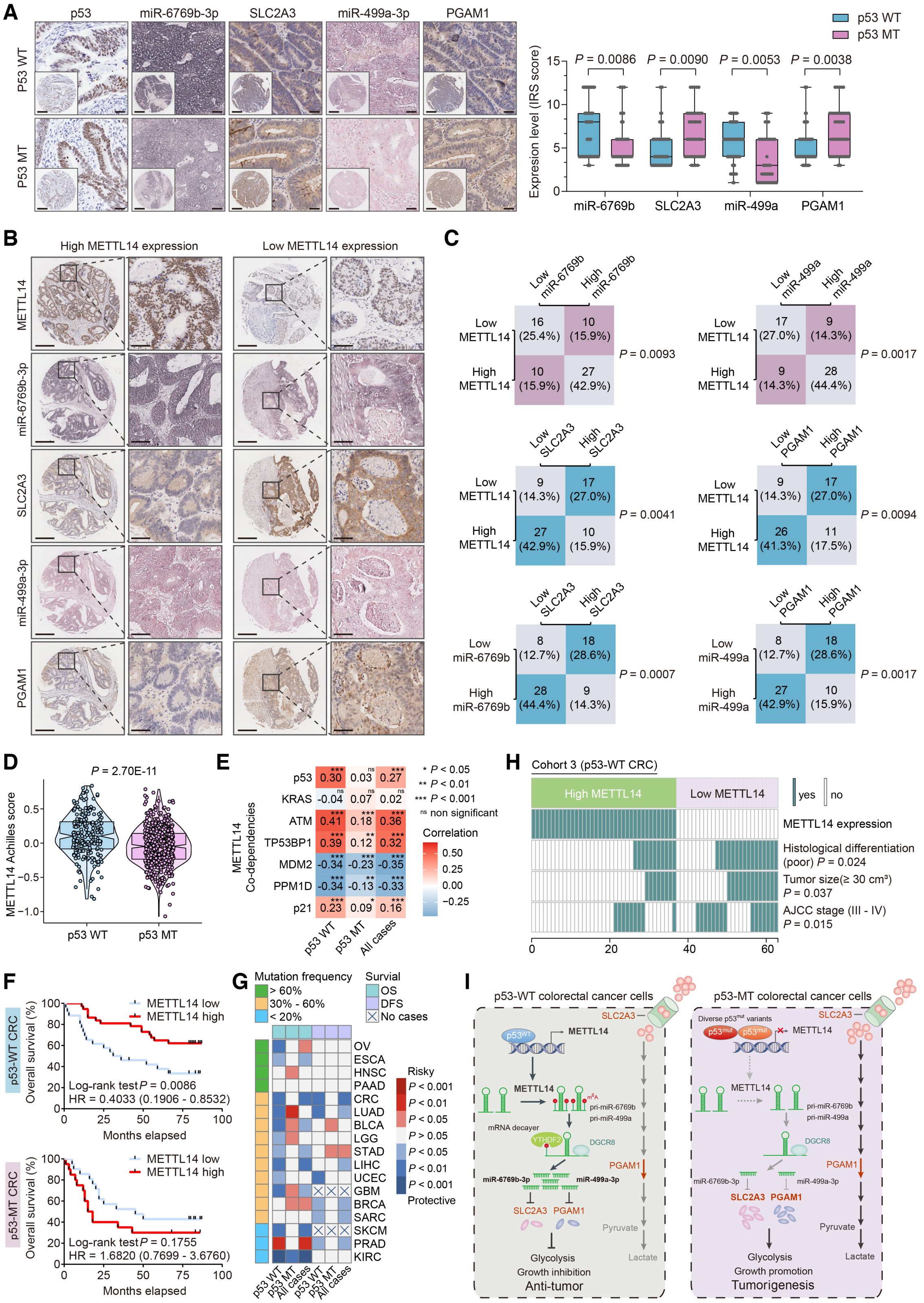
A
p53
miR-6769b-3p
SLC2A3
miR-499a-3p
PGAM1
P53 WT
P53 MT
p53 WT
p53 MT
Expression level (IRS score)
P = 0.0086
P = 0.0090
P = 0.0053
P = 0.0038
miR-6769b
SLC2A3
miR-499a
PGAM1
B
High METTL14 expression
Low METTL14 expression
METTL14
miR-6769b-3p
SLC2A3
miR-499a-3p
PGAM1
C
Low miR-6769b
High miR-6769b
Low METTL14
High METTL14
16 (25.4%)
10 (15.9%)
10 (15.9%)
27 (42.9%)
P = 0.0093
Low miR-499a
High miR-499a
17 (27.0%)
9 (14.3%)
9 (14.3%)
28 (44.4%)
P = 0.0017
Low SLC2A3
High SLC2A3
9 (14.3%)
17 (27.0%)
27 (42.9%)
10 (15.9%)
P = 0.0041
Low PGAM1
High PGAM1
9 (14.3%)
17 (27.0%)
26 (41.3%)
11 (17.5%)
P = 0.0094
Low miR-6769b
High miR-6769b
8 (12.7%)
18 (28.6%)
28 (44.4%)
9 (14.3%)
P = 0.0007
Low miR-499a
High miR-499a
8 (12.7%)
18 (28.6%)
27 (42.9%)
10 (15.9%)
P = 0.0017
D
P = 2.70E-11
METTL14 Achilles score
p53 WT
p53 MT
E
METTL14 Co-dependencies
p53 0.30 0.03 0.27
KRAS -0.04 0.07 0.02
ATM 0.41 0.18 0.36
TP53BP1 0.39 0.12 0.32
MDM2 -0.34 -0.23 -0.35
PPM1D -0.34 -0.13 -0.33
p21 0.23 0.09 0.16
p53 WT
p53 MT
All cases
* P < 0.05
** P < 0.01
*** P < 0.001
ns non significant
Correlation
H
Cohort 3 (p53-WT CRC)
High METTL14
Low METTL14
yes
no
METTL14 expression
Histological differentiation (poor) P = 0.024
Tumor size(≥ 30 cm³) P = 0.037
AJCC stage (III - IV) P = 0.015
F
p53-WT CRC
Overall survival (%)
METTL14 low
METTL14 high
Log-rank test P = 0.0086
HR = 0.4033 (0.1906 - 0.8532)
Months elapsed
p53-MT CRC
Log-rank test P = 0.1755
HR = 1.6820 (0.7699 - 3.6760)
G
Mutation frequency
> 60%
30% - 60%
< 20%
Survial
OS
DFS
No cases
OV
ESCA
HNSC
PAAD
CRC
LUAD
BLCA
LGG
STAD
LIHC
UCEC
GBM
BRCA
SARC
SKCM
PRAD
KIRC
Risky
P < 0.001
P < 0.01
P < 0.05
P > 0.05
P < 0.05
P < 0.01
P < 0.001
Protective
I
p53-WT colorectal cancer cells
p53-MT colorectal cancer cells
SLC2A3
METTL14
pri-miR-6769b
pri-miR-499a
mRNA decayer
YTHDF2
DGCR8
PGAM1
miR-6769b-3p
miR-499a-3p
SLC2A3 PGAM1
Pyruvate
Lactate
Glycolysis
Growth inhibition
Anti-tumor
Diverse p53mut variants
Growth promotion
Tumorigenesis

Figure 6.

(Appendix Fig S8A and B). We then mined data from TCGA and found that high METTL14 exhibited more favorable OS in many human cancer types with wild-type p53 regardless of spectrum of p53 mutations, whereas high METTL14 has no significant positive association with OS, or even has poorer OS in some types of tumors with medium to high frequency of p53 mutations. In addition, we also found that high METTL14 was significantly associated with a more favorable disease-free survival (DFS) in some p53-WT tumors, whereas high METTL14 did not confer significant favorable DFS or even was associated with a poorer DFS in some tumors with medium frequency of p53 (Fig 6G). Moreover, we observed that METTL14 was inversely correlated with poor histological differentiation, AJCC III/IV stage, and tumor size ($\geq$ 30 cm^3) in p53-WT CRC patients (Fig 6H), while no significant relationship was found between METTL14 and clinicopathological features of CRC patients with p53 mutations (Appendix Fig S9A). Finally, multivariate regression analysis revealed that METTL14 expression was an independent prognostic factor for p53-WT CRC patients in Cohort 3 and TCGA CRC (Appendix Fig S9B and C). In general, METTL14 is an independent predictor of beneficial prognosis in p53-WT CRC patients and functions as a tumor suppressor by selectively maturating miR-6769b-3p and miR-499a-3p, and then inhibiting the expression of SLC2A3 and PGAM1, and rewiring the cellular metabolism to reduce glycolysis and repress p53-WT CRC tumorigenesis (Fig 6I).

Discussion

The results of the present study demonstrated that METTL14 was transcriptionally activated by wild-type p53 and positively modulated the processing of pri-miR-6769b/pri-miR-499a in an m^6A-YTHDF2-dependent manner, which selectively enhanced the maturation of miR-6769b-3p/miR-499a-3p and down-regulated SLC2A3/PGAM1, respectively, thus attenuating glycolysis and tumor development in p53-WT CRC.

Selective deletion of Mettl14 in T cells impairs the induction of naïve T cells into induced Treg cells and leads to spontaneous colitis in mouse models, providing a new tool for investigating inflammatory bowel diseases (IBD) or IBD-associated malignancy (Lu *et al*, 2020). Mettl14 knockdown in tumor-associated macrophages in tumor microenvironment triggers $CD8^+$ T cells differentiating toward a dysfunctional state in mouse models, thus impairing their ability to eliminate tumors, highlighting the tumor-suppressive role of Mettl14 in tumor microenvironment (Dong *et al*, 2021). Besides, METTL14 engages in ETBF-mediated pri-miR-149 processing in HCT116 CRC cells and then miR-149-3p inhibits CRC cell proliferation through targeting PHF5A (Cao *et al*, 2021). Herein, we discovered the tumor-suppressive function of METTL14 in p53-WT CRC, which relied on the processing of pri-miR-6769b and pri-miR-499a in an m^6A-dependent manner. In addition, the biological function of m^6A depends on the selective recognition of m^6A sites by the reader protein YTHDF2 (Fu *et al*, 2014). YTHDF2 is the first validated and the most extensively studied m^6A reader, which can selectively bind to m^6A-containing mRNA and render the targeted mRNA to cellular RNA decay sites (Wang *et al*, 2014). Recent studies have revealed the engagement of m^6A reader proteins in regulating pri-miRNA stability and processing (Alarcón *et al*, 2015a; Hou *et al*, 2021), and our present study also demonstrated the importance of YTHDF2 in mediating pri-miRNA stability and processing. However, whether there exist additional stabilizers or degradosomes that could mediate similar processing effects at pri-miR-6769b/pri-miR-499a needs further investigation.

Studies have shown that mutant p53 loses the tumor-suppressive function of wild-type p53 and gains function that contributes to malignant progression; it is not surprising that a variety of therapies are being developed to target tumors expressing mutant p53. For example, compounds like PRIMA-1, maleimide analogs, and STIMA-1 have been developed to reactivate some levels of wild-type function in mutant p53. HDAC inhibitors, SIRT1, stathmin, and autophagy are reported to target mutant p53 for degradation; however, the underlying mechanisms have not been fully elucidated and their activities may not only confine to mutant p53 but also extend to degrade wild-type p53. In addition to directly targeting mutant p53, another approach is to target mutant p53-regulated pathways (Muller & Vousden, 2014). So far, these therapies have not yet been widely used and recognized. Besides, as another frequently mutant gene in CRC, mutant KRAS leads to resistance to EGFR inhibitors (EGFRIs), cetuximab and panitumumab, in metastatic CRC patients, whereas patients with wild-type KRAS respond well to EGFRIs (Martinelli *et al*, 2020). However, studies about therapeutic strategies for p53-WT CRC are limited. Additionally, we identified two glycolytic genes, SLC2A3 and PGAM1, which are crucial factors for METTL14-mediated aerobic glycolysis in p53-WT CRC. Notably, consistent with our discovery, previous studies have manifested the negative regulation of wild-type p53 on SLC2A3 expression by repressing activation of the IKK-NF-kB pathway (Kawauchi *et al*, 2008, 2009). Although researchers have developed chromopynones, glutor, glupin, and NV-5440 as small-molecule inhibitors of SLC2A3, and MJE3, EGCG, 14r, PGMI-004A, and HKB99 have been reported as potential PGAM1 inhibitors (Huang *et al*, 2019a; Reckzeh & Waldmann, 2020), these inhibitors are less specific or their applications in CRC have not been widely reported. So further studies are perspective and urgent. Strikingly, our findings suggest that the wild-type p53-modulated METTL14 can be tumor suppressive in the context of wild-type p53 yet displays no significant effects in p53-MT or p53-null CRC cells. Notably, METTL14 has been reported to be required for some cancer development, whereas METTL14 activation shows more tumor-suppressive activity in a variety of tumors including UCEC (Liu *et al*, 2018), LIHC (Ma *et al*, 2017), GBM (Cui *et al*, 2017), and skin cancer (Yang *et al*, 2021). Interestingly, Panneerdoss *et al* (2018) revealed that METTL14 knockdown in BRCA cells (MDA-MB-431, MDA-MB-468, and BT-549) carrying diverse p53 mutations led to reduced long-term viability, migration, and invasion of BRCA cells, which, together with clinical data (Fig 6G) revealing a different role for METTL14 in mutant tumors, supports the idea that METTL14 can only employ wild-type p53-dependent tumor-suppressive mechanisms. This difference in METTL14 action in the context of wild-type or deficient p53 also provides one potential explanation for observed differences in the role of METTL14 in cancer development. Our data suggest that p53 pathway status is one factor that dictates whether METTL14 is a tumor suppressor or nonfunction gene, even functions as an oncogene. Taken together, METTL14 and its related glycolytic factors are crucial for CRC tumorigenesis and development, and targeting these factors might be promising in the prevention and treatment of p53-WT CRC.

 © 2023 The Authors

In summary, biologically, METTL14 serves as a tumor suppressor and inhibits the progression of p53-WT CRC. Mechanically, METTL14 is transcriptionally activated by wild-type p53. METTL14-mediated m^6A modification selectively promotes YTHDF2-dependent processing of pri-miR-6769b and pri-miR-499a, which facilitates the maturation of corresponding miRNAs that target SLC2A3 and PGAM1, respectively, thereby inhibiting glycolysis in p53-WT CRC. Clinically, METTL14 expression inversely correlates with the prognosis of CRC, especially of p53-WT CRC. Targeting METTL14 and its associated factors may be promising for treating p53-WT CRC.

Materials and Methods

AOM and AOM/DSS-induced Villin-Cre$^+$/Mettl14$^{FL/FL}$ mouse models

In order to generate mice with a conditional deletion of METTL14 in the IECs, METTL14$^{flox/flox}$ mice were crossed with Villin-Cre transgenic mice. In the following steps, Villin-Cre-METTL14$^{flox/+}$ were crossed with Villin-Cre-METTL14$^{flox/+}$ to obtain Villin-Cre-METTL14$^{flox/flox}$ mice (named METTL14$^{\Delta IEC}$) and Villin-Cre-METTL14$^{+/+}$ (named METTL14^{WT}) littermates, which were used for the experiments. Generation of the Villin-Cre$^+$/Mettl14$^{FL/FL}$ conditional knockout mice was performed by Shanghai Model Organisms Inc. (Shanghai, China) and housed under specific pathogen-free conditions, as well as fed under a 12 h-dark/12 h-light cycle with food and water provided *ad libitum* in IVC Animal Laboratory, Shanghai Jiaotong University. For AOM-induced tumor mouse model, mice were administrated with AOM (Sigma-Aldrich; 10 mg/kg) by intraperitoneal injection for 6 consecutive weeks. For AOM/DSS-induced tumor mouse model, mice were injected intraperitoneally with AOM (Sigma-Aldrich; 10 mg/kg) on the first day. Then, these mice were fed for 7 days with 2% DSS (MP Biomedicals) in drinking water and then DSS-free water for another 14 days. This experimental series was repeated three times. Mice experiments were conducted in accordance with the National Institutes of Health Guidelines for the Care and Use of Laboratory Animals. All animal care and procedures were compliant with the Animal Ethics Committee of the Ninth People's Hospital Affiliated with Shanghai Jiaotong University School of Medicine.

Cell culture and treatment

Human p53-wild-type (p53-WT) CRC cell lines HCT116, RKO, Lovo, and other p53-mutant (p53-MT) cell lines were purchased from American Type Culture Collection (ATCC). NCM460 cell line was obtained from INCELL Corporation. Cell lines were tested to be free from mycoplasma contamination before use. All cells were cultured in the recommended medium (Gibco) supplemented with 10% fetal bovine serum (FBS; Gibco) and 1% penicillin/streptomycin (Gibco) in a humidified atmosphere of 5% CO2 at 37°C. FuGENE transfection reagent (Promega) and DharmaFECT 1 siRNA transfection reagent (Horizon Discovery) were used to transfect plasmid and small interfering RNA (siRNA) following the manufacturer's protocol, respectively. siRNAs, miRNA mimics, and inhibitors were purchased from GenePharma (Shanghai, China). p53-WT, p53R237H, and p53R175H were from Addgene and other plasmids were constructed by GeneRay (Shanghai, China) or GenePharma (Shanghai, China). Pifithrin-alpha (PFTα) and doxorubicin (Dox) were obtained from Sigma-Aldrich. Sequences of siRNA, miRNA mimics, and inhibitors were listed in Appendix Table S1. For transfection with plasmids, cells were seeded at 50% confluence 24 h before transfection, then transfected using FuGENE HD according to the manufacturer's instructions. Briefly, the transfection complex was made with 1 μg plasmid, 3 μl FuGENE HD and 100 μl Opti-MEM. Six hours after the complex was added to the cells, normal culture media were used to culture cells. After additional 48 h, cells were harvested for analyses. For transfection with siRNA, miRNA mimics or inhibitors, siRNA, miRNA mimics or inhibitors, and DharmaFECT 1 reagent were diluted in 100 μl Opti-MEM, respectively, and the contents were mixed gently. The mixtures were incubated for 10–20 min at room temperature. The incubated mixtures of siRNA and DharmaFECT 1 were then added to cells. After additional 48 h, cells were harvested for analyses.

To obtain stably transfected cell lines, cDNA was amplified and subcloned into the lentiviral vector. HEK-293T cells were then transfected with lentiviral plasmids and supernatants were collected for infecting CRC cell lines with 8 μg/ml polybrene. Stable cells were selected by treatment with puromycin, and the overexpression or knockdown efficiency was assessed by qRT–qPCR. The lentivirus control and lentiviral vectors expressing METTL14 were constructed by Shanghai OBiO medical biotechnology company, Shanghai, China.

Quantitative RT–PCR analysis (qRT–PCR)

Total RNA was isolated from the colorectal tissue specimens and cell lines by TRIzol reagent (Ambion) according to the manufacturer's instructions. Deoxyribonuclease I (Sigma-Aldrich) and phenol-chloroform were used to purify RNA. For analysis of pri-miRNA and mRNA, PrimeScript RT Reagent Kit (TAKARA) was used for reverse transcription into cDNA. Pre-miRNA and mature miRNA were extracted from total RNA using RNeasy MinElute Cleanup Kit (Qiagen) and their cDNAs were synthesized using miScript II RT Kit (Qiagen). Quantitative real-time PCR was carried out in triplicate with StepOnePlus real-time PCR system (Applied Biosystems). qPCR primers used in this study were listed in Appendix Table S2. ACTB and U6 small nuclear RNAs were used as internal controls. $2^{-\Delta\Delta Ct}$ method was used to calculate the relative expression of RNAs.

Patient specimens

We used four Cohorts of patients with CRC who received surgery between 2014 and 2020. Cohort 1 contained 118 paired CRC and para-cancerous samples. Cohort 2 and Cohort 3 with detailed clinicopathological characteristics and follow-up data were derived from 90 and 104 CRC patients, respectively. CRC was diagnosed by the histopathological examination. Tissue samples for RNA extraction were stored in liquid nitrogen, and those for IHC staining were embedded in paraffin. All the individuals received no preoperative chemotherapy or radiotherapy. The written informed consent form was obtained from each participant and this study was approved by the Ethics Committee of the Ninth People's Hospital Affiliated with

© 2023 The Authors

Shanghai Jiaotong University School of Medicine. The procedure was in accordance with the provisions of the Declaration of Helsinki of 1975. The clinical information of Cohort 3 was listed in Dataset EV3.

Western blot

Protein was extracted from CRC cells, specimens, or immunoprecipitation samples by RIPA lysis buffer (Beyotime Biotechnology) and incubated on ice for 1 h. Then, the supernatant was collected after centrifugation at 4°C for 20 min at 21,130 *g* and was quantified by BCA Assay Kit (Thermo Fisher Scientific). Total protein (40 μg) was separated by 10% SDS-PAGE and transferred onto PVDF membranes (Bio-Rad Laboratories). After blocking with 5% BSA for 2 h, membranes were incubated with primary antibodies at 4°C overnight. Antibodies against METTL14 (ab220030), DGCR8 (ab191875), Ki-67 (ab15580), MPC1 (ab74871), SLC2A3 (ab15311), and HK2 (ab227198) were purchased from Abcam. Antibodies against p53 (sc-126), DGCR8 (sc-377249) SLC2A1 (sc-377228), ELK1 (sc-365876), PFKP (sc-514824), SLC2A3 (sc-74399), GPI (sc-271459), FBP1 (sc-271241), ALDOA (sc-390733), ALDOC (sc-271593), GAPDHS (sc-293335), PGK1 (sc-130335), PGAM1 (sc-130334), ENO3 (sc-100811) and LDHA (sc-137243) were purchased from Santa Cruz Biotechnology. Antibodies against p21 (#2947) were purchased from Cell Signaling Technology. Antibodies against m^{6}A (Cat. No. 202003 and Cat. No. 202011) were purchased from Synaptic System. Antibody against METTL14 (A8530) was purchased from ABclonal. Antibody against ACTB was purchased from Kangcheng. Antibody against METTL14 (HPA038002) was purchased from Sigma-Aldrich. The next day, membranes were washed with TBS-T and incubated with Secondary antibodies (Kangcheng) labeled with HRP. Blots were imaged using the ECL detection system (Bio-Rad Laboratories).

In vitro cell viability and colony formation assays

Cell viability was assessed by Cell Counting Kit-8 (CCK8, Dojindo). Control and stably transfected CRC cells were seeded in 96-well plates at a density of 3,000 cells/well and incubated overnight. Then, cells were cultured for four consecutive days. CCK8 reagent was added to cells at a fixed time every day, and the volume ratio of CCK8 reagent to the serum-free medium was 1:10 (CCK8 reagent 10 μl/well). After adding CCK8 reagent, cells were incubated in the dark at 37°C for 2 h and the absorbance of each well was measured by OD at a wavelength of 450 nm. For colony formation assay, CRC cells were seeded into 6-well culture plates and cultured for 7–10 days. Subsequently, cells were fixed with 4% paraformaldehyde and stained with 0.1% crystal violet. After washing several times with PBS and drying, cell colonies were counted and cell clusters with more than 50 cells were counted as colonies.

Immunohistochemistry (IHC)

Colorectal cancer tissue sections were deparaffinized and rehydrated through an alcohol series followed by heat-mediated antigen retrieval carried out by microwaving with sodium citrate buffer. After blocking endogenous activity with 3% hydrogen peroxide and 5% normal goat serum, slides were incubated with appropriate antibodies in a humidified box at 4°C overnight. Antibodies against METTL14 (HPA038002) and PGAM1 (sc-130334) were purchased from Abcam. Antibody against p53 (sc-126) was purchased from Santa Cruz Biotechnology. PGAM2 (15550-1-AP), SLC2A1 (21829-1-AP), SLC2A3 (20403-1-AP) and METTL3 (15073-1-AP) were purchased from Proteintech. Color reaction was performed with horseradish peroxidase (HRP) conjugates with DAB detection, and hematoxylin was used to counterstain the cell nuclei. The IHC staining was scored by 2 independent investigators who were blinded to the clinical characteristics. The immunostaining degree was calculated according to the staining intensity and the percentage of positive cells. The staining intensity was scored from 0 to 3 (0, negative staining; 1, weak staining; 2, moderate staining; 3, strong staining) and the percentage of positive cells was also scored from 0 to 3: 0 (< 5%, negative), 1 (5–25%, sporadic), 2 (25–50%, focal), 3 (50–75%, diffuse) and 4 (> 75%, entire). The final immunoreactive score (IRS) ranged from 0 to 12, and the final IRS was classified as follows: negative expression (0), low expression (1–4), medium expression (6–8), and high expression (9–12). Besides, protein expression within xenografts or intestinal epithelial from mice was assessed on the basis of the intensity and the scale of staining using Image-Pro Plus 6.0 (Media Cybernetics, Rockville, MD, USA) (Lin *et al*, 2020).

Immunofluorescence (IF)

Cells were plated on coverslips, fixed with 4% formaldehyde, and permeabilized in 0.1% Triton X-100 in PBS. Then, cells were blocked with 1 mg/ml BSA in PBS and incubated with specific primary antibodies at 4°C overnight. Antibody against METTL14 (HPA038002) was purchased from Sigma-Aldrich. Antibody against p53 (sc-126) was purchased from Santa Cruz Biotechnology. Antibodies against SLC2A3 (sc-74399) and PGAM1 (sc-130334) were purchased from Santa Cruz Biotechnology. Next day, cells were washed and incubated accordingly with dye-conjugated secondary antibodies (Thermo Fisher Scientific). Finally, cells were counterstained with 4,6-diamidino-2-phenylindole (DAPI, Vector Laboratories). Images were captured using a Zeiss LSM-710 confocal microscope (Zeiss). The relative mean fluorescence density was analyzed by ImageJ (Shao *et al*, 2022).

In situ hybridization (ISH)

Samples were deparaffinized and treated with 10% hydrogen peroxide and pepsin (EXIQON), and hybridized with DIG-labeled LNA probes (EXIQON) complementary to miR-6769b-3p or miR-499a-3p. After being blocked with 10% normal goat serum and 5% BSA in PBS, specimens were incubated with anti-DIG Fab fragments (Roche) and secondary antibody conjugated with alkaline phosphatase (Invitrogen) using nitroblue tetrazolium/5-bromo-4-chloro-3-indolyl phosphate (NBT-BCIP) as substrate. Counterstaining was conducted using a nuclear fast red solution (Vector Laboratories). Nikon 80i microscope and Nikon NIS-Elements F 2.3 software (Nikon) were used for analysis. microRNAs expression was assessed on the basis of the intensity and the scale of staining using Image-Pro Plus 6.0 (Media Cybernetics, Rockville, MD, USA).

m^{6}A dot blot

Dot blot was conducted essentially as described for m^{6}A immunoblotting (above). The poly(A)+ RNA was loaded to the Hybond-N+

© 2023 The Authors

membrane (GE Healthcare) and UV crosslinking was performed. Subsequently, the membrane was blocked with 5% nonfat milk in PBST and incubated with anti-m^{6}A antibody (A8530, ABclonal) at 4°C overnight. Next day, the membrane was incubated with horseradish peroxidase-conjugated secondary antibody (Kangcheng) and detected with the ECL detection system (Biorad). The same amount of poly(A)+ RNA was loaded to the membrane, stained with 0.02% methylene blue (MB) in 0.3 M mol/l sodium acetate (pH = 5.2) as control.

Methylated RNA immunoprecipitation (MeRIP) assay

m^{6}A methylation levels of RNA were determined using Magna MeRIPTM m^{6}A Kit (Merck Millipore). Total RNA was isolated by TRIzol reagent (Ambion) and treated with deoxyribonuclease I (Sigma-Aldrich). RNA fragmentation reagents (NEB) were used to fragment RNA and the fragmented RNA was immunoprecipitated with anti-m^{6}A antibody (Synaptic Systems) according to the instructions from the manufacturer. 1/10 of the fragmented RNA was saved as input. Real-time PCR was performed after m^{6}A-IP to quantify the changes in m^{6}A methylation of RNA.

Glucose uptake assay

Glucose uptake assay was performed using Glucose Uptake Colorimetric Assay Kit (Abcam, ab136955) according to the manufacturer's instructions. For measurement of glucose uptake, cells were seeded into 10-cm plates, transfected or infected with indicated constructs, and incubated in RPMI 1640 supplemented with 10% FBS for 48 h. The transfected cells were harvested, and the cell number was determined. Treated cells were seeded to 96-well plates at a density of 1×10^4 cells/well and incubated at 37°C overnight, at which time the cell numbers were fairly similar across groups. Next day, cells were starved for glucose for 2 h and then incubated in 100 µl Krebs-Ringer-Phosphate-HEPES for 40 min. Subsequently, 10 µl 10 mM 2-deoxyglucose (2-DG) was added into each well and incubated for 20 min. Finally, cells were collected by extraction buffer and the glucose uptake was measured by OD at a wavelength of 412 nm. The results were normalized to cell numbers.

Lactate production assay

Lactate production assay was performed using a colorimetric L-Lactate Assay kit (Abcam, ab65331) according to the manufacturer's instructions. Cells were transfected or infected and harvested as described in glucose uptake assay. Culture media were removed, and cells were incubated in RPMI 1640 without FBS. After incubation for 1 h, supernatants were collected for measurement of lactate production. The reaction mixture was incubated for 30 min at room temperature and protected from light. The lactate level in the supernatants was measured at 450 nm wavelength and normalized with cell numbers. For measurement of the lactate levels of intestinal epithelial cells (IECs) isolated from AOM/DSS-induced Mettl14$^{\Delta IEC}$ and Mettl14^{WT} mice CRC models, 1×10^5 IECs were isolated and homogenized in the Lactate Assay Buffer (Abcam). After centrifugation, the soluble fractions were used for measurements, which were normalized to cell numbers.

ATP production assay

Cellular ATP production was measured using ATP Assay Kit (Abcam, ab83355) according to the manufacturer's instructions. Cells were transfected or infected as described above, and treated cells were seeded to 6-well plates and cultured at 37°C overnight, at which time the cell numbers were quite similar across groups. 5×10^6 cells from each group were homogenized in ATP Assay Buffer and cell lysates were mixed and incubated with ATP Reaction Mix for 30 min. Absorbance of the mixtures was measured at a 570 nm wavelength. Values were normalized to cell numbers. For measurement of the ATP levels of IECs isolated from AOM/DSS-induced Mettl14$^{\Delta IEC}$ and Mettl14^{WT} mice CRC models, 5×10^6 IECs were isolated and homogenized in the ATP Assay Buffer (Abcam). After centrifugation, the soluble fractions were used for measurements, which were normalized to cell numbers.

Pyruvate assay

The pyruvate level was measured using a colorimetric Pyruvate Assay Kit (BioVision, K609-100) according to the instructions from the manufacturer. Cells were transfected or infected as described above, and 5×10^5 cells from each group were centrifuged to remove insoluble material and the supernatants were collected. Then, 2–50 µl sample was added to 96-well plate, and the volume was adjusted to 50 µl/well with Pyruvate Assay Buffer. Subsequently, 50 µl Reaction Mix was added to each well and incubated for 30 min at room temperature. Pyruvate was measured at 570 nm wavelength and normalized with cell numbers. For measurement of the pyruvate levels of IECs isolated from AOM/DSS-induced Mettl14$^{\Delta IEC}$ and Mettl14^{WT} mice CRC models, 5×10^5 IECs were isolated and homogenized in the Pyruvate Assay Buffer (BioVision). After centrifugation, the soluble fractions were used for measurements, which were normalized to cell numbers.

PGAM enzyme activity assay

The PGAM enzyme activity was measured using a colorimetric pyruvate assay kit (BioVision, K2007-100) according to the instructions from the manufacturer. For measurement of the PGAM enzyme activity of IECs isolated from AOM/DSS-induced Mettl14$^{\Delta IEC}$ and Mettl14^{WT} mice CRC models, 1×10^6 IECs were isolated and homogenized in the Pyruvate Assay Buffer (BioVision). The samples were centrifuged, and the soluble fractions were used for measurements at a 570 nm wavelength, and the results were normalized to cell numbers.

Seahorse metabolic analysis

Extracellular acidification rate (ECAR) and oxygen consumption rate (OCR) were measured using Seahorse XF Glycolysis Stress Test Kit and Seahorse XF Cell Mito Stress Test Kit (Agilent Technologies). Treated CRC cells were seeded into the Seahorse plates and incubated overnight at 37°C, at which time the cell numbers were quite similar across groups. Next day, the monolayer cells were washed, and baseline concentration was measured. Then, glucose, oligomycin, and 2-DG were sequentially added for ECAR measurement, and oligomycin, FCCP (p-trifluoromethoxy carbonyl cyanide

© 2023 The Authors

phenylhydrazone), and antimycin A and rotenone were sequentially added for OCR measurement at specific time points. Data were analyzed by Seahorse XF-96 Wave software. OCR was reported in pmol/min and ECAR in mpH/min. The results were normalized to cell numbers.

RNA immunoprecipitation (RIP) assay

RNA immunoprecipitation was conducted using the Magna RIP™ RNA Kit (Merck Millipore) according to the manufacturer's protocol. Briefly, magnetic beads coated with specific antibodies or normal IgG (Thermo Fisher Scientific) were incubated with prepared cell lysates at 4°C overnight. Washed protein-RNA complexes were mixed with proteinase K digestion buffer. Then, the co-precipitated RNAs were purified using phenol: chloroform: isoamyl alcohol and subsequently subjected to qPCR to investigate the enrichment of RNA to specific proteins or subjected to m^6A immunoblotting as described above.

Co-immunoprecipitation (Co-IP)

Treated cells were lysed with IP lysis buffer (Beyotime Biotechnology) containing Protease/Phosphatase Inhibitor Cocktail (Beyotime Biotechnology). Cell lysates were centrifuged, and the supernatants were collected and incubated with specific antibodies or normal IgG overnight at 4°C. Next day, Protein A/G Beads (Santa Cruz Biotechnology) were added into the supernatants and incubated for 3 h at 4°C. After washing protein-binding beads, the co-precipitated proteins were subjected to western blot assays as described above.

Chromatin immunoprecipitation (ChIP) assays

Chromatin immunoprecipitation assay was performed using the EZ-Magna ChIPTM A/G Kit (Millipore) according to the manufacturer's protocol. Briefly, cells were treated with 1% formaldehyde, harvested in lysis buffer, and sonicated to generate DNA fragments. Pre-purified DNA of each sample was saved as input control. Then, DNA fragments were used for immunoprecipitation with p53 antibody or normal IgG (Cell Signaling Technology). Afterward, DNA was eluted and purified, and the purified chromatin DNA samples were used to detect the promoter of METTL14. The primer sequences used in ChIP assay were described in Appendix Table S3.

Luciferase assay

The luciferase assay was conducted using the Dual Luciferase Reporter Assay Kit (Promega) according to the manufacturer's protocol. Briefly, CRC cells were plated in 96-well plates and co-transfected with blank PGL3, PGAM1 3′-UTR-WT, PGAM1 3′-UTR-Mut, SLC2A3 3′-UTR-WT, SLC2A3 3′-UTR-Mut, METTL14 p53-BR, or METTL14 p53-BR mut, and control mimics, miR-6769b-3p mimics, miR-499a-3p mimics, vector, p53-WT, or MT plasmids. After 48 h of transfection, cells were lysed, and the firefly and Renilla luciferase activities were measured. Relative luciferase activity was shown as firefly luciferase activity normalized to Renilla luciferase activity.

RNA stability assay

Transfected CRC cells were treated with Actinomycin D (Sigma-Aldrich) at a final concentration of 5 μg/ml for the indicated time. Total RNA was extracted and quantified by RT–qPCR. The degradation rate of RNA (K_{decay}) was calculated by the following equation:

$$\ln\left(\frac{C}{C0}\right) = -K_{decay}t$$

t is the transcription inhibition time, and C is the RNA level at the time t. C0 is the level of RNA at 0 h in the equation, which means the mRNA level before decay starts. Thus, the RNA half-time ($t_{1/2}$) can be calculated by the equation:

$$\ln\left(\frac{1}{2}\right) = -K_{decay}t_{1/2}$$

CRC xenograft model

BALB/c nude mice were purchased from Charles River Laboratories (Beijing, China) and housed under specific pathogen-free conditions, and fed under a 12 h-dark/12 h-light cycle with food and water provided *ad libitum* in IVC Animal Laboratory, Shanghai Jiaotong University.

Colorectal cancer xenograft model was established by injecting cells into the right flank of 4-week-old male BALB/c nude mice subcutaneously. After the tumors were palpable, tumor growth was measured every 4 days and tumor volume was calculated as length × width^2 × 0.5. 2-DG (Selleck) or 3-Bromopyruvate (3-BP, Selleck) was administered, respectively, at 800 and 5 mg/kg body weight by intraperitoneal injection once every other day. Mice were sacrificed after 3 weeks, and the subcutaneous tumors were excised and weighed. Mice experiments were conducted in accordance with the National Institutes of Health Guidelines for the Care and Use of Laboratory Animals. All animal care and procedures were compliant with the Ethics Committee of Shanghai Jiao Tong University.

Isolation of intestinal epithelial cells

Intestinal epithelial cells were extracted as described previously, with some modifications (Weigmann *et al*, 2007). Briefly, colonic tissues were cleared and cut into 1–2 cm pieces. The tissue fragments were then incubated with 2.5 mM ethylenediaminetetraacetic acid (EDTA) and 1 mM Dithiothreitol (DTT) in HBSS for 20 min under slow rotation. Mixtures were then filtered by a cell strainer and intestinal epithelial cells were collected by centrifugation.

High-throughput sequencing

For RNA sequencing, total RNA was isolated from CRC cells with or without METTL14 knockdown. The RNA sequencing was performed by OE Biotech Co, Ltd (Shanghai, China) using Illumina Hiseq X Ten platform to generate subsequent raw data. Each group had four biological replicates. The analysis of RNA-seq data was performed according to the TopHat-HTSeq-DeSeq2 frame. Briefly, reads were mapped to the human genome (hg19) using TopHat v2.0.11 (http://tophat.cbcb.umd.edu) with the default options with a TopHat

© 2023 The Authors

transcript index built from Ensembl_GRCh37. Count files of the aligned sequencing reads were generated by the htseq-count script from the Python package HTSeq with union mode, using the GTF annotation file. The read counts from each sequenced sample were combined into a count file, which was subsequently used for the differential expression analysis. Differential analyses were performed on the count files using DESeq2 packages, following standard normalization procedures. For RNA-seq data, we explored the underlying glycolysis-related genes based on *P*-value and adj.*P*.Val (Dataset EV1).

The Cancer Genome Atlas CRC consisted of primary colorectal adenocarcinoma with wild-type p53 and mutant p53. To gain further insight into the biological pathways involved in diverse p53 status CRC occurrence and development through METTL14, the gene set enrichment analysis (GSEA) was performed. The gene sets collection (c2.all.v4.0.symbols.gmt) from the Molecular Signatures Database–MsigDB (http://www.broad.mit.edu/gsea/msigdb/index.jsp) was used for the enrichment analysis.

For miRNA microarrays, gene expression analyses of CRC cells with or without METTL14 overexpression were performed using Affymetrix miRNA 4.0 by OE Biotech Co, Ltd (Shanghai, China) and Sinotech Genomics Co, Ltd (Shanghai, China), respectively. Each group was sequenced in triplicate. Differential analyses were performed on the count files using the Student's *t*-test, following standard normalization procedures. For miRNA sequencing, we explored the underlying miRNAs based on *P*-value (Dataset EV2). Kyoto Encyclopedia of Genes and Genomes (KEGG) enrichment analysis of differential expression miRNA target genes was conducted by miRWalk (http://mirwalk.umm.uni-heidelberg.de/) and miRDB (http://mirdb.org/) database.

Statistical analysis

SPSS 24.0 (IBM Corporation) and GraphPad Prism 8.0 (GraphPad Software) were used to perform the statistical analysis. At least three independent experiments were performed and all of the data were shown as mean ± standard deviation (SD). Normally distributed measurement data with comparable variation were analyzed by the Student's *t*-test between two groups or analyzed by the one-way analysis of variance (ANOVA) test among three or more groups. If the data exhibited a significant difference, when the data presented a skewed distribution, comparisons between two groups were performed using the nonparametric Mann–Whitney U-test or Wilcoxon's rank-sum test. Spearman correlation analysis was performed to assess the relationship between different factors. Clinicopathological data were analyzed by the Chi-square test or Fisher exact test. The Kaplan–Meier method was used to assess survivals and curves were compared with the log-rank test. A Cox proportional hazards model was applied to identify independently significant variables. $P < 0.05$ was considered statistically significant.

Data availability

Our high-throughput sequencing data can be downloaded from NCBI GEO database under the accession numbers GSE186792 (http://www.ncbi.nlm.nih.gov/geo/query/acc.cgi?acc=GSE186792) and GSE210056 (http://www.ncbi.nlm.nih.gov/geo/query/acc.cgi?acc=GSE210056). Other sequencing data can be downloaded from GEO database under the accession number GSE39582 (http://www.ncbi.nlm.nih.gov/geo/query/acc.cgi?acc=GSE39582).

Expanded View for this article is available online.

Acknowledgements

We are grateful to Liang-Nian Song for the critical reading of the paper. This work was supported by the National Natural Science Foundation of China (Grant nos. 81874419, 82203245, 84003167), the Interdisciplinary Program of Shanghai Jiao Tong University (Grant nos. ZH4018QNA01), the Shanghai Sailing Program (Grant nos. 22YF1423100, 19YF1446900, and 44YF1443100), and the Shanghai Pujiang Program (Grant nos. 20PJ1409700).

Author contributions

Yichao Hou: Conceptualization; resources; data curation; software; formal analysis; validation; investigation; visualization; methodology; writing – original draft. **Xintian Zhang:** Data curation; formal analysis; investigation; writing – original draft. **Han Yao:** Data curation; formal analysis; investigation; writing – original draft. **Lidan Hou:** Data curation; formal analysis; investigation. **Qingwei Zhang:** Data curation; formal analysis; investigation. **Enwei Tao:** Data curation; formal analysis; investigation. **Xiaoqiang Zhu:** Data curation; formal analysis; investigation. **Shanshan Jiang:** Data curation; formal analysis; investigation. **Yimeng Ren:** Data curation; formal analysis; investigation. **Xialu Hong:** Data curation; formal analysis; investigation. **Shiyuan Lu:** Data curation; formal analysis; investigation. **Xiaoxu Leng:** Data curation; formal analysis; investigation. **Yile Xie:** Data curation; formal analysis; investigation. **Yaqi Gao:** Data curation; formal analysis; investigation. **Yu Liang:** Data curation; investigation. **Ting Zhong:** Data curation; formal analysis; investigation. **Bohan Long:** Data curation; formal analysis; investigation. **Jing-Yuan Fang:** Conceptualization; resources; supervision; project administration; writing – review and editing. **Xiangjun Meng:** Conceptualization; resources; supervision; funding acquisition; investigation; methodology; project administration; writing – review and editing.

Disclosure and competing interests statement

The authors declare that they have no conflict of interest.

References

Alarcón CR, Goodarzi H, Lee H, Liu X, Tavazoie S, Tavazoie SF (2015a) HNRNPA2B1 is a mediator of m(6)A-dependent nuclear RNA processing events. *Cell* 162: 1299–1308

Alarcón CR, Lee H, Goodarzi H, Halberg N, Tavazoie SF (2015b) N6-methyladenosine marks primary microRNAs for processing. *Nature* 519: 482–485

An Y, Duan H (2022) The role of m6A RNA methylation in cancer metabolism. *Mol Cancer* 21: 14

Apicella M, Giannoni E, Fiore S, Ferrari KJ, Fernández-Pérez D, Isella C, Granchi C, Minutolo F, Sottile A, Comoglio PM *et al* (2018) Increased lactate secretion by cancer cells sustains non-cell-autonomous adaptive resistance to MET and EGFR targeted therapies. *Cell Metab* 28: 848–865

Baker SJ, Fearon ER, Nigro JM, Hamilton SR, Preisinger AC, Jessup JM, vanTuinen P, Ledbetter DH, Barker DF, Nakamura Y *et al* (1989) Chromosome 17 deletions and p53 gene mutations in colorectal carcinomas. *Science* 244: 217–221

© 2023 The Authors

Bensaad K, Tsuruta A, Selak MA, Vidal MN, Nakano K, Bartrons R, Gottlieb E, Vousden KH (2006) TIGAR, a p53-inducible regulator of glycolysis and apoptosis. *Cell* 126: 107–120

Bieging-Rolett KT, Kaiser AM, Morgens DW, Boutelle AM, Seoane JA, Van Nostrand EL, Zhu C, Houlihan SL, Mello SS, Yee BA *et al* (2020) Zmat3 is a key splicing regulator in the p53 tumor suppression program. *Mol Cell* 80: 452–469

Cao Y, Wang Z, Yan Y, Ji L, He J, Xuan B, Shen C, Ma Y, Jiang S, Ma D *et al* (2021) Enterotoxigenic bacteroidesfragilis promotes intestinal inflammation and malignancy by inhibiting exosome-packaged miR-149-3p. *Gastroenterology* 161: 1552–1566

Chen M, Wei L, Law CT, Tsang FH, Shen J, Cheng CL, Tsang LH, Ho DW, Chiu DK, Lee JM *et al* (2018) RNA N6-methyladenosine methyltransferase-like 3 promotes liver cancer progression through YTHDF2-dependent posttranscriptional silencing of SOCS2. *Hepatology* 67: 2254–2270

Cui Q, Shi H, Ye P, Li L, Qu Q, Sun G, Sun G, Lu Z, Huang Y, Yang CG *et al* (2017) m(6)A RNA methylation regulates the self-renewal and tumorigenesis of glioblastoma stem cells. *Cell Rep* 18: 2622–2634

De Rosa V, Iommelli F, Monti M, Fonti R, Votta G, Stoppelli MP, Del Vecchio S (2015) Reversal of Warburg effect and reactivation of oxidative phosphorylation by differential inhibition of EGFR signaling pathways in non-small cell lung cancer. *Clin Cancer Res* 21: 5110–5120

Dong L, Chen C, Zhang Y, Guo P, Wang Z, Li J, Liu Y, Liu J, Chang R, Li Y *et al* (2021) The loss of RNA N(6)-adenosine methyltransferase Mettl14 in tumor-associated macrophages promotes $CD8^+$ T cell dysfunction and tumor growth. *Cancer Cell* 39: 945–957

Fresquet V, Garcia-Barchino MJ, Larrayoz M, Celay J, Vicente C, Fernandez-Galilea M, Larrayoz MJ, Calasanz MJ, Panizo C, Junza A *et al* (2021) Endogenous retroelement activation by epigenetic therapy reverses the Warburg effect and elicits mitochondrial-mediated cancer cell death. *Cancer Discov* 11: 1268–1285

Fu Y, Dominissini D, Rechavi G, He C (2014) Gene expression regulation mediated through reversible m^6A RNA methylation. *Nat Rev Genet* 15: 293–306

Hanahan D, Weinberg RA (2011) Hallmarks of cancer: the next generation. *Cell* 144: 646–674

Hou Y, Zhang Q, Pang W, Hou L, Liang Y, Han X, Luo X, Wang P, Zhang X, Li L *et al* (2021) YTHDC1-mediated augmentation of miR-30d in repressing pancreatic tumorigenesis via attenuation of RUNX1-induced transcriptional activation of Warburg effect. *Cell Death Differ* 28: 3105–3124

Huang K, Liang Q, Zhou Y, Jiang LL, Gu WM, Luo MY, Tang YB, Wang Y, Lu W, Huang M *et al* (2019a) A novel allosteric inhibitor of phosphoglycerate mutase 1 suppresses growth and metastasis of non-small-cell lung cancer. *Cell Metab* 30: 1107–1119

Huang Y, Su R, Sheng Y, Dong L, Dong Z, Xu H, Ni T, Zhang ZS, Zhang T, Li C *et al* (2019b) Small-molecule targeting of oncogenic FTO demethylase in acute myeloid leukemia. *Cancer Cell* 35: 677–691

Kawauchi K, Araki K, Tobiume K, Tanaka N (2008) p53 regulates glucose metabolism through an IKK-NF-kappaB pathway and inhibits cell transformation. *Nat Cell Biol* 10: 611–618

Kawauchi K, Araki K, Tobiume K, Tanaka N (2009) Loss of p53 enhances catalytic activity of IKKbeta through O-linked beta-N-acetyl glucosamine modification. *Proc Natl Acad Sci USA* 106: 3431–3436

Koppenol WH, Bounds PL, Dang CV (2011) Otto Warburg's contributions to current concepts of cancer metabolism. *Nat Rev Cancer* 11: 325–337

Levine AJ, Oren M (2009) The first 30 years of p53: growing ever more complex. *Nat Rev Cancer* 9: 749–758

Levine AJ, Puzio-Kuter AM (2010) The control of the metabolic switch in cancers by oncogenes and tumor suppressor genes. *Science* 330: 1340–1344

Li Z, Weng H, Su R, Weng X, Zuo Z, Li C, Huang H, Nachtergaele S, Dong L, Hu C *et al* (2017) FTO plays an oncogenic role in acute myeloid leukemia as a N(6)-methyladenosine RNA demethylase. *Cancer Cell* 31: 127–141

Li Z, Peng Y, Li J, Chen Z, Chen F, Tu J, Lin S, Wang H (2020) N(6)-methyladenosine regulates glycolysis of cancer cells through PDK4. *Nat Commun* 11: 2578

Lin Z, Niu Y, Wan A, Chen D, Liang H, Chen X, Sun L, Zhan S, Chen L, Cheng C *et al* (2020) RNA m(6)A methylation regulates sorafenib resistance in liver cancer through FOXO3-mediated autophagy. *EMBO J* 39: e103181

Liu J, Eckert MA, Harada BT, Liu SM, Lu Z, Yu K, Tienda SM, Chryplewicz A, Zhu AC, Yang Y *et al* (2018) m(6)A mRNA methylation regulates AKT activity to promote the proliferation and tumorigenicity of endometrial cancer. *Nat Cell Biol* 20: 1074–1083

Lu TX, Zheng Z, Zhang L, Sun HL, Bissonnette M, Huang H, He C (2020) A new model of spontaneous colitis in mice induced by deletion of an RNA m(6)A methyltransferase component METTL14 in T cells. *Cell Mol Gastroenterol Hepatol* 10: 747–761

Ma JZ, Yang F, Zhou CC, Liu F, Yuan JH, Wang F, Wang TT, Xu QG, Zhou WP, Sun SH (2017) METTL14 suppresses the metastatic potential of hepatocellular carcinoma by modulating N(6) -methyladenosine-dependent primary MicroRNA processing. *Hepatology* 65: 529–543

Martinelli E, Ciardiello D, Martini G, Troiani T, Cardone C, Vitiello PP, Normanno N, Rachiglio AM, Maiello E, Latiano T *et al* (2020) Implementing anti-epidermal growth factor receptor (EGFR) therapy in metastatic colorectal cancer: challenges and future perspectives. *Ann Oncol* 31: 30–40

Mathelier A, Fornes O, Arenillas DJ, Chen CY, Denay G, Lee J, Shi W, Shyr C, Tan G, Worsley-Hunt R *et al* (2016) JASPAR 2016: a major expansion and update of the open-access database of transcription factor binding profiles. *Nucleic Acids Res* 44: D110–D115

Meyers RM, Bryan JG, McFarland JM, Weir BA, Sizemore AE, Xu H, Dharia NV, Montgomery PG, Cowley GS, Pantel S *et al* (2017) Computational correction of copy number effect improves specificity of CRISPR-Cas9 essentiality screens in cancer cells. *Nat Genet* 49: 1779–1784

Muller PA, Vousden KH (2013) p53 mutations in cancer. *Nat Cell Biol* 15: 2–8

Muller PA, Vousden KH (2014) Mutant p53 in cancer: new functions and therapeutic opportunities. *Cancer Cell* 25: 304–317

Pajak B, Siwiak E, Sołtyka M, Priebe A, Zieliński R, Fokt I, Ziemniak M, Jaćkiewicz A, Borowski R, Domoradzki T *et al* (2019) 2-Deoxy-d-glucose and its analogs: from diagnostic to therapeutic agents. *Int J Mol Sci* 21: 234

Panneerdoss S, Eedunuri VK, Yadav P, Timilsina S, Rajamanickam S, Viswanadhapalli S, Abdelfattah N, Onyeagucha BC, Cui X, Lai Z *et al* (2018) Cross-talk among writers, readers, and erasers of m(6)A regulates cancer growth and progression. *Sci Adv* 4: eaar8263

Parang B, Barrett CW, Williams CS (2016) AOM/DSS model of colitis-associated cancer. *Methods Mol Biol* 1422: 297–307

Pavlova NN, Thompson CB (2016) The emerging hallmarks of cancer metabolism. *Cell Metab* 23: 27–47

Raj N, Wang M, Seoane JA, Zhao RL, Kaiser AM, Moonie NA, Demeter J, Boutelle AM, Kerr CH, Mulligan AS *et al* (2022) The Mettl3 epitranscriptomic writer amplifies p53 stress responses. *Mol Cell* 82: 2370–2384

Reckzeh ES, Waldmann H (2020) Small-molecule inhibition of glucose transporters GLUT-1-4. *Chembiochem* 21: 45–52

 © 2023 The Authors

Shao J, Shi T, Yu H, Ding Y, Li L, Wang X, Wang X (2022) Cytosolic GDH1 degradation restricts protein synthesis to sustain tumor cell survival following amino acid deprivation. *EMBO J* 41: e110306

Shen C, Xuan B, Yan T, Ma Y, Xu P, Tian X, Zhang X, Cao Y, Ma D, Zhu X *et al* (2020) m(6)A-dependent glycolysis enhances colorectal cancer progression. *Mol Cancer* 19: 72

Shi H, Wang X, Lu Z, Zhao BS, Ma H, Hsu PJ, Liu C, He C (2017) YTHDF3 facilitates translation and decay of N(6)-methyladenosine-modified RNA. *Cell Res* 27: 315–328

Shi H, Wei J, He C (2019) Where, when, and how: context-dependent functions of RNA methylation writers, readers, and erasers. *Mol Cell* 74: 640–650

Siegel RL, Miller KD, Fuchs HE, Jemal A (2021) Cancer statistics, 2021. *CA Cancer J Clin* 71: 7–33

Su R, Dong L, Li C, Nachtergaele S, Wunderlich M, Qing Y, Deng X, Wang Y, Weng X, Hu C *et al* (2018) R-2HG exhibits anti-tumor activity by targeting FTO/m(6)A/MYC/CEBPA signaling. *Cell* 172: 90–105

Vivona AA, Shpitz B, Medline A, Bruce WR, Hay K, Ward MA, Stern HS, Gallinger S (1993) K-ras mutations in aberrant crypt foci, adenomas and adenocarcinomas during azoxymethane-induced colon carcinogenesis. *Carcinogenesis* 14: 1777–1781

Vousden KH, Prives C (2009) Blinded by the light: the growing complexity of p53. *Cell* 137: 413–431

Vousden KH, Ryan KM (2009) p53 and metabolism. *Nat Rev Cancer* 9: 691–700

Walther A, Johnstone E, Swanton C, Midgley R, Tomlinson I, Kerr D (2009) Genetic prognostic and predictive markers in colorectal cancer. *Nat Rev Cancer* 9: 489–499

Wang X, Lu Z, Gomez A, Hon GC, Yue Y, Han D, Fu Y, Parisien M, Dai Q, Jia G *et al* (2014) N6-methyladenosine-dependent regulation of messenger RNA stability. *Nature* 505: 117–120

Wang X, Feng J, Xue Y, Guan Z, Zhang D, Liu Z, Gong Z, Wang Q, Huang J, Tang C *et al* (2016) Structural basis of N(6)-adenosine methylation by the METTL3-METTL14 complex. *Nature* 534: 575–578

Wang S, Peng Z, Wang S, Yang L, Chen Y, Kong X, Song S, Pei P, Tian C, Yan H *et al* (2018) KRAB-type zinc-finger proteins PITA and PISA specifically regulate p53-dependent glycolysis and mitochondrial respiration. *Cell Res* 28: 572–592

Wang Q, Chen C, Ding Q, Zhao Y, Wang Z, Chen J, Jiang Z, Zhang Y, Xu G, Zhang J *et al* (2020) METTL3-mediated m(6)A modification of HDGF mRNA promotes gastric cancer progression and has prognostic significance. *Gut* 69: 1193–1205

Weigmann B, Tubbe I, Seidel D, Nicolaev A, Becker C, Neurath MF (2007) Isolation and subsequent analysis of murine lamina propria mononuclear cells from colonic tissue. *Nat Protoc* 2: 2307–2311

Xu RH, Pelicano H, Zhou Y, Carew JS, Feng L, Bhalla KN, Keating MJ, Huang P (2005) Inhibition of glycolysis in cancer cells: a novel strategy to overcome drug resistance associated with mitochondrial respiratory defect and hypoxia. *Cancer Res* 65: 613–621

Yang Z, Yang S, Cui YH, Wei J, Shah P, Park G, Cui X, He C, He YY (2021) METTL14 facilitates global genome repair and suppresses skin tumorigenesis. *Proc Natl Acad Sci USA* 118: e2025948118

Zhang C, Lin M, Wu R, Wang X, Yang B, Levine AJ, Hu W, Feng Z (2011) Parkin, a p53 target gene, mediates the role of p53 in glucose metabolism and the Warburg effect. *Proc Natl Acad Sci USA* 108: 16259–16264

Zhang S, Zhao BS, Zhou A, Lin K, Zheng S, Lu Z, Chen Y, Sulman EP, Xie K, Bögler O *et al* (2017) m(6)A Demethylase ALKBH5 maintains tumorigenicity of glioblastoma stem-like cells by sustaining FOXM1 expression and cell proliferation program. *Cancer Cell* 31: 591–606

Zhang D, Tang Z, Huang H, Zhou G, Cui C, Weng Y, Liu W, Kim S, Lee S, Perez-Neut M *et al* (2019) Metabolic regulation of gene expression by histone lactylation. *Nature* 574: 575–580

Zhou KI, Shi H, Lyu R, Wylder AC, Matuszek Ż, Pan JN, He C, Parisien M, Pan T (2019) Regulation of co-transcriptional pre-mRNA splicing by m(6)A through the low-complexity protein hnRNPG. *Mol Cell* 76: 70–81

License: This is an open access article under the terms of the Creative Commons Attribution License, which permits use, distribution and reproduction in any medium, provided the original work is properly cited.

© 2023 The Authors

Expanded View Figures

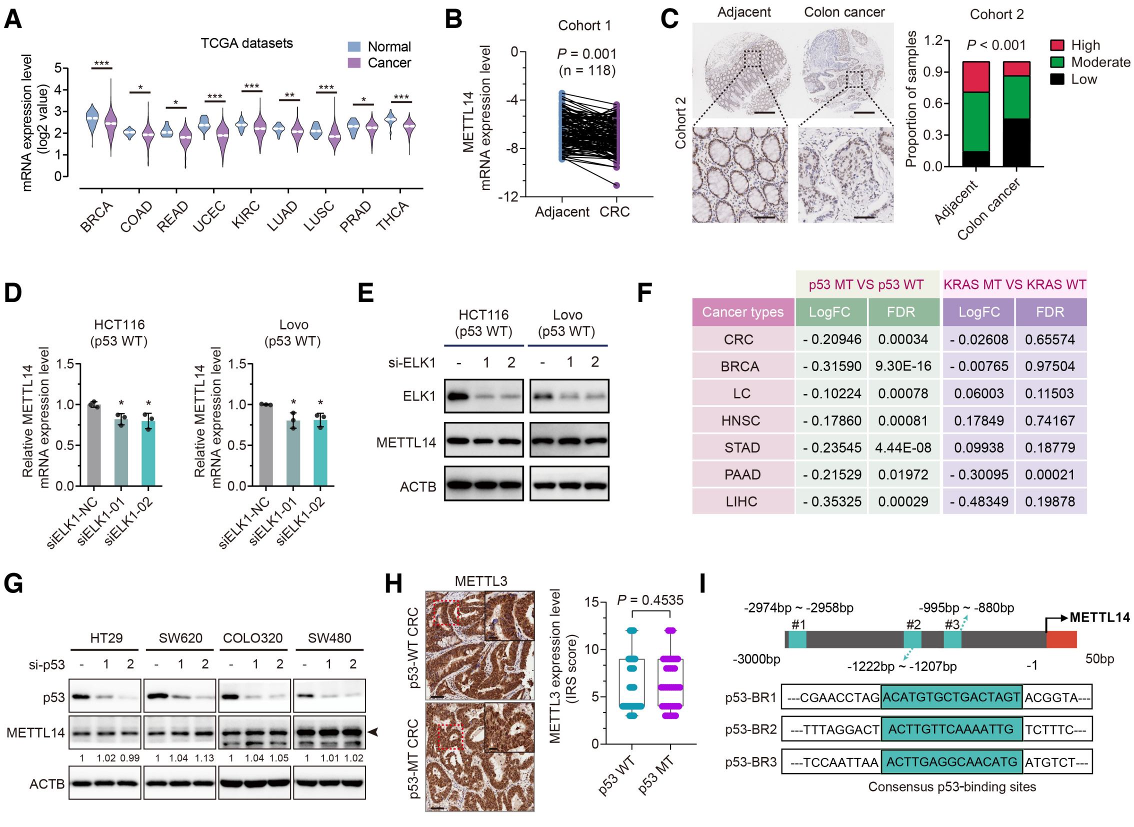

Figure EV1. Wild-type p53 activates METTL14 expression in CRC cell lines.

A METTL14 mRNA levels between tumor and normal tissues in the TCGA database (*$P < 0.05$, **$P < 0.01$, ***$P < 0.001$). Breast cancer (BRCA—normal tissues $n = 113$, cancer tissues $n = 1{,}104$); colon cancer (COAD—normal tissues $n = 41$, cancer tissues $n = 471$); rectal cancer (READ—normal tissues $n = 10$, cancer tissues $n = 167$); endometrioid cancer (UCEC—normal tissues $n = 35$, cancer tissues $n = 548$); kidney clear cell carcinoma (KIRC—normal tissues $n = 72$, cancer tissues $n = 535$); lung adenocarcinoma (LUAD—normal tissues $n = 59$, cancer tissues $n = 526$); lung squamous cell carcinoma (LUSC—normal tissues $n = 49$, cancer tissues $n = 501$); prostate cancer (PRAD—normal tissues $n = 52$, cancer tissues $n = 499$); and thyroid cancer (THCA—normal tissues $n = 58$, cancer tissues $n = 510$).

B qRT–PCR determination of the METTL14 level in CRC and adjacent normal tissues from Cohort 1 ($n = 118$).

C Representative IHC staining images and statistical analysis of METTL14 in tumor and para-tumor tissues from Cohort 2 ($n = 90$). Lower panels show enlarged images of indicated normal or CRC tissues. Scale bars = 200 μm (upper) and 40 μm (lower).

D qRT–PCR analysis of the mRNA levels of METTL14 after transfection with control or ELK1 siRNAs with 48 h treatment in HCT116 and Lovo cells. Data are presented as mean ± SD (biological replicates, $n = 3$; *$P < 0.05$).

E Western blot analysis of METTL14 and ELK1 protein levels after transfection with control or ELK1 siRNAs with 48 h treatment in HCT116 and Lovo cells.

F Expression analysis of METTL14 in p53-WT and p53-MT or KRAS-WT and KRAS-MT tumor tissues from in TCGA. Lung Cancer (LC); Head and Neck Cancer (HNSC); Stomach Cancer (STAD); Liver Cancer (LIHC) and Pancreatic Cancer (PAAD).

G HT29 (p53R273H), SW620 (p53R273H), COLO320 (p53R248W) and SW480 (p53R273H/P309S) cells were transfected with control or p53 siRNAs. Forty-eight hours after transfection, total protein was then analyzed by western blot analysis. Arrows indicate METTL14 protein.

H Representative IHC images, and statistical analysis of immunoreactive score (IRS) of METTL3 expression in p53-WT ($n = 63$) and p53-MT ($n = 41$) CRC samples from Cohort 3. The insets show enlarged images of indicated p53-WT and p53-MT CRC tissues, respectively. Scale bars = 20 μm and 2 μm (inset). The horizontal lines represent the median; the bottom and top of the boxes represent the 25 and 75% percentiles, respectively, and the vertical bars represent the range of the data.

I Schematic illustration of putative p53-binding site in METTL14 gene promoter.

Data information: For (A, B, D, and F), statistical significance was determined by a two-tailed Student's *t*-test. For (H), statistical significance was determined by the non-parametric Mann–Whitney test. For (C), statistical significance was determined by the χ^2 test. ACTB was used as a loading control.

 © 2023 The Authors

Figure EV2. METTL14 inhibits p53-WT CRC progression.

A, B qRT–PCR and Western blot validation of the METTL14 knockdown efficiency by stable transfection with shNC or shMETTL14 in p53-WT (HCT116 and Lovo) and p53-MT (HT29 and SW620) cells. Data are presented as mean ± SD (biological replicates $n = 3$; $***P < 0.001$).

C Cell viability assay of CRC cells stably transfected with lentivirus carrying control shRNA (shNC) or METTL14 shRNA (shMETTL14) for indicated time (0, 24, 48, and 96 h). Data are presented as mean ± SD (biological replicates, $n = 6$; $***P < 0.001$, ns, no significance).

D Representative HE and IHC staining images and quantitative analysis of Ki-67 in subcutaneous tumors from nude mice in Fig 2B and C. Scale bars = 10 μm. Data are presented as mean ± SD (biological replicates, $n = 6$; ns = no significance, $***P < 0.001$).

E Schematic diagrams of generation of Villin-Cre^{+}/Mettl14$^{FL/FL}$ conditional knockout mice.

F Representative IHC staining images of METTL14 in organs other than colorectum, including lung, liver, spleen, kidney, stomach, and heart, from Mettl14$^{\Delta IEC}$ ($n = 34$) and Mettl14^{WT} ($n = 38$) mice. The insets show enlarged images of indicated tissues. Scale bars = 400 μm and 10 μm (inset).

G Gross appearance of Mettl14$^{\Delta IEC}$ ($n = 13$, $n = 21$) and Mettl14^{WT} ($n = 14$, $n = 24$) C57BL/6 mice from AOM/DSS-induced (Left) and AOM-induced (Right) CRC model.

H The body weight changes during the course of acute colitis with DSS in Mettl14$^{\Delta IEC}$ ($n = 13$) and Mettl14^{WT} ($n = 14$) mice were recorded and expressed as the ratio relative to the initial weight before DSS treatment. Data are expressed as mean ± SD.

I Representative morphology of spleens in Mettl14$^{\Delta IEC}$ ($n = 13$, $n = 21$) and Mettl14^{WT} ($n = 14$, $n = 24$) mice extracted from AOM/DSS-induced (Left) and AOM-induced (Right) CRC models.

J Representative HE staining images of colorectum of Mettl14$^{\Delta IEC}$ ($n = 21$) and Mettl14^{WT} ($n = 24$) mice from AOM/DSS-induced (Left) and AOM-induced (Right) CRC model, showing representative inflammation. Lower panels show the magnified images of the indicated regions. Scale bars = 400 μm (upper) and 100 μm (lower).

Data information: For (A, C, D, and H), statistical significance was determined by a two-tailed Student's *t*-test. ACTB was used as a loading control.

© 2023 The Authors

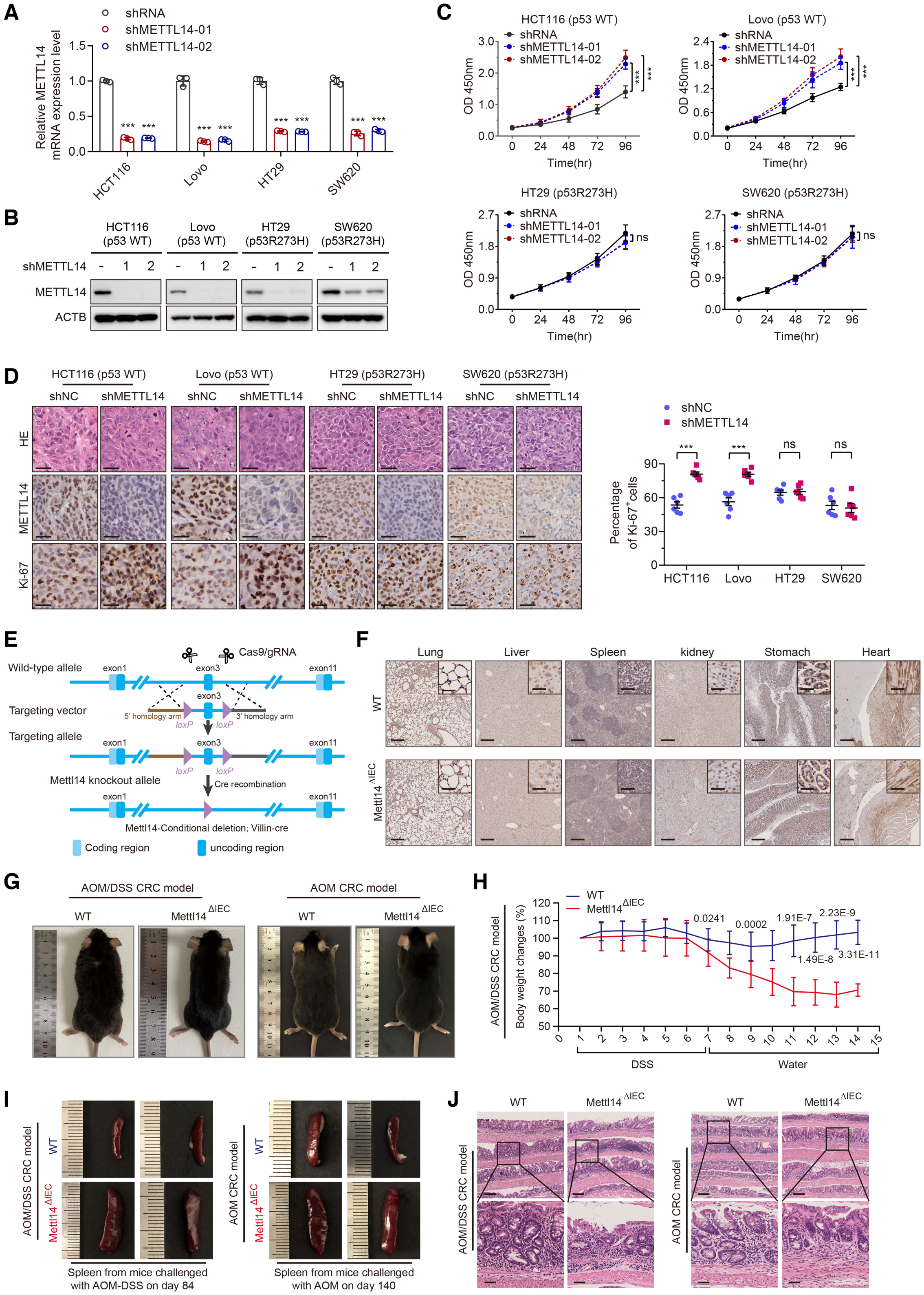

Figure EV2.

 © 2023 The Authors

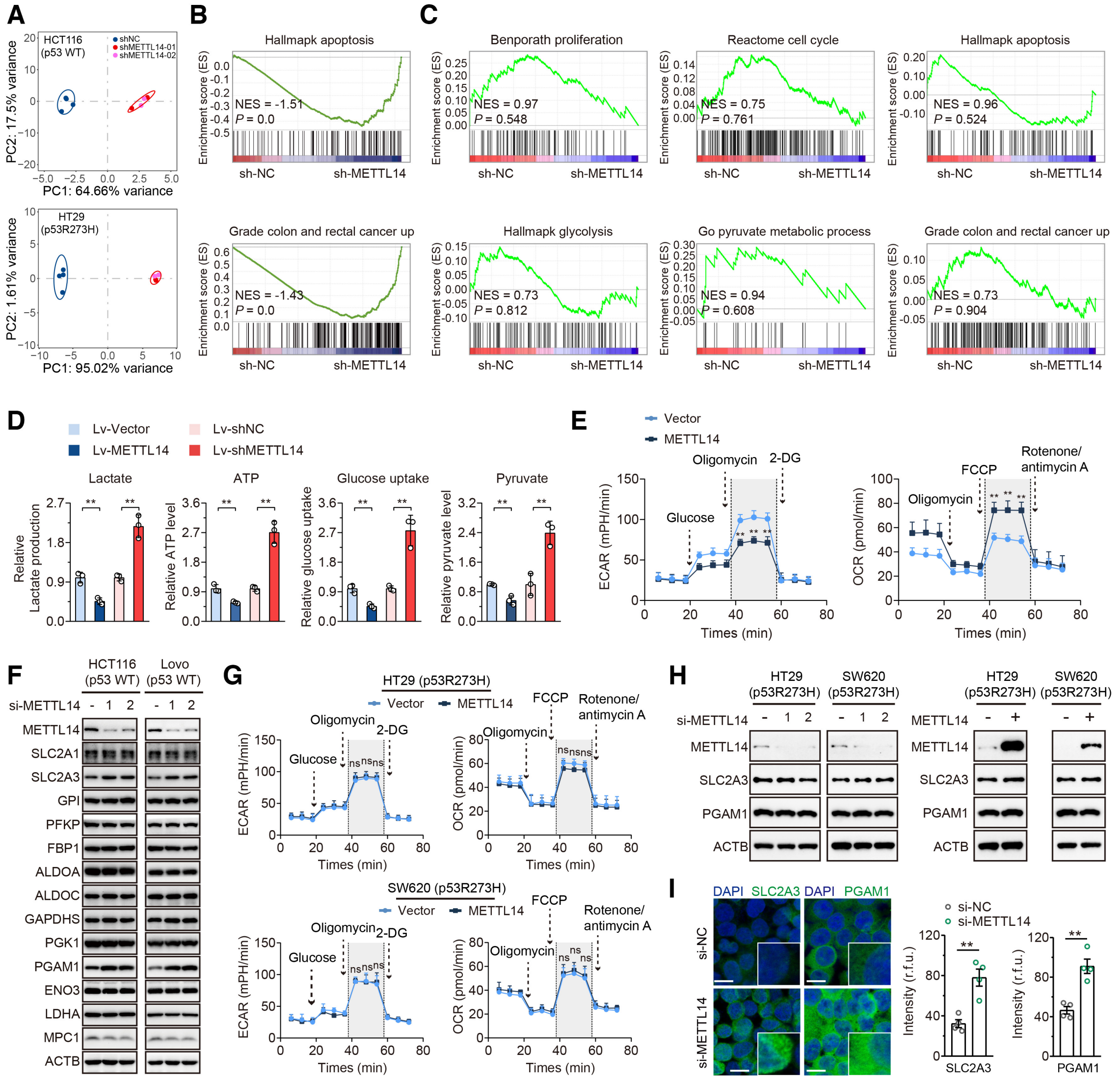

Figure EV3. METTL14 suppresses glycolysis of p53-WT CRC cells by targeting SLC2A3 and PGAM1.

A Principal component analysis of gene expression profiles of shNC ($n = 4$), shMETTL14-01 ($n = 2$), and shMETTL14-02 ($n = 2$) shRNA-expressing p53-WT HCT116 and p53-MT HT29 cells.

B, C Overview of GSEA used to identify the differential gene profiles in HCT116 (p53-WT) and HT29 (p53-MT) cells stably transfected with shNC and shMETTL14, respectively.

D Lactate production, ATP level, glucose uptake, and pyruvate level were determined in Lovo (p53-WT) cells stably transfected with Lv-vector or Lv-METTL14, or with shNC or shMETTL14. Data are presented as mean ± SD (biological replicates, $n = 3$; $^{**}P < 0.01$).

E ECAR and OCR were measured in Lovo (p53-WT) cells stably transfected Lv-vector or Lv-METTL14. Data are presented as mean ± SD (biological replicates, $n = 3$; $^{**}P < 0.01$).

F Western blot analysis of glycolytic gene expression in HCT116 and Lovo (p53-WT) cells transfected with control siRNA or METTL14 siRNAs for 48 h.

G ECAR and OCR were measured in p53-MT (HT29 and SW620) cells stably transfected with vector or METTL14. Data are presented as mean ± SD (biological replicates, $n = 3$; ns, no significance).

H Western blot analysis of METTL14, SLC2A3, and PGAM1 protein levels in p53-MT (HT29 and SW620) cells transfected with control siRNA or METTL14 siRNAs, and empty vector or METTL14 plasmid.

I Representative immunofluorescence (IF) staining and quantitative analysis of SLC2A3 (green) and PGAM1 (green) proteins in HCT116 (p53-WT) cells transfected with control or METTL14 siRNA. Nuclei were stained with DAPI (blue). The insets show enlarged images of CRC cells. Scale bars = 10 μm. Data are presented as mean ± SD (biological replicates, $n = 4$; $^{**}P < 0.01$).

Data information: For (D, E, G, I,), statistical significance was determined by a two-tailed Student's t-test. ACTB was used as a loading control. Source data are available online for this figure.

Figure EV4. METTL14 decreases SLC2A3 and PGAM1 levels by m^{6}A-YTHDF2-mediated pri-miR-6769b and pri-miR-499a processing.

A Schematic illustration of the protocol for screening miRNAs that are regulated by METT14 and simultaneously target SLC2A3 and PGAM1 using miRNA Microarray, TargetScan (http://www.targetscan.org/vert_71/) and miRDB (http://mirdb.org/miRDB/) database.

B qRT–PCR analysis of the SLC2A3 and PGAM1 levels in HCT116 and Lovo (p53-WT) cells transfected with miR-6769b-3p and miR-499a-3p mimics and corresponding inhibitors. Data are presented as mean ± SD (biological replicates, $n = 3$; $^{**}P < 0.01$, $^{***}P < 0.001$).

C Heatmap of known METTL14 target miRNAs in p53-WT cells identified by miRNA microarrays using HT29 cells stably infected with lentivirus carrying METTL14 overexpression or control vector.

D qRT–PCR analysis of the miR-6769b-3p and miR-499a-3p levels in stably transfected vector and METTL14 HT29 (p53-MT) cells. Data are presented as mean ± SD (biological replicates, $n = 3$; ns = no significance).

E Western blot analysis of SLC2A3 and PGAM1 in HT29 and SW620 (p53-MT) cells transfected with control or miRNA mimics and corresponding inhibitors.

F Schematic diagram of luciferase reporters expressing wild-type or mutant SLC2A3 3′UTRs and wild-type or mutant PGAM1 3′UTRs predicted by TargetScan and miRDB database.

G qRT–PCR was performed to determine the pri-miR-6769b and pri-miR-499a levels in HCT116 and Lovo (p53-WT) cells transfected with control siRNA or METTL14 siRNAs for 48 h. Data are presented as mean ± SD (biological replicates, $n = 3$; $^{**}P < 0.01$).

H qRT–PCR was performed to determine the miR-6769b-3p/miR-499a-3p and pri-miR-6769b/pri-miR-499a levels in p53-MT (HT29 and SW620) cells transfected with control or METTL14 siRNAs for 48 h. Data are presented as mean ± SD (biological replicates, $n = 3$; ns = no significance).

I The sequences of pri-miR-6769b/pre-miR-6769b/miR-6769b-3p and pri-miR-499a/pre-miR-499a/miR-499a-3p are presented and highlighted by different colors. The m^{6}A sites were predicted by SRAMP.

J qRT–PCR analysis of the pri-miR-6769b/pre-miR-6769b/miR-6769b-3p levels in HCT116 cells transfected with wild-type or mutant pri-miR-6769b plasmids. Data are presented as mean ± SD (biological replicates, $n = 3$, $^{**}P < 0.01$).

K qRT–PCR analysis of the pri-miR-499a/pre-miR-499a/miR-499a-3p levels in HCT116 cells transfected with wild-type or mutant pri-miR-499a plasmids. Data are presented as mean ± SD (biological replicates, $n = 3$, $^{**}P < 0.01$).

L qRT–PCR analysis of the pri-miR-6769b/pri-miR-499a levels in HCT116 cells transfected with empty vector, mutant METTL14 (MT-METTL14) or wild-type METTL14 (WT-METTL14) plasmids for 48 h. Data are presented as mean ± SD (biological replicates, $n = 3$).

M Western blot and m^{6}A dot blot analyses of METTL14, SLC2A3, PGAM1, and global m^{6}A levels in HCT116 cells transfected with empty vector, MT-METTL14 or WT-METTL14 plasmids for 48 h.

Data information: For (B–D, G, H, J–L), statistical significance was determined by a two-tailed Student's *t*-test. ACTB and methylene blue were used as a loading control.

 © 2023 The Authors

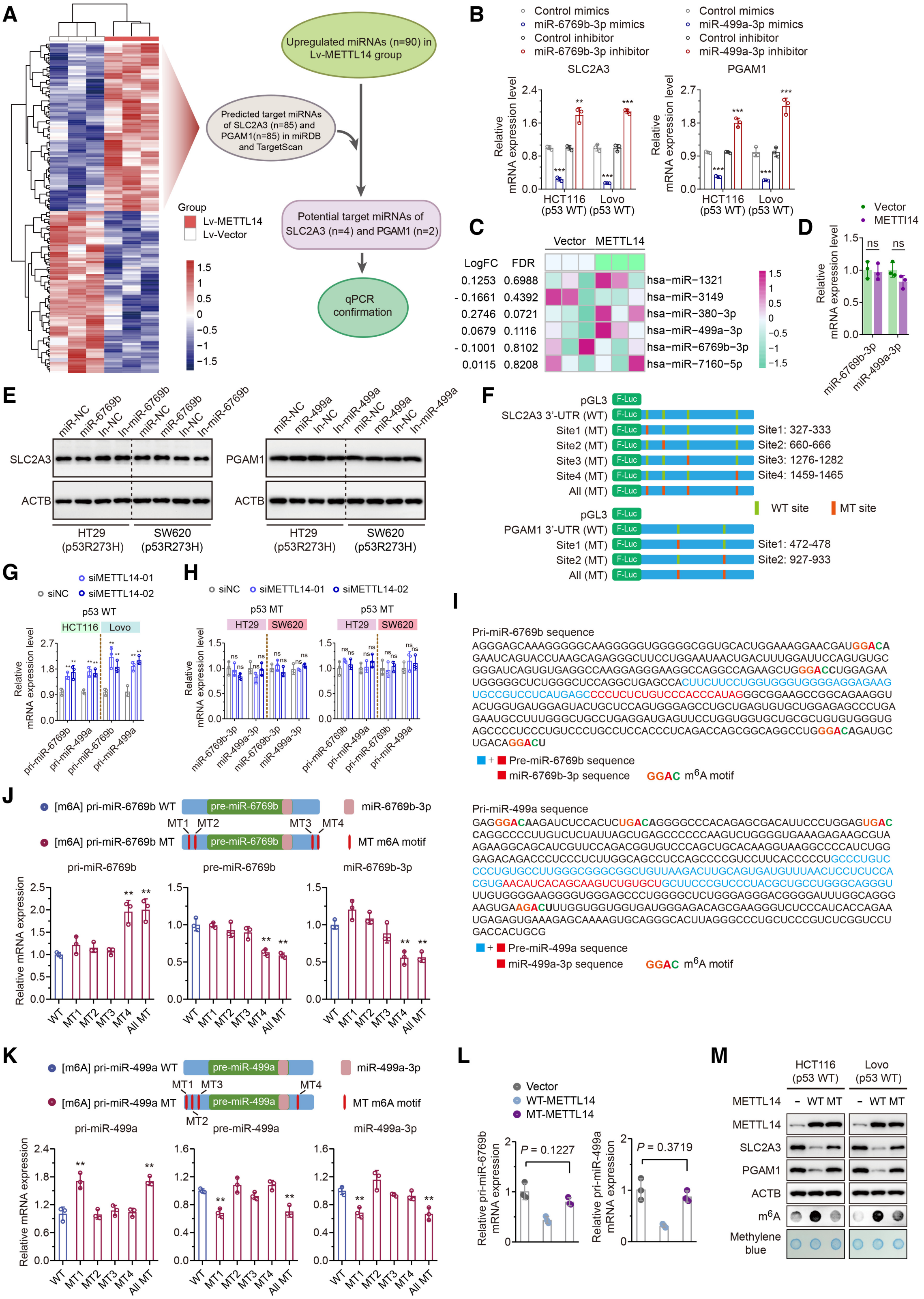
A
Upregulated miRNAs (n=90) in Lv-METTL14 group
Predicted target miRNAs of SLC2A3 (n=85) and PGAM1(n=85) in miRDB and TargetScan
Potential target miRNAs of SLC2A3 (n=4) and PGAM1 (n=2)
qPCR confirmation
Group
Lv-METTL14
Lv-Vector
B
Control mimics
miR-6769b-3p mimics
Control inhibitor
miR-6769b-3p inhibitor
miR-499a-3p mimics
miR-499a-3p inhibitor
SLC2A3
PGAM1
Relative mRNA expression level
HCT116 (p53 WT)
Lovo (p53 WT)
C
Vector
METTL14
LogFC
FDR
0.1253 0.6988 hsa−miR−1321
- 0.1661 0.4392 hsa−miR−3149
0.2746 0.0721 hsa−miR−380−3p
0.0679 0.1116 hsa−miR−499a−3p
- 0.1001 0.8102 hsa−miR−6769b−3p
0.0115 0.8208 hsa−miR−7160−5p
D
Vector
METTl14
ns
miR-6769b-3p
miR-499a-3p
E
miR-NC
miR-6769b
In-NC
In-miR-6769b
miR-499a
In-miR-499a
SLC2A3
PGAM1
ACTB
HT29 (p53R273H)
SW620 (p53R273H)
F
pGL3
SLC2A3 3'-UTR (WT)
Site1 (MT)
Site2 (MT)
Site3 (MT)
Site4 (MT)
All (MT)
F-Luc
Site1: 327-333
Site2: 660-666
Site3: 1276-1282
Site4: 1459-1465
WT site
MT site
PGAM1 3'-UTR (WT)
Site1: 472-478
Site2: 927-933
G
siNC
siMETTL14-01
siMETTL14-02
p53 WT
HCT116
Lovo
pri-miR-6769b
pri-miR-499a
H
p53 MT
HT29
SW620
miR-6769b-3p
miR-499a-3p
I
Pri-miR-6769b sequence
AGGGAGCAAAGGGGGCAAGGGGGUGGGGGCGGUGCACUGGAAAGGAACGAUGGACA
GAAUCAGUACCUAAGCAGAGGGCUUCCUGGAAUAACUGACUUUGGAUUCCAGUGUGC
GGGAUCAGUGUGAGGCCAAGGAGGGAAGGCCAGGCCAGAAGCUGGGACCUGGAGAA
UGGGGGCUCUGGGCUCCAGGCUGAGCCACUUCUUCCUGGUGGGUGGGGAGGAGAAG
UGCCGUCCUCAUGAGCCCCUCUCUGUCCCACCCAUAGGGCGGAAGCCGGCAGAAGGU
ACUGGUGAUGGAGUACUGCUCCAGUGGGAGCCUGCUGAGUGUGCUGGAGAGCCCUGA
GAAUGCCUUUGGGCUGCCUGAGGAUGAGUUCCUGGUGGUGCUGCGCUGUGUGGGUG
AGCCCCUCCCUGUCCCUGCCUCCACCCUCAGACCAGCGGCAGGCCUGGGACAGAUGC
UGACAGGACU
Pre-miR-6769b sequence
miR-6769b-3p sequence
GGAC m6A motif
Pri-miR-499a sequence
GAGGGACAAGAUCUCCACUCUGACAGGGGCCCACAGAGCGACAUUCCCUGGAGUGAC
CAGGCCCCUUGUCUCUAUUAGCUGAGCCCCCCAAGUCUGGGGUGAAAGAGAAGCGUA
AGAAGGCAGCAUCGUUCCAGACGGUGUCCCAGCUGCACAAGGUAAGGCCCCAUCUGG
GAGACAGACCCUCCCUCUUGGCAGCCUCCAGCCCCGUCCUUCACCCCCUGCCCUGUC
CCCUGUGCCUUGGGCGGGCGGCUGUUAAGACUUGCAGUGAUGUUUAACUCCUCUCCA
CGUGAACAUCACAGCAAGUCUGUGCUGCUUCCCGUCCCUACGCUGCCUGGGCAGGGU
UUGUGGGGAAGGGGUGGGAGCCCUGGGGCUCUGGGAGGGACGGGGAUUUGGCAGGG
AAGUGAAGACUUUGGUGGUGGUGAUGGGAGACAGCGAAGGGUCUCCCAUCACCAGAA
UGAGAGUGAAAGAGCAAAAGUGCAGGGCACUUAGGGCCCUGCUCCCGUCUCGGUCCU
GACCACUGCG
Pre-miR-499a sequence
miR-499a-3p sequence
GGAC m6A motif
J
[m6A] pri-miR-6769b WT
[m6A] pri-miR-6769b MT
pre-miR-6769b
miR-6769b-3p
MT m6A motif
MT1 MT2 MT3 MT4
pri-miR-6769b
pre-miR-6769b
miR-6769b-3p
Relative mRNA expression
WT
MT1
MT2
MT3
MT4
All MT
K
[m6A] pri-miR-499a WT
[m6A] pri-miR-499a MT
pre-miR-499a
miR-499a-3p
pri-miR-499a
L
Vector
WT-METTL14
MT-METTL14
P = 0.1227
P = 0.3719
Relative pri-miR-6769b mRNA expression
Relative pri-miR-499a mRNA expression
M
HCT116 (p53 WT)
Lovo (p53 WT)
METTL14
- WT MT
SLC2A3
PGAM1
ACTB
m6A
Methylene blue

Figure EV4.

Figure EV5. METTL14 attenuates the development of p53-WT CRC by repressing glycolysis through miR-6769b-3p/SLC2A3 and miR-499a-3p /PGAM1 axis.

A Lactate production, ATP level, glucose uptake, and pyruvate level were determined in HT29 (p53-MT) cells treated with control, miR-6769b-3p mimics, or inhibitor for 48 h. Data are presented as mean ± SD (biological replicates, n = 3; ns = no significance).

B Lactate production, ATP level, glucose uptake, and pyruvate level were determined in HT29 (p53-MT) cells treated with control, miR-499a-3p mimics, or inhibitor for 48 h. Data are presented as mean ± SD (biological replicates, n = 3; ns = no significance).

C ECAR and OCR were measured in HT29 (p53-MT) cells transfected with control or miR-6769b-3p mimics for 48 h. Data are presented as mean ± SD (biological replicates, n = 3; ns = no significance).

D ECAR and OCR were measured in HT29 (p53-MT) cells transfected with control or miR-499a-3p mimics for 48 h. Data are presented as mean ± SD (biological replicates, n = 3; ns = no significance).

E Cell viability assay was performed in HCT116 and Lovo (p53-WT) cells stably transfected with shNC or shMETTL14, or in the stable transfectants with shMETTL14 transfected with control, miR-6769b-3p or miR-499a-3p mimics. Data are presented as mean ± SD (biological replicates, n = 5, ***P < 0.001).

F Colony formation assay was performed in HCT116 and Lovo (p53-WT) cells stably transfected with shNC or shMETTL14, or in the stable transfectants with shMETTL14 transfected with control, miR-6769b-3p or miR-499a-3p mimics. Data are presented as mean ± SD (biological replicates, n = 3, **P < 0.01).

G Colony formation assay was performed in p53-WT (HCT116 and Lovo) and p53-MT (HT29 and SW620) cells stably transfected with control, shSLC2A3 or shPGAM1. Data are presented as mean ± SD (biological replicates, n = 3, ns = no significance, *P < 0.05, **P < 0.01).

H Western blot analysis of SLC2A3 and PGAM1 in p53-WT and p53-MT CRC cell lines.

I qRT–PCR analysis of miR-6769b-3p and miR-499a-3p mRNA level in p53-WT and p53-MT CRC cell lines. Data are presented as mean ± SD (biological replicates, n = 3, ns = no significance, *P < 0.05, **P < 0.01, ***P < 0.001). The dotted line represents basal mRNA expression of miR-6769b-3p and miR-499a-3p in RKO cells, acting as a control.

J Representative images of tumors and analysis in nude mice intervened with control, shSLC2A3 or shPGAM1 expression p53-WT (HCT116 and Lovo) cells. Data are presented as mean ± SD (biological replicates, n = 7).

K Representative images of tumors and analysis in nude mice intervened with control, shSLC2A3 or shPGAM1 expression p53-MT (HT29 and SW620) cells. Data are presented as mean ± SD (biological replicates, n = 7).

Data information: For (A–G, J, K), statistical significance was determined by a two-tailed Student's t-test. ACTB was used as a loading control.

 © 2023 The Authors

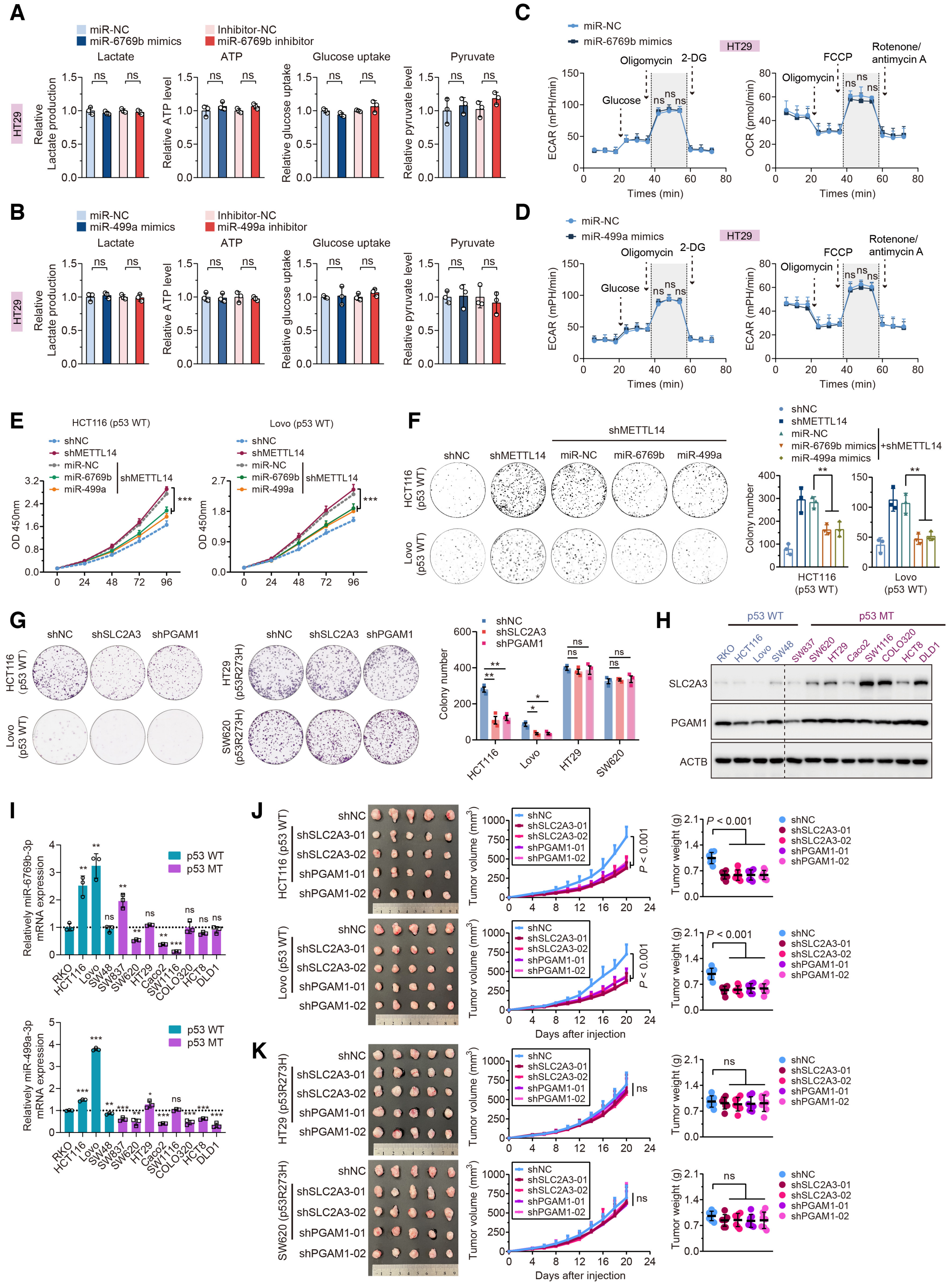
A
miR-NC
miR-6769b mimics
Inhibitor-NC
miR-6769b inhibitor
Lactate
ATP
Glucose uptake
Pyruvate
HT29
B
miR-499a mimics
miR-499a inhibitor
C
ECAR (mPH/min)
OCR (pmol/min)
Glucose
Oligomycin
2-DG
FCCP
Rotenone/ antimycin A
Times (min)
D
E
HCT116 (p53 WT)
Lovo (p53 WT)
shNC
shMETTL14
OD 450nm
F
Colony number
G
shSLC2A3
shPGAM1
HT29 (p53R273H)
SW620 (p53R273H)
H
p53 WT
p53 MT
SLC2A3
PGAM1
ACTB
I
Relatively miR-6769b-3p mRNA expression
Relatively miR-499a-3p mRNA expression
J
Tumor volume (mm3)
Tumor weight (g)
Days after injection
shSLC2A3-01
shSLC2A3-02
shPGAM1-01
shPGAM1-02
P < 0.001
K
ns

Figure EV5.

Appendix for METTL14 loss modulates glycolysis reprogramming to drive p53-wild type colorectal tumorigenesis

Yichao Hou[1,†], Xintian Zhang[1,†], Han Yao[1,†], Lidan Hou[1], Qingwei Zhang[2], Enwei Tao[2], Shanshan Jiang[2], Yimeng Ren[2], Xialu Hong[2], Shiyuan Lu[2], Xiaoxu Leng[2], Xiaoqiang Zhu[3], Yile Xie[2], Yaqi Gao[2], Yu Liang[1], Ting Zhong[1], Bohan Long[1], Jing-Yuan Fang [2,*], Xiangjun Meng[1,**]

Table of content Page

Appendix Figure S1……………………………………………………………2
Appendix Figure S2……………………………………………………………3
Appendix Figure S3……………………………………………………………4
Appendix Figure S4……………………………………………………………5
Appendix Figure S5……………………………………………………………6
Appendix Figure S6……………………………………………………………7
Appendix Figure S7……………………………………………………………8
Appendix Figure S8……………………………………………………………9
Appendix Figure S9…………………………………………………………..10
Appendix Table S1……………………………………………………..……..11
Appendix Table S2…………………………………………………..…12, 13
Appendix Table S3……………………………………………………………14

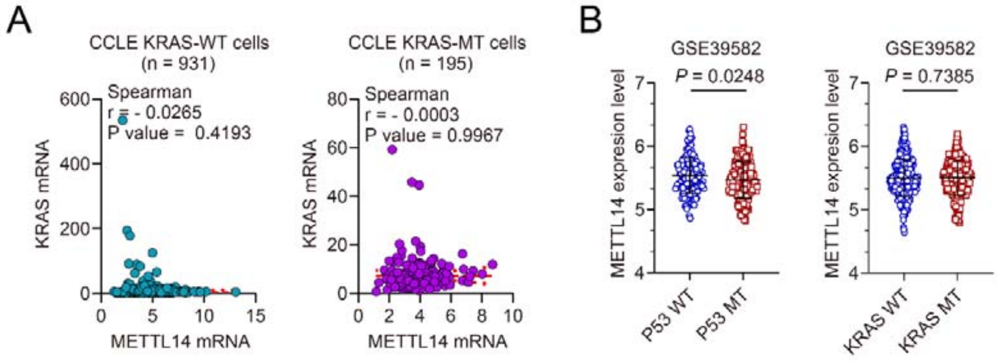

Appendix Figure S1. Correlations of METTL14 and p53 or KRAS in CRC cells or CRC tissues.

A Correlation of METTL14 and p53 mRNA levels in KRAS-wild type (KRAS-WT) and KRAS-mutant (KRAS-MT) CRC cell lines from CCLE database. Statistical significance was determined by analysis of the Pearson's correlation coefficient.

B. Expression analysis of METTL14 in p53-WT (Biological replicates n = 161) and p53-MT (Biological replicates n = 190) or KRAS-WT (Biological replicates n = 328) and KRAS-MT (Biological replicates n = 217) CRC tissues from GSE39582. Data are presented as mean ± SD. Statistical significance was determined by two-tailed Student's t test.

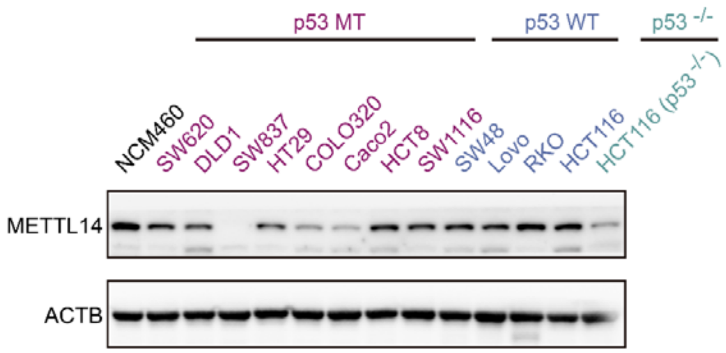

Appendix Figure S2. Western blot analysis of METTL14 protein level in normal (NCM460) and various CRC cell lines.

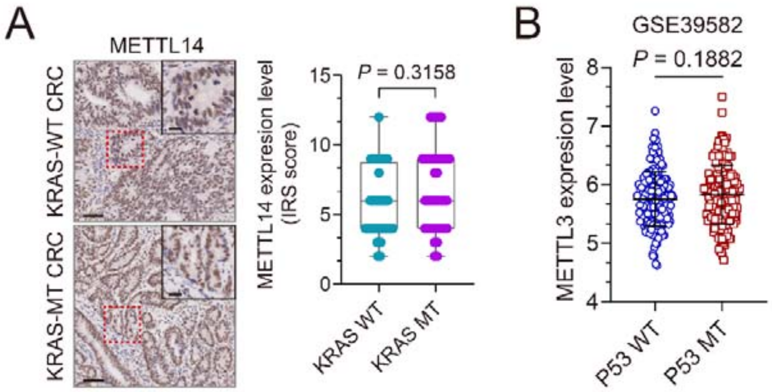

Appendix Figure S3. Expression of METTL3 in p53-WT and p53-MT CRC tissues.

A Representative IHC images, and statistical analysis of immunoreactive score (IRS) of METTL3 expression in p53-WT (Biological replicates n = 63) and p53-MT (Biological replicates n = 41) CRC samples. The insets show enlarged images of indicated p53-WT and p53-MT CRC tissues, respectively. Scale bars = 20 μm and 2 μm (inset). Statistical significance was determined by nonparametric Mann-Whitney test. The horizontal lines represent the median; the bottom and top of the boxes represent the 25% and 75% percentiles, respectively, and the vertical bars represent the range of the data.

B Expression of METTL3 in p53-WT (Biological replicates n = 161) and p53-MT (Biological replicates n =190) CRC tissues from GSE39582. Data are presented as mean ± SD. Statistical significance was determined by two-tailed Student's t test.

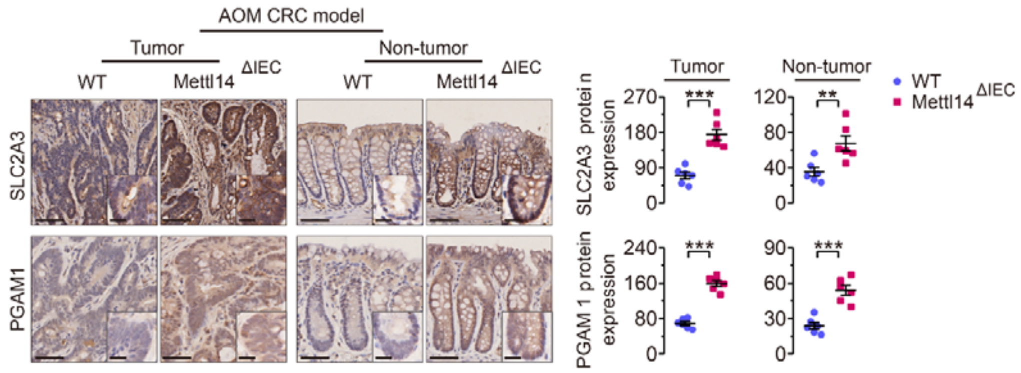

Appendix Figure S4. Representative IHC staining images and quantitative analysis of SLC2A3 and PGAM1 in tumor and non-tumor tissues from AOM-induced Mettl14$^{\Delta IEC}$ and Mettl14^{WT} mice CRC models.

The insets show enlarged images of tumor tissues and non-tumor tissues, respectively. Scale bars = 20 μm and 2 μm (inset). Data are presented as mean ± SD (Biological replicates n = 6; ****P* < 0.001). Statistical significance was determined by two-tailed Student’s t test.

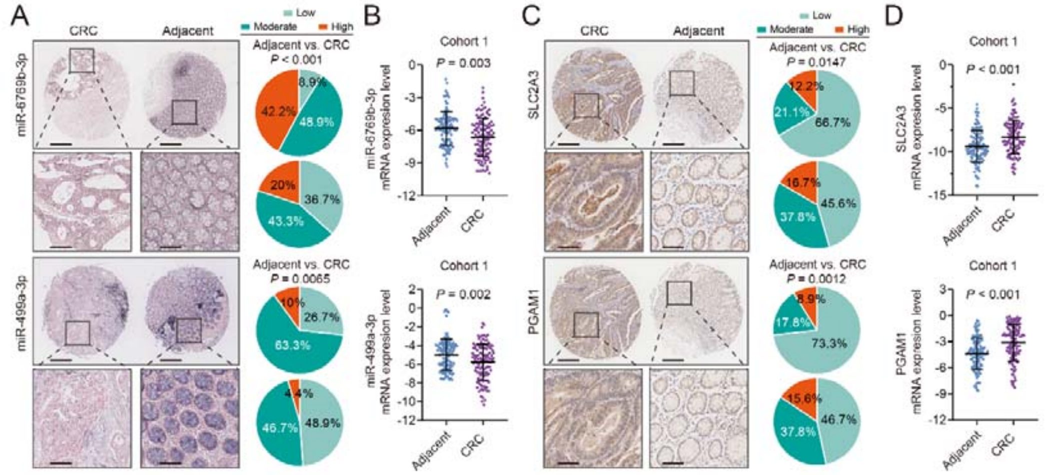

Appendix Figure S5. Expression levels of miR-6769b-3p/miR-499a-3p and SLC2A3/PGAM1 in CRC and adjacent tissues of patients.

A Representative IHC staining images and corresponding quantitative analysis of SLC2A3 and PGAM1 in CRC and adjacent tissues of patients from Cohort 2 (n = 90). Lower panels show enlarged images of indicated normal or CRC tissues. Scale bars = 200 μm (upper) and 40 μm (lower). Data are presented as mean ± SD. Percentages of low, moderate and high expression of SLC2A3 (upper) and PGAM1 (bottom) in CRC and adjacent tissues are shown as pie charts.

B qRT-PCR analysis of SLC2A3 (upper) and PGAM1(bottom) in CRC and adjacent tissues of patients from Jiuyuan Cohort 2 (n = 118). Data are presented as mean ± SD. Statistical significance was determined by two-tailed Student's t test.

C Representative ISH staining images and corresponding quantitative analysis of miR-6769b-3p and miR-499a-3p in CRC and adjacent tissues of patients from Cohort 2 (n = 90). Lower panels show enlarged images of indicated normal or CRC tissues. Scale bars = 200 μm (upper) and 40 μm (lower). Data are presented as mean ± SD. Percentages of low, moderate and high expression of miR-6769b-3p (upper) and miR-499a-3p (bottom) in CRC and adjacent tissues are shown as pie charts.

D qRT-PCR analysis of miR-6769b-3p (upper) and miR-499a-3p (bottom) in CRC and adjacent tissues of patients from Jiuyuan Cohort 2 (n = 118). Data are presented as mean ± SD. Statistical significance was determined by two-tailed Student's t test.

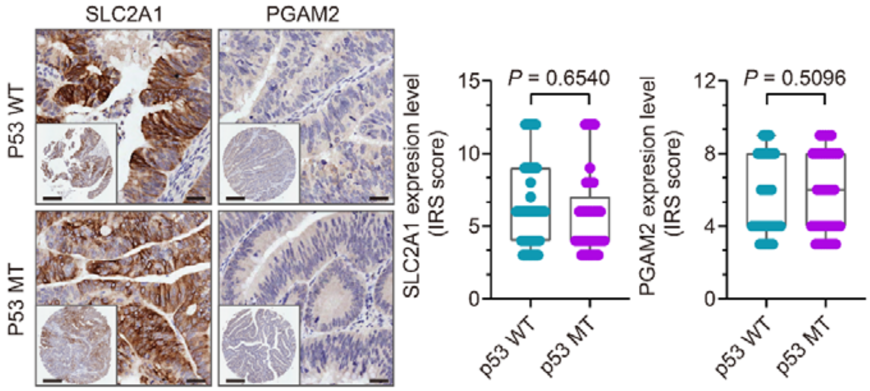

Appendix Figure S6. Expression levels of SLC2A1 and PGAM2 in p53-WT and p53-MT samples.

Representative IHC staining images and corresponding quantitative analysis of SLC2A3 and PGAM2 IRS in p53-WT (n = 63) and p53-MT (n = 41) samples from Cohort 3. The insets show enlarged images of indicated p53-WT and p53-MT CRC tissues, respectively. Scale bars = 200 μm and 20 μm (inset). Statistical significance was determined by nonparametric Mann-Whitney test. The horizontal lines represent the median; the bottom and top of the boxes represent the 25% and 75% percentiles, respectively, and the vertical bars represent the range of the data.

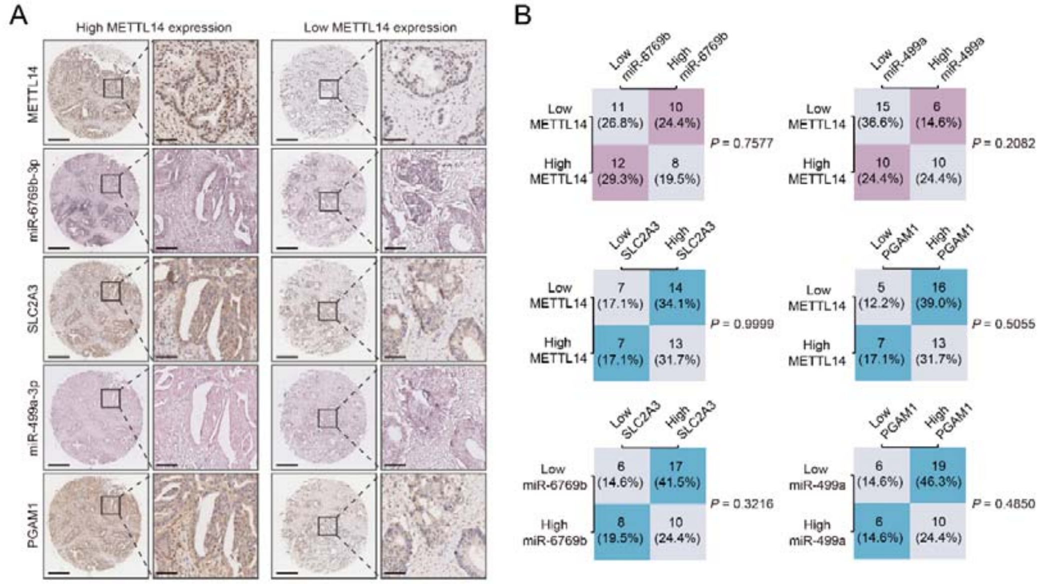

Appendix Figure S7. Correlation analysis of METTL14, SLC2A3, PGAM1, miR-6769b-3p and miR-499a-3p in p53-MT CRC tissues.

A Representative IHC and ISH images of METTL14, SLC2A3, PGAM1, miR-6769b-3p and miR-499a-3p in CRC tissues with higher or lower METTL14 expression in p53-MT (n = 41) samples from Cohort 3. The right panel show enlarged images of indicated p53-MT CRC tissues. Scale bars = 200 μm (left) and 20 μm (right).

B Statistical analysis of METTL14, SLC2A3, PGAM1, miR-6769b-3p and miR-499a-3p in CRC tissues with higher or lower METTL14 expression in p53-MT (n = 41) samples from Cohort 3. Statistical significance was determined by Fisher Exact test.

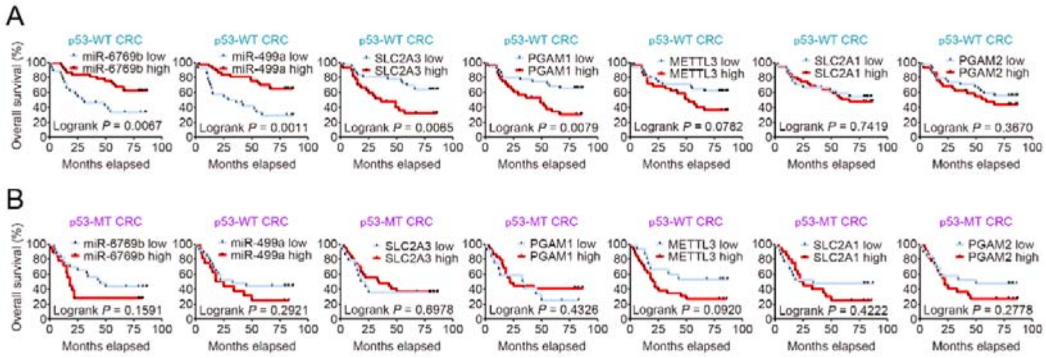

Appendix Figure S8. Survival analysis of CRC patients based on expression levels of miR-6769b-3p, miR-499a-3p, SLC2A3, PGAM1, METTL3, SLC2A1 and PGAM2.

A Kaplan-Meier survival curves of OS in CRC patients with wild type p53 (n = 63) from Cohort 3 database based on expression levels of miR-6769b-3p, miR-499a-3p, SLC2A3, PGAM1, METTL3, SLC2A1 and PGAM2. Statistical significance was performed by log-rank test.

B Kaplan-Meier survival curves of OS in CRC patients with mutant p53 (n = 41) from Cohort 3 database based on expression levels of miR-6769b-3p, miR-499a-3p, SLC2A3, PGAM1, METTL3, SLC2A1 and PGAM2. Statistical significance was performed by log-rank test.

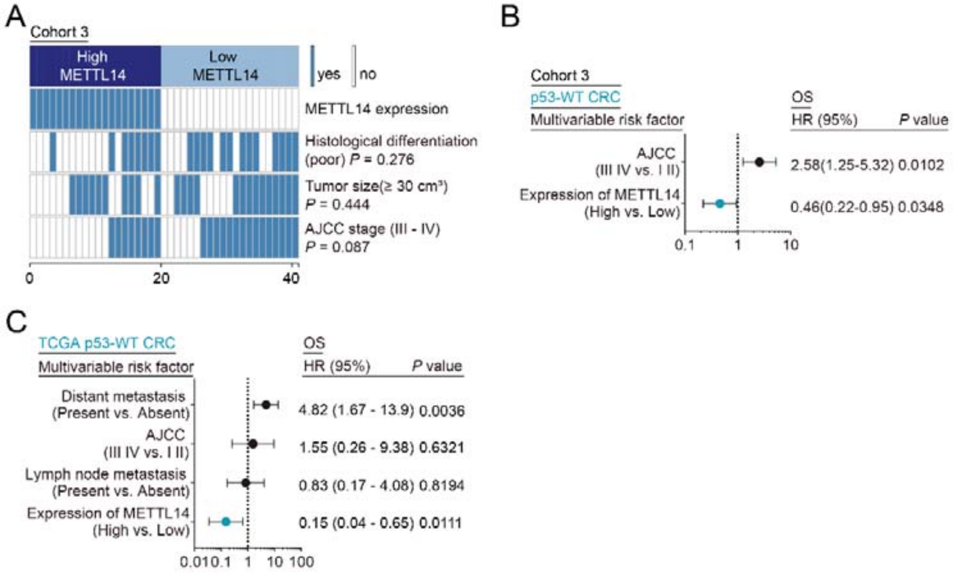

Appendix Figure S9. Correlation analysis of METTL14 and clinicopathological characteristics or OS in p53-MT patients.

A Comparison of clinicopathological characteristics between METTL14 high and low-expression tumors in p53-MT patients from Cohort 3 (n = 41). Statistical significance was performed by the Chi-square test.

B, C Multivariate Cox regression analysis of clinicopathologic factors for OS applied in p53-WT samples from Cohort 3 and TCGA CRC.

Appendix Table S1. The sequences of siRNAs, microRNA mimics and inhibitors used this study.

Gene	Sense	Antisense
METTL14-1	GGAUGAAGGAGAGACAGAUTT	AUCUGUCUCUCCUUCAUCCTT
METTL14-2	GCAGCACCUCGAUCAUUUATT	UAAAUGAUCGAGGUGCUGCTT
p53-1	GUAAUCUACUGGGACGGAATT	UUCCGUCCCAGUAGAUUACTT
p53-2	GAAGAAACCACUGGAUGGATT	UCCAUCCAGUGGUUUCUUCTT
ELK1-1	GCUGAGAGAGCAAGGCAAUTT	AUUGCCUUGCUCUCUCAGCTT
ELK1-2	CCCUUCUAUCAGCGUGGAUTT	AUCCACGCUGAUAGAAGGGTT
HIF-1α	GCUGAUUUGUGAACCCAUUTT	AAUGGGUUCACAAAUCAGCTT
MYC	GAGGAUAUCUGGAAGAAAUTT	AUUUCUUCCAGAUAUCCUCTT
SLC2A3-1	GUAGCUAAGUCGGUUGAAATT	UUUCAACCGACUUAGCUACTT
SLC2A3-2	CCUACAAAGUGCAGCCCUUTT	AAGGGCUGCACUUUGUAGGTT
PGAM1-1	GGCUAUGAGUUUGACAUCUTT	AGAUGUCAAACUCAUAGCCTT
PGAM1-2	CCUCAAUGAGCGGCACUAUTT	AUAGUGCCGCUCAUUGAGGTT
YTHDF1	GGCGUGUGUUCAUCAUCAATT	UUGAUGAUGAACACACGCCTT
YTHDF2	CCUACCAGAUGCAAUGUUUTT	AAACAUUGCAUCUGGUAGGTT
YTHDF3	AGCAGAGGAAACAGGCGAATT	UUCGCCUGUUUCCUCUGCUTT
YTHDC1	GGAGGAAGAAGAAGAAUAUTT	AUAUUCUUCUUCUUCCUCCTT
YTHDC2	GCGACUCAACAAUGGCAUATT	UAUGCCAUUGUUGAGUCGCTT
IGF2BP1	CCGCUUGUAAGAUGAUCUUTT	AAGAUCAUCUUACAAGCGGTT
IGF2BP2	GCGAAAGGAUGGUCAUCAUTT	AUGAUGACCAUCCUUUCGCTT
IGF2BP3	GCUGGAGCUUCAAUUAAGATT	UCUUAAUUGAAGCUCCAGCTT
hsa-miR-6769b-3p mimics	CCCUCUCUGUCCCACCCAUAG	AUGGGUGGGACAGAGAGGGUU
hsa-miR-1321 mimics	CAGGGAGGUGAAUGUGAU	CACAUUCACCUCCCUGUU
hsa-miR-7160-5p mimics	UGCUGAGGUCCGGGCUGUGCC	CACAGCCCGGACCUCAGCAUU
hsa-miR-380-3p mimics	UAUGUAAUAUGGUCCACAUCUU	GAUGUGGACCAUAUUACAUAUU
hsa-miR-3149 mimics	UUUGUAUGGAUAUGUGUGUGUAU	ACACACACAUAUCCAUACAAAUU
has-miR-499a-3p mimics	AACAUCACAGCAAGUCUGUGCU	CACAGACUUGCUGUGAUGUUUU
hsa-miR-6769b-3p inhibitor	CUAUGGGUGGGACAGAGAGGG	
hsa-miR-499a-3p inhibitor	AGCACAGACUUGCUGUGAUGUU	
hsa-mimics-NC	UUCUCCGAACGUGUCACGUTT	ACGUGACACGUUCGGAGAATT
hsa-inhibitor-NC	CAGUACUUUUGUGUAGUACAA	

Appendix Table S2. Sequence of primers used for real-time PCR.

Gene	Primer (Forward)	Primer (Reverse)
METTL14	AGTGCCGACAGCATTGGTG	GGAGCAGAGGTATCATAGGAAGC
p53	CAGCACATGACGGAGGTTGT	TCATCCAAATACTCCACACGC
p21	TGTCCGTCAGAACCCATGC	AAAGTCGAAGTTCCATCGCTC
ELK1	TCCCTGCTTCCTACGCATACA	GCTGCCACTGGATGGAAACT
ALDOA	ATGCCCTACCAATATCCAGCA	GCTCCCAGTGGACTCATCTG
ALDOB	GGCAGTTCCGAGAAATCCTCT	CTCCTTGGTCTAACTTGATTCCC
ALDOC	ATGCCTCACTCGTACCCAG	TTTCCACCCCAATTTGGCTCA
ENO1	TGGTGTCTATCGAAGATCCCTT	CCTTGGCGATCCTCTTTGG
ENO2	AGCCTCTACGGGCATCTATGA	TTCTCAGTCCCATCCAACTCC
ENO3	GGCTGGTTACCCAGACAAGG	TCGTACTTCCCATTGCGATAGAA
FBP1	CGCGCACCTCTATGGCATT	TTCTTCTGACACGAGAACACAC
FBP2	ACCCGCTACGTTATGGAAAAG	GCCGTCAGCATTGAGTTCAG
G6PC	ACTGGCTCAACCTCGTCTTTA	CGGAAGTGTTGCTGTAGTAGTCA
GAPDH	GGAGCGAGATCCCTCCAAAAT	GGCTGTTGTCATACTTCTCATGG
GAPDHS	TGTGGGCATCAATGGATTTGG	ACACCATGTATTCCGGGTCAAT
GCK	CCTGGGTGGCACTAACTTCAG	TAGTCGAAGAGCATCTCAGCA
GPI	CAAGGACCGCTTCAACCACTT	CCAGGATGGGTGTGTTTGACC
HIF-1α	GAACGTCGAAAAGAAAAGTCTCG	CCTTATCAAGATGCGAACTCACA
HK1	GCTCTCCGATGAAACTCTCATAG	GGACCTTACGAATGTTGGCAA
HK2	TGCCACCAGACTAAACTAGACG	CCCGTGCCCACAATGAGAC
HK3	GGACAGGAGCACCCTCATTTC	CCTCCGAATGGCATCTCTCAG
IGF1	GCTCTTCAGTTCGTGTGTGGA	GCCTCCTTAGATCACAGCTCC
LDHA	TTGACCTACGTGGCTTGGAAG	GGTAACGGAATCGGGCTGAAT
LDHAL6B	GGTGGGAGCGAATTTCCTATG	GCTTCTCGGAAGTGAAACGC
LDHB	TGGTATGGCGTGTGCTATCAG	TTGGCGGTCACAGAATAATCTTT
LDHC	AGAACATGGTGATTCTAGTGTGC	ACAGTCCAATAGCCCAAGAGG
MPC1	ACTATGTCCGAAGCAAGGATTTC	CGCCCACTGATAATCTCTGGAG
MPC2	TACCACCGGCTCCTCGATAAA	TATCAGCCAATCCAGCACACA
MYC	TCCCTCCACTCGGAAGGAC	CTGGTGCATTTTCGGTTGTTG
PFKL	GTACCTGGCGCTGGTATCTG	CCTCTCACACATGAAGTTCTCC
PFKM	GGTGCCCGTGTCTTCTTTGT	AAGCATCATCGAAACGCTCTC
PFKP	CGCCTACCTCAACGTGGTG	ACCTCCAGAACGAAGGTCCTC
PGAM1	TCTGGAGGCGCTCCTATGAT	TCTGTGAGGTCTGCATACCTG
PGAM2	AGAAGCACCCCTACTACAACTC	TCTGGGGAACAATCTCCTCGT
PGK1	TGGACGTTAAAGGGAAGCGG	GCTCATAAGGACTACCGACTTGG
PGK2	AAACTGGATGTTAGAGGGAAGCG	GGCCGACCTAGATGACTCATAAG
PKLR	TCAAGGCCGGGATGAACATTG	CTGAGTGGGGAACCTGCAAAG
PKM	ATGTCGAAGCCCCATAGTGAA	TGGGTGGTGAATCAATGTCCA
SLC2A1	GGCCAAGAGTGTGCTAAAGAA	ACAGCGTTGATGCCAGACAG

SLC2A3	GCTGGGCATCGTTGTTGGA	GCACTTTGTAGGATAGCAGGAAG
SLC2A5	GAGGCTGACGCTTGTGCTT	CCACGTTGTACCCATACTGGA
YTHDF1	ATGACAATGACTTTGAGCCCTA	AGGGAGTAAGGAAATCCAATGG
YTHDF2	ACTTCTCAGCATGGGGAAATAA	TATTCATGCCAGGAGCCTTATT
YTHDF3	GCTCCACCAACCCAACCAGTTC	CTGAGGTCCTTGTTGCTGCTGTG
YTHDC1	AGTGACTCTGGTTCTGAATCTG	CTGGTTTGATCTTTTCGGACAG
YTHDC2	GAGAATTGGGCTGTCGTTAAAG	TGAAGCAGGATGAAATCGTACT
IGF2BP1	GATGAAGGCCATCGAAACTTTC	GGGGTGGAATATTTCGGATTTG
IGF2BP2	GATGAACAAGCTTTACATCGGG	GATTTTCCCATGCAATTCCACT
IGF2BP3	GAGGCGCTTTCAGGTAAAATAG	AATGAGGCGGGATATTTCGTAT
ACTB	AGAGCCTCGCCTTTGCCGATCC	CTGGGCCTCGTCGCCCACATA
pre-SLC2A3	CAAGGTCACTTCTCCTGCTGA	GCCAACTTCTACGCCATTCC
pre-PGAM1	CACAACCAGGCACAACTTTACA	GACCACCAACAAACTACAGCAA
pre-miR-6769b	CTTCCTGGTGGGTGGGGA	CTATGGGTGGGACAGAGAGGG
pre-miR-499a	GCGGCTGTTAAGACTTGCA	GCACAGACTTGCTGTGATGTTC
pri-miR-6769b	GCTCCAGGCTGAGCCACTT	TCCATCACCAGTACCTTCTGCC
pri-miR-499a	ACAAGGTAAGGCCCCATCTG	AGCGTAGGGACGGGAAGC

Appendix Table S3. Sequence of primers used for Chip-PCR.

Gene	Primer (Forward)	Primer (Reverse)
METTL14 Promoter #1 (-3000~-2701)	TTAGGTATTTATACTCTCGAACCTAGACA	CCAGCTACTTGGGAGGCT
METTL14 Promoter #2 (-2700~-2401)	GATTACAGGCGCCCAACAC	CACATTATAACTTATGCATCTCTCCCCA
METTL14 Promoter #3 (-2400~-2101)	GATCTCTCTTTACTTCCTTAAATTAAGTGC	TTTACAAAACTGTTGGAGTTGGGC
METTL14 Promoter #4 (-2100~-1801)	TATTACATGGAAGTACATATCTGCATCAGT	CCAGTTGGAGCAGCCCC
METTL14 Promoter #5 (-1800~-1501)	CTCTAGACTTTGTAAGGGACATAGTCA	ATGAGTTTACTGTCTAATGGAGGATTTTTC
METTL14 Promoter #6 (-1500~-1201)	AAAGAAAAACCGACTTTAGTTTTTAATCTTTG	TGAAAGACAATTTTGAACAAGTAGTCCT
METTL14 Promoter #7 (-1200~-901)	GACCTAGTTCTTCGGAAATACATTATAATTA	TATTTGAGTTATTACCATTATTATTCACCACAC
METTL14 Promoter #8 (-900~-601)	AGACTTTGGCCTTTGCTGC	CCACTCTTCCCCACACCTTAC
METTL14 Promoter #9 (-600~-301)	GCAGATGTTGTGGCTGTTACC	CCACTCTTCCCCACACCTTAC
METTL14 Promoter #10 (-300~50)	CTGTCATCTGTGGAATCAATTGAAAAG	GAGTCATAGCCACAAAATTGAGATTATG
