# Supplementary material for: METTL14 modulates glycolysis to inhibit colorectal tumorigenesis in p53‐wild‐type cells
Source: EMBO Rep. 2023 Feb 16;24(4):e56325. doi: 10.15252/embr.202256325 (PMC10074077; doi:10.15252/embr.202256325)
Supplement: Supplementary file 12 [file EMBR-24-e56325-s006.pdf]

EMBO reports

METTL14 modulates glycolysis to inhibit colorectal tumorigenesis in p53-wild type cells

Yi-Chao Hou, Xintian Zhang, Han Yao, Lidan Hou, Qingwei Zhang, Enwei Tao, Xiaoqiang Zhu, Shanshan Jiang, Yimeng Ren, Xialu Hong, Shiyuan Lu, Xiaoxu Leng, Yile Xie, Yaqi Gao, Yu Liang, Ting Zhong, Bohan Long, Jing-Yuan FANG, and Xiangjun Meng
DOI: 10.15252/embr.202256325

Corresponding author(s): Xiangjun Meng (meng_xiangjun@yahoo.com) , Jing-Yuan FANG (jingyuanfang@sjtu.edu.cn)

Review Timeline:

Submission Date:	18th Oct 22
Editorial Decision:	19th Oct 22
Revision Received:	30th Oct 22
Editorial Decision:	29th Nov 22
Revision Received:	27th Dec 22
Editorial Decision:	17th Jan 23
Revision Received:	19th Jan 23
Accepted:	27th Jan 23

Editor: Achim Breiling

Transaction Report: This manuscript was transferred to EMBO reports following peer review at The EMBO Journal.

(Note: With the exception of the correction of typographical or spelling errors that could be a source of ambiguity, letters and reports are not edited. Depending on transfer agreements, referee reports obtained elsewhere may or may not be included in this compilation. Referee reports are anonymous unless the Referee chooses to sign their reports.)

Dear Prof. Meng,

Thank you for transferring your manuscript to EMBO reports. I now went through your manuscript and the referee report from The EMBO Journal (attached below). The referee acknowledges that the revised manuscript has improved, but explanations for several of the concerns mentioned by reviewers have not been provided.

EMBO reports emphasizes novel functional over detailed mechanistic insight, but asks for strong in vivo relevance of the findings, and clear experimental support of the major conclusions. Thus, we will not require addressing points regarding more mechanistic details experimentally. However, it will be necessary that in a revised manuscript you address all points questioning the main conclusions of the study, and all technical concerns, or points regarding the experimental designs, model systems used, or data presentation.

I thus invite you to further revise your manuscript with the understanding that all remaining concerns must be addressed in the revised manuscript or in a detailed point-by-point response. Acceptance of your manuscript will depend on a positive outcome of another round of review.

Moreover, as all three referees already indicated in their original reports that the paper is presently much too long and contains redundant and confirmatory data with limited advance, I request that the manuscript is significantly cut down down, simplified and streamlined, with not more than 6 main figures.

When submitting your revised manuscript, please also carefully review the instructions that follow below.

PLEASE NOTE THAT upon resubmission revised manuscripts are subjected to an initial quality control prior to exposition to re-review. Upon failure in the initial quality control, the manuscripts are sent back to the authors, which may lead to delays. Frequent reasons for such a failure are the lack of the data availability section (please see below) and the presence of statistics based on n=2 (the authors are then asked to present scatter plots or provide more data points).

When submitting your revised manuscript, we will require:

1) a .docx formatted version of the final manuscript text (including legends for main figures, EV figures and tables), but without the figures included. Please make sure that changes are highlighted to be clearly visible. Figure legends should be compiled at the end of the manuscript text.

2) individual production quality figure files as .eps, .tif, .jpg (one file per figure), of main figures and EV figures. Please upload these as separate, individual files upon re-submission. Please make sure that all figure panels are called out separately and sequentially in the manuscript text

The Expanded View format, which will be displayed in the main HTML of the paper in a collapsible format, has replaced the Supplementary information. You can submit up to 5 images as Expanded View. Please follow the nomenclature Figure EV1, Figure EV2 etc. The figure legend for these should be included in the main manuscript document file in a section called Expanded View Figure Legends after the main Figure Legends section. Additional Supplementary material should be supplied as a single pdf file labeled Appendix. The Appendix should have page numbers and needs to include a table of content on the first page (with page numbers) and legends for all content. Please follow the nomenclature Appendix Figure Sx, Appendix Table Sx etc. throughout the text, and also label the figures and tables according to this nomenclature.

For more details please refer to our guide to authors:
http://www.embopress.org/page/journal/14693178/authorguide#manuscriptpreparation

See also our guide for figure preparation:
http://wol-prod-cdn.literatumonline.com/pb-assets/embo-site/EMBOPress_Figure_Guidelines_061115-1561436025777.pdf

See also the guidelines for figure legend preparation:
https://www.embopress.org/page/journal/14693178/authorguide#figureformat

3) a .docx formatted letter INCLUDING the reviewers' reports and your detailed point-by-point responses to their comments. As part of the EMBO Press transparent editorial process, the point-by-point response is part of the Review Process File (RPF), which will be published alongside your paper.

4) a complete author checklist, which you can download from our author guidelines (https://www.embopress.org/page/journal/14693178/authorguide). Please insert page numbers in the checklist to indicate where the requested information can be found in the manuscript. The completed author checklist will also be part of the RPF.

Please also follow our guidelines for the use of living organisms, and the respective reporting guidelines: http://www.embopress.org/page/journal/14693178/authorguide#livingorganisms

5) that primary datasets produced in this study (e.g. RNA-seq, ChIP-seq and array data) are deposited in an appropriate public database. This is now mandatory (like the COI statement). If no primary datasets have been deposited in any database, please state this in this section (e.g. 'No primary datasets have been generated and deposited').

See also: http://embor.embopress.org/authorguide#datadeposition

Please remember to provide a reviewer password if the datasets are not yet public.

The accession numbers and database should be listed in a formal "Data Availability " section (placed after Materials & Methods) that follows the model below. Please note that the Data Availability Section is restricted to new primary data that are part of this study.

Data availability

The datasets produced in this study are available in the following databases:

- RNA-Seq data: Gene Expression Omnibus GSE46843 (https://www.ncbi.nlm.nih.gov/geo/query/acc.cgi?acc=GSE46843)
- [data type]: [name of the resource] [accession number/identifier/doi] ([URL or identifiers.org/DATABASE:ACCESSION])

*** Note - All links should resolve to a page where the data can be accessed. ***

Moreover, I have these editorial requests:

6) We now request the publication of original source data with the aim of making primary data more accessible and transparent to the reader. Our source data coordinator will contact you to discuss which figure panels we would need source data for and will also provide you with helpful tips on how to upload and organize the files.

7) Our journal encourages inclusion of *data citations in the reference list* to directly cite datasets that were re-used and obtained from public databases. Data citations in the article text are distinct from normal bibliographical citations and should directly link to the database records from which the data can be accessed. In the main text, data citations are formatted as follows: "Data ref: Smith et al, 2001" or "Data ref: NCBI Sequence Read Archive PRJNA342805, 2017". In the Reference list, data citations must be labeled with "[DATASET]". A data reference must provide the database name, accession number/identifiers and a resolvable link to the landing page from which the data can be accessed at the end of the reference. Further instructions are available at: http://www.embopress.org/page/journal/14693178/authorguide#referencesformat

8) Regarding data quantification and statistics, please make sure that the number "n" for how many independent experiments were performed, their nature (biological versus technical replicates), the bars and error bars (e.g. SEM, SD) and the test used to calculate p-values is indicated in the respective figure legends (also for potential EV figures and all those in the final Appendix). Please also check that all the p-values are explained in the legend, and that these fit to those shown in the figure. Please provide statistical testing where applicable. Please avoid the phrase 'independent experiment', but clearly state if these were biological or technical replicates. Please also indicate (e.g. with n.s.) if testing was performed, but the differences are not significant. In case n=2, please show the data as separate datapoints without error bars and statistics.
See also:
http://www.embopress.org/page/journal/14693178/authorguide#statisticalanalysis

9) Please note our reference format:
http://www.embopress.org/page/journal/14693178/authorguide#referencesformat

10) We updated our journal's competing interests policy in January 2022 and request authors to consider both actual and perceived competing interests. Please review the policy https://www.embopress.org/competing-interests and add a statement declaring your competing interests. Please name that section 'Disclosure and Competing Interests Statement' and add it after the author contributions section.

11) Please order the manuscript sections like this using these names:
Title page - Abstract - Introduction - Results - Discussion - Materials and Methods - Data availability section (DAS) - Acknowledgements - Disclosure and Competing Interests Statement - References - Figure legends - Expanded View Figure legends

12) Please make sure that all the funding information is also entered into the online submission system and is complete and similar to the one in the manuscript text file (acknowledgements).

13) We now use CRediT to specify the contributions of each author in the journal submission system. CRediT replaces the author contribution section. Please use the free text box to provide more detailed descriptions. Thus, please remove the author contributions section from the manuscript text file. See also guide to authors: https://www.embopress.org/page/journal/14693178/authorguide#authorshipguidelines

I look forward to seeing a revised version of your manuscript when it is ready. Please let me know if you have questions or comments regarding the revision.

Please use this link to submit your revision: https://embor.msubmit.net/cgi-bin/main.plex

Kind regards,

Achim

Achim Breiling
Senior Editor
EMBO Reports

Referee #2:

Authors did a great effort to improve the quality off the manuscript in this revised version. However, to my view, the have failed to provide explanations for several of the concerns mentioned by reviewers in the previous submission.
I'm primarily concerned about the observation that p53-mutant or -depleted cells show reduced METLL14 levels, which may contribute to their increased tumorigenesis, but modifying METLL14 only impacts on p53 WT cells. Why is that? Does it mean that levels of METLL14 in tumors carrying p53 mutations are irrelevant, and differences in patient survival are primarily found at expenses of the p53WT subset of tumors?

When p53 mutation is linked with low METTL14 levels, being p53 a key factor in cancer progression and prognosis, the OS and DFS analysis need to be done again to test whether METLL14 is a prognosis factor inside the p53WT and MUT patients (although some analysis is included at the end of the manuscript in EV7F).
In Figure 4A, it should be crucial to know how many biological and technical replicates have been used to determine genes differentially expressed downstream of METLL14 depletion. This is an important information that should be mentioned and complemented with the analysis of inter-experimental variation (i.e. in PCA analysis). In 4B, authors study CRC datasets to confirm data from HCT116 cells but they do not consider p53 status. If METLL14 is associated with p53 status in patients, authors could be just looking at the correlation between p53 functionality and tumor metabolism. Specific analysis of WT and p53MUT tumors should considered.
In Figure 4, it is shown that upregulation of METTL14 suppresses glycolysis by down-regulating SLC2A3 and PGAM1, thus reducing the Warburg effect (production of lactate from glucose) specifically in p53-WT CRC cells. I do not see major differences in SLC2A3 in several of the experiments (i.e. 4G or 4I). What are the levels of SLC2A3 and PGAM1 in p53 MUT cells in comparison with p53 WT? Are different levels of these enzymes responsible for METLL14 insensitivity in the p53MUT background?
Also, I'm not an expert in glycolysis, but I would expect decreased ATP production (in Figure 4J) as result of a shift from mitochondrial metabolism to glycolysis, but as said I'm probably wrong.

Text corresponding to Figure 5 is again extremely dense and difficult to follow for non-experts on miRNA regulation (and probably also for experts). In brief, this section implicates METLL14 together with DGCR8 in the m6A modification of specific miRNA targeting SLC2A3 and PGAM1, and the additional involvement of the m6A reader YTHDF2. In the text it is mentioned repeatedly that all this mechanism works in p53WT cells but there is no experimental demonstration that it requires p53WT and why?

Then, authors show that maturation of miR-6769b-3p and miR-499a-3p (mediated by METLL14-induced m6A modification and targeting SLC2A3 and PGAM1) regulates glycolysis in p53WT cells lines, but I still don't see the mechanistic bases for this p53 specificity.

Finally, authors analyzed the expression levels of the different elements in relation to patient survival and determine that METLL14 is an independent prognosis factor in p53WT tumors specifically but again, all others factors are not differentially analyzed and there are no clues about p53 selectivity (i.e. maybe m6A modification is achieved by other components in p53 MUT tumors, or other glycolytic enzymes at are the base of Warburg effect in the presence of mutant p53...).

To my view, this manuscript contains a massive number of interesting and relevant results but it would need further refinement before being published in a comprehensive format.

Response to Reviewers Letter

Responses to editors' and reviewers' comments on the manuscript submitted by Hou *et al.*, "*METTL14 promotes m⁶A-dependent miRNA maturation to suppress p53-wild type colorectal cancer growth*" (EMBOR-2022-56325-T)

We appreciate the editor and reviewers for their constructive and valuable comments. We have revised our manuscript considerably according to the editor' and reviewers' comments, questions, and suggestions. In the event that we missed any one of the comments please let us know. This document includes our responses to reviewers' comments point by point:

Page

Comments from Reviewer 2...1-24

Comments from Reviewers:

General response to Reviewers: We thank the Reviewers for their careful assessment of our paper and helpful suggestions for improving our manuscript. We tried to address all your suggestions, both in terms of experiments and text. We have performed several new experiments to address the concerns of the reviewers, which has considerably improved the manuscript. We have revised the manuscript according to the reviewers' comments (in black), as indicated below in our point-by-point reply (in blue). The newly added text in the revised manuscript is highlighted in purple.

Reviewer: 2

Reviewer's comments:

General comment: Authors did a great effort to improve the quality of the manuscript in this revised version. However, to my view, the have failed to provide explanations for several of the concerns mentioned by reviewers in the previous submission.

Response:

We appreciate the reviewer's careful review of our manuscript. We fully accept the reviewer's thoughtful and professional comment and suggestion. To solve the conceptual issues, we included additional experimental evidence, in cells and mice, to strengthen the link between METTL14 and p53 and colorectal cancer (CRC) occurrence and development, and we also highlight the specific links between METTL14 and its downstream target genes and glycolysis in the context of wild-type p53 status. As suggested, we have made the following improvements: 1) to strengthen the association of METTL14 with p53-wild type (p53-WT) CRC, the correlations between METTL14 and p53 mRNA levels in p53-WT or p53 mutant (p53-MT) cells from the Cancer Cell Line Encyclopedia (CCLE) database, the differential expression of METTL14 among p53-MT and p53-MT tumor tissues from The Cancer Genome Atlas (TCGA) datasets, prognostic values of overall survival (OS) and disease-free survival (DFS) in p53-WT or p53-MT tumors from TCGA based on expression levels of METTL14, the Gene set enrichment analysis (GSEA) analysis in TCGA CRC dataset based on the median of METTL14 expression levels in p53-WT or p53-MT CRC, and especially functional significance of the p53-METTL14 axis in p53-WT or p53-MT

human tumor derived from DepMap portal, were further analyzed and the bioinformatic data that were not significantly correlated with p53-WT CRC were removed; 2) an additional independent cohort was used to investigate the differential expression and prognostic value of METTL14 and corresponding downstream gene miR-6769b-3p/miR-499a-3p, SLC2A3/PGAM1 in p53-WT and p53-MT samples; 3) in order to verify the specificity of the regulation of the p53-METTL14 axis and association between METTL14 downstream glycolytic components and wild type p53, the links between other key m^6A methyltransferase METTL3, glucose transporter SLC2A1 and phosphoglycerate mutase PGAM2 and different states of p53 were further explored; 4) to reinforce the important conceptual results that modification of METLL14 only affects p53 WT cells but not p53-MT cells, we used another p53-MT CRC cell line named SW620 to investigate the effects of METTL14 on cell proliferation, cell cycle and apoptosis of p53-MT CRC cells in vitro and vivo; 5) to define the specific relationship between METTL14 and functional downstream effectors and signaling pathways in p53-WT CRC, an RNA sequencing (RNA-seq) analysis was performed to compare the gene expression profiles of stable knockdown METTL14 group and control group in p53-MT HT29 cells once again; 6) to further test that METTL14 might function through affecting miRNAs expression, and ultimately modulating SLC2A3 and PGAM1 expression in p53-WT CRC cells, a genome-wide miRNA expression profiling was performed in p53-MT HT29 cells with stable overexpression of METTL14 and control transfectants once again; 7) to focus on the disparate role of METTL14 in p53-WT and p53-MT CRC, we removed experimental data obtained in HCT116 ($p53^{-/-}$) cells; 8) in vitro and in vivo assays were conducted to explore the effect of miR-6769b-3p/miR-499a-3p and SLC2A3/PGAM1 on p53-WT (HCT116 and Lovo) CRC cells and p53-MT (HT29 and SW620) CRC cells; 9) redo a series of experiments of western blot, real-time PCR, luciferase reporter assay, immunohistochemistry (IHC), immunofluorescence (IF), in situ hybridization (ISH), glucose consumption assay, lactate production assay, quantitative assay of ATP and pyruvate levels, and seahorse assay to prove that m^6A-YTHDF2-mediated maturation of miR-6769b-3p and miR-499a-3p regulates glycolysis in p53-WT CRC cells; 10) add some introductory descriptions according to new experimental results, explain some experimental results objectively and correctly, and reconstruct the Results sections and optimize Discussion sections of the manuscript. We would like to sincerely thank the Reviewers for their constructive comments, which we have used as the basis for revising our manuscript. We are determined to improve the quality of the manuscript as much as possible and to help guide the readers through an extensive series of new and validatory experiments. Taken together, we believe these have addressed each of the key areas of concern and significantly improved the manuscript.

Comment 1: I'm primarily concerned about the observation that p53-mutant or -depleted cells show reduced METLL14 levels, which may contribute to their increased tumorigenesis, but modifying METLL14 only impacts on p53 WT cells. Why is that? Does it mean that levels of METLL14 in tumors carrying p53 mutations are irrelevant, and differences in patient survival are primarily found at expenses of the p53WT subset of tumors?

When p53 mutation is linked with low METTL14 levels, being p53 a key factor in cancer progression and prognosis, the OS and DFS analysis need to be done again to test whether METLL14 is a prognosis factor inside the p53WT and MUT patients (although some analysis is included at the end of the manuscript in EV7F).

Response:

Thank you for your detailed review and we accept your suggestion. As suggested, our reanalysis of the CCLE database found that the METTL14 and p53 mRNA level correlated in p53-WT cancer cells but not in p53-MT cancer cells, whereas no significant correlation was present in KRAS-WT or KRAS-MT cancer cells (Fig 1G and Appendix Fig S1A in the revised manuscript) (panels A and B, Figure 1 for reviewers). Similarly, datasets from TCGA also showed that METTL14 mRNA levels in diverse tumors harboring wild type p53 are higher than those in tumors harboring p53 mutations (Fig EV1F in the revised manuscript) (panel C, Figure 1 for reviewers). These results indicate that METTL14 is differentially expressed between p53-WT and p53-MT colon cancers, which is P53-specific, since METTL14 is not differentially expressed in a similar manner between KRAS-WT and KRAS-MT colon cancers. Intriguingly, depletion of mutant p53 had no effect on METTL14 protein expression in four p53-MT CRC cell lines HT29 (p53R273H), SW620 (p53R273H), COLO320 (R248W) and SW480 (R273H) (Fig EV1G in the revised manuscript) (panel D, Figure 1 for reviewers), indicating that the levels of p53 in CRC cells carrying p53 mutations were not related to METTL14 levels. As expected, p53-WT CRC had a much higher protein level of METTL14 than p53-MT samples (Fig 1J in the revised manuscript) (panel E, Figure 1 for reviewers), while no differences were found between KRAS-WT and KRAS-MT CRC samples in Cohort 3 (Appendix Fig S3A in the revised manuscript) (panel F, Figure 1 for reviewers). In addition, we found that METTL3, another crucial m^6A methyltransferase, displays no obvious differential expression in p53-WT and p53-MT CRC tissues (Fig EV1H and Appendix Fig S3B in the revised manuscript) (panels G and H, Figure 1 for reviewers). These findings suggest that METTL14 might play a more tissue-specific role in p53-mediated tumor suppression. To further determine whether the p53-BR1 site confers p53-dependent activity, luciferase reporter assay was performed. Results showed that co-transfection of wild-type p53 specifically enhanced the transcriptional activity of reporters with intact p53-BR1, whereas mutant p53 (p53R273H and p53R175H) did not activate reporter activity in p53-null CRC cells (Fig 1M in the revised manuscript) (panel I, Figure 1 for reviewers). Virtually all p53 mutants studied to date have lost the ability to bind to DNA, thereby impairing its function as a transcription factor, and it seems likely that loss of this molecular function largely explains its role in tumor formation (Kato et al, 2003). Therefore, we figured that wild type p53 can transcriptionally activate METTL14 expression, while mutant or deficient-p53 lose its transcriptional activation ability, and thus cannot affect the expression level of METTL14, which accorded with the results that METLL14 levels in p53-MT or p53-depleted cells is lower than those in p53-WT CRC cells. In line with these findings, it is also demonstrated that METTL14 protein expression levels in p53-WT CRC tissues are higher than those in p53-MT CRC tissues.

To determinate the functional significance of the p53-METTL14 axis in human tumor,

we mined data from DepMap portal, a database comprising many perturbation datasets from hundreds of human cancer cell lines (Meyers et al, 2017). We specifically inquired the functional effect of METTL14 knockout by CRISPR-Cas9 in various human cancer cell lines. We parsed the cell lines based on p53 status into either wild type or mutant and plotted gene effect score, termed the Achilles score, that reflects the essentiality of individual genes for proliferation (Bieging-Rolett et al, 2020; Raj et al, 2022). Interestingly, the Achilles scores for METTL14 were obviously higher in p53-WT cancer cell lines than in cancer cell lines harboring p53 mutations and p53-MT cancer cell lines were less affected by METTL14 perturbation than p53-WT cancer cell lines (Fig 6D in the revised manuscript) (panel A, Figure 2 for reviewers), reminiscent of the insusceptible proliferation behavior seen in p53-MT and p53-null CRC cells upon METTL14 silencing. Moreover, the top METTL14 co-dependencies include not only p53 and the p53 positive modulators ATM and TP53BP1 as well as p53 main effector $p21^{WAF1/Cip1}$ (with Achilles score positively correlated with that of METTL14), but also p53 negative modulators MDM2 and PPM1D (with Achilles score negatively correlated with that of METTL14) in p53-WT cancer cells lines or whole cancer cell lines, whereas the significant correlations between METTL14 and p53 disappeared, and the correlations between METTL14 and regulators and effector of p53 tended to get weak in p53-MT cell lines (Fig 6E in the revised manuscript) (panel B, Figure 2 for reviewers). As expected, METTL14 and KRAS Achilles scores were not correlated in cancer cell lines among these groups (Fig 8E in the revised manuscript) (panel B, Figure 2 for reviewers). Additionally, stable lentivirus-mediated METTL14 silencing and overexpression did not affect cell growth of one other p53-MT (SW620) CRC cells compared with the respective controls in vitro and in vivo (Fig EV2B, C, D and Fig 2C in the revised manuscript) (Figure 3 for reviewers). Similarly, Mello SS et al. (Mello et al, 2017) found that Ptpn14 controlled by p53 displays tumor suppressor activity in p53-proficient cancer cells whereas did not affect colony growth in p53-deficient cells, which also suggests Ptpn14 has potent p53-dependent tumor suppression activity. We have removed experimental data obtained in HCT116 ($p53^{-/-}$) cells and added cellular function and mechanistic data related to another p53 mutant cell line named SW620, mainly to focus on the disparate role of METTL14 in p53-WT and p53-MT CRC. Above all, these functional studies thus further emphasize a growth-suppressive role for METTL14 in p53-WT cells, however, these results also suggested that there are no obvious effects of METTL14 in p53-MT or p53-deficient cells.

We then analyzed the correlation between METTL14 expression and different clinicopathological features in Cohort 3. The results demonstrated that METTL14 was inversely correlated with poor histological differentiation, AJCC III/IV stage and tumor size (≥ 30 cm^3) in p53-WT CRC patients (Fig 6H in the revised manuscript) (panel C, Figure 2 for reviewers), while no significant relation was found between METTL14 and clinicopathological features of CRC patients with p53 mutations (Appendix Fig S9A in the revised manuscript) (panel D, Figure 2 for reviewers). We next assessed the association between METTL14 and OS after tumor resection in CRC patients with diverse p53 status from Cohort 3. The analysis showed that higher levels of METTL14 predicted a better prognosis, whereas METTL14 could not serve as prognostic marker

in p53-MT CRC (Fig 6F in the revised manuscript) (panel E, Figure 2 for reviewers). Moreover, we mined data from TCGA and found that high METTL14 exhibited more favorable OS in many human cancer types, including CRC, ovarian cancer (OV), esophageal cancer (ESCA), lung adenocarcinoma (LUAD), bladder cancer (BLCA), lower grade glioma (LGG), stomach cancer (STAD), liver cancer (LIHC), endometrioid cancer (UCEC), glioblastoma (GBM), Melanoma (SKCM), kidney clear cell carcinoma (KIRC) carrying wild type p53 regardless of spectrum of p53 mutations, whereas high METTL14 has no significant positive association with OS, or even had poorer OS in some types of tumors such as head and neck cancer (HNSC), LUAD, BLCA, LGG, GBM, breast cancer (BRCA) with medium to high frequency of p53 mutations. In addition, we also found that high METTL14 was significantly associated with a more favorable disease-free survival (DFS) in some p53-WT tumors such as CRC, LUAD, UCEC, BRCA, sarcoma (SARC) and PRAD whereas high METTL14 did not confer significant favorable DFS, or even was associated with a poorer DFS in some tumors, including BLAC and STAD, with medium frequency of p53 (Fig 6G in the revised manuscript) (panel F, Figure 2 for reviewers). Finally, multivariate regression analysis revealed that METTL14 expression (HR = 0.46, 95% CI (0.22 to 0.95)) and AJCC stage were independent prognostic factors for p53-WT CRC patients in Cohort 3 (Appendix Fig S9B in the revised manuscript) (panel G, Figure 2 for reviewers), which further confirmed that METTL14 expression (HR = 0.15, 95% CI (0.04 to 0.65)) could also serve as an independent prognostic factor for p53-WT in TCGA CRC (Appendix Fig S9C in the revised manuscript) (panel H, Figure 2 for reviewers). From these observations, we conclude that elevated expression of METTL14 may herald p53-WT CRC patients with favorable prognosis.

Strikingly, our findings suggest that the wild type p53 modulated-METTL14 can be tumor suppressive in the context of wild type p53 yet displays no significant effects in p53-MT or p53-null CRC cells. Notably, METTL14 has been reported to be required for some cancer development, whereas METTL14 activation shows more tumor-suppressive activity in a variety of tumors including UCEC (Liu et al, 2018), LIHC (Ma et al, 2017), GBM (Cui et al, 2017) and skin cancer (Yang et al, 2021). Interestingly, Panneerdoss S et al. (Panneerdoss et al, 2018) revealed that METTL14 knockdown in BRCA cells (MDA-MB-231, MDA-MB-468, and BT-549) carrying diverse p53 mutations led to reduced long-term viability, migration, and invasion of BRCA cells, which, together with clinical data (Fig 6G in in the revised manuscript) (panel F, Figure 2 for reviewers) revealing a different role for METTL14 in mutant tumors support the idea that METTL14 can only employ wild type p53-dependent tumor suppressive mechanisms. This difference in METTL14 action in the context of wild type or deficient p53 also provides one potential explanation for observed differences in the role of the METTL14 in cancer development. As suggested, we redescribed the new data in the **Results** section, and we also rewrote it in the **Discussion** section as follows "Strikingly, our findings suggest that the wild type p53 modulated-METTL14 can be tumor suppressive in the context of wild type p53 yet displays no significant effects in p53-MT or p53-null CRC cells. Notably, METTL14 has been reported to be required for some cancer development, whereas METTL14 activation shows more tumor-

suppressive activity in a variety of tumors including UCEC (Liu et al, 2018), LIHC (Ma et al, 2017), GBM (Cui et al, 2017) and skin cancer (Yang et al, 2021). Interestingly, Panneerdoss S et al. (Panneerdoss et al, 2018) revealed that METTL14 knockdown in BRCA cells (MDA-MB-231, MDA-MB-468, and BT-549) carrying diverse p53 mutations led to reduced long-term viability, migration, and invasion of BRCA cells, which, together with clinical data (Fig 8G) revealing a different role for METTL14 in mutant tumors support the idea that METTL14 can only employ wild type p53-dependent tumor suppressive mechanisms. This difference in METTL14 action in the context of wild type or deficient p53 also provides one potential explanation for observed differences in the role of the METTL14 in cancer development. Our data suggest that p53 pathway status is one factor that dictates whether METTL14 is a tumor suppressor or non-function gene, even functions as an oncogene.” (L488-503 in the revised manuscript)

Figure 1 for reviewers

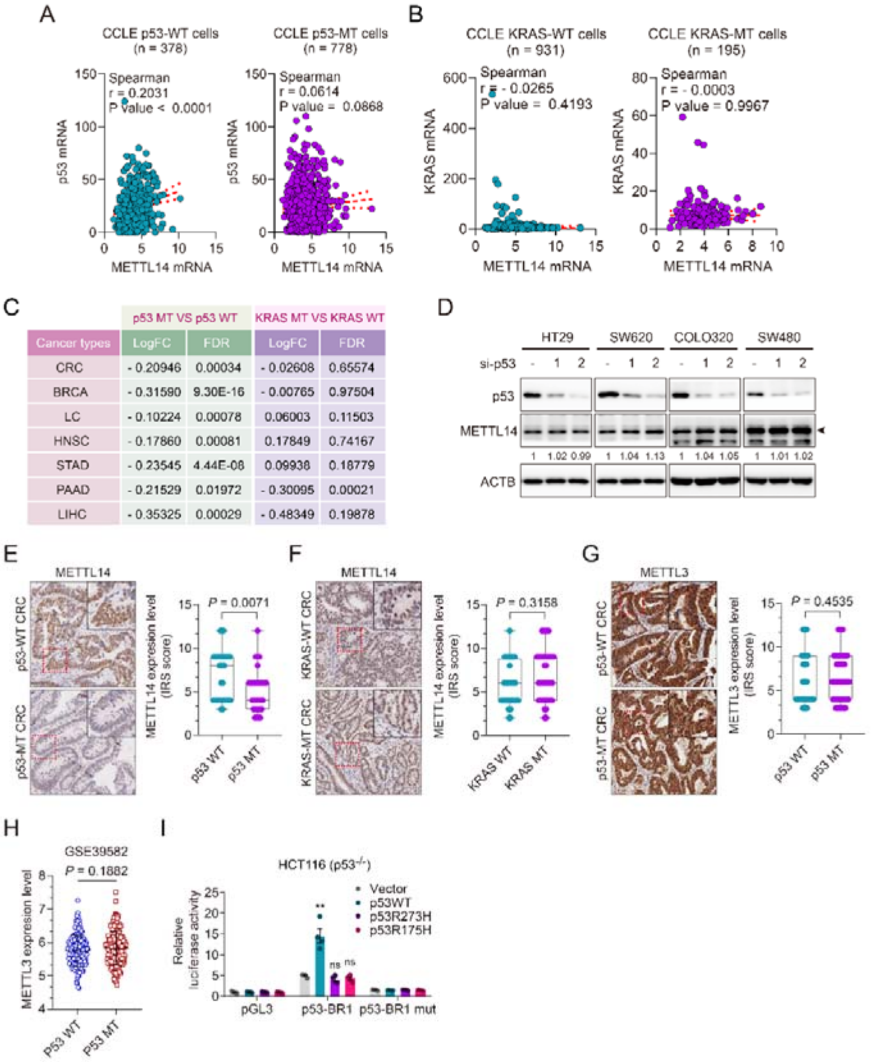

Figure 2 for reviewers

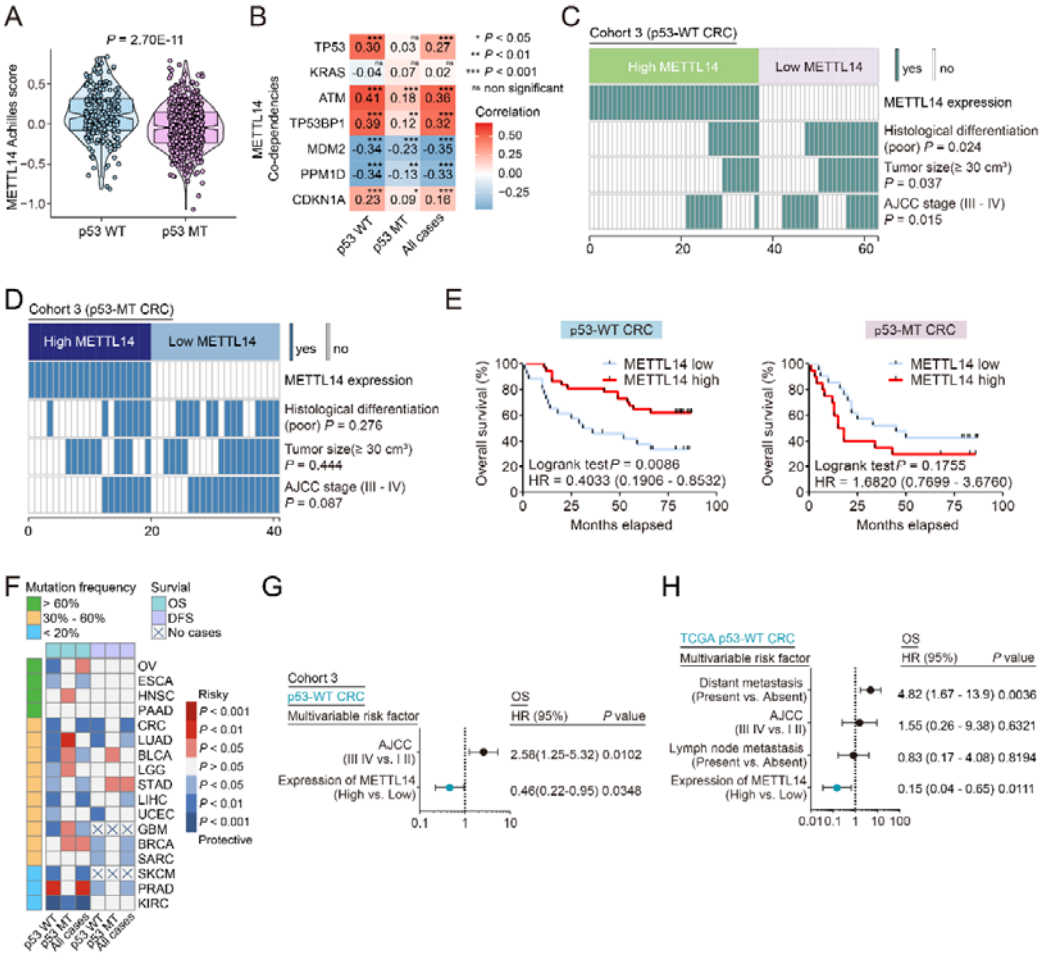

Figure 3 for reviewers

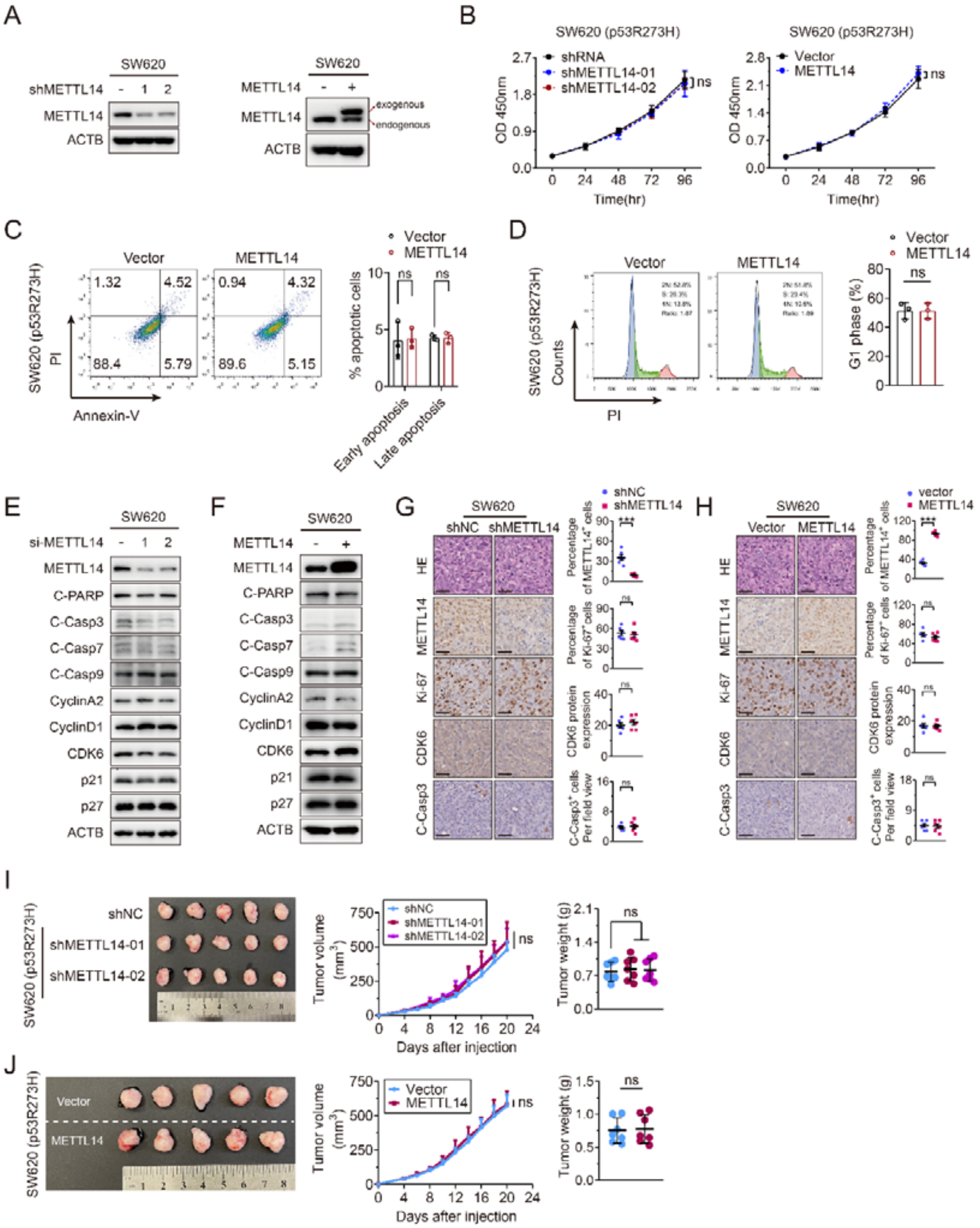

Comment 2: In Figure 4A, it should be crucial to know how many biological and technical replicates have been used to determine genes differentially expressed downstream of METLL14 depletion. This is an important information that should be mentioned and complemented with the analysis of inter-experimental variation (i.e. in PCA analysis). In 4B, authors study CRC datasets to confirm data from HCT116 cells but they do not consider p53 status. If METLL14 is associated with p53 status in patients, authors could be just looking at the correlation between p53 functionality and tumor metabolism. Specific analysis of WT and p53MUT tumors should considered.

Response:

Thank you very much for your rigorous comments and insightful suggestion. As suggested, principal component analysis was performed to reveal that control and shMETTL14 stable expressing samples clearly segregated in p53-WT (HCT116) cells (Fig EV3A in the revised manuscript) (panel A, Figure 4 for reviewers). There are four technical replicates in control or shMETTL14 group, respectively. In shMETTL14 group, there were two equal numbers of biological replicates named shMETTL14-01 and shMETTL14-02, which showed a close association in principal component analysis. To especially further gain insight into the functional downstream effectors and signaling pathways mediated by METTL14, an RNA sequencing (RNA-seq) analysis was again performed to compare the gene expression profiles of stable knockdown METTL14 group and control group in p53-MT (HT29) CRC cells. Firstly, the control and METTL14 knockdown samples also clearly segregated, with shMETTL14-01 and shMETTL14-02 samples showing a close association, as expected (Fig EV3A in the revised manuscript) (panel A, Figure 4 for reviewers). Then, differentially expressed genes (P < 0.05) were detected (raw data accessible via GSE210056) after stable knockdown of METTL14 in p53-MT CRC cells (Appendix Table S3). Notably, the gene set enrichment analysis (GSEA) showed that no significant correlation or even opposite trend between METTL14 knockdown and the signatures was identified in p53-MT CRC cells (Fig EV3C in the revised manuscript) (panel B, Figure 4 for reviewers). We are totally agreed with your thoughtful and professional suggestion that specific analysis of p53-WT and p53-MT CRC should be considered now that METLL14 is associated with p53 status in CRC patients. Then, we performed the GSEA analysis based on the median of METTL14 expression levels in p53-WT or p53-MT CRC from TCGA CRC dataset. Biological process analysis showed that cell proliferative signatures (Go_Regulation_Of_Epithelial_Cell_Proliferation and Go_Epithelial_Cell_Proliferation), apoptotic signatures (Kegg_Apoptosis, Reactome_Apoptosis and Hallmapk_Apoptosis), cell cycle regulatory signatures (Go_Regulation_Of_Cell_Cycle_Arrest, Go_Positive_Regulation_Of_Cell_Cycle_Arrest and Go_Negative_Regulation_Of_Cell_Cycle_Phase_Transition), signatures of CRC occurrence (Grade_Colon_and_Rectal_Cancer_Dn and Grade_Colon_Cancer_Dn) and glycolytic signatures (Hallmapk_Glycolysis, Kegg_Glycolysis_Gluconeogenesis and Go_Pyruvate_Metabolic_Process) were significantly enriched in p53-WT CRC with high METTL14 expression (Fig 3A in the revised manuscript) (panel C, Figure 4 for reviewers). Remarkably, no statistically significant association with above-mentioned biological process was identified based on the median of METTL14 expression in patients with p53-MT CRC (Fig 3A in the revised manuscript) (panel C, Figure 4 for reviewers). The gene sets were visualized as interaction networks with the Cytoscape based on the median of METTL14 expression levels in p53-WT CRC. The results showed that the gene signatures of p53 signals-related cell cycle, cell proliferation and apoptosis, and pathways in cancer as well as glycolysis were enriched in p53-WT CRC (panel D, Figure 4 for reviewers). Strikingly, no significant differences in alteration of ECAR and OCR were identified in another p53-MT (SW620) cell line with METTL14 overexpression (Fig EV3G in the revised manuscript) (panel E, Figure 4 for reviewers).

In p53-MT CRC cells, however, METTL14 displays no significant effect on SLC2A3 and PGAM1 expression, or even has a negative impact on many glycolysis-associated genes (SLC2A1, HK1, GPI, PFKP, ALDOA, GAPDH, PGK1, ENO2, PKM, LDHA and LDHB) in p53-MT CRC cells via heat-map clustering analysis, echoing the results of the GSEA analysis (Fig 3E in the revised manuscript and Dataset EV1) (panels F and G, Figure 4 for reviewers). Importantly, no significant differences in the expression level of SLC2A3 and PGAM1 were identified in p53-MT (SW620) cells in response to METTL14 overexpression or silencing (panels H and I, Figure 4 for reviewers). In summary, these data suggest that METTL14 might be related to wild type p53 and serves as a key modulator of glycolytic metabolism to affect p53-WT CRC tumorigenesis. We rewrote it in the **Results** section as follows "The Gene set enrichment analysis (GSEA) showed that cell proliferative signatures, apoptotic signatures, cell cycle regulatory signatures, signatures of CRC occurrence and glycolytic signatures were significantly enriched in p53-WT CRC but not p53-MT with high METTL14 expression (Fig 3A). An RNA sequencing (RNA-seq) analysis was performed to compare the gene expression profiles of stable knockdown METTL14 group and control group in p53-WT and p53-MT cells. Firstly, principal component analysis revealed that control and METTL14 knockdown-treated samples clearly segregated, as expected (Fig EV3A). GSEA showed that besides the gene sets related to cell proliferation, cell cycle, apoptosis and CRC-specific signature, gene sets related to glycolysis were enriched in METTL14 knockdown group in p53-WT CRC cells (Fig 3B and EV3B). No significant correlation or even opposite trend between METTL14 knockdown and the above mentioned signatures was identified in p53-MT cells (Fig EV3C). These data suggest that METTL14 might be related to wild type p53 and serve as a key modulator of glycolytic metabolism to affect p53-WT CRC tumorigenesis." (L228-241 in the revised manuscript)

Figure 4 for reviewers

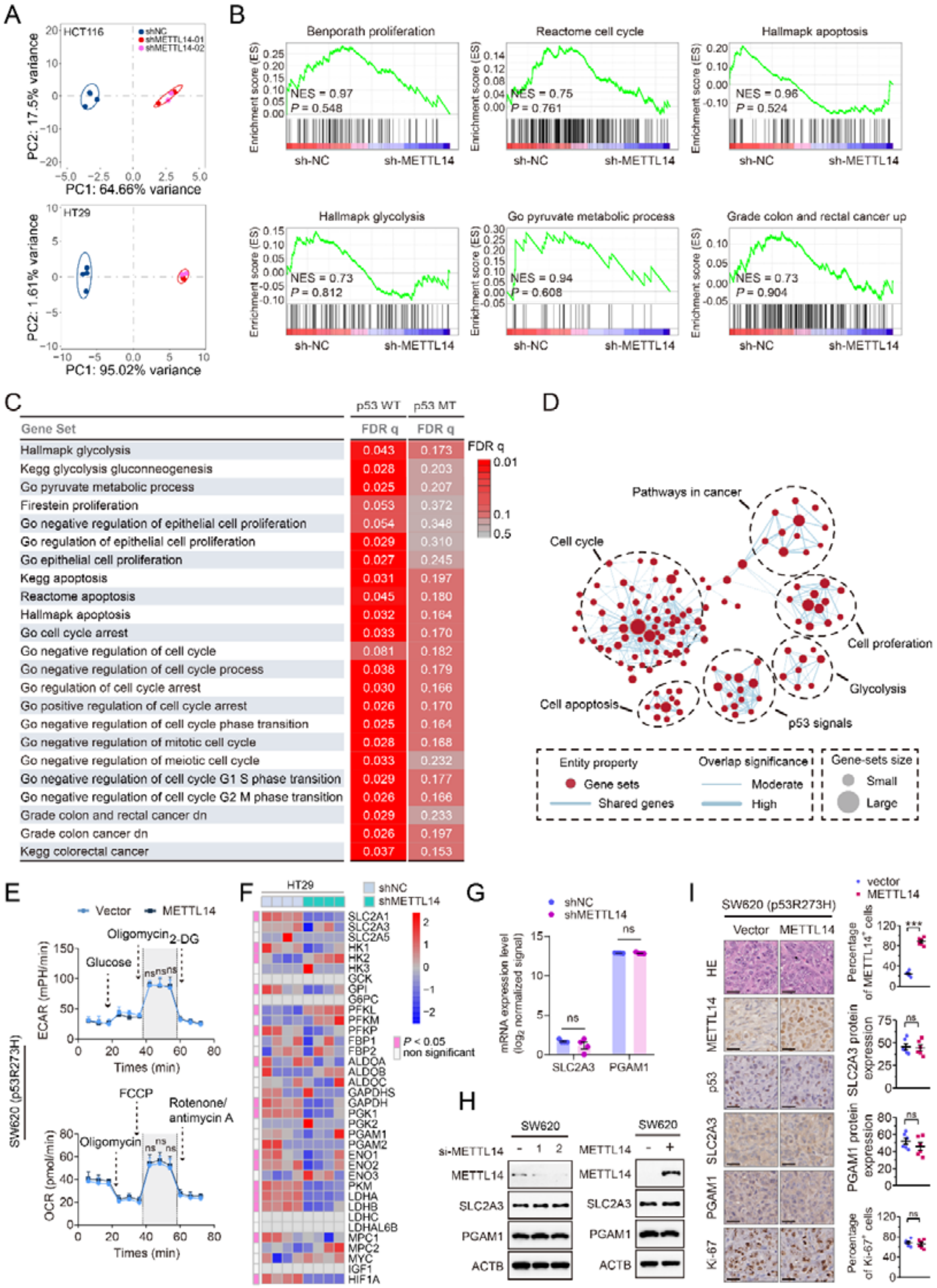

Comment 3: In Figure 4, it is shown that upregulation of METTL14 suppresses glycolysis by down-regulating SLC2A3 and PGAM1, thus reducing the Warburg effect (production of lactate from glucose) specifically in p53-WT CRC cells. I do not see major differences in SLC2A3 in several of the experiments (i.e. 4G or 4I). What are the levels of SLC2A3 and PGAM1 in p53 MUT cells in comparison with p53 WT? Are different levels of these enzymes responsible for METLL14 insensitivity in the

p53MUT background?

Response:

Thank you for your rigorous comments. We purchase new SLC2A3 antibodies (20403-1-AP, Proteintech) (Cho et al, 2015) suitable for use in mouse tissues. As suggested, we redone IHC assay and presented representative images and corresponding quantitative analysis. Consistently, METTL14 depletion increased the expression of SLC2A3 ($P <$ 0.001), as assessed by IHC staining in matched histological sections during carcinogen-induced two CRC models (Fig 3I and Appendix Fig S4 in the revised manuscript) (panel A, Figure 5 for reviewers). As suggested, we assessed the expression of SLC2A3 and PGAM1 in multiple CRC cell lines, and found that expression level of SLC2A3/PGAM1 was lower in p53-WT CRC cell line than in CRC cell line with a p53 mutation (Fig EV5H in the revised manuscript) (panel B, Figure 5 for reviewers). In contrast, miR-6769b-3p/miR-499a-3p expression levels were higher in the p53-WT CRC cell line than in the p53-MT CRC cell line (Fig EV5I in the revised manuscript) (panel C, Figure 5 for reviewers). Importantly, we found that higher miR-6769b-3p/miR-499a-3p tended to have lower SLC2A3/PGAM1 in p53-WT cell lines, whereas no such tendency was detected in p53-MT CRC cell line. Then, we tested whether SLC2A3 and PGAM1 played a role in METTL14/miR-6769b-3p and METTL14/miR-499a-3p axis-mediated regulation of p53-WT tumor growth. Results demonstrated that SLC2A3/PGAM1 down-regulation suppressed the proliferation of p53-WT CRC cells, while imposed no obvious effects on cell growth in p53-MT CRC cells (Fig EV5G in the revised manuscript) (panels D and E, Figure 5 for reviewers). Given glycolysis-related genes SLC2A3/PGAM1, as validated target genes of miR-6769b-3p/miR-499a-3p, were also directly or indirectly regulated by METTL14, and as pointed out by the reviewer, we also wondered that whether different levels of glucose transporter SLC2A3 and glycolytic enzyme PGAM1 were responsible for METLL14 insensitivity in the p53-MT background. We then extended these in vitro findings to xenograft tumors using p53-WT (HCT116 and Lovo) and p53-MT (HT29 and SW620) cell lines. The stable knockdown of SLC2A3/PGAM1 in CRC cells demonstrated significant reduction in tumor volume and weight derived from p53-WT cells compared with those from control-treated cells, whereas the p53-MT xenograft did not respond to stable knockdown of SLC2A3/PGAM1 (Fig EV5J and EV5K in the revised manuscript) (panels F and G, Figure 5 for reviewers). These results indicate that miR-6769b-3p/miR-499a-3p target genes SLC2A3/PGAM1 modulate CRC cells growth, which is also dependent on wild type p53, and different levels of SLC2A3/PGAM might be responsible for METLL14 insensitivity in the p53-MT background. As suggested, we added elaborate explanations on new data into the **Results** section in revised manuscript.

Figure 5 for reviewers

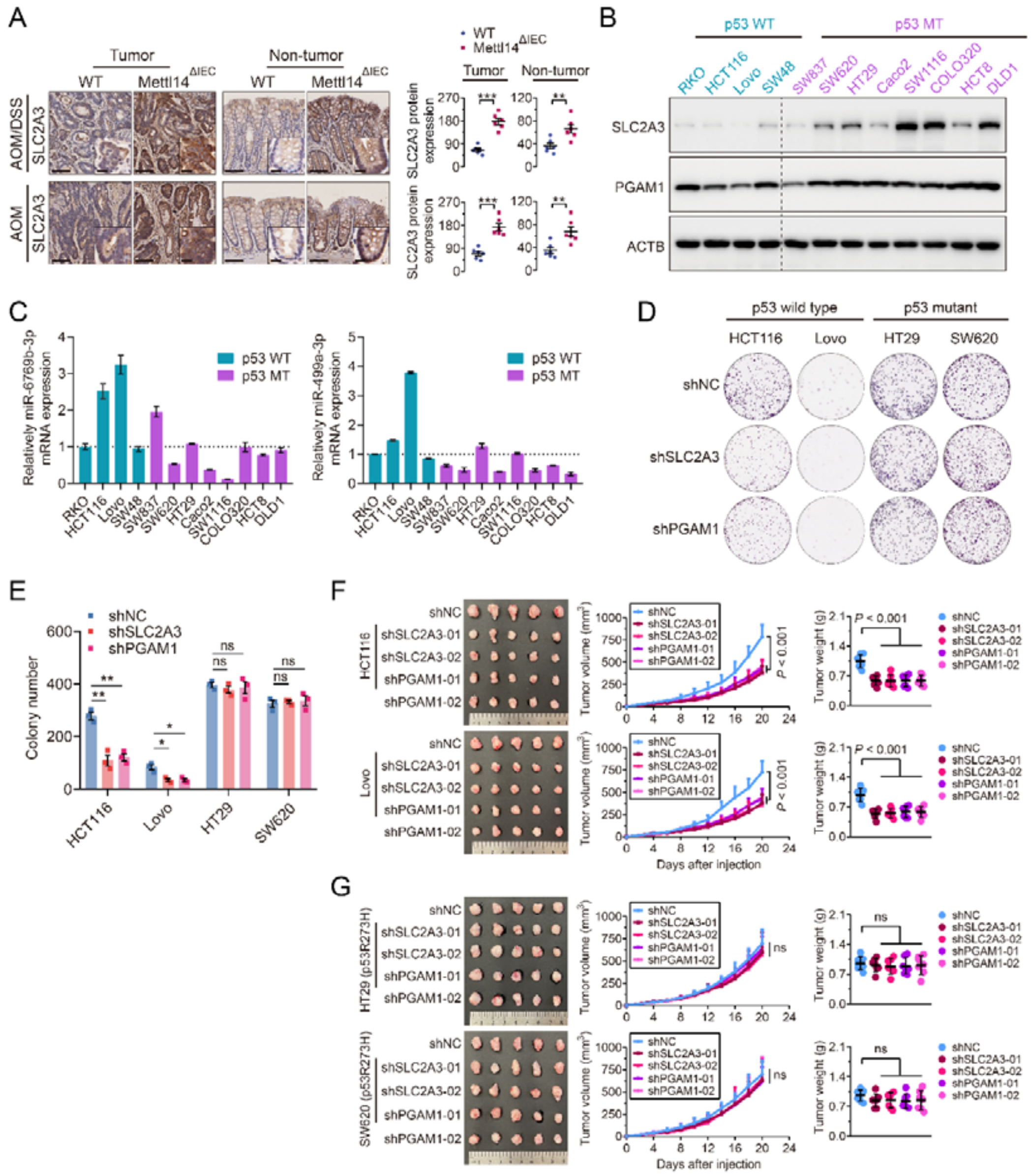

Major points 4: Also, I'm not an expert in glycolysis, but I would expect decreased ATP production (in Figure 4J) as result of a shift from mitochondrial metabolism to glycolysis, but as said I'm probably wrong.

Response:

Thank you for your rigorous comments. In the 1920s, a seminal finding by Otto Warburg showed that cancer cells usually prefer glycolysis over mitochondrial respiration for ATP generation despite a prevailing aerobic condition (Eniafe & Jiang, 2021). Glycolysis is an anaerobic oxidative process because it occurs in the absence of free oxygen, and there is a loss of hydrogen. In this process, one molecule of glucose is broken down into two molecules of pyruvic acid. In this process, two molecules of ATP are used to produce four molecules of ATP. This process takes place in two phases: 1) Preparatory or Energy Investment Phase- In this phase, glucose is converted to

glyceraldehyde-33-phosphate, and energy or ATP is consumed; 2) Pay-off or Energy Harvesting Phase- In this phase, triose phosphates are converted to pyruvate, and energy or ATP synthesized (DeBerardinis et al, 2008; Vander Heiden et al, 2009). In present study, we identified that METTL14 affects glycolysis signaling by RNA-seq, bioinformatics analysis and function assay. The results showed that upregulation of METTL14 suppresses glucose consumption, lactate production, ATP and pyruvate levels by down-regulating SLC2A3 and PGAM1, thus impeding Warburg effect of p53-WT CRC cells, sequentially attenuating CRC tumorigenesis. Additionally, Li et al. (Li et al, 2018) found that tumor suppressor gene miR-548a-3p reduces glucose uptake, pyruvate level, lactate production, and ATP level in ZR75-1 and HepG2 cells, and miR-548a-3p overexpression also displays decreased extracellular acidification rate (ECAR), which reflects overall glycolytic flux, and increased oxygen consumption rate (OCR), an indicator of mitochondrial oxidative respiration. Similarly, tumor suppressor gene miR-30d suppresses lactate secretion, glucose uptake and intracellular ATP levels (Hou et al, 2021). Bian et al. (Bian et al, 2017) found that overexpression of Nur77 in Huh7 cells not only reduces glucose uptake and lactate excretion but also suppresses ATP level. Fig 3J coincided with these results, which indicates that intestinal epithelial cells (IECs) isolated from specific METTL14 knockout (Mettl14$^{\Delta IEC}$) mice possessed higher ATP level, lactate production and pyruvate level than Mettl14^{WT} mice.

Major points 5: Text corresponding to Figure 5 is again extremely dense and difficult to follow for non-experts on miRNA regulation (and probably also for experts). In brief, this section implicates METLL14 together with DGCR8 in the m6A modification of specific miRNA targeting SLC2A3 and PGAM1, and the additional involvement of the m6A reader YTHDF2. In the text it is mentioned repeatedly that all this mechanism works in p53WT cells but there is no experimental demonstration that it requires p53WT and why?

Response:

We accept your thoughtful and professional comments and suggestions. As reviewer noted, text corresponding to Figure 5 is really dense. In order to improve the quality and readability of this paper, we have deleted some less important data. In new Figure 4 correspond to previous Figure 5, we represented the findings that m^{6}A-induced miR-6769b-3p and miR-499a-3p biogenesis via YTHDF2-mediated RNA stability regulation. For details, please refer to the revised manuscript.

As reviewer noted, confirmatory experiments should be conducted to demonstrate that m^{6}A-induced miR-6769b-3p and miR-499a-3p biogenesis via YTHDF2-mediated RNA stability regulation rely on wild type p53. We then determine whether METTL14 affects the expression of miR-6769b-3p/miR-499a-3p and pri-miR-6769b/pri-miR-499a in p53-MT CRC cells (HT29 and SW620). As expect, METTL14 did not modulate the expression of miR-6769b-3p/miR-499a-3p and pri-miR-6769b/pri-miR-499a in p53-MT CRC cells (Fig EV4H in the revised manuscript) (panel A, Figure 6 for reviewers). Moreover, METTL14 and YTHDF2 imposed no effect on pri-miR-6769b and pri-miR-499a stability (panels B-D, Figure 6 for reviewers), indicating that METTL14 might regulate the miRNA biogenesis by YTHDF2-mediated RNA stability regulation in the context of wild-type p53 status. Since miR-6769b-3p and miR-499a-

3p were downstream target genes of METTL14, we wondered whether miR-6769b-3p and miR-499a-3p affects the growth of CRC cells dependent on wild-type p53. To test this hypothesis, we overexpressed miR-6769b-3p and miR-499a-3p, and examined the proliferation of p53-MT CRC cells. Similar to phenotype that the effects of METTL14 were dependent on wild-type p53, miR-6769b-3p/miR-499a-3p had no significant effects on cell proliferation of p53-MT HT29 and SW620 cells (panels E and F, Figure 6 for reviewers). Consistent with these data in vitro, miR-6769b-3p/miR-499a-3p up-regulation suppressed p53-WT CRC cells-formative tumor growth in vivo (Fig 4N in the revised manuscript) (panel G, Figure 6 for reviewers), while xenograft tumors from p53-MT CRC cells expressing control or miR-6769b-3p/miR-499a-3p exhibited no significant differences (Fig 4O in the revised manuscript) (panel H, Figure 6 for reviewers). These results indicated that the regulatory effect of miR-6769b-3p/miR-499a-3p, the downstream target genes of METTL14, on the tumorigenesis of CRC is dependent on the wild type status of p53.

Figure 6 for reviewers

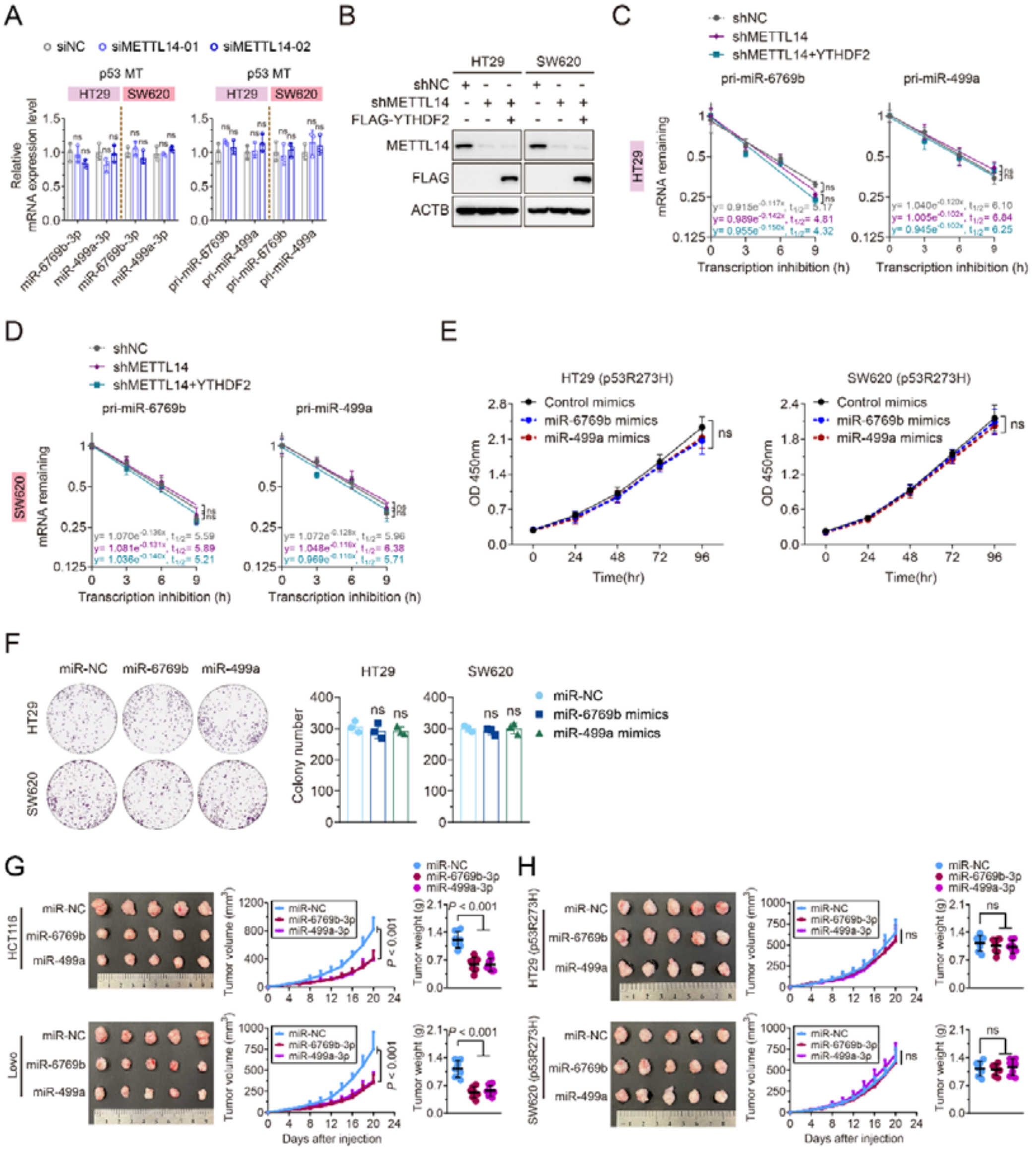

Major points 6: Then, authors show that maturation of miR-6769b-3p and miR-499a-3p (mediated by METLL14-induced m6A modification and targeting SLC2A3 and PGAM1) regulates glycolysis in p53WT cells lines, but I still don't see the mechanistic bases for this p53 specificity.

Response:

Thank you for your insightful and rigorous comments. To further make clear of the roles of METTL14 in miR-6769b-3p/miR-499a-3p regulation, a genome-wide miRNA expression profiling was again employed in p53-MT HT29 cells with stable overexpression of METTL14 and control transfectants. Differentially expressed miRNAs ($P < 0.05$) were detected (raw data accessible via GSE210056) after stable overexpression of METTL14 in p53-MT CRC cells (Dataset EV2 in the revised manuscript). The results differ noticeably from the observations made for the p53-WT cells, no significant relation between METTL14 and glycolytic signaling was identified

in p53-MT cells (Fig 4C in the revised manuscript) (panel A, Figure 7 for reviewers). Importantly, we found that METTL14 could not modulate miR-6769b-3p and miR-499a-3p expression in p53-MT HT29 cells by miRNA microarrays (Fig EV4C in the revised manuscript) (panel B, Figure 7 for reviewers). Moreover, real-time PCR results support that METTL14 did not regulate the expression of miR-6769b-3p and miR-499a-3p in p53-MT HT29 cells (Fig EV4D in the revised manuscript) (panel C, Figure 7 for reviewers). Consistently, miR-6769b-3p and miR-499a-3p display no significant impact on SLC2A3 and PGAM1 protein level in p53-MT HT29 and SW620 cells (Fig EV4E in the revised manuscript) (panels D and E, Figure 7 for reviewers), which was also verified by IF assay (Fig 4G in the revised manuscript) (panel F, Figure 7 for reviewers). Moreover, miR-6769b-3p and miR-499a-3p had no obvious effects on WT or MT reporter activity of SLC2A3 and PGAM1 3'UTR (panel G, Figure 7 for reviewers). Therefore, these results suggest regulatory effects of SLC3A3 and PGAM1 separately by miR-6769b-3p and miR-499a-3p required wild-type p53, thus linking miR-6769b-3p/miR-499a-3p-mediated Warburg effects to a wild type p53-dependent mechanism. The above results led us to hypothesize that miR-6769b-3p and miR-499a-3p may regulate METTL14-controlled glycolysis reprogramming dependent on wild type p53. As expect, miR-6769b-3p and miR-499a-3p did not affect metabolic phenotype in p53-MT CRC cells (Fig EV5A-D in the revised manuscript) (panels H-O, Figure 7 for reviewers), indicating that the METTL14 target genes miR-6769b-3p and miR-499a-3p also have potent p53-dependent regulatory effects on glycolysis in cancer cells. Therefore, these results suggest regulatory effects of SLC3A3 and PGAM1 separately by miR-6769b-3p and miR-499a-3p required wild-type p53, thus linking miR-6769b-3p/miR-499a-3p-mediated Warburg effects to a wild type p53-dependent mechanism.

Figure 7 for reviewers

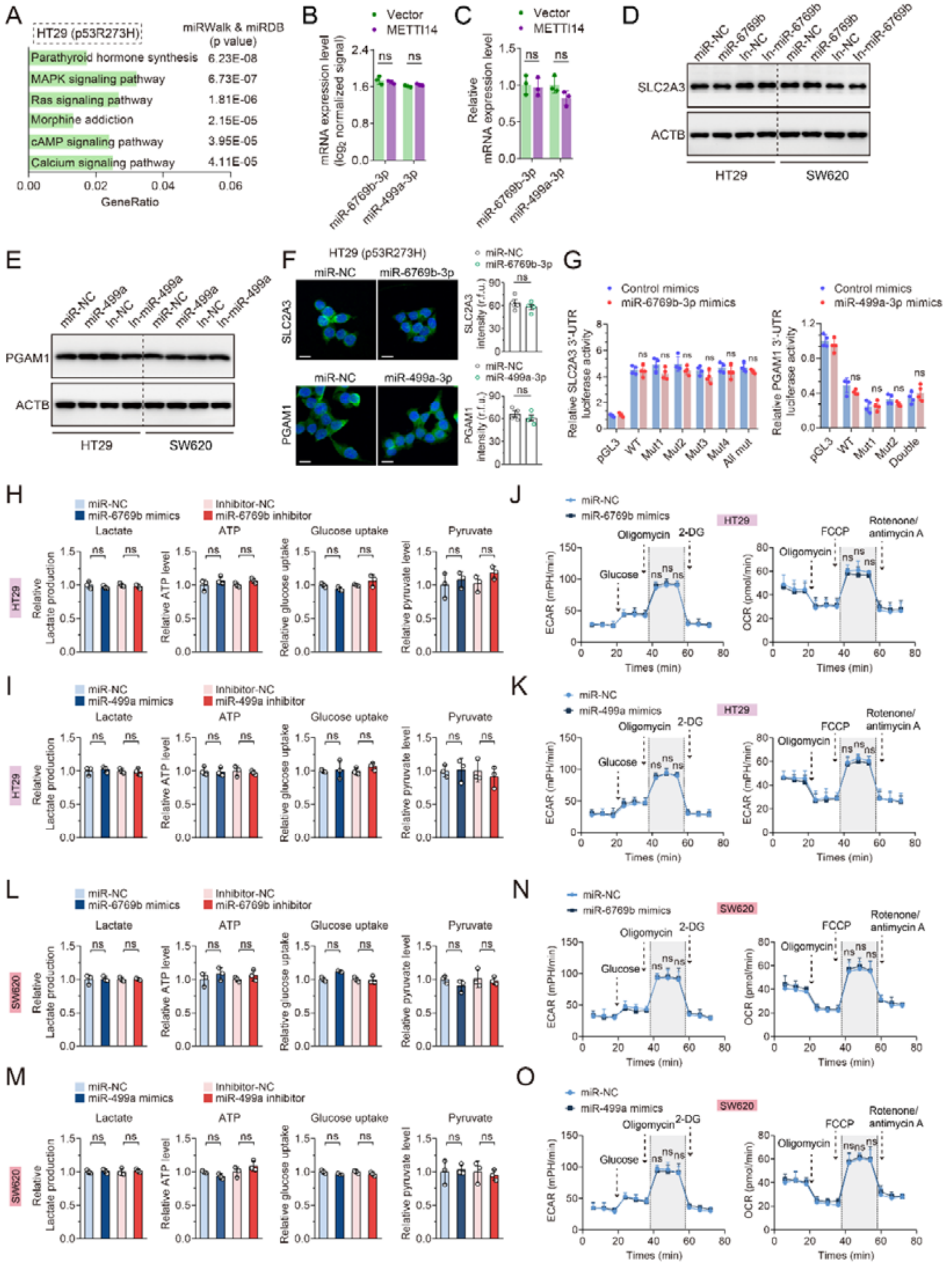

Major points 7: Finally, authors analyzed the expression levels of the different elements in relation to patient survival and determine that METLL14 is an independent prognosis factor in p53WT tumors specifically but again, all others factors are not differentially analyzed and there are no clues about p53 selectivity (i.e. maybe m6A modification is achieved by other components in p53 MUT tumors, or other glycolytic enzymes at are the base of Warburg effect in the presence of mutant p53...).

Response:

Thank you for your detailed review and we totally agree with you. As suggested, we investigated the clinical implications of miR-6769b-3p/SLC2A3 and miR-499a-3p/PGAM1 in patients with CRC harboring wild type p53 and p53 mutations, respectively. Biopsies from an additional independent cohort confirmed that p53-WT CRC had much higher levels of miR-6769b-3p/miR-499a-3p, much lower levels of SLC2A3/PGAM1 than p53-MT samples (Fig 6A in the revised manuscript) (panel A, Figure 8 for reviewers). Furthermore, the samples with high expression of METTL14 displayed strong staining for miR-6769b-3p and miR-499a-3p, weak staining for SLC2A3 and PGAM1, while samples with low expression of METTL14 were with low levels of miR-6769b-3p and miR-499a-3p, and high levels of SLC2A3 and PGAM1 in p53-WT CRC samples (Fig 6B and 6C in the revised manuscript) (panels B and C, Figure 8 for reviewers). However, miR-6769b-3p/miR-499a-3p, SLC2A3/PGAM1 did not show correlation with METTL14 expression in p53-MT CRC samples, and levels of miR-6769b-3p/miR-499a-3p and SLC2A3/PGAM1 were also not correlated in p53-MT CRC samples (Fig EV9F in the revised manuscript) (panels D and E, Figure 8 for reviewers), indicating that activation of miR-6769b-3p/miR-499a-3p and subsequent inhibition of SLC2A3/PGAM1 expression via METTL14 might occur in the background of wild type p53. We next assessed the association between miR-6769b-3p/miR-499a-3p, glycolysis components SLC2A3/PGAM1 and OS after tumor resection in CRC patients with diverse p53 status from Cohort 3. The analysis showed that higher levels of miR-6769b-3p and miR-499a-3p predicted better prognosis, but elevated expression of SLC2A3 and PGAM1 exhibited robustly shorter OS in patients with p53-WT CRC (Fig EV9G in the revised manuscript) (panel B, Figure 9 for reviewers). However, METTL14, miR-6769b-3p/miR-499a-3p, SLC2A3/PGAM1 could not serve as independent prognostic markers in p53-MT CRC (Appendix Fig S8B in the revised manuscript) (panel B, Figure 9 for reviewers). These results suggest that activation of miR-6769b-3p/miR-499a-3p and subsequent inhibition of SLC2A3/PGAM1 expression via METTL14 might occur in the background of wild type p53.

METTL3 is one of the earliest identified m^{6}A methyltransferases that regulate the functions of m^{6}A. A large number of studies have shown that METTL3 establishes a cross-talk with tumor cells and development of various human diseases (Deng et al, 2018). Chen et al. (Chen et al, 2021) demonstrated that METTL3 induces SLC2A1 translation in an m^{6}A-dependent manner, which subsequently promotes glucose uptake and lactate production, leading to the activation of mTORC1 signaling and CRC development. Among their findings, they identified that METTL3 knockdown reduces CRC cell proliferation and colony formation, promotes G1-phase cell cycle arrest and cell apoptosis in three different CRC cells, including HCT116, DLD1 and SW480 harboring wild type or mutant p53. Notably, they did not mention whether METTL3 affects the occurrence of CRC by accelerating glucose metabolism depending on the status of p53. As expected, we found that METTL3, as another crucial m^{6}A methyltransferase, displays no obvious differential expression in p53-WT and p53-MT CRC tissues (Fig EV1H and Appendix Fig S3B in the revised manuscript) (panel G and H, Figure 1 for reviewers). Additionally, METTL3 display indiscriminately prognostic

value in p53-WT and p53-MT CRC (Appendix Fig S8B in the revised manuscript) (panel B, Figure 9 for reviewers). These results suggest that other m^{6}A RNA-methyltransferase components such as METTL3 might affect the occurrence and development of CRC independent of p53 status.

Glucose transporter 1 (GLUT1), the uniporter protein encoded by the SLC2A1 gene, is a key rate-limiting factor in the transport of glucose in cancer cells, and frequently expressed in a significant proportion of human cancers. Numerous studies have reported paradoxical evidence of the relationship between SLC2A1 expression and prognosis in solid human tumors. Shen et al. (Shen et al, 2020) found that downregulation of HK2 or SLC2A1 dramatically impairs METTL3-induced cell proliferation and colony formation in two different CRC cells, including HCT116 and DLD1 harboring wild type p53 or mutant p53. In addition, PGAM2, as another important phosphoglycerate mutase, is abundant in several types of tissues and malignant tumors. To further query the correlation between glycolysis and p53 status, we performed IHC staining to assess the expression levels of another glucose transporter SLC2A1 and phosphoglycerate mutase PGAM2 in human CRC. Our results showed that the SLC2A1 and PGAM2 IHC signals did not differ between the two groups (Appendix Fig S6 in the revised manuscript) (panel A, Figure 9 for reviewers), indicating that other glycolytic genes apart from SLC2A3 and PGAM1 perform Warburg effects in the presence of mutant p53. Additionally, SLC2A1 and PGAM2 display indiscriminately prognostic value in p53-WT and p53-MT CRC (Appendix Fig S8B in the revised manuscript) (panel B, Figure 9 for reviewers). These results suggest that other glucose transporters or glycolytic enzymes such as SLC2A1 and PGAM2 might affect the occurrence and development of CRC by modulating Warburg effect independent of p53 status. In general, METTL14 is an independent predictor of beneficial prognosis in p53-WT CRC patients and functions as a tumor suppressor by selectively maturating miR-6769b-3p and miR-499a-3p, and then inhibiting the expression of SLC2A3 and PGAM1, and rewiring the cellular metabolism to reduce glycolysis and repress p53-WT CRC tumorigenesis.

Figure 8 for reviewers

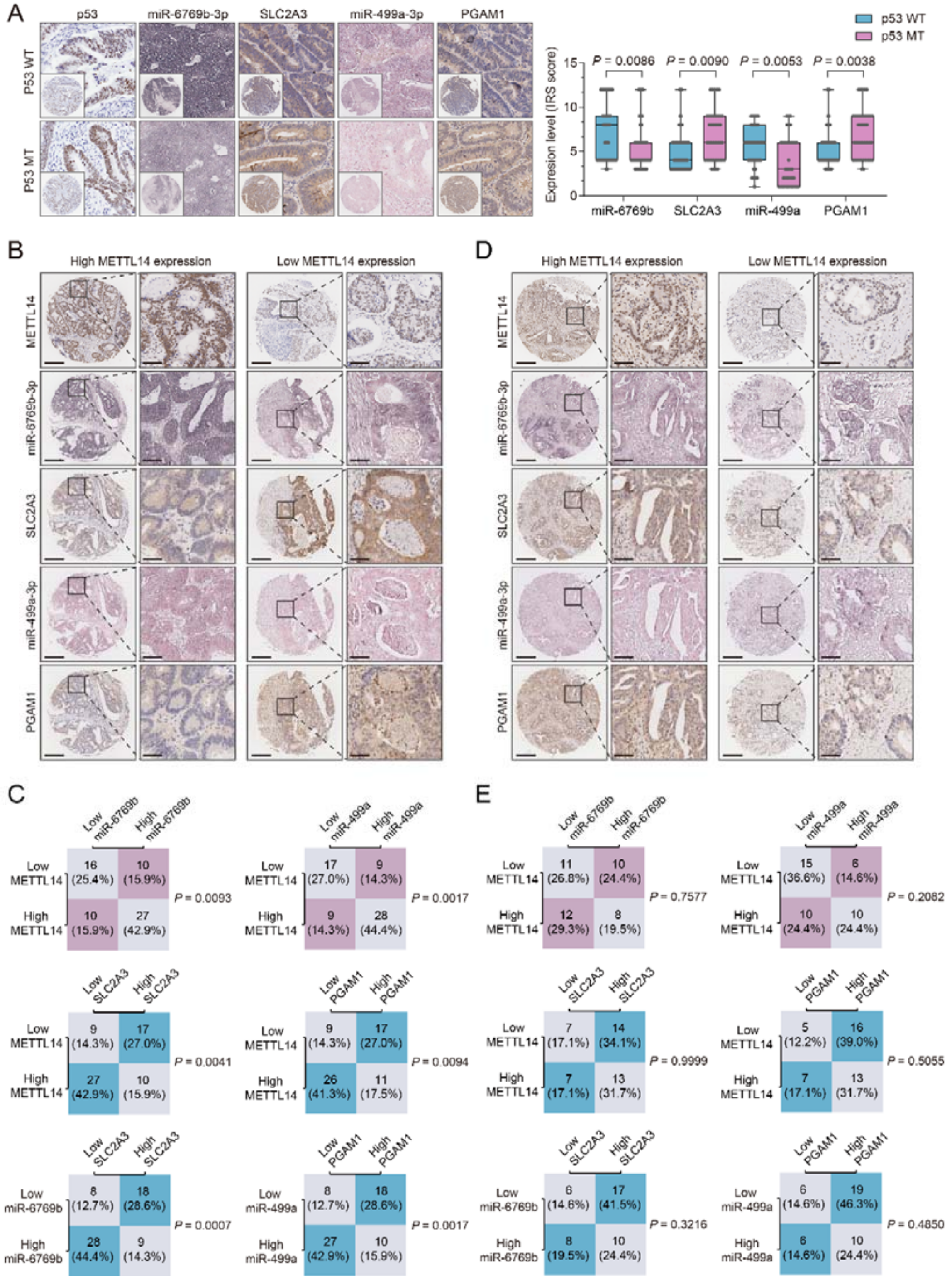

Figure 9 for reviewers

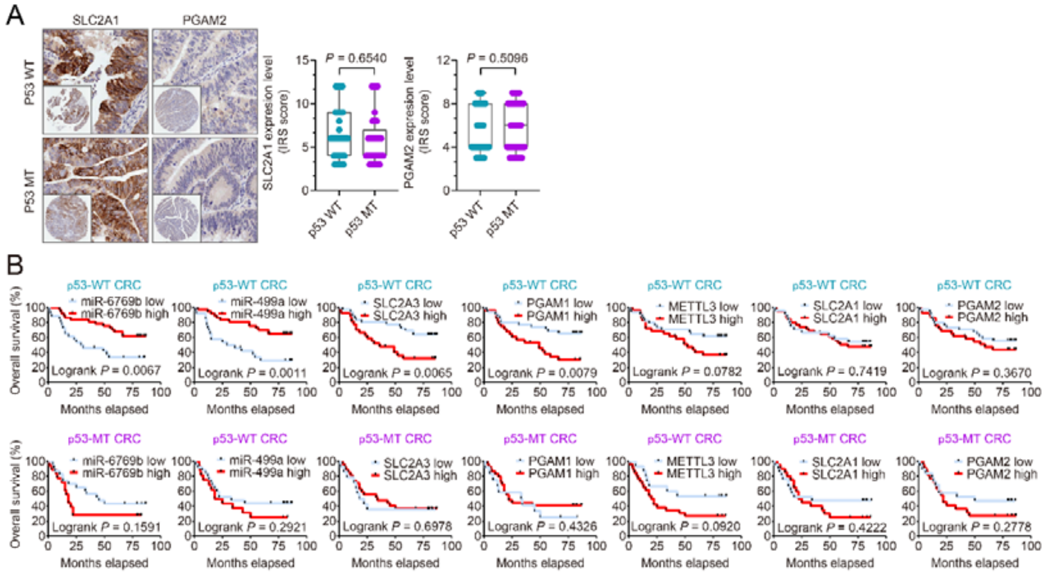

Major points 7: To my view, this manuscript contains a massive number of interesting and relevant results but it would need further refinement before being published in a comprehensive format.

Response:

Thank you for your insightful and rigorous comments. We also appreciate your professional comments and valuable suggestions. As a result, we removed the majority of data demonstrating that METTL14 is downregulated in CRC and a potential tumor suppressor gene suggested by editor and reviewers. To highlight the effect of METTL14 on glycolysis in p53-WT CRC, we change the **Title** to "METTL14 loss modulates glycolysis reprogramming to drive p53-wild type colorectal tumorigenesis". Correspondingly, we redescribed the **Abstract** section. In addition, we refine the manuscript by implemented some new experiments, and we also have reorganized the **Results** section. We believe that this revised manuscript could impress the readers more comprehensively with important information. Finally, we wish to thank the Reviewer for the insightful suggestions for additional experiments that helped to improve this manuscript.

References

Bian XL, Chen HZ, Yang PB, Li YP, Zhang FN, Zhang JY, Wang WJ, Zhao WX, Zhang S, Chen QT, Zheng Y, Sun XY, Wang XM, Chien KY, Wu Q (2017) Nur77 suppresses hepatocellular carcinoma via switching glucose metabolism toward gluconeogenesis through attenuating phosphoenolpyruvate carboxykinase sumoylation. Nature communications 8: 14420

Bieging-Rolett KT, Kaiser AM, Morgens DW, Boutelle AM, Seoane JA, Van Nostrand EL, Zhu C, Houlihan SL, Mello SS, Yee BA, McClendon J, Pierce SE, Winters IP, Wang M, Connolly AJ, Lowe SW, Curtis C, Yeo GW, Winslow MM, Bassik MC, Attardi LD (2020) Zmat3 Is a Key Splicing Regulator in the p53 Tumor Suppression Program. Molecular cell 80: 452-469 e459

Chen H, Gao S, Liu W, Wong CC, Wu J, Wu J, Liu D, Gou H, Kang W, Zhai J, Li C, Su H, Wang S, Soares F, Han J, He HH, Yu J (2021) RNA N(6)-Methyladenosine Methyltransferase METTL3

Facilitates Colorectal Cancer by Activating the m(6)A-GLUT1-mTORC1 Axis and Is a Therapeutic Target. Gastroenterology 160: 1284-1300 e1216

Cho MH, Park CK, Park M, Kim WK, Cho A, Kim H (2015) Clinicopathologic Features and Molecular Characteristics of Glucose Metabolism Contributing to ^{18}F-fluorodeoxyglucose Uptake in Gastrointestinal Stromal Tumors. PloS one 10: e0141413

Cui Q, Shi H, Ye P, Li L, Qu Q, Sun G, Sun G, Lu Z, Huang Y, Yang CG, Riggs AD, He C, Shi Y (2017) m(6)A RNA Methylation Regulates the Self-Renewal and Tumorigenesis of Glioblastoma Stem Cells. Cell reports 18: 2622-2634

DeBerardinis RJ, Lum JJ, Hatzivassiliou G, Thompson CB (2008) The biology of cancer: metabolic reprogramming fuels cell growth and proliferation. Cell metabolism 7: 11-20

Deng X, Su R, Weng H, Huang H, Li Z, Chen J (2018) RNA N(6)-methyladenosine modification in cancers: current status and perspectives. Cell research 28: 507-517

Eniafe J, Jiang S (2021) The functional roles of TCA cycle metabolites in cancer. Oncogene 40: 3351-3363

Hou Y, Zhang Q, Pang W, Hou L, Liang Y, Han X, Luo X, Wang P, Zhang X, Li L, Meng X (2021) YTHDC1-mediated augmentation of miR-30d in repressing pancreatic tumorigenesis via attenuation of RUNX1-induced transcriptional activation of Warburg effect. Cell death and differentiation 28: 3105-3124

Kato S, Han SY, Liu W, Otsuka K, Shibata H, Kanamaru R, Ishioka C (2003) Understanding the function-structure and function-mutation relationships of p53 tumor suppressor protein by high-resolution missense mutation analysis. Proceedings of the National Academy of Sciences of the United States of America 100: 8424-8429

Li L, Liang Y, Kang L, Liu Y, Gao S, Chen S, Li Y, You W, Dong Q, Hong T, Yan Z, Jin S, Wang T, Zhao W, Mai H, Huang J, Han X, Ji Q, Song Q, Yang C, Zhao S, Xu X, Ye Q (2018) Transcriptional Regulation of the Warburg Effect in Cancer by SIX1. Cancer cell 33: 368-385 e367

Liu J, Eckert MA, Harada BT, Liu SM, Lu Z, Yu K, Tienda SM, Chryplewicz A, Zhu AC, Yang Y, Huang JT, Chen SM, Xu ZG, Leng XH, Yu XC, Cao J, Zhang Z, Liu J, Lengyel E, He C (2018) m(6)A mRNA methylation regulates AKT activity to promote the proliferation and tumorigenicity of endometrial cancer. Nature cell biology 20: 1074-1083

Ma JZ, Yang F, Zhou CC, Liu F, Yuan JH, Wang F, Wang TT, Xu QG, Zhou WP, Sun SH (2017) METTL14 suppresses the metastatic potential of hepatocellular carcinoma by modulating N(6) -methyladenosine-dependent primary MicroRNA processing. Hepatology (Baltimore, Md) 65: 529-543

Mello SS, Valente LJ, Raj N, Seoane JA, Flowers BM, McClendon J, Bieging-Rolett KT, Lee J, Ivanochko D, Kozak MM, Chang DT, Longacre TA, Koong AC, Arrowsmith CH, Kim SK, Vogel H, Wood LD, Hruban RH, Curtis C, Attardi LD (2017) A p53 Super-tumor Suppressor Reveals a Tumor Suppressive p53-Ptpn14-Yap Axis in Pancreatic Cancer. Cancer cell 32: 460-473 e466

Meyers RM, Bryan JG, McFarland JM, Weir BA, Sizemore AE, Xu H, Dharia NV, Montgomery PG, Cowley GS, Pantel S, Goodale A, Lee Y, Ali LD, Jiang G, Lubonja R, Harrington WF, Strickland M, Wu T, Hawes DC, Zhivich VA, Wyatt MR, Kalani Z, Chang JJ, Okamoto M, Stegmaier K, Golub TR, Boehm JS, Vazquez F, Root DE, Hahn WC, Tsherniak A (2017) Computational correction of copy number effect improves specificity of CRISPR-Cas9 essentiality screens in cancer cells. Nature genetics 49: 1779-1784

Panneerdoss S, Eedunuri VK, Yadav P, Timilsina S, Rajamanickam S, Viswanadhapalli S,

Abdelfattah N, Onyeagucha BC, Cui X, Lai Z, Mohammad TA, Gupta YK, Huang TH, Huang Y, Chen Y, Rao MK (2018) Cross-talk among writers, readers, and erasers of m(6)A regulates cancer growth and progression. Science advances 4: eaar8263

Raj N, Wang M, Seoane JA, Zhao RL, Kaiser AM, Moonie NA, Demeter J, Boutelle AM, Kerr CH, Mulligan AS, Moffatt C, Zeng SX, Lu H, Barna M, Curtis C, Chang HY, Jackson PK, Attardi LD (2022) The Mettl3 epitranscriptomic writer amplifies p53 stress responses. Molecular cell 82: 2370-2384 e2310

Shen C, Xuan B, Yan T, Ma Y, Xu P, Tian X, Zhang X, Cao Y, Ma D, Zhu X, Zhang Y, Fang JY, Chen H, Hong J (2020) m(6)A-dependent glycolysis enhances colorectal cancer progression. Molecular cancer 19: 72

Vander Heiden MG, Cantley LC, Thompson CB (2009) Understanding the Warburg effect: the metabolic requirements of cell proliferation. Science (New York, NY) 324: 1029-1033

Yang Z, Yang S, Cui YH, Wei J, Shah P, Park G, Cui X, He C, He YY (2021) METTL14 facilitates global genome repair and suppresses skin tumorigenesis. Proceedings of the National Academy of Sciences of the United States of America 118

1st Revision - Editorial Decision **29th Nov 2022**

Dear Prof. Meng,

Thank you for the submission of your revised manuscript to our editorial offices. I have now received the reports from the two referees that I asked to re-evaluate your study, you will find below. As you will see, both referees think that the study has significantly improved and should be published in EMBO reports. Nevertheless, both referees have remaining points and suggestions to improve the study I ask you to address in a final revised manuscript. Please also provide a final detailed p-b-p-response addressing all the remaining referee comments.

Moreover, I have these editorial requests I ask you to address:

- Please have your final manuscript file carefully proofread by a native speaker.

- Please provide the abstract written in present tense throughout and with not more than 175 words.

- We now use CRediT to specify the contributions of each author in the journal submission system. CRediT replaces the author contribution section. Please use the free text box to provide more detailed descriptions. Thus, please remove the author contributions section from the manuscript text file. See also guide to authors: https://www.embopress.org/page/journal/14693178/authorguide#authorshipguidelines

- Please remove the 'Competing Interest Statement' from the main manuscript text file and just keep the 'Disclosure and Competing Interests Statement'.

- Please make sure that all the funding information is also entered into the online submission system and that it is complete and similar to the one in the acknowledgement section of the manuscript text file.

- Please make sure that the number "n" for how many independent experiments were performed, their nature (biological versus technical replicates), the bars and error bars (e.g. SEM, SD) and the test used to calculate p-values is indicated in the respective figure legends (main, EV and Appendix figures), and that statistical testing has been done where applicable. Please avoid phrases like 'independent experiment', but clearly state if these were biological or technical replicates. Please add complete statistical testing to all diagrams (main, EV and Appendix figures). Please also indicate (e.g. with n.s.) if testing was performed, but the differences are not significant. In case n=2, please show the data as separate datapoints without error bars and statistics. If n<5, please show single datapoints for diagrams.

- Please indicate in all figure panels with microscopic images with magnifying boxes their origin. This is presently missing for panels 2D, 3I, 4I, 6A, EV2F, EV3I, S4 and S6. Please mark in the main image where the box comes from (as in 1J or 6B).

- Please make sure that all figure panels are called out separately and sequentially (main, EV and Appendix figures). Presently, there seem to be no callouts for panels 1J and 4D, and no separate callouts for Appendix Figs. S5 and S7. Please check.

- Please name the Appendix file 'Appendix' and a dd a phrase to the title page ('Appendix for ...').

- Thanks for providing the source data (SD). Please upload this as one pdf file per figure (main and EV figures) or as one pdf file for the Appendix SD.

- Please format the reference according to our journal style (we need 'et al' for > 10 authors): http://www.embopress.org/page/journal/14693178/authorguide#referencesformat

- Please provide the author checklist with a completed section 'Dual Use Research of Concern (DURC)'.

- Please note that all corresponding authors are required to supply an ORCID ID for their name upon submission of a revised manuscript. Please ask co-corresponding author Jing-Yuan Fang to do that. Please find instructions on how to link the ORCID ID to the account in our manuscript tracking system in our Author guidelines: http://www.embopress.org/page/journal/14693178/authorguide#authorshipguidelines

- Finally, please find attached a word file of the manuscript text (provided by our publisher) with changes we ask you to include in your final manuscript text. Please use the attached file as basis for further revisions and provide your final manuscript file with track changes, in order that we can see any modifications done.

In addition, I would need from you:
- a short, two-sentence summary of the manuscript (not more than 35 words).
- two to four short bullet points highlighting the key findings of your study (two lines each).
- a schematic summary figure (in jpeg or tiff format with the exact width of 550 pixels and a height of not more than 400 pixels)

that can be used as a visual synopsis on our website.

I look forward to seeing the final revised version of your manuscript when it is ready. Please let me know if you have questions regarding the revision.

Please use this link to submit your revision: https://embor.msubmit.net/cgi-bin/main.plex

Best,

Achim Breiling
Senior Editor
EMBO Reports

Referee #1:

As I said in my previous review, the manuscript by Hou et al is very interesting and includes a massive amount of relevant information. The revised version of the manuscript is substantially improved compared with previous versions and the message is now clearer. However, I need to mention some remaining issues that should be improved before publication.

In particular, about the fact that cells with a metabolic bias towards glycolysis (and despite the exhaustive explanation in the rebuttal letter on what Otto Warburg demonstrated in 1920), I still don't understand that changing the metabolisms towards glycolysis results in higher production of ATP, being mitochondrial respiration 18 times more efficient in ATP production that glycolysis. I understand that specific cellular adaptation may result in a massive incorporation of glucose thus leading to high ATP production even in the presence of aerobic glycolytic metabolism (high glucose consumption is in fact used in the clinics for tumor detection). And the opposite can be also true. However, based on Figure 3C the efficiency of ATP production in METTL14-edited cells (OE or SH) is absolutely the same than in METTL14 controls (when glucose consumption is up or down, lactate and ATP production are modified at a comparable extent). Authors should consider revising this section and provide a better explanation for the unmodified Glucose/ATP ratio observed in METTL14 edited cells. Cells with a preferential use of glycolysis should be less efficient in ATP production (unless they use sources of energy other than glucose).

Also related with this, authors mention that cancer cells prefer glycolysis for obtaining the energy, but I should disagree with this sentence as it is reasonably established that tumor cells use glycolysis not only to obtain ATP, which could be obtained much easier in the mitochondria, but also macromolecules and other elements required for the generation of new cells in the context of a rapid growth.

As a very minor issue, some figures contain the label Mettle14, which is probably a mistake.

Referee #2:

This manuscript is very much improved from prior versions. The Authors have successfully organized the data and explained it in such a way that the story makes sense, and I agree that their identification of new vulnerabilities in p53 WT colorectal cancer can have an impact on the field. I have a few relatively minor technical points that must be addressed below, and there are two Figure panels I do not understand, but overall I am enthusiastic about this manuscript.

• Throughout the manuscript, the Authors compare p53 WT and p53 mutant cell lines. So that readers do not have to guess which ones are which, the Authors should label in each Figure which cell lines are p53 WT, which ones are p53 Mut, and which mutation they have. They did this in Figure 4, they should extend it to the whole paper.
• The GSEA results in Figure 3A are not presented accurately. First, GSEA uses an FDR.Q cutoff of 0.25, not 0.05 (see https://software.broadinstitute.org/cancer/software/gsea/wiki/index.php/FAQ), so it is not correct to say that glycolytic and apoptotic signatures were significantly enriched in p53 WT but not p53 Mut. Second, GSEA tests for both positive and negative enrichment. The Authors should indicate which way each set was enriched, and add the NES to their table in 3A.
• I do not understand what the Authors are trying to present in Figure 3E. The Authors state that, "METTL14 downregulation resulted in significant alternation of glucose transporters, glycolytic enzymes and glycolysis regulators in p53-WT CRC cells", but the only ones that look significant to me are the three glucose transporters on the top. If other genes were significantly changed, the Authors should show these data a different way and also show their statistical tests.
• Figure 4D is not mentioned in the text.
• Figure 6G is not presented in a way I can understand. The Authors want to stratify survival based on p53 status and METTL14 expression, but I cannot tell from reading the graph how to determine what METTL14 expression is, so I cannot evaluate this Figure.
• On line 491, the Authors incorrectly list SLC4A3, where I believe they mean to list SLC2A3.

Response to Editor and Reviewers Letter

Responses to editors' and reviewers' comments on the manuscript submitted by Hou *et al.*, "*METTL14 loss modulates glycolysis reprogramming to drive p53-wild type colorectal tumorigenesis*" (EMBOR-2022-56325V2)

We thank reviewers for providing helpful comments. We have addressed all the issues pointed out by reviewers and editor below. We hope that the revised manuscript has been improved enough to be published in EMBO reports. Any changes made in accordance with reviewers' comments are highlighted in red in the revised manuscript.

Page

Comments from Editors…....................................1-4

Comments from Reviewer 1....................................4-5

Comments from Reviewer 2....................................5-8

Editorial comments:

General comment: Thank you for the submission of your revised manuscript to our editorial offices. I have now received the reports from the two referees that I asked to re-evaluate your study, you will find below. As you will see, both referees think that the study has significantly improved and should be published in EMBO reports. Nevertheless, both referees have remaining points and suggestions to improve the study I ask you to address in a final revised manuscript. Please also provide a final detailed p-b-p-response addressing all the remaining referee comments.

Response:

Thank you. We appreciate the editor and reviewers for their constructive and valuable comments. We have revised our manuscript according to the editor' and reviewers' comments, questions, and suggestions.

Comment 1: Please have your final manuscript file carefully proofread by a native speaker.

Response:

We accept your thoughtful and professional comment and suggestion. We had revised language mistakes. At the same time, the article has been edited and revised carefully by a biologist from America.

Comment 2: Please provide the abstract written in present tense throughout and with not more than 175 words.

Response:

OK! We have made modifications as required.

Comment 3: We now use CRediT to specify the contributions of each author in the journal submission system. CRediT replaces the author contribution section. Please use the free text box to provide more detailed descriptions. Thus, please remove the author contributions section from the manuscript text file. See also guide to authors:
https://www.embopress.org/page/journal/14693178/authorguide#authorshipguidelines

Response:

Thanks for your reminder. We have removed the author contributions section from the manuscript text file. Meanwhile, we used the free text box to provide more detailed descriptions as requested.

Comment 4: Please remove the 'Competing Interest Statement' from the main manuscript text file and just keep the 'Disclosure and Competing Interests Statement'.

Response:

As suggested, we have removed the 'Competing Interest Statement' from the main manuscript and just keep the 'Disclosure and Competing Interests Statement'.

Comment 5: Please make sure that all the funding information is also entered into the online submission system and that it is complete and similar to the one in the acknowledgement section of the manuscript text file.

Response:

OK! We have made the correction in the acknowledgement section of the manuscript.

Comment 6: Please make sure that the number "n" for how many independent experiments were performed, their nature (biological versus technical replicates), the bars and error bars (e.g. SEM, SD) and the test used to calculate p-values is indicated in the respective figure legends (main, EV and Appendix figures), and that statistical testing has been done where applicable. Please avoid phrases like 'independent experiment', but clearly state if these were biological or technical replicates. Please add complete statistical testing to all diagrams (main, EV and Appendix figures). Please also indicate (e.g. with n.s.) if testing was performed, but the differences are not significant. In case n=2, please show the data as separate datapoints without error bars and statistics. If n<5, please show single datapoints for diagrams.

Response:

Thank you very much for your insightful suggestion. According to your comment, we have made the modifications in figure legends throughout the manuscript.

Comment 7: Please indicate in all figure panels with microscopic images with magnifying boxes their origin. This is presently missing for panels 2D, 3I, 4I, 6A, EV2F, EV3I, S4 and S6. Please mark in the main image where the box comes from (as in 1J or 6B).

Response:

Thank you for your detailed review. We have made modifications as required.

Comment 8: Please make sure that all figure panels are called out separately and sequentially (main, EV and Appendix figures). Presently, there seem to be no callouts for panels 1J and 4D, and no separate callouts for Appendix Figs. S5 and S7. Please check.

Response:

We apologize for the inaccuracy and have now corrected them throughout the manuscript.

Comment 9: Please name the Appendix file 'Appendix' and add a phrase to the title page ('Appendix for ...').

Response:

As requested, we make corresponding modifications.

Comment 10: Thanks for providing the source data (SD). Please upload this as one

pdf file per figure (main and EV figures) or as one pdf file for the Appendix SD.

Response:

OK! We have uploaded main and EV figures and Appendix figures as one PDF file, respectively.

Comment 11: Please format the reference according to our journal style (we need 'et al' for > 10 authors):
http://www.embopress.org/page/journal/14693178/authorguide#referencesformat

Response:

Yes, we format the reference according to the online guidelines.

Comment 12: Please provide the author checklist with a completed section 'Dual Use Research of Concern (DURC)'.

Response:

Thanks for your reminding. We completed section 'Dual Use Research of Concern (DURC)' in the author checklist.

Comment 13: Please note that all corresponding authors are required to supply an ORCID ID for their name upon submission of a revised manuscript. Please ask co-corresponding author Jing-Yuan Fang to do that. Please find instructions on how to link the ORCID ID to the account in our manuscript tracking system in our Author guidelines:
http://www.embopress.org/page/journal/14693178/authorguide#authorshipguidelines

Response:

Yes, we have supplied the ORCID ID of co-corresponding author Jing-Yuan Fang upon submission of revised manuscript according to the Author guidelines.

Comment 14: Finally, please find attached a word file of the manuscript text (provided by our publisher) with changes we ask you to include in your final manuscript text. Please use the attached file as basis for further revisions and provide your final manuscript file with track changes, in order that we can see any modifications done.

Response:

As suggested, we revised our manuscript in the word text from attached file.

Comment 15: In addition, I would need from you:
- a short, two-sentence summary of the manuscript (not more than 35 words).
- two to four short bullet points highlighting the key findings of your study (two lines each).
- a schematic summary figure (in jpeg or tiff format with the exact width of 550 pixels and a height of not more than 400 pixels) that can be used as a visual synopsis on our website.

Response:

We have uploaded a 'Synopsis' PDF file that includes a synopsis blurb and 4 bullet points as described below.

Synopsis blurb:

METTL14 specifically impedes p53-wild type colorectal tumorigenesis by attenuating aerobic glycolysis. METTL14 decreases SLC2A3 and PGAM1 expression via selectively promoting m^6A-YTHDF2-dependent pri-miR-6769b and pri-miR-499a

processing, and then biosynthesizing mature miR-6769b-3p and miR-499a-3p.
Bullet points:

1. METTL14 is transcriptionally activated by wild-type p53 and specifically inhibits progression of p53-wild type colorectal cancer.
2. METTL14-mediated m^6A modification selectively promotes the processing of pri-miR-6769b and pri-miR-499a depending on YTHDF2.
3. METTL14-regulated miR-499a-3p and miR-6769b-3p target SLC2A3 and PGAM1 respectively, thereby inhibiting glycolysis in p53-wild type colorectal cancer.
4. METTL14 expression inversely correlates with prognosis in p53-wild type colorectal cancer.

Reviewer: 1
Reviewer's comments:
General comment: As I said in my previous review, the manuscript by Hou et al is very interesting and includes a massive amount of relevant information. The revised version of the manuscript is substantially improved compared with previous versions and the message is now clearer. However, I need to mention some remaining issues that should be improved before publication.

Response:
Thank you. We have made the modifications according to your comment.

Comment 1: In particular, about the fact that cells with a metabolic bias towards glycolysis (and despite the exhaustive explanation in the rebuttal letter on what Otto Warburg demonstrated in 1920), I still don't understand that changing the metabolisms towards glycolysis results in higher production of ATP, being mitochondrial respiration 18 times more efficient in ATP production that glycolysis. I understand that specific cellular adaptation may result in a massive incorporation of glucose thus leading to high ATP production even in the presence of aerobic glycolytic metabolism (high glucose consumption is in fact used in the clinics for tumor detection). And the opposite can be also true. However, based on Figure 3C the efficiency of ATP production in METTL14-edited cells (OE or SH) is absolutely the same than in METTL14 controls (when glucose consumption is up or down, lactate and ATP production are modified at a comparable extent). Authors should consider revising this section and provide a better explanation for the unmodified Glucose/ATP ratio observed in METTL14 edited cells. Cells with a preferential use of glycolysis should be less efficient in ATP production (unless they use sources of energy other than glucose).

Response:
Thank you very much for your rigorous comments. Mounting evidence suggests that cancer cells engage in a unique metabolic program that allows for rapid cell proliferation. Nonproliferating cells can use glycolysis products to generate ATP for their energy needs. Such cells generally have low rates of glycolysis followed by oxidation of pyruvate in the mitochondria, leading to efficient generation of ATP. Dividing cells, in contrast, also use glycolysis intermediates for the synthesis of

macromolecules and must therefore balance their ATP requirements and biosynthetic needs (Israelsen & Vander Heiden, 2010). Metabolism of glucose by aerobic glycolysis, a phenomenon known as the Warburg effect, may help dividing cells strike this balance. In contrast to normal differentiated cells, which rely primarily on mitochondrial oxidative phosphorylation to generate the energy needed for cellular processes, most cancer cells instead rely on aerobic glycolysis, a phenomenon termed "the Warburg effect". Aerobic glycolysis is an inefficient way to generate adenosine 5'-triphosphate (ATP), and the advantage it confers to cancer cells has been unclear. Matthew G et al. (Vander Heiden *et al*, 2009) propose that the metabolism of cancer cells, and indeed all proliferating cells, is adapted to facilitate the uptake and incorporation of nutrients into the biomass (e.g., nucleotides, amino acids, and lipids) needed to produce a new cell. Supporting this idea are some studies showing that (i) several signaling pathways implicated in cell proliferation also regulate metabolic pathways that incorporate nutrients into biomass (DeBerardinis *et al*, 2008; Hsu & Sabatini, 2008); and that (ii) certain cancer-associated mutations enable cancer cells to acquire and metabolize nutrients in a manner conducive to proliferation rather than efficient ATP production (Parsons *et al*, 2008; Selak *et al*, 2005). In addition, you find that METTL14 does not affect Glucose/ATP ratio, which was similar to what were found in the previously published articles (Bian *et al*, 2017; Hou *et al*, 2021; Li *et al*, 2018). We failed to find any papers on the relationship between glycolysis and Glucose/ATP ratio in tumor cells. But we are sorry for incorrect description that METTL14 overexpression significantly reduced glucose uptake, lactate production, ATP and pyruvate levels while METTL14 knockdown led to the opposite results in p53-WT cells (Fig 3C and EV3D). Thereinto, it should be that METTL14 overexpression significantly reduced glucose uptake but that METTL14 overexpression did not significantly reduce glucose consumption. Thank you again for your valuable comments. In the future, our research group will focus on exploring why tumor cells prefer to select glycolytic pathways that produce less ATP to support their growth, as well as exploring the biological mechanism why METTL14 does not affect Glucose/ATP ratio. As suggested, we correct it in the Results section as follows "METTL14 overexpression significantly reduced glucose uptake, lactate production, ATP and pyruvate levels while METTL14 knockdown led to the opposite results in p53-WT cells (Fig 3C and EV3D)." (L245-247 in the revised manuscript)

Comment 2: Also related with this, authors mention that cancer cells prefer glycolysis for obtaining the energy, but I should disagree with this sentence as it is reasonably established that tumor cells use glycolysis not only to obtain ATP, which could be obtained much easier in the mitochondria, but also macromolecules and other elements required for the generation of new cells in the context of a rapid growth.

Response:

Thank you very much for your rigorous comments. In cancer cells, cancer cells display altered glucose metabolism characterized by a preference for aerobic glycolysis (Kwak *et al*, 2020). As suggested, we also rewrote it in the Introduction section as follows "Cancer cells display altered glucose metabolism characterized by

a preference for aerobic glycolysis even supplemented with abundant oxygen, the aberrant metabolism has been known as the Warburg effect, which promotes tumor progression with higher rate of glucose uptake and elevated ATP and lactate production as well as supervenient macromolecules and other elements required for the generation of new cells in the context of a rapid growth." (L76-81 in the revised manuscript)

Comment 3: As a very minor issue, some figures contain the label Mettle14, which is probably a mistake.

Response:

Thanks, it is corrected.

Reviewer: 2

Reviewer's comments:

General comment: This manuscript is very much improved from prior versions. The Authors have successfully organized the data and explained it in such a way that the story makes sense, and I agree that their identification of new vulnerabilities in p53 WT colorectal cancer can have an impact on the field. I have a few relatively minor technical points that must be addressed below, and there are two Figure panels I do not understand, but overall, I am enthusiastic about this manuscript.

Response:

Thank you. We have made the modifications as suggested.

Comment 1: Throughout the manuscript, the Authors compare p53 WT and p53 mutant cell lines. So that readers do not have to guess which ones are which, the Authors should label in each Figure which cell lines are p53 WT, which ones are p53 Mut, and which mutation they have. They did this in Figure 4, they should extend it to the whole paper.

Response:

Thank you for your detailed review and we accept your suggestion. We have now appended labels of p53-WT or p53-MT CRC cells in mentioned figures.

Comment 2: The GSEA results in Figure 3A are not presented accurately. First, GSEA uses an FDR.Q cutoff of 0.25, not 0.05 (see https://software.broadinstitute.org/cancer/software/gsea/wiki/index.php/FAQ), so it is not correct to say that glycolytic and apoptotic signatures were significantly enriched in p53 WT but not p53 Mut. Second, GSEA tests for both positive and negative enrichment. The Authors should indicate which way each set was enriched, and add the NES to their table in 3A.

Response:

We accept your thoughtful and professional comments and suggestions. As reviewer noted, GSEA uses an FDR.Q cutoff of 0.25, not 0.05. Previously, we've actually noticed this issue, and GSEA analysis was indeed carried out in accordance with such requirements. We selected the signature of FDR.Q cutoff < 0.25 obtained by GSEA enrichment and visualized these GSEA enrichment results by Cytoscape software. Under GSEA 4.3.3, there is an enrichment map visualization option. Under this feature option, you can set enrichment map parameters including p-value cutoff or

FDR Q-value cutoff for further visualization analysis. Some of the relevant results (panels D, Figure 4 for reviewers) had been shown in the last cycle of response to reviewer letter. In addition, we specified which way each set was enriched, and add the normalized enrichment scores (NES) to their table in Figure 3A. As suggested, we have deleted the inaccurate description "FDR of 0.25 was set as cut-off value for the determination of biologically relevant genes. Gene sets showing FDR more than 0.25 were considered enriched under comparison", but this does not affect the GSEA analysis results. Moreover, we redescribed the figure legend in Figure 3A as follows "Positive or negative normalized enrichment scores (NES) correspond to enrichment of a given set in genes that are up- or down-regulated, respectively, in response to METTL14 high expression. Data are presented as pseudo-heatmap with NES magnitude color-coded as indicated in the legend; all light red or non-gray comparisons have False Discovery Rate q-value (FDR q) < 0.05."

Comment 3: I do not understand what the Authors are trying to present in Figure 3E. The Authors state that, "METTL14 downregulation resulted in significant alternation of glucose transporters, glycolytic enzymes and glycolysis regulators in p53-WT CRC cells", but the only ones that look significant to me are the three glucose transporters on the top. If other genes were significantly changed, the Authors should show these data a different way and also show their statistical tests.

Response:

Thank you very much for your rigorous comments and insightful suggestion. The previous heatmap did not distinguish the differentially expressed genes very clearly. As requested, we redrew the heatmap and redefined significant differences of glucose transporters, glycolytic enzymes and glycolysis regulators between shNC group and shMETTL14 group using Adjusted p-value instead of P value. As suggested, we redescribed the figure legends in Figure 3E as follows "Significantly differentially expressed genes were identified by DESeq2 under the requirement of Adjusted p-value (adj.P.Val) < 0.05, 0.001 < adj.P.Val < 0.01 or adj.P.Val < 0.001." Additionally, we rewrote it in the **Results** section as follows "Next, the heat-map clustering analysis showed that METTL14 downregulation resulted in significant alternation of glucose transporters and glycolytic enzymes in p53-WT CRC cells (Fig 3E)." (L250-253 in the revised manuscript)

Comment 4: Figure 4D is not mentioned in the text.

Response:

We apologize for the inaccuracy and have now corrected it in the manuscript.

Comment 5: Figure 6G is not presented in a way I can understand. The Authors want to stratify survival based on p53 status and METTL14 expression, but I cannot tell from reading the graph how to determine what METTL14 expression is, so I cannot evaluate this Figure.

Response:

Thank you for your rigorous comments. First, we divided tumor patients diagnosed with the same tumor into patients with wild-type p53, patients with mutant p53 and patients regardless of p53 status. Second, according to optimal cutoff values, patients in three groups were divided into a high-expression METTL14 group and a

low-expression METTL14 group, respectively. The optimal cutoff values are defined as the ones with the most significant (likelihood ratio test or log-rank test) split (Chang *et al*, 2017; Vinh-Hung *et al*, 2009). Survival differences between the low-expression METTL14 group and high-expression METTL14 group in each set were assessed by the Kaplan-Meier estimate, and compared using the log-rank test. To make the results more understandable, we rewrote it in the figure legends in Figure 6G as follows "First, we divided tumor patients diagnosed with the same tumor into patients with wild-type p53, patients with mutant p53 and patients regardless of p53 status. Second, according to optimal cutoff values, patients in three groups were divided into a high-expression METTL14 group and a low-expression METTL14 group, respectively. Finally, Survival differences between the low-expression METTL14 group and high-expression METTL14 group in each set were assessed by the Kaplan-Meier estimate, and compared using the log-rank test."

Comment 6: On line 491, the Authors incorrectly list SLC4A3, where I believe they mean to list SLC2A3.

Response:

We apologize for the inaccuracy and have now corrected all mis-spellings within the manuscript.

References

Bian XL, Chen HZ, Yang PB, Li YP, Zhang FN, Zhang JY, Wang WJ, Zhao WX, Zhang S, Chen QT et al (2017) Nur77 suppresses hepatocellular carcinoma via switching glucose metabolism toward gluconeogenesis through attenuating phosphoenolpyruvate carboxykinase sumoylation. Nature communications 8: 14420

Chang C, Hsieh MK, Chang WY, Chiang AJ, Chen J (2017) Determining the optimal number and location of cutoff points with application to data of cervical cancer. PloS one 12: e0176231

DeBerardinis RJ, Lum JJ, Hatzivassiliou G, Thompson CB (2008) The biology of cancer: metabolic reprogramming fuels cell growth and proliferation. Cell metabolism 7: 11-20

Hou Y, Zhang Q, Pang W, Hou L, Liang Y, Han X, Luo X, Wang P, Zhang X, Li L et al (2021) YTHDC1-mediated augmentation of miR-30d in repressing pancreatic tumorigenesis via attenuation of RUNX1-induced transcriptional activation of Warburg effect. Cell death and differentiation 28: 3105-3124

Hsu PP, Sabatini DM (2008) Cancer cell metabolism: Warburg and beyond. Cell 134: 703-707

Israelsen WJ, Vander Heiden MG (2010) ATP consumption promotes cancer metabolism. Cell 143: 669-671

Kwak CH, Jin L, Han JH, Han CW, Kim E, Cho M, Chung TW, Bae SJ, Jang SB, Ha KT (2020) Ilimaquinone Induces the Apoptotic Cell Death of Cancer Cells by Reducing Pyruvate Dehydrogenase Kinase 1 Activity. International journal of molecular sciences 21

Li L, Liang Y, Kang L, Liu Y, Gao S, Chen S, Li Y, You W, Dong Q, Hong T et al (2018) Transcriptional Regulation of the Warburg Effect in Cancer by SIX1. Cancer cell 33: 368-385.e367

Parsons DW, Jones S, Zhang X, Lin JC, Leary RJ, Angenendt P, Mankoo P, Carter H, Siu IM, Gallia GL et al (2008) An integrated genomic analysis of human glioblastoma multiforme. Science (New York, NY) 321: 1807-1812

Selak MA, Armour SM, MacKenzie ED, Boulahbel H, Watson DG, Mansfield KD, Pan Y, Simon MC,

Thompson CB, Gottlieb E (2005) Succinate links TCA cycle dysfunction to oncogenesis by inhibiting HIF-alpha prolyl hydroxylase. Cancer cell 7: 77-85

Vander Heiden MG, Cantley LC, Thompson CB (2009) Understanding the Warburg effect: the metabolic requirements of cell proliferation. Science (New York, NY) 324: 1029-1033

Vinh-Hung V, Verkooijen HM, Fioretta G, Neyroud-Caspar I, Rapiti E, Vlastos G, Deglise C, Usel M, Lutz JM, Bouchardy C (2009) Lymph node ratio as an alternative to pN staging in node-positive breast cancer. Journal of clinical oncology : official journal of the American Society of Clinical Oncology 27: 1062-1068

2nd Revision - Editorial Decision **17th Jan 2023**

Dear Prof. Meng,

Thank you for the submission of your further revised manuscript to our editorial offices. I now went through this and your p-b-p-response, and I consider the remaining referee concerns as adequately addressed.

Before we can proceed with formal acceptance, I have these further editorial requests:

- I would suggest this modified title:
METTL14 modulates glycolysis to inhibit colorectal tumorigenesis in p53-wild type cells

- Please find attached a final word file of the manuscript text with changes I ask you to include in your final manuscript text and comments. Please use the attached file as basis for further revisions. Please go through the changes and answer/address the comments.

- Please provide statistics for the data shown Fig. EV5I.

- Please upload all source data for one figure as one pdf per figure (main or EV figures) or (if there is more than one file) ZIPed together as one folder. The source data for the Appendix is fine.

I look forward to seeing the final revised version of your manuscript when it is ready. Please let me know if you have questions regarding the revision.

Please use this link to submit your revision: https://embor.msubmit.net/cgi-bin/main.plex

Best,

Achim Breiling
Senior Editor
EMBO Reports

Response to Editor Letter

Responses to editor' comments on the manuscript submitted by Hou *et al.*, "*METTL14 loss modulates glycolysis reprogramming to drive p53-wild type colorectal tumorigenesis*" (EMBOR-2022-56325V3)

Our sincere thanks to editor for providing helpful comments and improvement. We have addressed all the issues pointed out by editor below. We hope that the revised manuscript has been improved enough to be published in EMBO reports.

Page

Comments from Editor…..1

Editorial comments:

General comment: Thank you for the submission of your further revised manuscript to our editorial offices. I now went through this and your p-b-p-response, and I consider the remaining referee concerns as adequately addressed.

Response:

Thank you very much. We appreciate the editor for his valuable comments, helpful improvement and friendly modifications. We have revised our manuscript according to the editor' comments.

Comment 1: I would suggest this modified title:

METTL14 modulates glycolysis to inhibit colorectal tumorigenesis in p53-wild type cells.

Response:

We totally accept your thoughtful and professional suggestion.

Comment 2: Please find attached a final word file of the manuscript text with changes I ask you to include in your final manuscript text and comments. Please use the attached file as basis for further revisions. Please go through the changes and answer/address the comments.

Response:

OK! We have made modifications as required.

Comment 3: Please provide statistics for the data shown Fig. EV5I.

Response:

OK! We have provided statistics as required.

Comment 4: Please upload all source data for one figure as one pdf per figure (main or EV figures) or (if there is more than one file) ZIPed together as one folder. The source data for the Appendix is fine.

Response:

As suggested, we have uploaded all source data for one figure as one pdf per figure.

Finally, we appreciate the editor' kind and constructive suggestions to our manuscript.

Thank you for your further consideration of our manuscript.

Prof. Xiangjun Meng
Shanghai Nineth People's Hospital, School of Medicine, Shanghai Jiao Tong University
China

Dear Prof. Meng,

I am very pleased to accept your manuscript for publication in the next available issue of EMBO reports. Thank you for your contribution to our journal.

At the end of this email I include important information about how to proceed. Please ensure that you take the time to read the information and complete and return the necessary forms to allow us to publish your manuscript as quickly as possible.

As part of the EMBO publication's Transparent Editorial Process, EMBO reports publishes online a Review Process File to accompany accepted manuscripts. As you are aware, this File will be published in conjunction with your paper and will include the referee reports, your point-by-point response and all pertinent correspondence relating to the manuscript.

If you do NOT want this File to be published, please inform the editorial office within 2 days, if you have not done so already, otherwise the File will be published by default [contact: emboreports@embo.org]. If you do opt out, the Review Process File link will point to the following statement: "No Review Process File is available with this article, as the authors have chosen not to make the review process public in this case." Please note that the author checklist will still be published even if you opt out of the transparent process.

Thank you again for your contribution to EMBO reports and congratulations on a successful publication. Please consider us again in the future for your most exciting work.

Yours sincerely,

Achim Breiling
Editor
EMBO Reports

****

THINGS TO DO NOW:

Please note that you will be contacted by Wiley Author Services to complete licensing and payment information. The required 'Page Charges Authorization Form' is available here: https://www.embopress.org/pb-assets/embo-site/er_apc.pdf - please download and complete the form and return to embopressproduction@wiley.com

EMBO Press participates in many Publish and Read agreements that allow authors to publish Open Access with reduced/no publication charges. Check your eligibility: https://authorservices.wiley.com/author-resources/Journal-Authors/open-access/affiliation-policies-payments/index.html

You will receive proofs by e-mail approximately 2-3 weeks after all relevant files have been sent to our Production Office; you should return your corrections within 2 days of receiving the proofs.

Please inform us if there is likely to be any difficulty in reaching you at the above address at that time. Failure to meet our deadlines may result in a delay of publication, or publication without your corrections.

All further communications concerning your paper should quote reference number EMBOR-2022-56325V4 and be addressed to emboreports@wiley.com.

Should you be planning a Press Release on your article, please get in contact with emboreports@wiley.com as early as possible, in order to coordinate publication and release dates.

EMBO Press Author Checklist

Corresponding Author Name: Xiangjun Meng	
Journal Submitted to: EMBO reports	
Manuscript Number: EMBOR-2022-56325-V2	

USEFUL LINKS FOR COMPLETING THIS FORM
The EMBO Journal - Author Guidelines
EMBO Reports - Author Guidelines
Molecular Systems Biology - Author Guidelines
EMBO Molecular Medicine - Author Guidelines

Reporting Checklist for Life Science Articles (updated January 2022)
This checklist is adapted from Materials Design Analysis Reporting (MDAR) Checklist for Authors. MDAR establishes a minimum set of requirements in transparent reporting in the life sciences (see Statement of Task: 10.31222/osf.io/9sm4x). Please follow the journal's guidelines in preparing your manuscript.
Please note that a copy of this checklist will be published alongside your article.

Abridged guidelines for figures
1. Data
The data shown in figures should satisfy the following conditions:
- the data were obtained and processed according to the field's best practice and are presented to reflect the results of the experiments in an accurate and unbiased manner.
- ideally, figure panels should include only measurements that are directly comparable to each other and obtained with the same assay.
- plots include clearly labeled error bars for independent experiments and sample sizes. Unless justified, error bars should not be shown for technical replicates.
- if n<5, the individual data points from each experiment should be plotted. Any statistical test employed should be justified.
- Source Data should be included to report the data underlying figures according to the guidelines set out in the authorship guidelines on Data Presentation.

2. Captions
Each figure caption should contain the following information, for each panel where they are relevant:
- a specification of the experimental system investigated (eg cell line, species name).
- the assay(s) and method(s) used to carry out the reported observations and measurements.
- an explicit mention of the biological and chemical entity(ies) that are being measured.
- an explicit mention of the biological and chemical entity(ies) that are altered/varied/perturbed in a controlled manner.
- the exact sample size (n) for each experimental group/condition, given as a number, not a range;
- a description of the sample collection allowing the reader to understand whether the samples represent technical or biological replicates (including how many animals, litters, cultures, etc.).
- a statement of how many times the experiment shown was independently replicated in the laboratory.
- definitions of statistical methods and measures:
  - common tests, such as t-test (please specify whether paired vs. unpaired), simple χ2 tests, Wilcoxon and Mann-Whitney tests, can be unambiguously identified by name only, but more complex techniques should be described in the methods section;
  - are tests one-sided or two-sided?
  - are there adjustments for multiple comparisons?
  - exact statistical test results, e.g., P values = x but not P values < x;
  - definition of 'center values' as median or average;
  - definition of error bars as s.d. or s.e.m.

Please complete ALL of the questions below.
Select "Not Applicable" only when the requested information is not relevant for your study.

Materials

Newly Created Materials	Information included in the manuscript?	In which section is the information available? (Reagents and Tools Table, Materials and Methods, Figures, Data Availability Section)
New materials and reagents need to be available; do any restrictions apply?	Not Applicable	

Antibodies	Information included in the manuscript?	In which section is the information available? (Reagents and Tools Table, Materials and Methods, Figures, Data Availability Section)
For **antibodies** provide the following information:<br>- Commercial antibodies: RRID (if possible) or supplier name, catalogue number and or/clone number<br>- Non-commercial: RRID or citation	Yes	Materials and Methods

DNA and RNA sequences	Information included in the manuscript?	In which section is the information available? (Reagents and Tools Table, Materials and Methods, Figures, Data Availability Section)
**Short novel DNA or RNA including primers, probes**: provide the sequences.	Yes	Appendix Table S1, 2, 3

Cell materials	Information included in the manuscript?	In which section is the information available? (Reagents and Tools Table, Materials and Methods, Figures, Data Availability Section)
**Cell lines:** Provide species information, strain. Provide accession number in repository **OR** supplier name, catalog number, clone number, and/**OR** RRID.	Yes	Materials and Methods
**Primary cultures:** Provide species, strain, sex of origin, genetic modification status.	Not Applicable	
Report if the cell lines were recently **authenticated** (e.g., by STR profiling) and tested for mycoplasma contamination.	Yes	Materials and Methods

Experimental animals	Information included in the manuscript?	In which section is the information available? (Reagents and Tools Table, Materials and Methods, Figures, Data Availability Section)
**Laboratory animals or Model organisms:** Provide species, strain, sex, age, genetic modification status. Provide accession number in repository **OR** supplier name, catalog number, clone number, **OR** RRID.	Yes	Materials and Methods
**Animal observed in or captured from the field:** Provide species, sex, and age where possible.	Not Applicable	
Please detail **housing and husbandry conditions**.	Yes	Materials and Methods

Plants and microbes	Information included in the manuscript?	In which section is the information available? (Reagents and Tools Table, Materials and Methods, Figures, Data Availability Section)
**Plants:** provide species and strain, ecotype and cultivar where relevant, unique accession number if available, and source (including location for collected wild specimens).	Not Applicable	
**Microbes:** provide species and strain, unique accession number if available, and source.	Not Applicable	

Human research participants	Information included in the manuscript?	In which section is the information available? (Reagents and Tools Table, Materials and Methods, Figures, Data Availability Section)
If collected and within the bounds of privacy constraints report on age, sex and gender or ethnicity for all study participants.	Yes	Dataset EV3

Core facilities	Information included in the manuscript?	In which section is the information available? (Reagents and Tools Table, Materials and Methods, Figures, Data Availability Section)
If your work benefited from core facilities, was their service mentioned in the acknowledgments section?	Yes	ACKNOWLEDGEMENTS

Design

Study protocol	Information included in the manuscript?	In which section is the information available? (Reagents and Tools Table, Materials and Methods, Figures, Data Availability Section)
If study protocol has been **pre-registered, provide DOI in the manuscript**. For clinical trials, provide the trial registration number **OR** cite DOI.	Not Applicable	
Report the **clinical trial registration number** (at ClinicalTrials.gov or equivalent), where applicable.	Not Applicable	

Laboratory protocol	Information included in the manuscript?	In which section is the information available? (Reagents and Tools Table, Materials and Methods, Figures, Data Availability Section)
Provide DOI OR other citation details if **external detailed step-by-step protocols** are available.	Not Applicable	

Experimental study design and statistics	Information included in the manuscript?	In which section is the information available? (Reagents and Tools Table, Materials and Methods, Figures, Data Availability Section)
Include a statement about **sample size** estimate even if no statistical methods were used.	Yes	Materials and Methods
Were any steps taken to minimize the effects of subjective bias when allocating animals/samples to treatment (e.g. **randomization procedure**)? If yes, have they been described?	Yes	Materials and Methods
Include a statement about **blinding** even if no blinding was done.	Not Applicable	
Describe **inclusion/exclusion criteria** if samples or animals were excluded from the analysis. Were the criteria pre-established?<br><br>If sample or data points were omitted from analysis, report if this was due to attrition or intentional exclusion and provide justification.	Not Applicable	
For every figure, are **statistical tests** justified as appropriate? Do the data meet the assumptions of the tests (e.g., normal distribution)? Describe any methods used to assess it. Is there an estimate of variation within each group of data? Is the variance similar between the groups that are being statistically compared?	Yes	Materials and Methods

Sample definition and in-laboratory replication	Information included in the manuscript?	In which section is the information available? (Reagents and Tools Table, Materials and Methods, Figures, Data Availability Section)
In the figure legends: state number of times the experiment was **replicated** in laboratory.	Yes	Figure legends
In the figure legends: define whether data describe **technical or biological replicates**.	Yes	Figure legends

Ethics

Ethics	Information included in the manuscript?	In which section is the information available? (Reagents and Tools Table, Materials and Methods, Figures, Data Availability Section)
Studies involving **human participants**: State details of **authority granting ethics approval** (IRB or equivalent committee(s), provide reference number for approval.	Not Applicable	
Studies involving **human participants**: Include a statement confirming that **informed consent** was obtained from all subjects and that the experiments conformed to the principles set out in the WMA Declaration of Helsinki and the Department of Health and Human Services Belmont Report.	Not Applicable	
Studies involving **human participants:** For publication of **patient photos**, include a statement confirming that consent to publish was obtained.	Not Applicable	
Studies involving experimental **animals**: State details of **authority granting ethics approval** (IRB or equivalent committee(s), provide reference number for approval. Include a statement of compliance with ethical regulations.	Yes	Materials and Methods
Studies involving **specimen and field samples:** State if relevant **permits** obtained, provide details of authority approving study; if none were required, explain why.	Yes	Materials and Methods

Dual Use Research of Concern (DURC)	Information included in the manuscript?	In which section is the information available? (Reagents and Tools Table, Materials and Methods, Figures, Data Availability Section)
Could your study fall under dual use research restrictions? Please check biosecurity documents and list of **select agents and toxins** (CDC): https://www.selectagents.gov/sat/list.htm	Not Applicable	
If you used a select agent, is the security level of the lab appropriate and reported in the manuscript?	Not Applicable	
If a study is subject to dual use research of concern regulations, is the name of the **authority granting approval and reference number** for the regulatory approval provided in the manuscript?	Not Applicable	

Reporting

The MDAR framework recommends adoption of discipline-specific guidelines, established and endorsed through community initiatives. Journals have their own policy about requiring specific guidelines and recommendations to complement MDAR.

Adherence to community standards	Information included in the manuscript?	In which section is the information available? (Reagents and Tools Table, Materials and Methods, Figures, Data Availability Section)
State if relevant guidelines or checklists (e.g., **ICMJE, MIBBI, ARRIVE, PRISMA**) have been followed or provided.	Not Applicable	
For **tumor marker prognostic studies,** we recommend that you follow the **REMARK** reporting guidelines (see link list at top right). See author guidelines, under 'Reporting Guidelines'. Please confirm you have followed these guidelines.	Not Applicable	
For **phase II and III randomized controlled trials**, please refer to the **CONSORT** flow diagram (see link list at top right) and submit the CONSORT checklist (see link list at top right) with your submission. See author guidelines, under 'Reporting Guidelines'. Please confirm you have submitted this list.	Not Applicable	

Data Availability

Data availability	Information included in the manuscript?	In which section is the information available? (Reagents and Tools Table, Materials and Methods, Figures, Data Availability Section)
Have **primary datasets** been deposited according to the journal's guidelines (see 'Data Deposition' section) and the respective accession numbers provided in the Data Availability Section?	Yes	Data Availability Section
Were **human clinical and genomic datasets** deposited in a public access-controlled repository in accordance to ethical obligations to the patients and to the applicable consent agreement?	Not Applicable	
Are **computational models** that are central and integral to a study available without restrictions in a machine-readable form? Were the relevant accession numbers or links provided?	Not Applicable	
If publicly available data were reused, provide the respective **data citations in the reference list**.	Not Applicable	
